# Impact of COVID-19 restrictions on diabetes health checks and prescribing for people with type 2 diabetes: a UK-wide cohort study involving 618 161 people in primary care

**DOI:** 10.1136/bmjqs-2021-013613

**Published:** 2021-10-12

**Authors:** Matthew J Carr, Alison K Wright, Lalantha Leelarathna, Hood Thabit, Nicola Milne, Naresh Kanumilli, Darren M Ashcroft, Martin K Rutter

**Affiliations:** 1 Centre for Pharmacoepidemiology and Drug Safety, Division of Pharmacy and Optometry, The University of Manchester, Manchester, UK; 2 NIHR Greater Manchester Patient Safety Translational Research Centre, University of Manchester, Manchester, UK; 3 Division of Diabetes, Endocrinology and Gastroenterology, School of Medical Sciences, The University of Manchester, Manchester, UK; 4 Diabetes, Endocrinology and Metabolism Centre, Manchester University NHS Foundation Trust, Manchester, UK

**Keywords:** COVID-19, diabetes mellitus, primary care, health services research

## Abstract

**Objective:**

To compare rates of performing National Institute for Health and Care Excellence-recommended health checks and prescribing in people with type 2 diabetes (T2D), before and after the first COVID-19 peak in March 2020, and to assess whether trends varied by age, sex, ethnicity and deprivation.

**Methods:**

We studied 618 161 people with T2D followed between March and December 2020 from 1744 UK general practices registered with the Clinical Practice Research Datalink. We focused on six health checks: haemoglobin A1c, serum creatinine, cholesterol, urinary albumin excretion, blood pressure and body mass index assessment. Regression models compared observed rates in April 2020 and between March and December 2020 with trend-adjusted expected rates derived from 10-year historical data.

**Results:**

In April 2020, in English practices, rates of performing health checks were reduced by 76%–88% when compared with 10-year historical trends, with older people from deprived areas experiencing the greatest reductions. Between May and December 2020, the reduced rates recovered gradually but overall remained 28%–47% lower, with similar findings in other UK nations. Extrapolated to the UK population, there were ~7.4 million fewer care processes undertaken March–December 2020. In England, rates for new medication fell during April with reductions varying from 10% (95% CI: 4% to 16%) for antiplatelet agents to 60% (95% CI: 58% to 62%) for antidiabetic medications. Overall, between March and December 2020, the rate of prescribing new diabetes medications fell by 19% (95% CI: 15% to 22%) and new antihypertensive medication prescribing fell by 22% (95% CI: 18% to 26%), but prescribing of new lipid-lowering or antiplatelet therapy was unchanged. Similar trends were observed across the UK, except for a reduction in new lipid-lowering therapy prescribing in the other UK nations (reduction: 16% (95% CI: 10% to 21%)). Extrapolated to the UK population, between March and December 2020, there were ~31 800 fewer people with T2D prescribed a new type of diabetes medication and ~14 600 fewer prescribed a new type of antihypertensive medication.

**Conclusions:**

Over the coming months, healthcare services will need to manage this backlog of testing and prescribing. We recommend effective communications to ensure patient engagement with diabetes services, monitoring and opportunities for prescribing, and when appropriate use of home monitoring, remote consultations and other innovations in care.

## Introduction

In 2008, the National Institute for Health and Care Excellence (NICE), serving the National Health Service (NHS) in England and Wales, recommended nine essential ‘health checks’ or so-called ‘care processes’ that define high-quality diabetes care.^1^ NICE recommended that people with diabetes should have at least annual checks of weight, blood pressure, smoking status, haemoglobin A1c (HbA1c), cholesterol, creatinine, urinary albumin, retinopathy and feet. NICE guidelines apply in England, Wales and Northern Ireland. In Scotland, diabetes guidelines recommend a similar system of health checks,^2^ and these are accepted measures of diabetes care quality worldwide.^3–5^ Since 2008, these health checks have been incorporated in National Diabetes Audits and have also been used effectively in the Quality Outcomes Framework (QOF) with increasing year-on-year rates of performing these checks, except for albuminuria testing.^6–8^ The QOF is a voluntary annual reward and incentive programme for all General Practitioners (GPs) in England and is designed to measure achievements and to reward good practice.

The COVID-19 pandemic has had major health and economic effects across the world. As of 12 August 2021, there have been more than 131 000 COVID-19-related deaths in the UK, with disproportionate impacts in people with diabetes; in the early phase of the pandemic, nearly one-third of all COVID-19-related deaths occurred in people with diabetes.^9–11^ The impact on the NHS, and in particular on diabetes services, has been enormous, with the suspension of much routine care. As the COVID-19 pandemic continues, there is an urgent need to minimise the harm done through reduction of routine services and to prioritise care and resources to areas of greatest need. The management of type 2 diabetes (T2D) occurs almost exclusively in primary care.^12^ Therefore, lower general practice attendance due to COVID-19 would likely restrict the ability to perform these essential health checks. Consequently, this could have adverse effects on patient safety and increase the risk of developing long-term diabetes-related complications.

We have recently shown the indirect consequences of the COVID-19 pandemic on diagnosis rates, HbA1c monitoring and mortality in T2D.^13^ However, there are limited data on the impact of the COVID-19 pandemic on diabetes health checks and prescribing in primary care. Therefore, we used a large primary care longitudinal dataset, broadly representative of the UK population, to compare the frequency of health checks and prescribing in people with T2D, before and after the first nationwide COVID-19 lockdown in March 2020. In UK-wide data, we compared observed and predicted rates using data covering 10 years prior to the pandemic. Since older people, minority ethnic groups and more socially disadvantaged people have been disproportionally affected by COVID-19 infections, and since the same groups may be more adversely impacted by changes in healthcare delivery imposed by COVID-19, we aimed to study variation in outcomes by age, sex, ethnicity and deprivation level.

## Methods

### Data sources

We conducted a retrospective cohort study using primary care electronic health records obtained from the Clinical Practice Research Datalink (CPRD) *Aurum* and *GOLD* databases.^13 14^ The CPRD contains anonymised consultation records and includes patient demographic information, symptoms, diagnoses, medication prescriptions and date of death. In CPRD *Aurum*, there were 1370 contributing general practices (15% of all UK general practices): 99% in England and 1% in Northern Ireland.^15^ Due to English general practices transitioning from the Vision GP system to the EMIS system, the number of English practices contributing to CPRD *GOLD* decreased over time. CPRD *GOLD* was composed of 394 contributing practices (4% of UK general practices) of which 14% were in England, 10% in Northern Ireland, 50% in Scotland and 26% in Wales.^15^ We also examined practice-level Index of Multiple Deprivation (IMD) quintiles,^14^ a measure representing an area’s relative level of deprivation, ranked within each UK nation.

Our primary analysis was conducted using *Aurum* data from English practices (covering 99% of contributing practices in the database). Analyses were replicated in *GOLD* providing information on practices in Northern Ireland, Scotland and Wales.

### Definitions, measurements and clinical coding

To enable comparisons of rates before and after the start of the COVID-19 outbreak, we included patient records from January 2010 to establish long-term trends and patterns of seasonality. We focused primarily on reporting observed versus expected rates from 1 March 2020 to 10 December 2020. We also chose to study rates of health checks and prescribing in April 2020 since this was the first full month following the national lockdown. For the diabetes monitoring component of the study, we restricted our investigation to the following six care processes because there was a high level of confidence that they had or had not been performed based on the available primary care records: HbA1c, serum creatinine, cholesterol, urinary albumin excretion, blood pressure, body mass index (BMI) measurement. It seemed possible that smoking status had been assessed and foot checks performed but not recorded in the practice records and therefore we did not report these outcomes. Several novel models of diabetes-related foot care have evolved during the pandemic^16 17^ and therefore we felt that primary care data alone might not adequately capture foot assessment activity. Eye screening for retinopathy is performed by optometrists that are not linked to GP practices and the results are not routinely captured in the primary care record.

For the medication prescribing component of the study, we focused on medications commonly prescribed to patients with a diagnosis of T2D: antidiabetics, antihypertensives, lipid-lowering drugs and antiplatelet agents. To compare prescribing behaviours before and after the start of the pandemic, we applied two distinct definitions: first, we assessed the prescribing of new medication within a 3-year window of a first diagnosis of T2D where new prescriptions were identified among those eligible to receive new treatment, that is, people within 3 years of diagnosis not currently or historically prescribed the medication of interest. Second, we assessed the overall prescribing rate (new and repeat) among patients with a prior diagnosis.

We also compared the incidence and event rates for eight separate strata by combining attributes of the study cohort via the dichotomisation of sex, age (less than 65 years vs greater than or equal to 65 years), and IMD (quintiles 4 and 5 (most deprived) vs quintiles 1, 2 and 3) as shown in online supplemental table 1.

10.1136/bmjqs-2021-013613.supp1 Supplementary data

Care processes were identified using Read/SNOMED/EMIS codes used in CPRD *GOLD* and *Aurum*. Medication prescribing events were identified using CPRD product codes linked to codes from the dictionary of medicines and devices. All medical and drug code lists used in the analyses are available as online supplemental appendices.

Ethnicity was classified from primary care and Hospital Episode Statistics (HES) records as white/Asian/black/mixed/other or white/other dependent on sample size. For patients with multiple records and conflicts, we defined ethnicity as (a) the category with most corresponding records; (b) according to the last available record when record numbers were tied across two or more categories; and (c) by randomly selecting between candidate ethnicities when record numbers were tied across two or more categories and multiple categories were entered in the last available record.

All code lists and medication lists were verified by two senior clinical academics (a diabetologist: MKR, and a senior academic pharmacist: DMA).

### Study design

For each patient, we defined a 'period of eligibility' for study inclusion which commenced on the latest of: the study start date (1 January 2010); the patient’s most recent registration with their practice; the date on which data from the practice were deemed to be ‘up-to-standard’ by the CPRD; the patient’s first diagnosis of T2D. A patient’s period of eligibility ended on the earliest of: registration termination; the end of data collection from their practice; death. We also applied a ‘look-back’ period during which a patient was required to have been registered for at least a year prior to their diagnosis of T2D. The denominator for the incidence and event rates was the aggregate person-months at risk for the whole eligible study cohort. With our open-cohort design, patients could enter or exit the cohort at any time during the study period depending on their date of registration, diagnosis and migration from their practice. As such, a given patient in a specific month could contribute a full or partial month of follow-up to the person-time denominator.

### Statistical analysis

The data were structured in a time-series format with event counts and ‘person-months at risk’ aggregated (by year and month) with stratification by sex, age group, ethnicity, deprivation quintile and region (or nation in *GOLD*). Mean-dispersion negative binomial regression models were used to estimate expected monthly event counts from March 2020 onward based on antecedent trends since 2010. The natural logarithm of the denominator (person-months at risk) was used as an offset in each regression model. To account for possible seasonality and long-term linear trends, calendar month was fitted as a categorical variable and time as a continuous variable with the number of months since the start of the study serving as the unit of measurement. For each month studied, observed and expected event counts were converted to rates using the observed person-month denominator. The monthly expected rates, and their 95% CIs, were plotted against the observed rates. As they share a common denominator, differences between expected and observed monthly rates are expressed as a percentage ‘rate reduction (or increase)’.

Although it was not possible to directly estimate the number of patients who missed out on a given care process, we estimated the shortfall in the number of care processes undertaken between March and December 2020 and the number of patients prescribed new medications.

All data processing and statistical analyses were conducted using Stata V.16 (StataCorp, College Station, Texas, USA). We followed REporting of studies Conducted using Observational Routinely-collected health Data (RECORD) guidance.^18^

## Results

### Study cohort

Our focus was on the impact of the COVID-19 pandemic between March and December 2020. Using the inclusion criteria described in the Study design section, a mixed cohort was used consisting of those patients from the study population whose period of eligibility began before 1 March 2020 and those who became eligible for inclusion between 1 March 2020 and 10 December 2020 (online supplemental figures 1 and 2). The study population consisted of 965 964 patients with a diagnosis of T2D: 824 698 patients from 1470 general practices in England, with a further 141 266 patients from 361 practices across Northern Ireland (16 408 patients in 40 practices), Scotland (69 935 patients in 208 practices), and Wales (54 923 patients in 113 practices). From the study population, 934 214 patients (from 1828 UK general practices) contributed to the estimation of the expected rates in the pre-COVID-19 period between January 2010 and February 2020. The study cohort that contributed to the comparisons of observed and expected rates between March and December 2020 comprised 618 161 people with T2D from 1744 UK general practices. The median (IQR) age was 68 (58–77) years, 44% were female and 25% lived in an area that was in the most deprived quintile compared with the rest of the UK.

Ethnicity data were available in 699 572 (85%) of those with T2D in England and 64 457 (46%) of those in Northern Ireland, Scotland and Wales with the following breakdown: England (white 80%, Asian 13%, black 6%, mixed 1%, other 0.4%); other UK nations (white 96%, Asian 3%, black 0.5%, mixed 0.2%, other 0.2%).

### Impacts of COVID-19 on care processes in T2D

**Figure 1:**
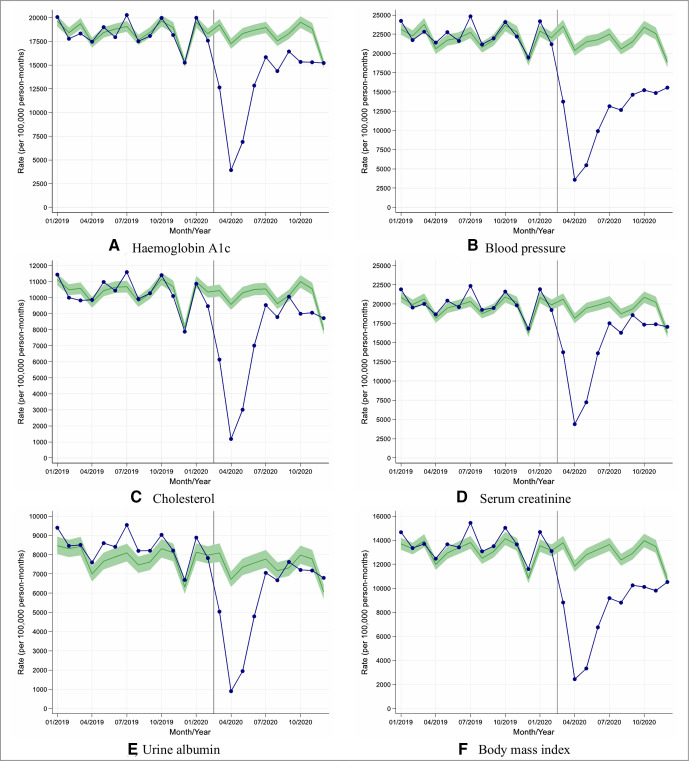
Observed and expected care process rates in people with type 2 diabetes during 2019 and 2020, in England. (A–F) Blue lines indicate observed monthly rates (years 2019 and 2020), and green-shaded regions indicate expected rates with 95% CIs based on 10-year historical trends from January 2010; the vertical line at 1 March 2020 separates the rates in primary care before and after the start of the COVID-19 pandemic, and x-axis markers indicate mid-months. Created by the authors.

In April 2020, in English primary care, the rate of performing health checks was reduced by 76%–88% when compared with 10-year historical trends (figure 1), with similar reductions (74%–88%) in other UK nations (online supplemental figure 3).

**Figure 2:**
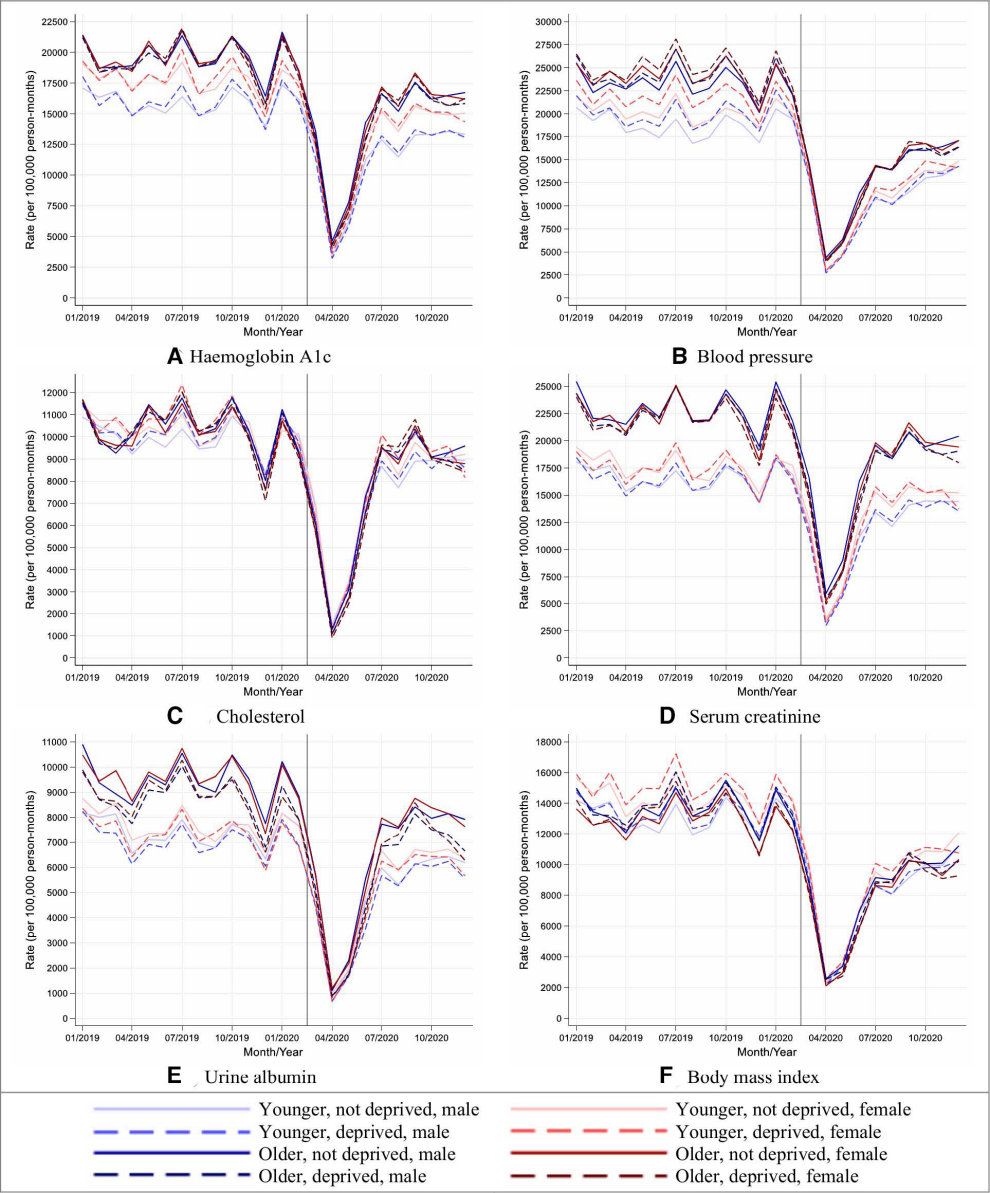
Stratified care process rates in people with type 2 diabetes during 2019 and 2020, in England. (A–F) Lines indicate observed monthly rates (years 2019 and 2020); the vertical line at 1 March 2020 separates the rates in primary care before and after the start of the COVID-19 pandemic, and x-axis markers indicate mid-months. Created by the authors.

Although reductions in rates of performing health checks were similar by age, sex and socioeconomic group, older people from deprived areas tended to have the greatest reductions in rates due to having higher background testing rates (England, figure 2; other UK nations, online supplemental figure 4).

Between May and December 2020, the reduced rates of performing health checks recovered gradually though rates remained well below expected especially for blood pressure and BMI monitoring (England, figure 1; other UK nations, online supplemental figure 3).

Overall in English practices, between 1 March and 10 December 2020, the rate of performing each of the health checks was reduced by between 28% and 47% compared with historical trends (table 1); the most affected health check being blood pressure testing (rate reduction (95% CI): 47% (45% to 49%)) and the least affected being urine albumin monitoring (reduction (95% CI): 28% (23% to 32%)). Similar trends were observed in other UK nations with rate reductions varying between 37% and 51% across the health checks (online supplemental table 2); blood pressure monitoring being most significantly reduced (rate reduction (95% CI): 51% (49% to 53%)).

**Table 1:** Comparison of observed and expected frequencies of diabetes-related care process implementation and new medication initiation in people with type 2 diabetes between March and December 2020 and in April 2020, in England; created by the authors

	Between March and December 2020			April 2020		
	Observed frequency	Expected frequency* (95% CI)	Percentage reduction (95% CI)	Observed frequency	Expected frequency (95% CI)	Percentage reduction (95% CI)
**Care processes**						
Haemoglobin A1c	573 172	833 676 (808 345 to 859 800)	31.2 (29.1 to 33.3)	19 365	85 075 (82 493 to 87 738)	77.2 (76.5 to 77.9)
Blood pressure	522 907	990 146 (956 661 to 1 024 803)	47.2 (45.3 to 49.0)	17 652	100 551 (97 156 to 104 065)	82.4 (81.8 to 83.0)
Cholesterol	321 728	462 829 (446 824 to 479 407)	30.5 (28.0 to 32.9)	5831	47 196 (45 565 to 48 885)	87.6 (87.2 to 88.1)
Serum creatinine	635 863	888 188 (857 708 to 919 751)	28.4 (25.9 to 30.9)	21 662	89 467 (86 399 to 92 645)	75.8 (74.9 to 76.6)
Urine albumin	244 389	338 493 (319 151 to 359 007)	27.8 (23.4 to 31.9)	4447	33 087 (31 197 to 35 092)	86.6 (85.7 to 87.3)
Body mass index	352 263	590 024 (567 077 to 613 899)	40.3 (37.9 to 42.6)	12 051	58 423 (56 149 to 60 789)	79.4 (78.5 to 80.2)
**New medication**						
Antidiabetic						
DPP-4i	2722	4148 (3847 to 4473)	34.4 (29.2 to 39.1)	190	455 (422 to 490)	58.2 (55.0 to 61.2)
GLP-1ag	525	712 (633 to 800)	26.3 (17.1 to 34.4)	25	78 (69 to 88)	67.9 (63.8 to 71.6)
Insulin	1467	1245 (1164 to 1331)	−17.8 (–26.0 to –10.2)	126	146 (136 to 156)	13.7 (7.4 to 19.2)
Metformin	15 055	18 883 (18 026 to 19 781)	20.3 (16.5 to 23.9)	845	2162 (2065 to 2265)	60.9 (59.1 to 62.7)
SGLT2i	2852	4183 (3656 to 4786)	31.8 (22.0 to 40.4)	154	440 (385 to 504)	65.0 (60.0 to 69.4)
Sulphonylurea	2582	2579 (2432 to 2735)	−0.1 (–6.2 to 5.6)	183	304 (287 to 322)	39.8 (36.2 to 43.2)
Any†	15 652	19 261 (18 407 to 20 155)	18.7 (15.0 to 22.3)	888	2219 (2120 to 2321)	60.0 (58.1 to 61.7)
Antihypertensive						
ACEi	3012	3883 (3648 to 4134)	22.4 (17.4 to 27.1)	153	437 (410 to 465)	65.0 (62.7 to 67.1)
α-blocker	1165	1563 (1474 to 1658)	25.5 (21.0 to 29.7)	101	171 (161 to 182)	40.9 (37.3 to 44.5)
ARB	1076	1534 (1433 to 1641)	29.9 (24.9 to 34.4)	74	177 (166 to 190)	58.2 (55.4 to 61.1)
β-blocker	1689	2009 (1903 to 2121)	15.9 (11.2 to 20.4)	157	224 (212 to 237)	29.9 (25.9 to 33.8)
Calcium channel blocker	2255	2994 (2825 to 3172)	24.7 (20.2 to 28.9)	192	333 (314 to 353)	42.3 (38.9 to 45.6)
Diuretic	1843	2101 (1982 to 2226)	12.3 (7.0 to 17.2)	147	238 (225 to 252)	38.2 (34.7 to 41.7)
Any‡	3430	4376 (4159 to 4604)	21.6 (17.5 to 25.5)	244	491 (467 to 517)	50.3 (47.8 to 52.8)
Lipid lowering						
Statin	8436	8041 (7436 to 8695)	−4.9 (–13.4 to 3.0)	434	868 (803 to 939)	50.0 (46.0 to 53.8)
Ezetimibe	224	193 (157 to 236)	−16.1 (–42.7 to 5.1)	24	21 (17 to 26)	−14.3 (–41.2 to 7.7)
Fibrate	97	90 (76 to 107)	−7.8 (–27.6 to 9.3)	<5	–	–
Any§	8428	8040 (7436 to 8693)	−4.8 (–13.3 to 3.0)	434	869 (803 to 939)	50.1 (46.0 to 53.8)
Antiplatelet						
Aspirin	1268	1163 (1084 to 1247)	−9.0 (–17.0 to –1.7)	133	133 (124 to 143)	0 (–7.3 to 7.0)
Clopidogrel	888	1158 (1038 to 1291)	23.3 (14.5 to 31.2)	82	120 (107 to 134)	31.7 (23.4 to 38.8)
Other¶	237	239 (193 to 295)	0.8 (–22.8 to 19.7)	17	26 (21 to 33)	34.6 (19.0 to 48.5)
Any	1592	1515 (1419 to 1617)	−5.1 (–12.2 to 1.5)	153	170 (159 to 181)	10.0 (3.8 to 15.5)

*The expected frequencies were estimated using negative binomial regression modelling on 10 years of historical data.

†Also includes α-glucosidase inhibitors, meglitinides and thiazolidinediones (glitazones).

‡Also includes central-acting agents, peripheral adrenergic inhibitors and vasodilators.

§Also includes cholestyramine, colesevelam, colestipol, niacin, lomitapide and PCSK9 inhibitors.

¶Includes cangrelor, dipyridamole, glycoprotein inhibitors, prasugrel and ticagrelor.

ACEi, angiotensin-converting enzyme inhibitor; ARB, angiotensin receptor blocker; DPP-4i, dipeptidyl peptidase-4 inhibitor; GLP-1ag, glucagon-like peptide 1 receptor agonist; PCSK9, proprotein convertase subtilisin/kexin type 9; SGLT2i, sodium/glucose cotransporter-2 inhibitor.

Similar patterns were observed in ethnicity-stratified analysis in England (online supplemental figure 8) and in other UK nations (online supplemental figure 9).

Extrapolated to the UK population, there were ~7.4 million fewer care processes undertaken between March and December 2020: ~2.3 million of these being blood pressure checks and ~1.2 million being HbA1c checks (online supplemental tables 3 and 4).

### Impacts of COVID-19 on diabetes-related prescribing

**Figure 3:**
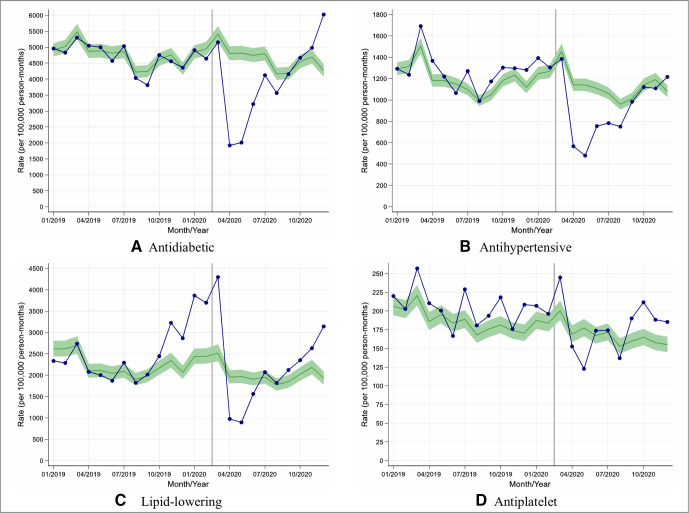
Observed and expected rates of new medication initiation in people with type 2 diabetes during 2019 and 2020, in England. (A–D) Blue lines indicate observed monthly rates (years 2019 and 2020), and green-shaded regions indicate expected rates with 95% CIs based on 10-year historical trends from January 2010; the vertical line at 1 March 2020 separates the rates in primary care before and after the start of the COVID-19 pandemic, and x-axis markers indicate mid-months. Created by the authors.

We assessed changes in rates of prescribing of new diabetes medication along with new antihypertensive, lipid-lowering and antiplatelet medications in people with T2D. In England, prescribing of new medication fell during April with rate reductions varying from 10% (95% CI: 4% to 16%) for antiplatelet agents to 60% (95% CI: 58% to 62%) for antidiabetic medications (figure 3). Similar patterns were observed in other UK nations with rate reductions varying between 26% (95% CI: 15% to 34%) for antiplatelet agents and 64% (95% CI: 61% to 66%) for lipid-lowering therapy (online supplemental figure 5).

In contrast to the data on rates of performing care processes, the largest reductions in rates of prescribing new diabetes medication and new lipid-lowering medication in England were seen in younger individuals from deprived and non-deprived backgrounds (online supplemental figure 6).

Overall in English practices, between 1 March and 10 December 2020, the overall rate of prescribing new diabetes medications was reduced by 19% (95% CI: 15% to 22%) when compared with historical trends (table 1); the most affected medication being dipeptidyl peptidase-4 inhibitors (reduction (95% CI): 34% (29% to 39%)) and the least affected being insulin which was initiated more frequently during this period compared with historical trends (increase (95% CI): 18% (10% to 26%)).

Similarly, the prescribing of new antihypertensive medication was reduced by 22% (95% CI: 18% to 25%) during this period whereas there was no significant change in the prescribing of new lipid-lowering or new antiplatelet therapy (table 1).

Between 1 March and 10 December 2020, similar reductions in the trends for prescribing of new medication were observed in other UK nations except that there was a significant reduction in new lipid-lowering therapy prescribing (reduction (95% CI): 15% (10% to 21%); online supplemental table 2).

Similar patterns of prescribing were observed in ethnicity-stratified analysis in England (online supplemental figure 10); data being too sparse to provide conclusive results in other UK nations.

Extrapolated to the UK population, between March and December 2020, there were ~31 800 fewer people with T2D prescribed a new type of diabetes medication including ~17 100 without a prior prescription of any first antidiabetic. Similarly, there were ~14 600 fewer people prescribed a new type of antihypertensive medication including ~4500 without a prior prescription of any first antihypertensive (online supplemental tables 3 and 4).

**Figure 4:**
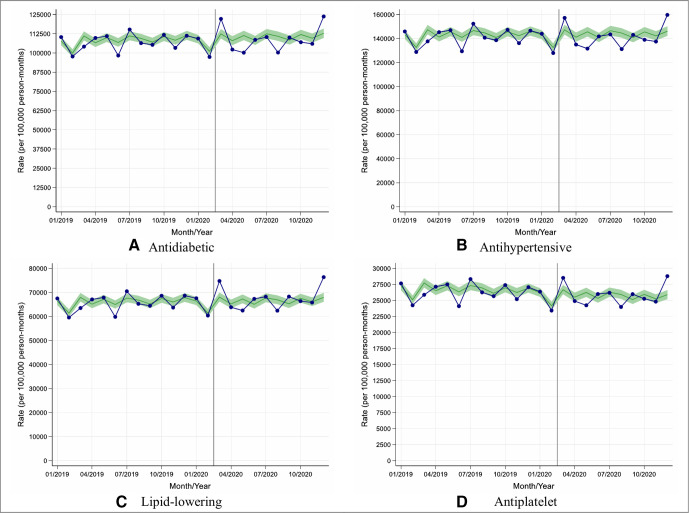
Observed and expected new and repeat medication prescribing rates in people with type 2 diabetes during 2019 and 2020, in England. (A–D) Blue lines indicate observed monthly rates (years 2019 and 2020), and green-shaded regions indicate expected rates with 95% CIs based on 10-year historical trends from January 2010; the vertical line at 1 March 2020 separates the rates in primary care before and after the start of the COVID-19 pandemic, and x-axis markers indicate mid-months. Created by the authors.

When considering both new prescribing and repeat prescribing combined, there was no significant change in the prescribing of antidiabetic, antihypertensive, lipid-lowering or antiplatelet therapies between March 2020 and December 2020 (England, figure 4; other UK nations, online supplemental figure 7).

## Discussion

We used primary care electronic health records from more than 600 000 people with T2D in the UK, and 10-year historical data, to show that during the 9 months following the first nationwide ‘lockdown’, the indirect consequences of the COVID-19 pandemic were associated with clinically significant changes in care quality and prescribing that could adversely impact patient safety. When compared with historical trends, we showed that: (1) there were 28%–47% reductions in rates of performing a range of health checks including a near halving of blood pressure testing rates; (2) older people with T2D and those from more deprived backgrounds experienced the greatest reduction in health checks; (3) overall rates of prescribing new diabetes and antihypertensive medication were reduced by 19%–22%; and (4) reassuringly, when considering rates of new and repeat medication prescribing combined, there were no significant differences.

To provide some context for our health check data, National Diabetes Audit data indicate that during the 15 months prior to the national lockdown in March 2020, most people with T2D in England had the six health checks performed: haemoglobin A1c (94% of people), serum creatinine (92%), cholesterol (91%), urinary albumin excretion (69%), blood pressure (95%) and BMI (88%).^7^

There are limited data on the impact of the COVID-19 pandemic on rates of performing health checks and prescribing in people with T2D diabetes. An earlier UK-wide study using primary care data from people with T2D showed a 31% reduction in HbA1c testing, a 20% reduction in new metformin prescribing and 5% reduction in new insulin prescribing between March and December 2020 compared with historical trends.^13^ Here we extend these observations by assessing the impact of the COVID-19 pandemic on a much wider range of health checks and a wider range of diabetes-related medication including agents that reduce cardiovascular risk.

Our findings have important clinical implications for diabetes care quality and safety. In early March 2020, GPs were advised to minimise the number of face-to-face contacts they had with their patients.^19–22^ Our data suggest that this reduction of clinical services has contributed to major reductions in the monitoring of T2D and the prescribing of new medication, particularly for hypertension. T2D is a progressive condition and therefore without intervention, levels of glucose and associated cardiovascular disease (CVD) risk factors such as blood pressure tend to increase over time. It is concerning that we observed a 22% reduction in new antihypertensive medication prescribing between March and December 2020, perhaps caused by less frequent monitoring and restricted face-to-face clinical contacts. Anecdotally, we understand that some practices were reluctant to prescribe ACE inhibitors as they would not be able to routinely check renal bloods after initiation. There are already concerns about clinical inertia in diabetes management in the UK,^23^ and therefore any further reduction in monitoring and related prescribing could increase the risk of mortality and long-term complications.

Our data indicate that reductions in prescribing relate to new prescriptions but not repeat prescriptions. Robust systems for repeat prescribing in the UK appear to have helped minimise the harm done through reductions in face-to-face consultations during the pandemic.

The effect of COVID-19 lockdown restrictions on metabolic control in people with diabetes appears heterogeneous. Some studies in insulin-treated people have suggested improvements in glucose control perhaps brought about through more regular mealtimes, increased consumption of homemade foods, decreased workloads, less exercise and more time for self-care.^24–26^

However, in many people with T2D, there is evidence that national lockdowns have had detrimental effects on CVD risk, which could be exacerbated by the reduced monitoring and prescribing observed in our study. In surveys of UK adults conducted during the first lockdown (April-–May 2020), participants reported adverse changes in several behaviours that promote weight gain (adverse changes in diet, physical activity, alcohol consumption, mental health and sleep quality).^27 28^ Some^29–31^ but not all studies^32 33^ in people with T2D have shown worsening of glycaemic control in relation to COVID-19 lockdown which could explain the increased rates of insulin prescribing we observed between March and December 2020.

As engagement with health services increases over the coming months, we predict marked increases in the need for monitoring and prescribing of new medication in people with T2D. Healthcare services will need to manage this backlog of testing and prescribing, and the anticipated greater deterioration of HbA1c and other CVD risk factors such as blood pressure levels. Older people from deprived backgrounds appear to be most adversely affected by reduced monitoring and therefore these individuals may be particular groups to target for early intervention. During this pandemic and its associated lockdowns, effective communications should ensure that patients remain engaged with diabetes services, monitoring and opportunities for prescribing,^34^ and make use of home monitoring of blood pressure, weight and HbA1c (when available), and remote consultations.^35–37^ Investment in such technology and devices would be expected to yield important health benefits.

The COVID-19 pandemic provides us with a unique opportunity to improve the current care model by providing greater investment in patient education, devices and technology and greater use of remote consultations to deliver the high standards of care that people with diabetes should expect to receive.

Although our data relate directly to people with T2D, they have relevance to other medical conditions. We,^38 39^ and others,^40^ have shown substantial reductions in primary care contacts for a wide range of physical and mental health conditions following COVID-19 restrictions. Maintaining healthcare access for all should be a key priority in public health planning.

Our study had several strengths: this is the first UK-wide study reporting the indirect impact of the COVID-19 pandemic on health checks and prescribing in people with T2D. Our findings in English practices were replicated in other parts of the UK and are likely to be representative of the UK in general. Our study has some limitations: first, we did not report data on retinopathy, smoking and foot checks (the remaining three of the nine health checks recommended by NICE). Retinopathy screening is performed in the community and therefore these data are not available in primary care records. While assessments of smoking status and foot checks are performed in primary care, we were less confident in defining whether or not these checks had been performed based on the available data. Second, we do not present data on type 1 diabetes as the majority of care for these individuals is delivered in secondary care centres. Third, ethnicity coding is not adequately captured in primary care and therefore we had limited ability to explore ethnicity-related variation in outcomes. Fourth, we did not assess risk factor levels because our focus was on processes of care and prescribing. Fifth, our data would not capture assessments of weight and blood pressure assessed by patients in their homes. Results of home blood pressure recordings may have had an influence on prescribing between March and December 2020 because the reduction in prescribing of new antihypertensive agents (~13%) was less than the reduction in blood pressure monitoring performed in primary care (~51%). Finally, although our results and conclusions are relevant to the UK population, generalisability to other healthcare systems may be limited. However, a pan-European survey of diabetes specialist nurses reported that the level of care provided for people with diabetes had been significantly disrupted during the pandemic.^34^

In conclusion, we highlight marked reductions in the rate of health checks and new prescribing in people with T2D as indirect consequences of the COVID-19 pandemic. Over the coming months, healthcare services will need to manage this backlog of testing and prescribing, and the anticipated greater deterioration of HbA1c and other CVD risk factors such as blood pressure levels. Older people from deprived backgrounds with T2D may be specific groups to target for early health checks and intervention.

During the next year, effective communications should ensure that patients remain engaged with diabetes services including opportunities for prescribing and that they make use of home monitoring of blood pressure, weight, foot health and blood glucose when appropriate. Telemedicine, digital services and remote consultations may provide opportunities to engage some groups of people living with diabetes who have been difficult to reach. Healthcare planners should seize opportunities provided by the COVID-19 pandemic to improve models, processes and standards of care for people with diabetes. A positive lasting legacy of COVID-19 might be accelerated innovation in diabetes care and other chronic diseases.^35^

## Data Availability

Data may be obtained from a third party and are not publicly available. Electronic health records are, by definition, considered “sensitive” data in the UK by the Data Protection Act and cannot be shared via public deposition because of information governance restriction in place to protect patient confidentiality. Access to data are available only once approval has been obtained through the individual constituent entities controlling access to the data. The primary care data can be requested via application to the Clinical Practice Research Datalink (www.cprd.com).
