# Supplementary material for: Impact of COVID-19 restrictions on diabetes health checks and prescribing for people with type 2 diabetes: a UK-wide cohort study involving 618 161 people in primary care
Source: BMJ Qual Saf. 2021 Oct 12;31(7):503–14. doi: 10.1136/bmjqs-2021-013613 (PMC8520602; doi:10.1136/bmjqs-2021-013613)
Supplement: Supplementary data [file bmjqs-2021-013613supp001.pdf]

Supplemental Material

Table of Contents

Tables

Supplementary Table 1. Strata applied to study cohorts 3

Supplementary Table 2. Comparison of observed and expected rates of diabetes-related care process implementation and new medication initiation in people with type 2 diabetes between March and December 2020 and in April 2020, in Northern Ireland, Scotland, and Wales 4

Supplementary Table 3. Estimating the proportion of the UK population covered by the CPRD databases 5

Supplementary Table 4. Extrapolating the estimated shortfall in care processes undertaken and new medications prescribed between March and December 2020 from the CPRD sample to the UK population 6

Figures

Supplementary Figure 1. Flow diagram of the cohort from CPRD Aurum 7

Supplementary Figure 2. Flow diagram of the cohort from CPRD GOLD 8

Supplementary Figure 3. Observed and expected care process rates in people with type 2 diabetes during 2019 and 2020, in Northern Ireland, Scotland and Wales 9

Supplementary Figure 4. Stratified care process rates in people with type 2 diabetes during 2019 and 2020, in Northern Ireland, Scotland and Wales 10

Supplementary Figure 5. Observed and expected rates of new medication initiation in people with type 2 diabetes during 2019 and 2020, in Northern Ireland, Scotland and Wales 11

Supplementary Figure 6. Stratified rates of new medication initiation in people with type 2 diabetes during 2019 and 2020, in England 12

Supplementary Figure 7. Observed and expected new and repeat medication prescribing rates in people with type 2 diabetes during 2019 and 2020, in Northern Ireland, Scotland and Wales 13

Supplementary Figure 8. Observed ethnicity-stratified care process rates in people with type 2 diabetes during 2019 and 2020, in England 14

Supplementary Figure 9. Observed ethnicity-stratified care process rates in people with type 2 diabetes during 2019 and 2020, in Northern Ireland, Scotland, and Wales 15

Supplementary Figure 10. Observed ethnicity-stratified rates of new medication initiation in people with type 2 diabetes during 2019 and 2020, in England 16

APPENDICES: Clinical and Drug Prescription Codes

Part A1. SNOMED CT codes used to interrogate CPRD *Aurum*

Appendix 1. SNOMED CT codes for diagnoses of type 2 diabetes 17

Appendix 2. SNOMED CT codes for haemoglobin A1c monitoring 19

Appendix 3. SNOMED CT codes for blood pressure monitoring 21

Carr MJ, *et al. BMJ Qual Saf* 2022; 31:503–514. doi: 10.1136/bmjqs-2021-013613

Appendix 4. SNOMED CT codes for cholesterol monitoring 23

Appendix 5. SNOMED CT codes for serum creatinine monitoring 25

Appendix 6. SNOMED CT codes for urine albumin monitoring 28

Appendix 7. SNOMED CT codes for recording body mass index 31

Part A2. Drug prescription codes used to interrogate CPRD *Aurum*

Appendix 8. Product codes for prescriptions of antidiabetic medications 33

Appendix 9. Product codes for prescriptions of antihypertensive medications 47

Appendix 10. Product codes for prescriptions of lipid-lowering medications 80

Appendix 11. Product codes for prescriptions of antiplatelet medications 84

Part B1. Read codes used to interrogate CPRD *GOLD*

Appendix 12. Read codes for diagnoses of type 2 diabetes 87

Appendix 13. Read codes for haemoglobin A1c monitoring 90

Appendix 14. Read codes for blood pressure monitoring 91

Appendix 15. Read codes for cholesterol monitoring 93

Appendix 16. Read codes for serum creatinine monitoring 95

Appendix 17. Read codes for urine albumin monitoring 97

Appendix 18. Read codes for recording body mass index 99

Part B2. Drug prescription codes used to interrogate CPRD *GOLD*

Appendix 19. Product codes for prescriptions of antidiabetic medications 100

Appendix 20. Product codes for prescriptions of antihypertensive medications 121

Appendix 21. Product codes for prescriptions of lipid-lowering medications 201

Appendix 22. Product codes for prescriptions of antiplatelet medications 212

Supplementary Table 1. Strata applied to study cohorts

Stratum	Label	Older	Deprived	Female
1	Younger, not deprived, male	0	0	0
2	Younger, not deprived, female	0	0	1
3	Younger, deprived male	0	1	0
4	Younger, deprived female	0	1	1
5	Older, not deprived, male	1	0	0
6	Older, not deprived, female	1	0	1
7	Older, deprived male	1	1	0
8	Older, deprived female	1	1	1

- Older = 0,1 if age ≥ 65
- Deprived = 0,1 if IMD quintile = 4 or 5
- Female = 0,1

Carr MJ, *et al. BMJ Qual Saf* 2022; 31:503–514. doi: 10.1136/bmjqs-2021-013613

Supplementary Table 2. Comparison of observed and expected frequencies of diabetes-related care process implementation and new medication initiation in people with type 2 diabetes between March and December 2020 and in April 2020, in Northern Ireland, Scotland, and Wales

	Between March and December 2020			April 2020		
	Observed Frequency	Expected Frequency (95% CI)	Percentage Reduction (95% CI)	Observed Frequency	Expected Frequency (95% CI)	Percentage Reduction (95% CI)
**Care processes**						
Haemoglobin A1c	72,084	113,705 (110,149 to 117,376)	36.6 (34.6 to 38.6)	1862	11,513 (11,153 to 11,885)	83.8 (83.3 to 84.3)
Blood pressure	72,650	147,623 (142,287 to 153,159)	50.8 (48.9 to 52.6)	2050	14,967 (14,427 to 15,526)	86.3 (85.8 to 86.8)
Cholesterol	48,674	77,035 (74,341 to 79,826)	36.8 (34.5 to 39.0)	944	7891 (7615 to 8177)	88.0 (87.6 to 88.5)
Serum creatinine	112,867	195,299 (184,827 to 206,364)	42.2 (38.9 to 45.3)	5260	20,049 (18,976 to 21,182)	73.8 (72.3 to 75.2)
Urine albumin	36,881	63,500 (60,730 to 66,396)	41.9 (39.3 to 44.5)	774	6393 (6115 to 6684)	87.9 (87.3 to 88.4)
Body mass index	48,242	96,139 (92,499 to 99,922)	49.8 (47.8 to 51.7)	1151	9659 (9293 to 10,038)	88.1 (87.6 to 88.5)
**New medication**						
Antidiabetic						
DPP-4i	414	619 (566 to 677)	33.1 (26.9 to 38.8)	27	68 (62 to 74)	60.3 (56.5 to 63.5)
GLP-1ag	85	82 (67 to 102)	-3.7 (-26.9 to 16.7)	<5	-	-
Insulin	234	224 (195 to 258)	-4.5 (-20.0 to 9.3)	21	23 (20 to 27)	8.7 (-5.0 to 22.2)
Metformin	2874	3310 (3121 to 3510)	13.2 (7.9 to 18.1)	138	386 (364 to 409)	64.2 (62.1 to 66.3)
SGLT2i	616	929 (806 to 1070)	33.7 (23.6 to 42.4)	26	92 (79 to 107)	71.7 (67.1 to 75.7)
Sulphonylurea	599	625 (578 to 675)	4.2 (-3.6 to 11.3)	41	75 (69 to 81)	45.3 (40.6 to 49.4)
Any [1]	2985	3417 (3224 to 3622)	12.6 (7.4 to 17.6)	147	403 (381 to 427)	63.5 (61.4 to 65.6)
Antihypertensive						
ACEi	526	685 (636 to 737)	23.2 (17.3 to 28.6)	23	77 (71 to 83)	70.1 (67.6 to 72.3)
α-blocker	214	249 (220 to 282)	14.1 (2.7 to 24.1)	11	26 (22 to 29)	57.7 (50.0 to 62.1)
ARB	180	223 (194 to 256)	19.3 (7.2 to 29.7)	9	24 (21 to 28)	62.5 (57.1 to 67.9)
β-blocker	328	384 (342 to 432)	14.6 (4.1 to 24.1)	21	39 (34 to 44)	46.2 (38.2 to 52.3)
CC-blocker	363	472 (430 to 518)	23.1 (15.6 to 29.9)	22	56 (51 to 61)	60.7 (56.9 to 63.9)
Diuretic	375	384 (344 to 428)	2.3 (-9.0 to 12.4)	20	42 (37 to 46)	52.4 (45.9 to 56.5)
Any [2]	616	738 (684 to 795)	16.5 (9.9 to 22.5)	40	81 (75 to 87)	50.6 (46.7 to 54.0)
Lipid-lowering						
Statin	1196	1420 (1325 to 1522)	15.8 (9.7 to 21.4)	55	153 (143 to 165)	64.1 (61.5 to 66.7)
Ezetimibe	33	18 (11 to 29)	-83.3 (-200.0 to -13.8)	<5	-	-
Fibrate	25	16 (11 to 23)	-56.3 (-127.3 to -8.7)	<5	-	-
Any [3]	1199	1419 (1325 to 1520)	15.5 (9.5 to 21.1)	56	153 (143 to 164)	63.4 (60.8 to 65.9)
Antiplatelet						
Aspirin	239	229 (202 to 260)	-4.4 (-18.3 to 8.1)	16	24 (21 to 27)	33.3 (23.8 to 40.7)
Clopidogrel	196	228 (192 to 271)	14.0 (-2.1 to 27.7)	14	23 (19 to 27)	39.1 (26.3 to 48.1)
Other [4]	61	61 (42 to 88)	0 (-45.2 to 30.7)	5	5 (4 to 8)	0 (-25.0 to 37.5)
Any	300	298 (266 to 336)	-0.7 (-12.8 to 10.7)	23	31 (27 to 35)	25.8 (14.8 to 34.3)

╪ The expected frequencies were estimated using negative binomial regression modelling on 10-years of historical data

1. Also includes α-glucosidase inhibitors, meglitinides, and thiazolidinediones (glitazones).
2. Also includes central-acting agents, peripheral adrenergic inhibitors, and vasodilators.
3. Also includes cholestyramine, colesevelam, colestipol, niacin, lomitapide, and PCSK9 inhibitors.
4. Includes cangrelor, dipyridamole, glycoprotein inhibitors, prasugrel, and ticagrelor.

Carr MJ, *et al. BMJ Qual Saf* 2022; 31:503–514. doi: 10.1136/bmjqs-2021-013613

Supplementary Table 3. Estimating the proportion of the UK population covered by the CPRD databases

	Population ǂ		Sample	
	**All ages**	**Aged ≥5**	**Aged ≥5**	**Coverage (%)**
**United Kingdom**	66,796,807	62,939,544	14,863,091	23.61
**England**	56,286,961	52,987,324	12,550,611	23.69
**NI, Scotland, Wales**	10,509,846	9,952,220	2,312,480	23.24

ǂ Source: https://www.ons.gov.uk/peoplepopulationandcommunity/populationandmigration/populationestimates

- Footnote: The population estimates were obtained from mid-year data for 2019 available from the ONS. To determine corresponding sample sizes, we placed the same restrictions on the eligible pool of patients (25,837,067 from CPRD *Aurum* and 4,263,728 from CPRD *GOLD*) that we applied when delineating the cohort of patients with type 2 diabetes: see Supplementary Figures 1 and 2. We limited inclusion to patients who a) contributed at least one year of follow-up during the study period; b) did not exit the study prior to their 5th birthday and c) did not exit the study population prior to 1st March 2020.

Supplementary Table 4. Extrapolating the estimated shortfall in care processes undertaken and new medications prescribed between March and December 2020 from the CPRD sample to the UK population

	England		NI, Scotland, Wales		UK combined	
	Sample	Population ǂ	Sample	Population ǂ	Sample	Population
**Care processes**						
Haemoglobin A1c	260,504	1,099,820	41,621	179,124	302,125	1,278,944
Blood pressure	467,239	1,972,633	74,973	322,661	542,212	2,295,294
Cholesterol	141,101	595,713	28,361	122,057	169,462	717,770
Serum creatinine	252,325	1,065,289	82,432	354,763	334,757	1,420,052
Urine albumin	94,104	397,297	26,619	114,560	120,723	511,857
Body mass index	237,761	1,003,801	47,897	206,134	285,658	1,209,935
**New medication**						
Antidiabetic:						
DPP-4i	1426	6020	205	882	1631	6902
GLP-1ag	187	789	-3	-13	184	776
Insulin	-222	-937	-10	-43	-232	-980
Metformin	3828	16,161	436	1876	4264	18,037
SGLT2i	1331	5619	313	1347	1644	6966
Sulphonylurea	-3	-13	26	112	23	99
Any [1]	3609	15,237	432	1859	4041	17,096
Antihypertensive:						
ACEi	871	3677	159	684	1030	4361
α-blocker	398	1680	35	151	433	1831
ARB	458	1934	43	185	501	2119
β-blocker	320	1351	56	241	376	1592
CC-blocker	739	3120	109	469	848	3589
Diuretic	258	1089	9	39	267	1128
Any [2]	946	3994	122	525	1068	4519
Lipid-lowering:						
Statin	-395	-1668	224	964	-171	-704
Ezetimibe	-31	-131	-15	-65	-46	-196
Fibrate	-7	-30	-9	-39	-16	-69
Any [3]	-388	-1638	220	947	-168	-691
Antiplatelet:						
Aspirin	-105	-443	-10	-43	-115	-486
Clopidogrel	270	1140	32	138	302	1278
Other [4]	2	8	0	0	2	8
Any	-77	-325	-2	-9	-79	-334

ǂ The expected frequencies were estimated using negative binomial regression modelling on 10-years of historical data. Extrapolations of the shortfall in care processes undertaken and new medications prescribed were derived using the estimated coverage of each CRPD database (Supplementary Table 3).

1. Also includes α-glucosidase inhibitors, meglitinides, and thiazolidinediones (glitazones).
2. Also includes central-acting agents, peripheral adrenergic inhibitors, and vasodilators.
3. Also includes cholestyramine, colesevelam, colestipol, niacin, lomitapide, and PCSK9 inhibitors.
4. Includes cangrelor, dipyridamole, glycoprotein inhibitors, prasugrel, and ticagrelor.

Carr MJ, *et al. BMJ Qual Saf* 2022; 31:503–514. doi: 10.1136/bmjqs-2021-013613

Supplementary Figure 1. Flow diagram of the cohort from CPRD *Aurum*

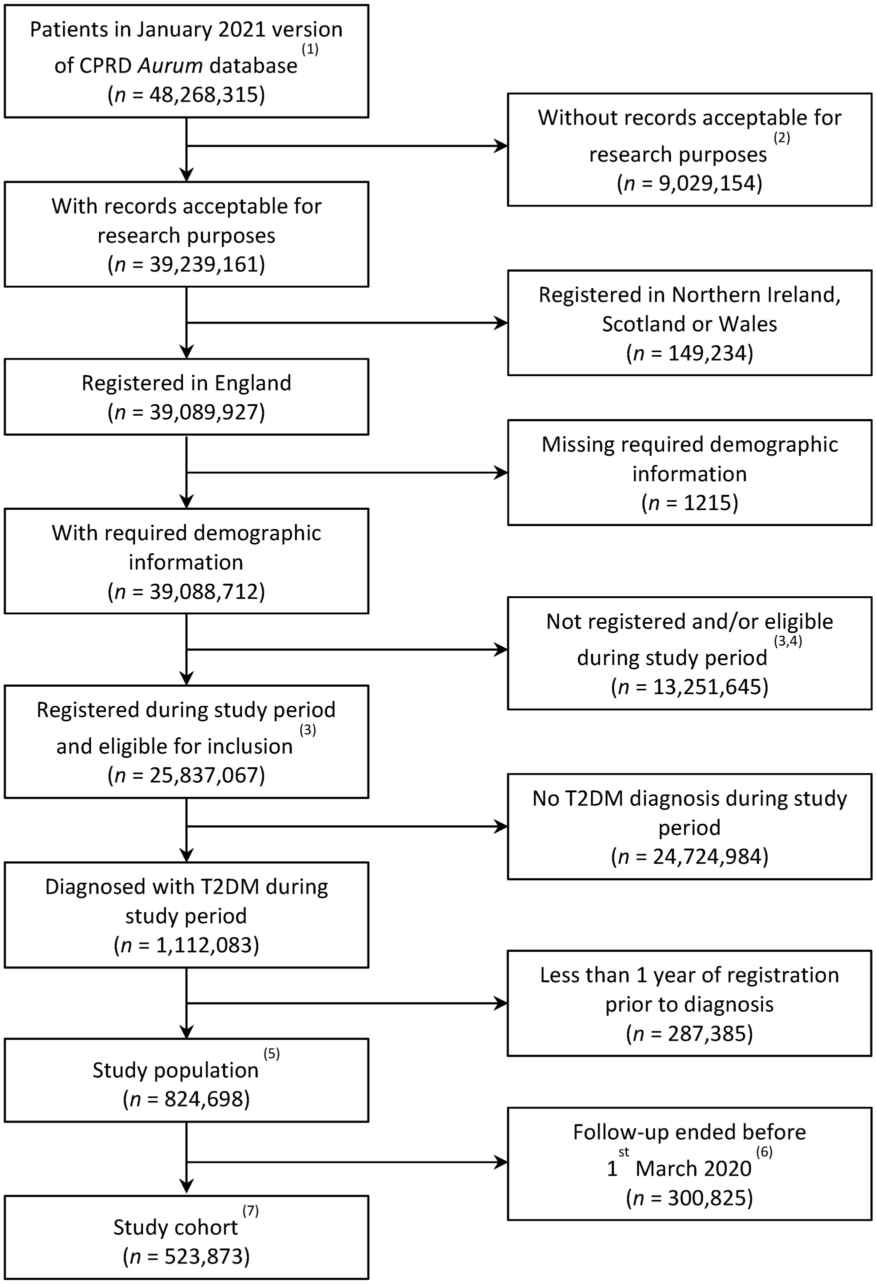

1. Permanent registrations only.
2. Acceptability defined by CPRD as meeting certain quality standards.
3. Study period: 1st January 2010 to 10th December 2020.
4. Reasons: (a) registration ended prior to study period due to death or migration from practice (*n* = 13,242,965); (b) registration end date defined on or prior to registration start date (*n* = 8,680).
5. Study population used to model trends (between January 2010 and February 2020) and/or compare expected and observed rates (between March and December 2020).
6. Only contributed to modelling trends.
7. At least one day of follow-up during 1st March to 10th December 2020.

Supplementary Figure 2. Flow diagram of the cohort from CPRD *GOLD*

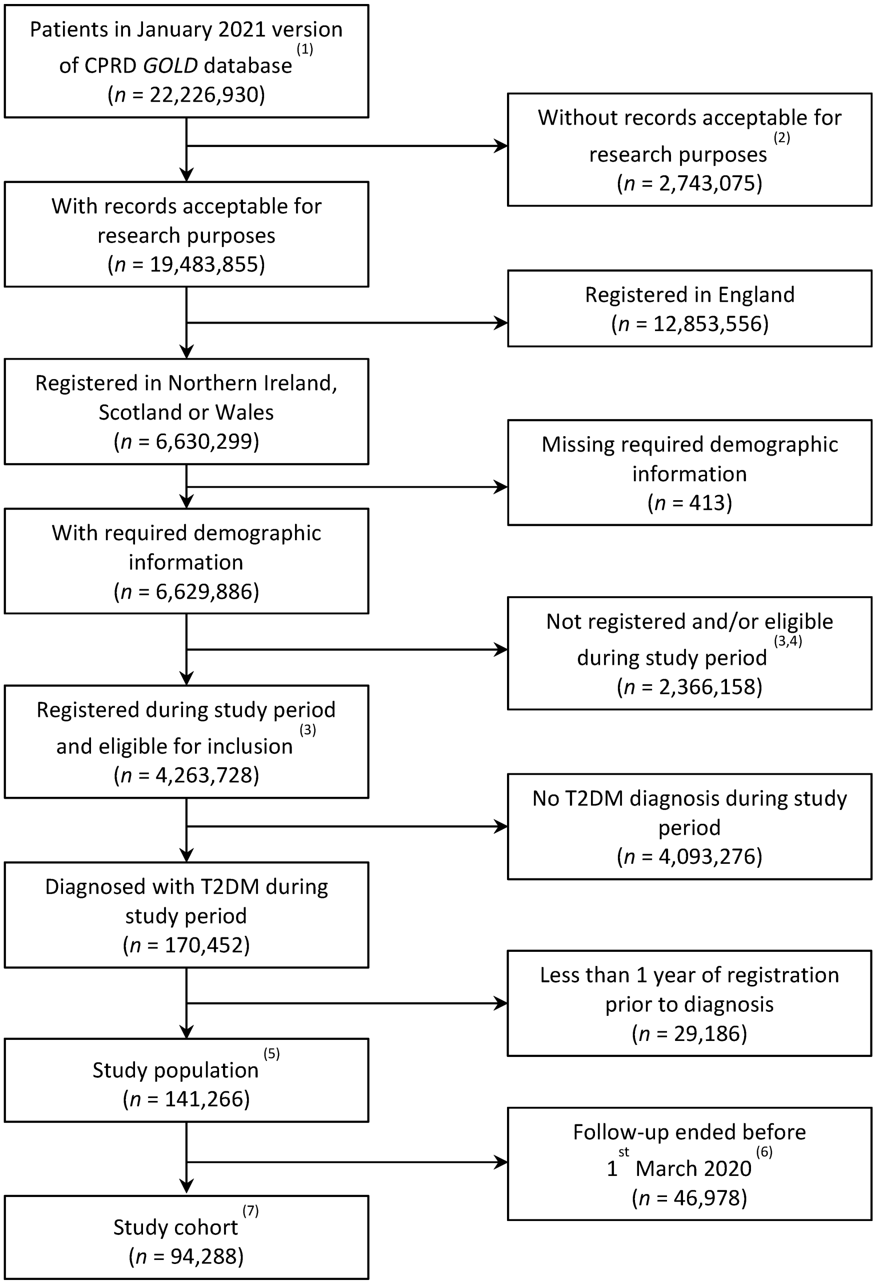

1. Permanent registrations only.
2. Acceptability defined by CPRD as meeting certain quality standards.
3. Study period: 1st January 2010 to 10th December 2020.
4. Reasons: (a) registration ended prior to study period due to death or migration from practice (*n* = 2,300,863); (b) registration end date defined on or prior to registration start date (*n* = 65,295).
5. Study population used to model trends (between January 2010 and February 2020) and/or compare expected and observed rates (between March and December 2020).
6. Only contributed to modelling trends.
7. At least one day of follow-up during 1st March to 10th December 2020.

Supplementary Figure 3. Observed and expected care process rates in people with type 2 diabetes during 2019 and 2020, in Northern Ireland, Scotland and Wales

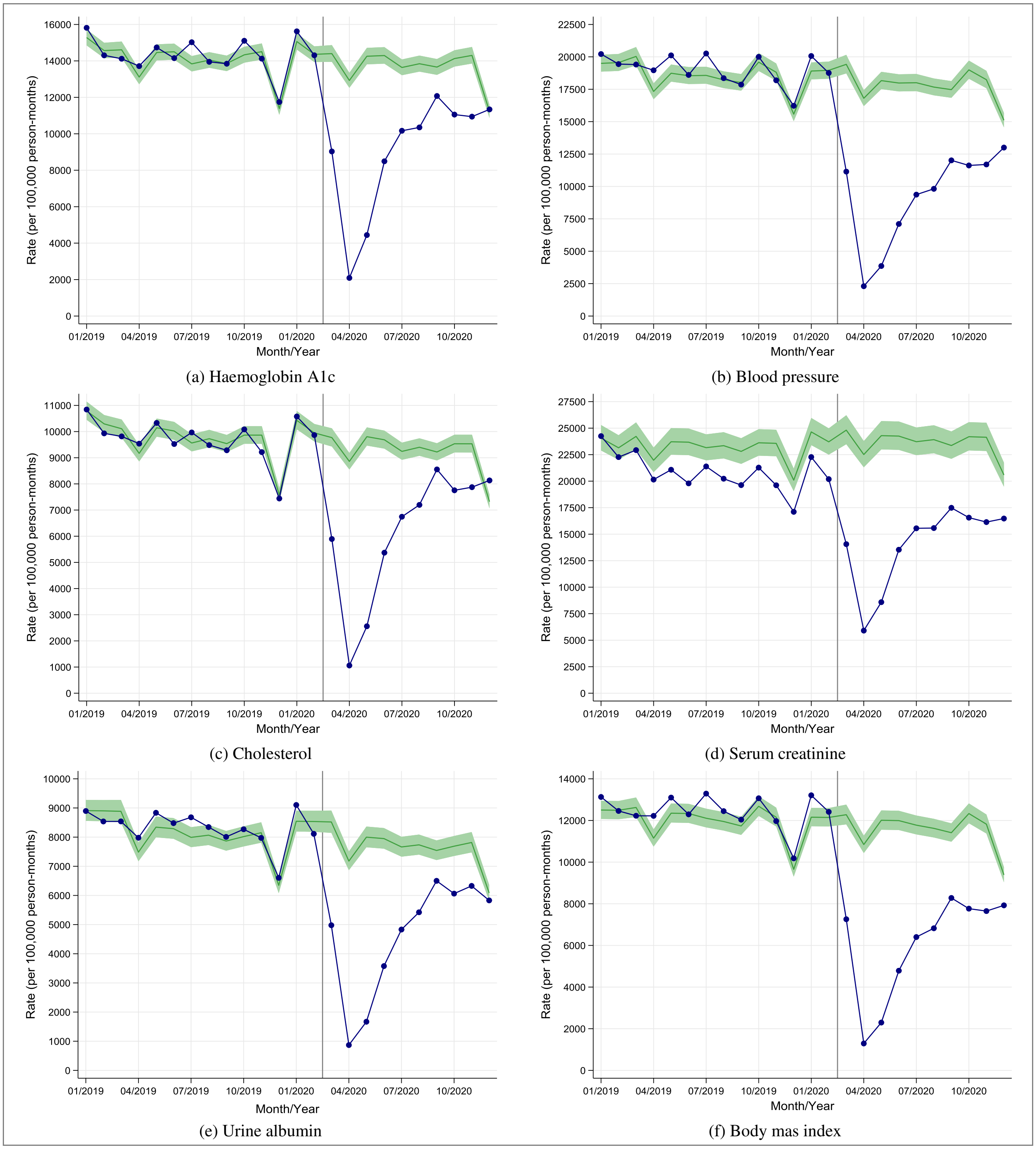

Carr MJ, *et al. BMJ Qual Saf* 2022; 31:503–514. doi: 10.1136/bmjqs-2021-013613

Supplementary Figure 4. Stratified care process rates in people with type 2 diabetes during 2019 and 2020, in Northern Ireland, Scotland and Wales

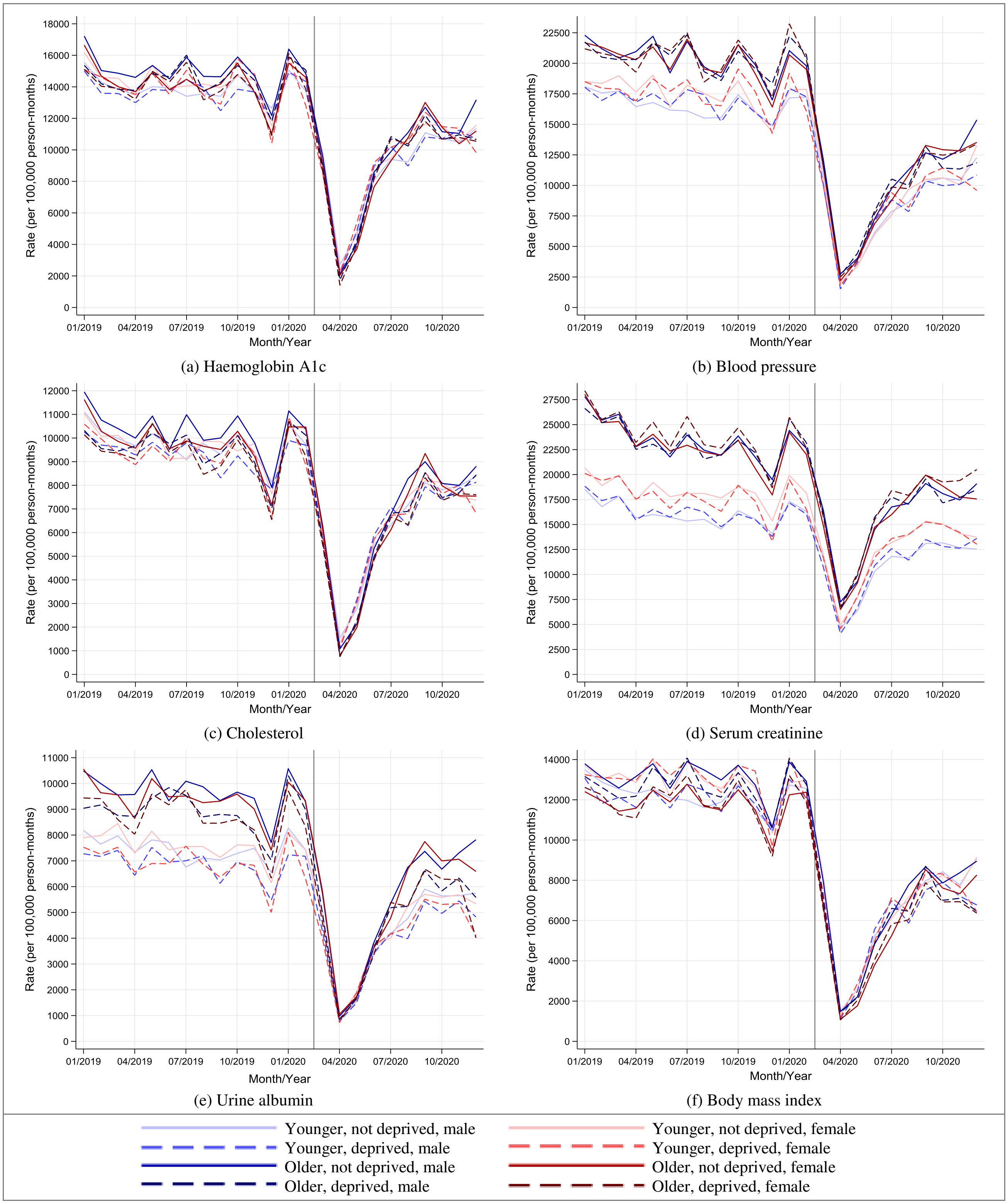

(a) Haemoglobin A1c

(b) Blood pressure

(c) Cholesterol

(d) Serum creatinine

(e) Urine albumin

(f) Body mass index

Carr MJ, *et al. BMJ Qual Saf* 2022; 31:503–514. doi: 10.1136/bmjqs-2021-013613

Supplementary Figure 5. Observed and expected rates of new medication initiation in people with type 2 diabetes during 2019 and 2020, in Northern Ireland, Scotland and Wales

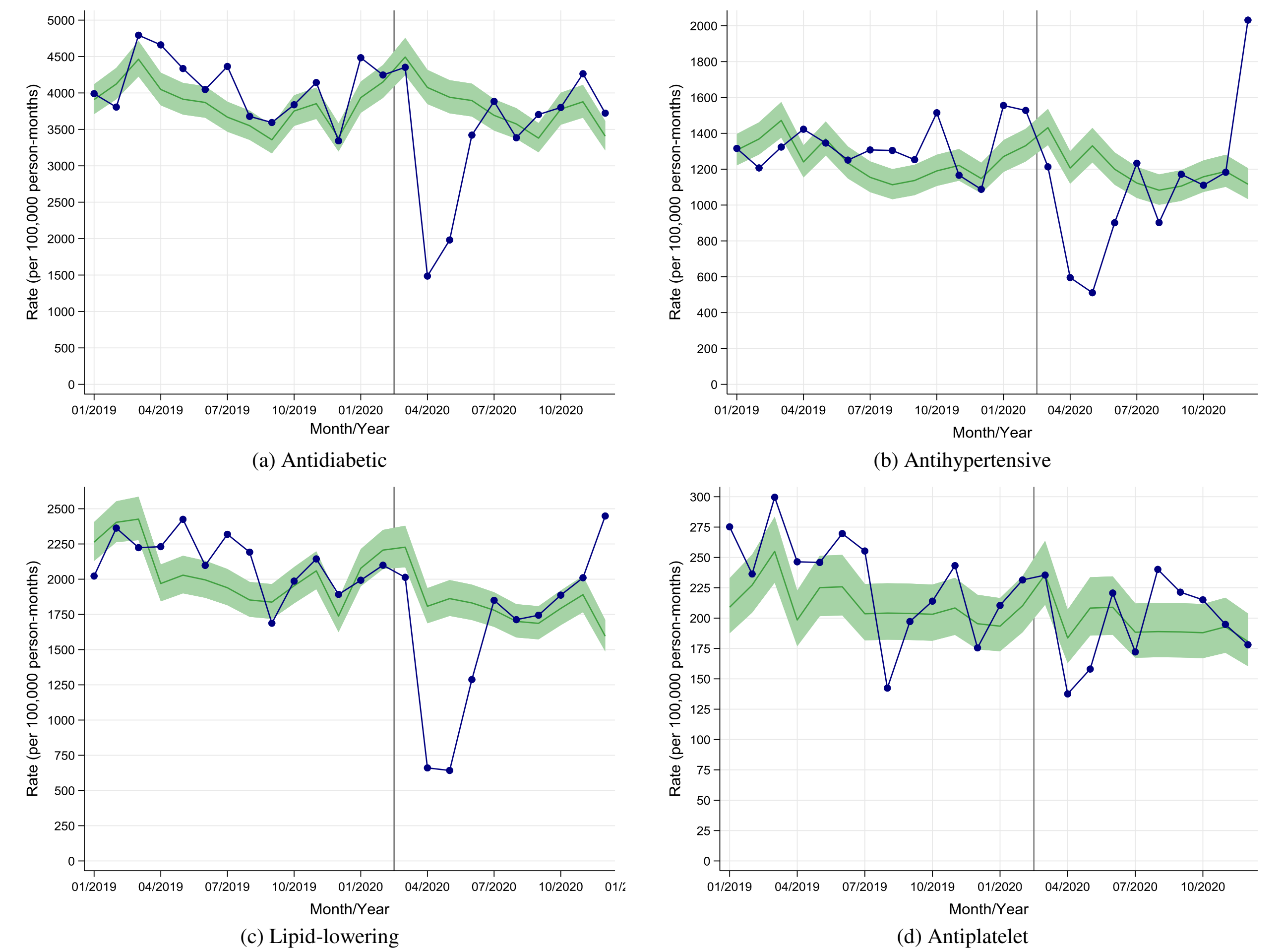

(a) Antidiabetic

(b) Antihypertensive

(c) Lipid-lowering

(d) Antiplatelet

Carr MJ, *et al. BMJ Qual Saf* 2022; 31:503–514. doi: 10.1136/bmjqs-2021-013613

Supplementary Figure 6. Stratified rates of new medication initiation in people with type 2 diabetes during 2019 and 2020, in England

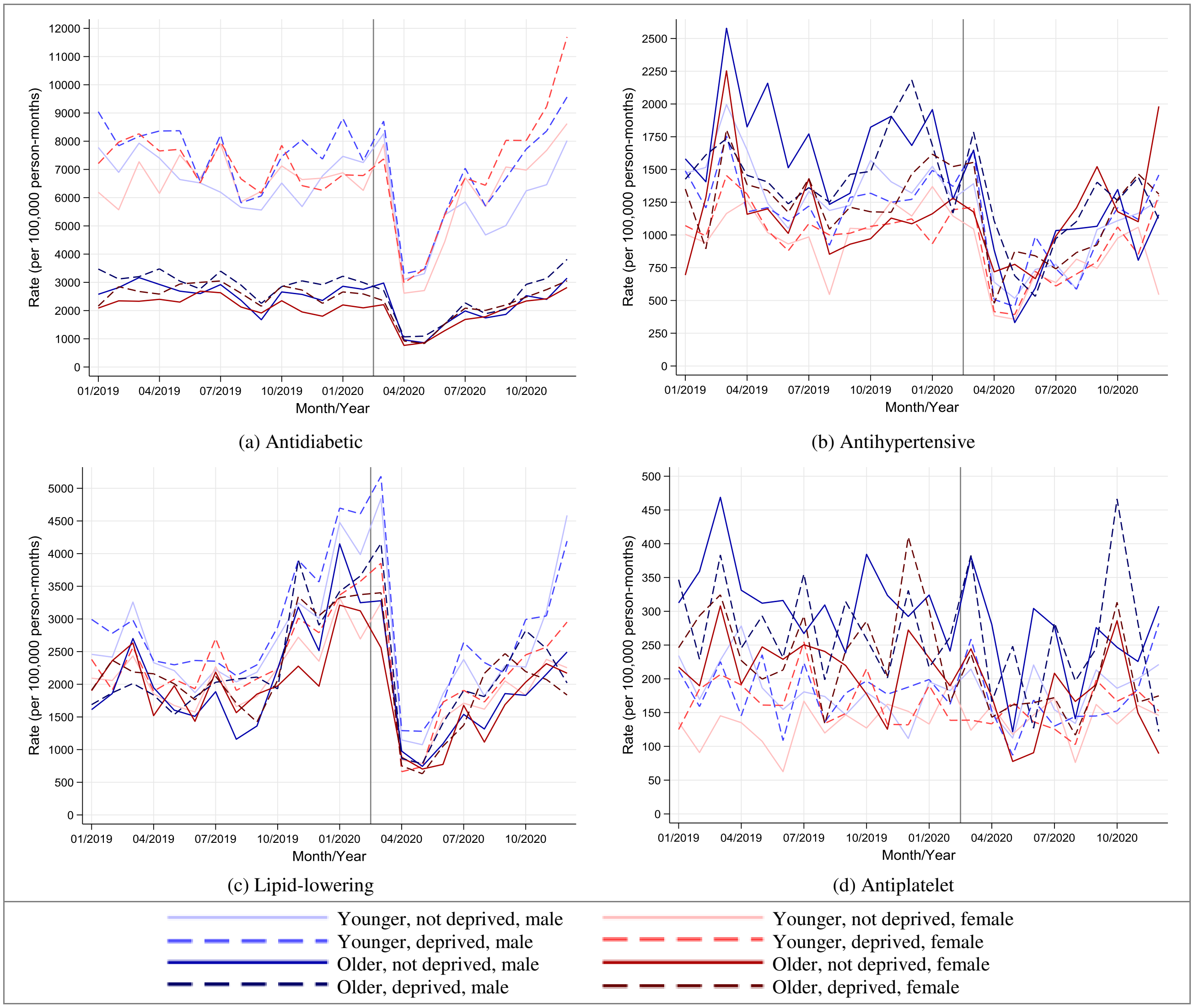

Supplementary Figure 7. Observed and expected new and repeat medication prescribing rates in people with type 2 diabetes during 2019 and 2020, in Northern Ireland, Scotland and Wales

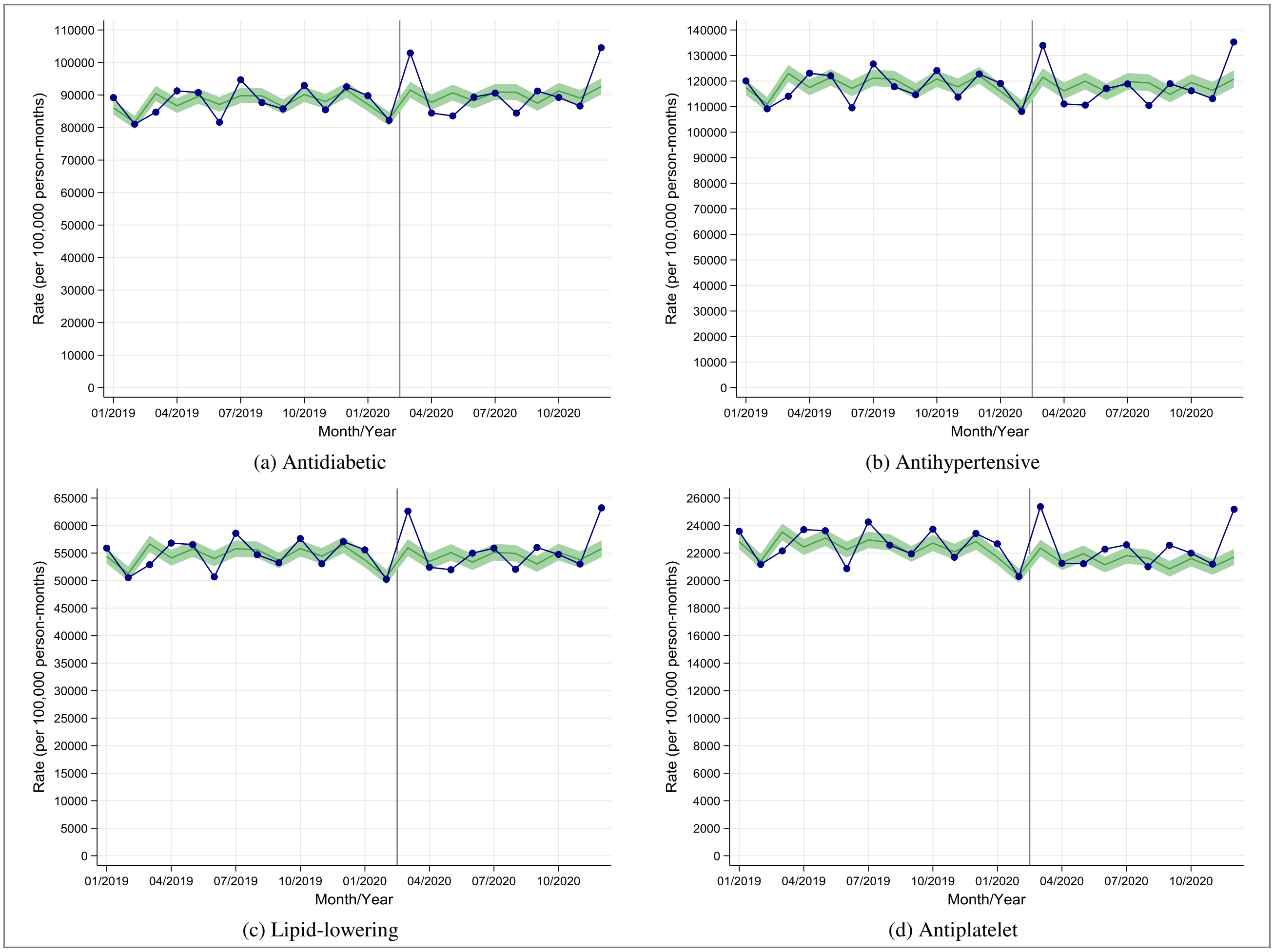

(a) Antidiabetic

(b) Antihypertensive

(c) Lipid-lowering

(d) Antiplatelet

Supplementary Figure 8. Observed ethnicity-stratified care process rates in people with type 2 diabetes during 2019 and 2020, in England

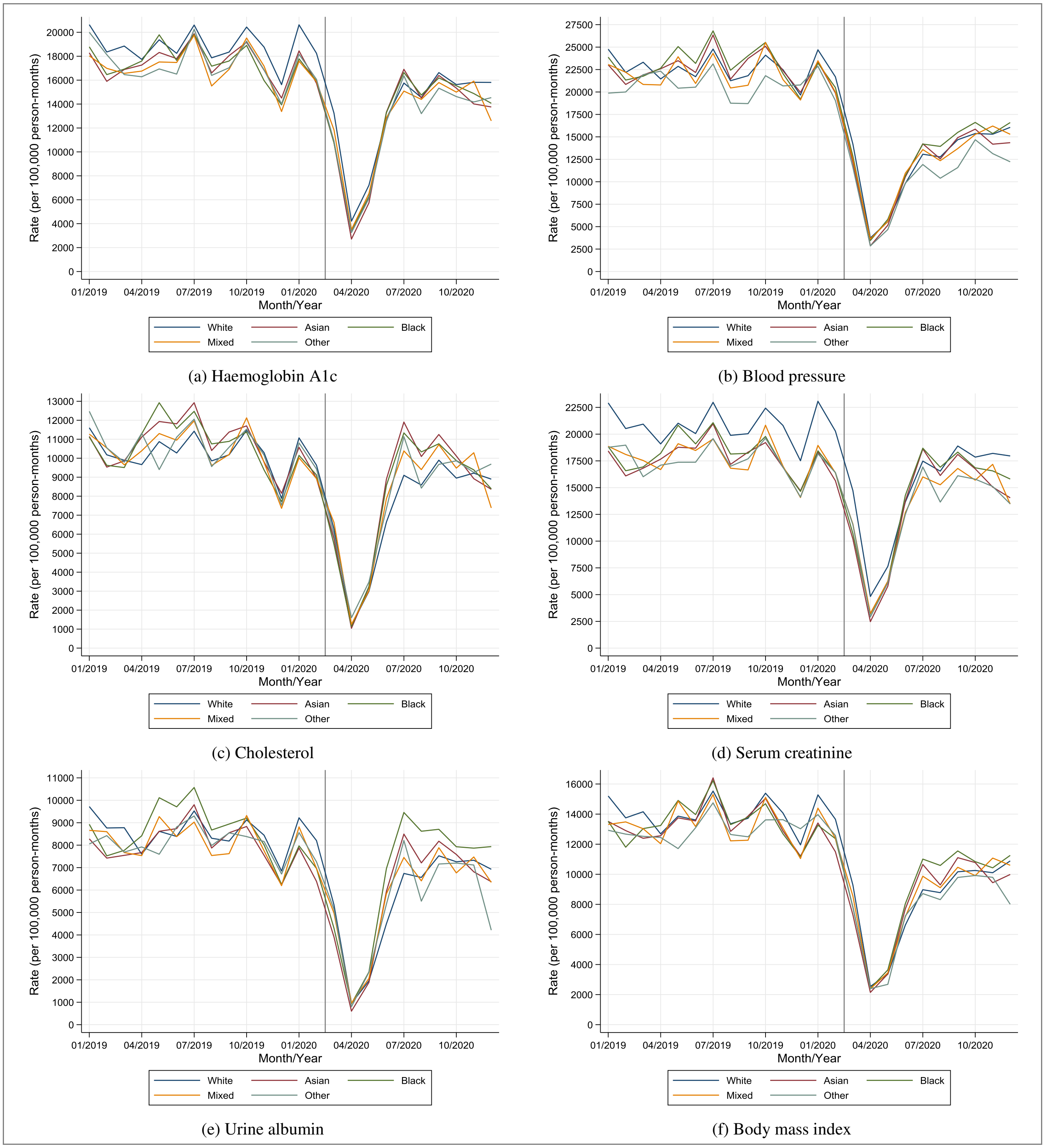

(a) Haemoglobin A1c
(b) Blood pressure
(c) Cholesterol
(d) Serum creatinine
(e) Urine albumin
(f) Body mass index

- The vertical line at March 1, 2020, separates the rates in primary care before and after the start of the COVID-19 pandemic, and x-axis markers indicate mid-months.

Carr MJ, *et al. BMJ Qual Saf* 2022; 31:503–514. doi: 10.1136/bmjqs-2021-013613

Supplementary Figure 9. Observed ethnicity-stratified care process rates in people with type 2 diabetes during 2019 and 2020, in Northern Ireland, Scotland, and Wales

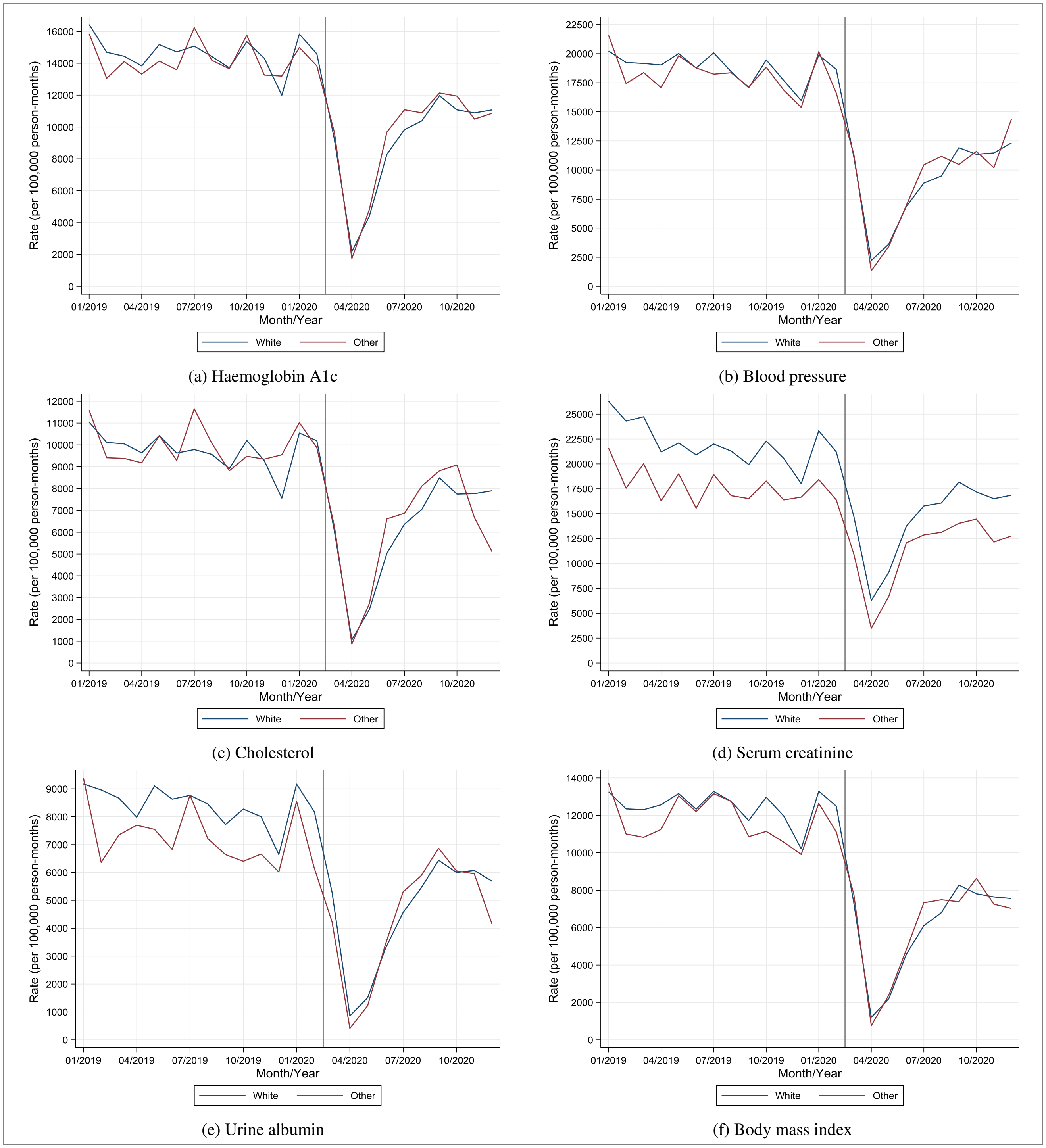

(a) Haemoglobin A1c (b) Blood pressure (c) Cholesterol (d) Serum creatinine (e) Urine albumin (f) Body mass index

- The vertical line at March 1, 2020, separates the rates in primary care before and after the start of the COVID-19 pandemic, and x-axis markers indicate mid-months.

Supplementary Figure 10. Observed ethnicity-stratified rates of new medication initiation in people with type 2 diabetes during 2019 and 2020, in England

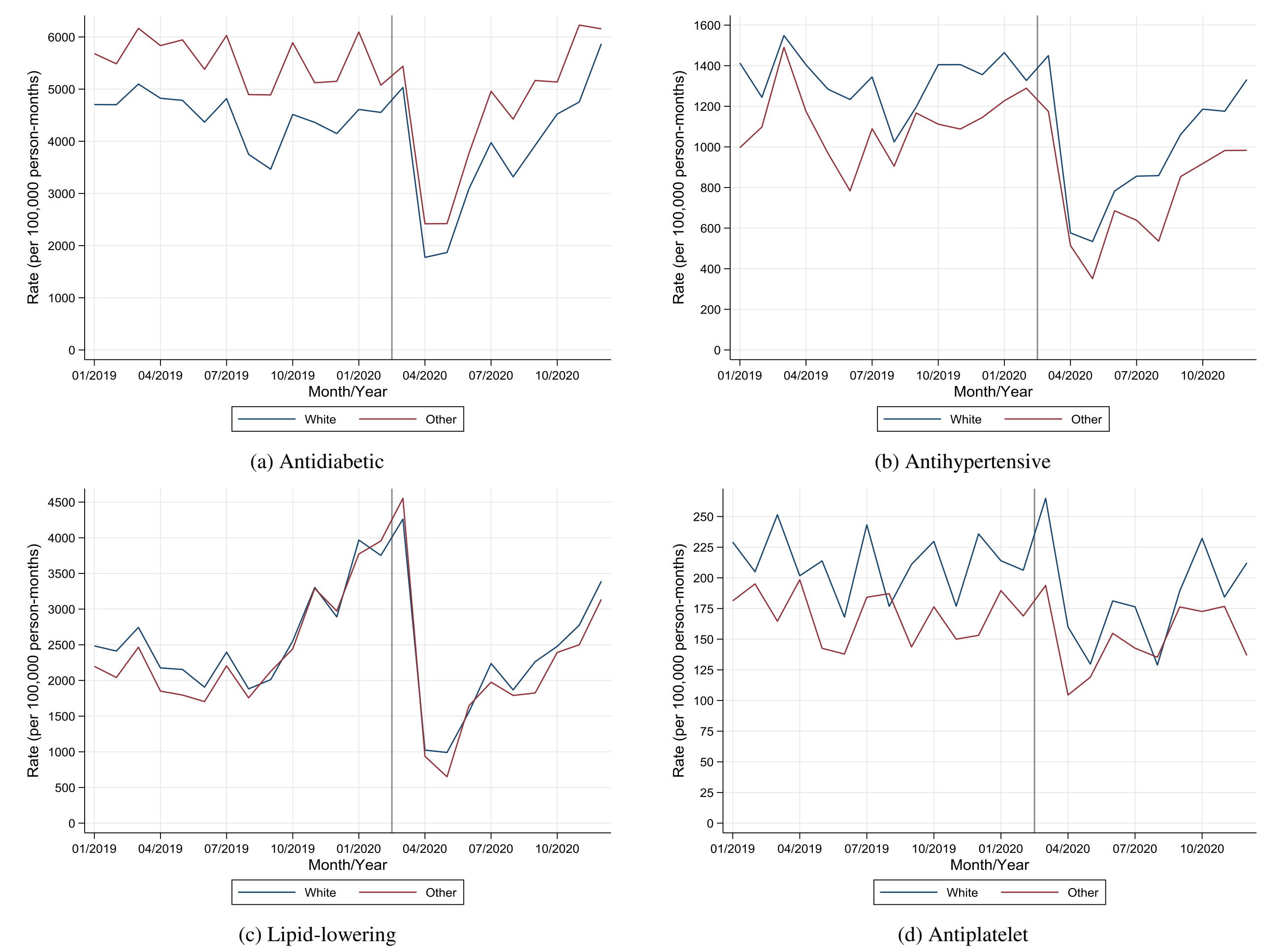

(a) Antidiabetic

(b) Antihypertensive

(c) Lipid-lowering

(d) Antiplatelet

- The vertical line at March 1, 2020, separates the rates in primary care before and after the start of the COVID-19 pandemic, and x-axis markers indicate mid-months.

APPENDICES: Clinical and Drug Prescription Codes

Part A1. SNOMED CT codes used to interrogate CPRD *Aurum*

Appendix 1. SNOMED CT codes for diagnoses of type 2 diabetes

SNOMED CT Concept	SNOMED CT Description	Term
190388001	292576013	Type II diabetes mellitus with multiple complications
190388001	292577016	Type 2 diabetes mellitus with multiple complications
190388001	292578014	Non-insulin-dependent diabetes mellitus with multiple comps
190389009	292579018	Type 2 diabetes mellitus with ulcer
190389009	292580015	Non-insulin dependent diabetes mellitus with ulcer
190389009	292581016	Type II diabetes mellitus with ulcer
237599002	1223147012	Insulin treated Type II diabetes mellitus
237599002	1223148019	Insulin treated non-insulin dependent diabetes mellitus
237599002	2967820013	Insulin treated Type 2 diabetes mellitus
313436004	457328010	Non-insulin-dependent diabetes mellitus without complication
313436004	457329019	Type 2 diabetes mellitus without complication
313436004	457330012	Type II diabetes mellitus without complication
314902007	459306016	Type II diabetes mellitus with peripheral angiopathy
314902007	459307013	Non-insulin-dependent d m with peripheral angiopath
314902007	459308015	Type 2 diabetes mellitus with peripheral angiopathy
314903002	459309011	Non-insulin dependent diabetes mellitus with arthropathy
314903002	459310018	Type 2 diabetes mellitus with arthropathy
314903002	459311019	Type II diabetes mellitus with arthropathy
314904008	459312014	Type II diabetes mellitus with neuropathic arthropathy
314904008	459313016	Type 2 diabetes mellitus with neuropathic arthropathy
314904008	459314010	Non-insulin dependent d m with neuropathic arthropathy
395204000	1488898011	Hyperosmolar non-ketotic state in type 2 diabetes mellitus
395204000	1667891000000113	Hyperosmolar non-ketotic state in type II diabetes mellitus
420279001	2615535015	Non-insulin-dependent diabetes mellitus with renal comps
420279001	2618208012	Type 2 diabetes mellitus with renal complications
420279001	3013392012	Type II diabetes mellitus with renal complications
420279001	3013392012	Type II diabetes mellitus with nephropathy
420279001	3035432019	Type 2 diabetes mellitus with nephropathy
420279001	3035432019	Non-insulin dependent diabetes mellitus with nephropathy
420436000	2618201018	Type 2 diabetes mellitus with mononeuropathy
420436000	3697667019	Non-insulin dependent diabetes mellitus with mononeuropathy
420436000	3697667019	Type II diabetes mellitus with mononeuropathy
420715001	2967769016	Type II diabetes mellitus with persistent microalbuminuria
420715001	2967769016	Type 2 diabetes mellitus with persistent microalbuminuria
420756003	3035281015	Non-insulin depend diabetes mellitus with diabetic cataract
420756003	3688522015	Type 2 diabetes mellitus with diabetic cataract
420756003	3688522015	Type II diabetes mellitus with diabetic cataract
421326000	3695406015	Type II diabetes mellitus with neurological complications
421326000	3695406015	Non-insulin-dependent diabetes mellitus with neuro comps
421326000	3695406015	Type 2 diabetes mellitus with neurological complications

421631007	2618206011	Type II diabetes mellitus with gangrene
421631007	2618206011	Type 2 diabetes mellitus with gangrene
421631007	2967779019	Non-insulin dependent diabetes mellitus with gangrene
421750000	2967754017	Type 2 diabetes mellitus with ketoacidosis
421750000	2967754017	Type II diabetes mellitus with ketoacidosis
421779007	2618237011	Type 2 diabetes mellitus with exudative maculopathy
421779007	3698529010	Type II diabetes mellitus with exudative maculopathy
421847006	2618218019	Type 2 diabetes mellitus with ketoacidotic coma
421847006	2618218019	Type II diabetes mellitus with ketoacidotic coma
421986006	2967751013	Type 2 diabetes mellitus with persistent proteinuria
421986006	2967751013	Type II diabetes mellitus with persistent proteinuria
422034002	3035295017	Non-insulin-dependent diabetes mellitus with retinopathy
422034002	3699410014	Type 2 diabetes mellitus with retinopathy
422034002	3699410014	Type II diabetes mellitus with retinopathy
422099009	3698440016	Non-insulin-dependent diabetes mellitus with ophthalm comps
422099009	3698440016	Type II diabetes mellitus with ophthalmic complications
422099009	3698440016	Type 2 diabetes mellitus with ophthalmic complications
422166005	2623039019	NIDDM with peripheral circulatory disorder
44054006	197761014	Type 2 diabetes mellitus
44054006	493773010	Non-insulin dependent diabetes mellitus
44054006	493773010	NIDDM - Non-insulin dependent diabetes mellitus
44054006	493774016	Type II diabetes mellitus
443694000	2842387018	Non-insulin dependent diabetes mellitus - poor control
443694000	2921019012	Type 2 diabetes mellitus - poor control
443694000	2921019012	Type II diabetes mellitus - poor control
713703005	3698412014	Type II diabetes mellitus with gastroparesis
713703005	3698412014	Type 2 diabetes mellitus with gastroparesis
713706002	3297353013	Type 2 diabetes mellitus with polyneuropathy
713706002	3297353013	Type II diabetes mellitus with polyneuropathy
713706002	3297353013	Non-insulin dependent diabetes mellitus with polyneuropathy
719216001	3316336015	Type 2 diabetes mellitus with hypoglycaemic coma
719216001	3316336015	Type II diabetes mellitus with hypoglycaemic coma
719216001	3316336015	Non-insulin dependent diabetes mellitus with hypoglyca coma
791000119109	2967590010	Angina associated with type II diabetes mellitus
791000119109	2967662015	Diabetic angina pectoris associated with type 2 diabetes mellitus
791000119109	2967773018	Angina associated with type 2 diabetes mellitus

Appendix 2. SNOMED CT codes for haemoglobin A1c monitoring

SNOMED CT Concept	SNOMED CT Description	Term
109871000006109	109871000006113	Test request : Haemoglobin A1c
165679005	2769803019	Hemoglobin A1c less than 7% indicating good diabetic control
997671000000106	2563461000000115	Blood glucose level
1003671000000109	2560611000000113	Haemoglobin A1c level
999791000000106	2572901000000113	Haemoglobin A1c level - IFCC standardised
446074002	1725291000000118	HbA1c target level - IFCC standardised
1927791000006103	1927791000006119	Raised HbA1c level
1049301000000100	2643101000000114	HbA1c level (diagnostic reference range) - IFCC standardised
1049321000000109	2643141000000112	HbA1c level (monitoring ranges) - IFCC standardised
1013511000000100	2574561000000110	Total glycosylated haemoglobin level
408591000	1739591000000117	HBA1c target
1010951000000100	2557081000000116	Haemoglobin A1c (diagnostic reference range)
1010941000000103	2582421000000115	Haemoglobin A1c (monitoring ranges)
1011001000000107	2562071000000110	Haemoglobin A quantitation
165679005	257619018	Hb. A1C < 7% - good control
165680008	257620012	Hb. A1C 7-10% - borderline
165681007	257621011	Hb. A1C > 10% - bad control
269823000	404444016	Hb. A1C - diabetic control NOS
166892002	259312011	Random blood sugar raised
166918003	259350017	Blood glucose 7-9.9 mmol/L
166919006	259351018	Blood glucose 10-13.9 mmol/L
43396009	72395012	Haemoglobin A1c measurement
43396009	72394011	Hemoglobin A1c measurement
43396009	493589010	HbA1c - Haemoglobin A1c level
43396009	493590018	HbA1c - Hemoglobin A1c level
43396009	129761000000114	Haemoglobin A1c level
269823000	1206383011	Hb. A1C - diabetic control
997681000000108	2572691000000111	Fasting blood glucose level
1022431000000105	2553471000000116	Hb estimation
1019411000000102	2566151000000118	Haemoglobin variant test
1003681000000106	2577721000000116	Haemoglobin A1 level
365845005	1206639010	HbA1 - diabetic control
1019551000000104	2583661000000115	HbA1 level (DCCT aligned)
1019431000000105	2566161000000115	HbA1c level (DCCT aligned)
165679005	4595261000006118	Haemoglobin A1c less than 7% indicating good diabetic control
165679005	2772122013	Hemoglobin A1c (HbA1c) less than 7% indicating good diabetic control
165679005	2773783018	Haemoglobin A1c (HbA1c) less than 7% indicating good diabetic control
165680008	2768431010	Hemoglobin A1c between 7%-10% indicating borderline diabetic control
165680008	2770714019	Hemoglobin A1c (HbA1c) between 7%-10% indicating borderline diabetic control
165680008	2772787010	Haemoglobin A1c (HbA1c) between 7%-10% indicating borderline diabetic control

165681007	2767753013	Haemoglobin A1c (HbA1c) greater than 10% indicating poor diabetic control
165681007	2767755018	Hemoglobin A1c greater than 10% indicating poor diabetic control
165681007	2773408014	Hemoglobin A1c (HbA1c) greater than 10% indicating poor diabetic control
1019361000000108	2569641000000116	Haemoglobin A
997671000000106	2563461000000115	Blood glucose result
997671000000106	2563461000000115	Blood sugar result
269823000	404443010	Hemoglobin A1C - diabetic control interpretation
365845005	489331011	Hemoglobin A1C - diabetic control finding
997681000000108	2572691000000111	Fasting blood sugar
408591000	2160141012	HBA1c target
408591000	2163995019	Target HbA1c level
408591000	2621920018	Haemoglobin A1c (HBA1c) target
408591000	2621921019	Hemoglobin A1c (HBA1c) target
408591000	2914480010	Hemoglobin A1c target
446074002	2883677017	Haemoglobin A1c target value using International Federation of Clinical Chemistry and Laboratory Medicine standardised method
446074002	2883676014	Hemoglobin A1c target value using International Federation of Clinical Chemistry and Laboratory Medicine standardized method
446074002	1725281000000115	HbA1c (haemoglobin A1c) target - IFCC (International Federation of Clinical Chemistry and Laboratory Medicine) standardised
446074002	1728751000000114	Haemoglobin A1c target - International Federation of Clinical Chemistry and Laboratory Medicine standardised
269823000	1206383011	Glycated haemoglobin
269823000	1206383011	Glycosylated Hb
1022431000000105	2553471000000116	Haemoglobin estimation
165679005	2770693019	HbA1 < 7% - good control
165681007	2767754019	HbA1 > 10% - bad control
165680008	2768430011	HbA1 7 - 10% - borderline control
443694000	2921019012	Type II diabetes mellitus - poor control

Appendix 3. SNOMED CT codes for blood pressure monitoring

SNOMED CT Concept	SNOMED CT Description	Term
1009231000006100	1009231000006116	24 hr blood pressure abnormal
1009241000006105	1009241000006114	24 hr blood pressure normal
72313002	120159016	Systolic blood pressure
125231000006103	125231000006119	Standing Diastolic Bp
125251000006105	125251000006114	Standing Systolic Bp
715051000000108	1566291000000115	BP screen administration
448678005	2900160016	Application of ambulatory blood pressure monitor
400974009	1780182014	Standing systolic blood pressure
400975005	1780183016	Standing diastolic blood pressure
1805081000006105	1805081000006114	Blood pressure monitoring in both arms
1808011000006107	1808011000006111	Systolic blood pressure - left arm
1808021000006104	1808021000006115	Diastolic blood pressure - left arm
1808041000006106	1808041000006110	Systolic blood pressure - right arm
1808051000006108	1808051000006112	Diastolic blood pressure - right arm
1928511000006100	1928511000006116	Baseline systolic blood pressure
1928521000006108	1928521000006112	Baseline diastolic blood pressure
1993951000006106	1993951000006110	Home diastolic blood pressure
1994021000006104	1994021000006115	Home systolic blood pressure
2011941000006103	2011941000006119	Blood pressure recorded in community
814081000000101	2122371000000114	Diastolic blood pressure centile
814101000000107	2122411000000113	Systolic blood pressure centile
407554009	2159155011	Sitting systolic blood pressure
407555005	2159156012	Sitting diastolic blood pressure
407556006	2159157015	Lying systolic blood pressure
407557002	2159158013	Lying diastolic blood pressure
135840009	216600016	Blood pressure monitoring
924481000000109	2371211000000110	Self-monitoring of blood pressure
928021000000108	2378631000000110	Baseline blood pressure
716632005	3306936015	Baseline diastolic blood pressure
716579001	3306694016	Baseline systolic blood pressure
413153004	2474699019	Blood pressure recorded by patient at home
413606001	2533615017	Average home systolic blood pressure
413605002	2533636015	Average home diastolic blood pressure
163020007	254063019	O/E - blood pressure reading
163020007	254064013	O/E - blood pressure
163020007	254065014	O/E - BP reading
163023009	254068011	O/E - BP reading low
163029008	254074011	O/E - BP reading:postural drop
163033001	254079018	Lying blood pressure reading
163034007	254080015	Standing blood pressure reading
163035008	254083018	Sitting blood pressure reading
163037000	254085013	O/E - BP labile
163020007	254063019	O/E-blood pressure reading NOS
164783007	256343017	Ambulatory blood pressure recording

1036531000000108	2616321000000118	Non-invasive central blood pressure
1036551000000101	2616361000000114	Non-invasive central systolic blood pressure
1036571000000105	2616401000000117	Non-invasive central diastolic blood pressure
170599006	264498011	24 hr blood pressure monitoring
198081000000101	298281000000114	Ambulatory systolic blood pressure
198091000000104	298311000000112	Ambulatory diastolic blood pressure
24184005	1784950015	[D]Raised blood pressure reading
271870002	406863012	[D]Low blood pressure reading
314440001	458803012	Average systolic blood pressure
314445006	458808015	Average night interval systolic blood pressure
314446007	458809011	Average day interval systolic blood pressure
314449000	458812014	Average 24 hour systolic blood pressure
314453003	458816012	Average diastolic blood pressure
314460009	458823013	Average night interval diastolic blood pressure
314461008	458824019	Average day interval diastolic blood pressure
314462001	458825018	Average 24 hour diastolic blood pressure
314956000	459379010	Borderline blood pressure
274785000	410648013	[V]Examination of blood pressure
251074006	1224586010	Non-invasive mean blood pressure
1091811000000102	2734671000000117	Diastolic blood pressure
335661000000109	622341000000118	Self measured blood pressure reading
24184005	196353013	High blood pressure
501631000000108	895081000006112	Nonspecific low BP reading [D]
923841000006109	923841000006113	O/E - blood pressure reading
961661000006104	961661000006115	Elevated pulse/respirations/blood pressure
961921000006100	961921000006116	Abnormal blood pressure reading

Appendix 4. SNOMED CT codes for cholesterol monitoring

SNOMED CT Concept	SNOMED CT Description	Term
107301000006104	107301000006115	Target serum cholesterol
1015271000000109	2565581000000114	Serum HDL:non-HDL cholesterol ratio
1005691000000109	2551281000000116	Serum triglycerides
1017161000000104	2552911000000113	Plasma total cholesterol level
1024231000000104	2567531000000117	Fluid sample cholesterol level
1014501000000104	2552501000000118	Calculated LDL cholesterol level
395153009	1488851015	Pre-treatment serum cholesterol level
1005671000000105	2551271000000118	Serum cholesterol
1822511000006106	1822511000006110	Test request : Serum HDL cholesterol level
121868005	186091018	Total cholesterol measurement
1864161000006103	1864161000006119	Test request : Serum triglycerides
1879211000006101	1879211000006117	Test request : Serum cholesterol
1916571000006101	1916571000006117	Serum triglyceride/HDL cholesterol ratio
920471000000100	2362791000000114	Estimated serum non-HDL cholesterol level
1083861000000102	2716101000000116	Serum HDL cholesterol:triglyceride ratio
1015881000000105	2583111000000110	Lipid screen
1006191000000106	2585861000000117	Serum non high density lipoprotein cholesterol level
994351000000103	2581451000000115	Serum total cholesterol level
1016381000000109	2583231000000110	Fluid sample triglyceride level
166828006	259227014	Serum cholesterol normal
166829003	259228016	Serum cholesterol borderline
166830008	259229012	Serum cholesterol raised
166831007	259230019	Serum cholesterol very high
1005681000000107	2577901000000110	Serum HDL cholesterol level
1022191000000100	2566581000000116	Serum LDL cholesterol level
1003441000000101	2607471000000113	Serum VLDL cholesterol level
1026441000000100	2599591000000110	Serum cholesterol studies
1026451000000102	2599931000000119	Serum fasting HDL cholesterol level
1026461000000104	2599971000000117	Serum random HDL cholesterol level
1026471000000106	2599941000000111	Serum fasting LDL cholesterol level
1026481000000108	2599981000000115	Serum random LDL cholesterol level
1028551000000102	2602701000000112	Total cholesterol:HDL ratio
1005671000000105	2551271000000118	Serum cholesterol NOS
166846000	259256014	Serum triglycerides normal
166847009	259257017	Serum triglycerides borderline
166848004	259258010	Serum triglycerides raised
1026491000000105	2599951000000114	Serum fasting triglyceride level
1026501000000104	2599961000000112	Serum random triglyceride level
365796000	489238019	Serum triglycerides NOS
1028831000000106	2600041000000114	Plasma random HDL cholesterol level
1028841000000102	2600031000000117	Plasma fasting HDL cholesterol level
1028851000000104	2600051000000112	Plasma random LDL cholesterol level
1028861000000101	2600061000000110	Plasma fasting LDL cholesterol level
1010601000000105	2564971000000119	Plasma triglyceride level

Carr MJ, *et al. BMJ Qual Saf* 2022; 31:503–514. doi: 10.1136/bmjqs-2021-013613

1028871000000108	2600071000000115	Plasma random triglyceride level
1031321000000109	2600081000000118	Plasma fasting triglyceride level
1031421000000101	2602691000000112	HDL : total cholesterol ratio
84698008	34521000033114	Cholesterol
238084008	356884010	Very low density lipoprotinaemia
1083761000000106	2715881000000113	Serum fasting total cholesterol
1003411000000102	2607481000000110	Serum VLDL triglyceride level
313811003	457911015	Cholesterol/HDL ratio
1003381000000104	2561141000000112	Plasma VLDL triglyceride level
1015681000000109	2574851000000110	Serum cholesterol/HDL ratio
1015691000000106	2557701000000111	Plasma cholesterol/HDL ratio
1015701000000106	2565681000000110	Serum cholesterol/LDL ratio
1015711000000108	2580131000000114	Plasma cholesterol/LDL ratio
1015721000000102	2557711000000113	Serum cholesterol/VLDL ratio
1015731000000100	2580141000000117	Plasma cholesterol/VLDL ratio
1010581000000101	2557051000000110	Plasma HDL cholesterol level
1010591000000104	2586471000000117	Plasma LDL cholesterol level
1030411000000101	2595211000000119	Non HDL cholesterol level
526321000033107	526321000033111	Triglycerides
667191000006107	667191000006111	Fasting serum cholesterol
398036000	1783622013	Low-density-lipoprotein-type (LDL) hyperlipoproteinaemia
398036000	1783622013	Low density lipoproteinaemia
854481000006106	854481000006110	Fasting triglycerides
855771000006102	855771000006118	HDL cholesterol level
857141000006100	857141000006116	LDL cholesterol level
850991000000104	2204851000000114	Triglyceride level
987321000006108	987321000006112	Pre-treatment fasting HDL cholesterol

Appendix 5. SNOMED CT codes for serum creatinine monitoring

SNOMED CT Concept	SNOMED CT Description	Term
109761000006107	109761000006111	Test request : Creatinine
167183007	259691011	Creatinine clearance-glom filt abnormal
167182002	259690012	Creatinine clearance-glom filt normal
857971000000104	2220291000000118	eGFR (estimated glomerular filtration rate) using CKD-Epi (Chronic Kidney Disease Epidemiology Collaboration) formula per 1.73 square metres
80274001	2773141000000116	GFR - glomerular filtration rate
80274001	133205018	Glomerular filtration rate
996231000000108	2581641000000115	GFR (glomerular filtration rate) calculated by abbreviated Modification of Diet in Renal Disease Study Group calculation adjusted for African American origin
1744631000006108	1744631000006112	Estimated glomerular filtration rate (eGFR) monitoring
1030991000000102	2603641000000111	Urine protein/creatinine index
1854991000006103	1854991000006119	EGFR 3 month repeat test for CKD confirmatory
1864131000006107	1864131000006111	Test request : Creatinine clearance test
857971000000104	2220261000000112	Estimated glomerular filtration rate using Chronic Kidney Disease Epidemiology Collaboration formula
1019801000000102	2575411000000110	24 hour urine creatinine output
197690007	303978013	Rapidly progressive nephritic syndrome, minor glomerular abnormality
950181000000106	2428021000000111	CKD G4A1 - chronic kidney disease with glomerular filtration rate category G4 and albuminuria category A1
950211000000107	2428091000000114	CKD G4A2 - chronic kidney disease with glomerular filtration rate category G4 and albuminuria category A2
950231000000104	2428141000000115	CKD G4A3 - chronic kidney disease with glomerular filtration rate category G4 and albuminuria category A3
950251000000106	2428191000000113	CKD G5A1 - chronic kidney disease with glomerular filtration rate category G5 and albuminuria category A1
950291000000103	2428281000000117	CKD G5A2 - chronic kidney disease with glomerular filtration rate category G5 and albuminuria category A2
950311000000102	2428331000000110	CKD G5A3 - chronic kidney disease with glomerular filtration rate category G5 and albuminuria category A3
949401000000103	2426331000000114	CKD G1A1 - chronic kidney disease with glomerular filtration rate category G1 and albuminuria category A1
949421000000107	2426381000000113	CKD G1A2 - chronic kidney disease with glomerular filtration rate category G1 and albuminuria category A2
949481000000108	2426511000000114	CKD G1A3 - chronic kidney disease with glomerular filtration rate category G1 and albuminuria category A3
949521000000108	2426601000000111	CKD G2A1 - chronic kidney disease with glomerular filtration rate category G2 and albuminuria category A1
949561000000100	2426691000000116	CKD G2A2 - chronic kidney disease with glomerular filtration rate category G2 and albuminuria category A2
949621000000109	2426821000000118	CKD G2A3 - chronic kidney disease with glomerular filtration rate category G2 and albuminuria category A3
949881000000106	2427381000000110	CKD G3aA1 - chronic kidney disease with glomerular filtration rate category G3a and albuminuria category A1
949901000000109	2427401000000110	CKD G3aA2 - chronic kidney disease with glomerular filtration rate category G3a and albuminuria category A2

949921000000100	2427451000000111	CKD G3aA3 - chronic kidney disease with glomerular filtration rate category G3a and albuminuria category A3
950061000000103	2427751000000117	CKD G3bA1 - chronic kidney disease with glomerular filtration rate category G3b and albuminuria category A1
950081000000107	2427801000000112	CKD G3bA2 - chronic kidney disease with glomerular filtration rate category G3b and albuminuria category A2
950101000000101	2427851000000113	CKD G3bA3 - chronic kidney disease with glomerular filtration rate category G3b and albuminuria category A3
1011491000000107	2565071000000119	eGFR (estimated glomerular filtration rate) using cystatin C Chronic Kidney Disease Epidemiology Collaboration equation per 1.73 square metres
1011481000000105	2579541000000113	eGFR (estimated glomerular filtration rate) using creatinine Chronic Kidney Disease Epidemiology Collaboration equation per 1.73 square metres
968191000000100	2465711000000116	Estimated creatinine clearance (Cockcroft-Gault formula)
395680003	2470380013	Estimated creatinine clearance
166714005	259050010	Serum creatinine abnormal
166715006	259051014	Serum creatinine low
166716007	259052019	Serum creatinine normal
166717003	259053012	Serum creatinine raised
1000731000000107	2577271000000111	Serum creatinine NOS
1015981000000103	2580171000000111	Creatinine clearance test
167182002	2666654019	Creatinine clearance-glomerular filtration normal
167183007	2666655018	Creatinine clearance-glomerular filtration abnormal
197593004	303832016	Nephrotic syndrome, minor glomerular abnormality
197594005	303833014	Nephrotic syndrome, focal and segmental glomerular lesions
231131000000104	371441000000114	Glomerular filtration rate testing
1000731000000107	2577271000000111	Serum creatinine level
197679002	303967017	[X]Isolated proteinuria, with oth specif morpholog changes
390361000000103	891551000000118	[X]Isolated proteinuria, with unspecified morphological changes
64212008	106739010	[X]Unspecified nephritic syndrome, diffuse concentric glomerulonephritis
1000981000000109	2584361000000111	Corrected plasma creatinine level
1000991000000106	2571851000000113	Corrected serum creatinine level
1001011000000107	2584381000000119	Plasma creatinine level
167182002	4602231000006112	Creatinine clearance-glomerular filtration normal
167183007	4602251000006117	Creatinine clearance-glomerular filtration abnormal
1015981000000103	2580171000000111	Creatinine clearance
1015981000000103	2580171000000111	Creatinine clearance-glom filt
1003271000000106	2573321000000113	Urine creatinine level
857971000000104	8294801000006111	Estimated glomerular filtration rate using Chronic Kidney Disease Epidemiology Collaboration formula
857971000000104	2221401000000117	eGFR (estimated glomerular filtration rate) using CKD-Epi (Chronic Kidney Disease Epidemiology Collaboration) formula
949401000000103	2426361000000116	Chronic kidney disease with glomerular filtration rate category G1 and albuminuria category A1
949421000000107	2426411000000110	Chronic kidney disease with glomerular filtration rate category G1 and albuminuria category A2
949481000000108	2426541000000110	Chronic kidney disease with glomerular filtration rate category G1

		and albuminuria category A3
949521000000108	2426671000000115	Chronic kidney disease with glomerular filtration rate category G2 and albuminuria category A1
949561000000100	2426801000000110	Chronic kidney disease with glomerular filtration rate category G2 and albuminuria category A2
949621000000109	2426851000000111	Chronic kidney disease with glomerular filtration rate category G2 and albuminuria category A3
949881000000106	2427351000000116	Chronic kidney disease with glomerular filtration rate category G3a and albuminuria category A1
949901000000109	2427431000000116	Chronic kidney disease with glomerular filtration rate category G3a and albuminuria category A2
949921000000100	2427481000000117	Chronic kidney disease with glomerular filtration rate category G3a and albuminuria category A3
950061000000103	2427781000000111	Chronic kidney disease with glomerular filtration rate category G3b and albuminuria category A1
950081000000107	2427831000000118	Chronic kidney disease with glomerular filtration rate category G3b and albuminuria category A2
950101000000101	2427881000000119	Chronic kidney disease with glomerular filtration rate category G3b and albuminuria category A3
950181000000106	2428071000000110	Chronic kidney disease with glomerular filtration rate category G4 and albuminuria category A1
950211000000107	2428121000000110	Chronic kidney disease with glomerular filtration rate category G4 and albuminuria category A2
950231000000104	2428171000000114	Chronic kidney disease with glomerular filtration rate category G4 and albuminuria category A3
950251000000106	2428261000000114	Chronic kidney disease with glomerular filtration rate category G5 and albuminuria category A1
950291000000103	2428311000000119	Chronic kidney disease with glomerular filtration rate category G5 and albuminuria category A2
950311000000102	2428361000000117	Chronic kidney disease with glomerular filtration rate category G5 and albuminuria category A3
1020291000000106	2569781000000116	GFR (glomerular filtration rate) calculated by abbreviated Modification of Diet in Renal Disease Study Group calculation
1032061000000108	2606391000000117	Creatinine level
993971000006101	993971000006117	Estimated creatinine clearance using Cockcroft-Gault equation

Carr MJ, *et al. BMJ Qual Saf* 2022; 31:503–514. doi: 10.1136/bmjqs-2021-013613

Appendix 6. SNOMED CT codes for urine albumin monitoring

SNOMED CT Concept	SNOMED CT Description	Term
110181000006108	110181000006112	Test request : Urinary Microalbumin
312975006	3672941010	Moderately increased albuminuria
1028811000000103	2600611000000116	Urine protein level
199481000000100	301611000000118	Diabetic monitoring - lower risk albumin excretion
199491000000103	301641000000117	Diabetic monitoring - higher risk albumin excretion
1023491000000104	2768311000000117	Albumin/creatinine ratio in urine
1016051000000101	2583141000000111	24 hour urine protein output
1002901000000100	2561061000000112	Overnight albumin excretion rate
1756971000006105	1756971000006114	Test request : Urine albumin:creatinine ratio
1807231000006107	1807231000006111	Urine microalbumin = trace
1807241000006102	1807241000006118	Urine microalbumin = +
1807251000006100	1807251000006116	Urine microalbumin = ++
1807261000006103	1807261000006119	Urine microalbumin = +++
1822701000006102	1822701000006118	Test request : 24 hour urine protein excretion test
1028571000000106	2599111000000112	24 hour urine protein excretion test
950181000000106	2428021000000111	CKD G4A1 - chronic kidney disease with glomerular filtration rate category G4 and albuminuria category A1
950211000000107	2428091000000114	CKD G4A2 - chronic kidney disease with glomerular filtration rate category G4 and albuminuria category A2
950231000000104	2428141000000115	CKD G4A3 - chronic kidney disease with glomerular filtration rate category G4 and albuminuria category A3
950251000000106	2428191000000113	CKD G5A1 - chronic kidney disease with glomerular filtration rate category G5 and albuminuria category A1
950291000000103	2428281000000117	CKD G5A2 - chronic kidney disease with glomerular filtration rate category G5 and albuminuria category A2
950311000000102	2428331000000110	CKD G5A3 - chronic kidney disease with glomerular filtration rate category G5 and albuminuria category A3
949401000000103	2426331000000114	CKD G1A1 - chronic kidney disease with glomerular filtration rate category G1 and albuminuria category A1
949421000000107	2426381000000113	CKD G1A2 - chronic kidney disease with glomerular filtration rate category G1 and albuminuria category A2
949481000000108	2426511000000114	CKD G1A3 - chronic kidney disease with glomerular filtration rate category G1 and albuminuria category A3
949521000000108	2426601000000111	CKD G2A1 - chronic kidney disease with glomerular filtration rate category G2 and albuminuria category A1
949561000000100	2426691000000116	CKD G2A2 - chronic kidney disease with glomerular filtration rate category G2 and albuminuria category A2
949621000000109	2426821000000118	CKD G2A3 - chronic kidney disease with glomerular filtration rate category G2 and albuminuria category A3
949881000000106	2427381000000110	CKD G3aA1 - chronic kidney disease with glomerular filtration rate category G3a and albuminuria category A1
949901000000109	2427401000000110	CKD G3aA2 - chronic kidney disease with glomerular filtration rate category G3a and albuminuria category A2
949921000000100	2427451000000111	CKD G3aA3 - chronic kidney disease with glomerular filtration rate category G3a and albuminuria category A3
950061000000103	2427751000000117	CKD G3bA1 - chronic kidney disease with glomerular filtration rate category G3b and albuminuria category A1

950081000000107	2427801000000112	CKD G3bA2 - chronic kidney disease with glomerular filtration rate category G3b and albuminuria category A2
950101000000101	2427851000000113	CKD G3bA3 - chronic kidney disease with glomerular filtration rate category G3b and albuminuria category A3
994621000000103	2567991000000113	Urine microalbumin profile
1011581000000106	2565101000000111	Random urine protein level
1007881000000101	2573851000000112	Urine dipstick test
1030631000000106	2603511000000111	Urine protein test
167273002	259803019	Urine protein test negative
167274008	259804013	Urine protein test = trace
167275009	259805014	Urine protein test = +
167276005	259806010	Urine protein test = ++
167277001	259807018	Urine protein test = +++
167278006	259808011	Urine protein test = ++++
271346009	406084015	Urine protein test NOS
167582007	260307015	Urine microalbumin positive
167583002	260308013	Urine microalbumin negative
8022000	14262017	Exercise albuminuria
8022000	14263010	Athletic proteinuria
8022000	14264016	Effort proteinuria
29738008	49757010	Abnormal presence of protein in urine
199481000000100	1749411000000114	Diabetes monitoring lower risk albumin excretion
199491000000103	1749311000000117	Diabetes monitoring higher risk albumin excretion
32482005	54232010	Orthostatic albuminuria
32482005	485236012	Postural albuminuria
29738008	49755019	[D]Proteinuria
274769005	410631017	Albuminuria
29738008	49755019	[D]Proteinuria NOS
71628007	118999019	Abnormal presence of albumin
998691000000106	2569351000000111	Microalbumin excretion rate
1030631000000106	2603511000000111	Urine dipstick for protein
1000941000000101	2571821000000115	Albumin excretion rate
312975006	456794012	Microalbuminuria
1019891000000109	2569701000000114	24 hour urine albumin output
1028271000000109	2602141000000114	Albumin/creatinine ratio
1010251000000109	2586441000000111	Urine microalbumin level
29738008	49755019	Proteinuria
271346009	406084015	Urine dipstick for protein
271346009	465541000000111	Urine dipstick for protein test
1027791000000103	2602161000000110	Urine microalbumin/creatinine ratio
420715001	2618209016	Persistent microalbuminuria associated with type II diabetes mellitus
1003301000000109	2556151000000113	Urine albumin level
1023491000000104	2553591000000116	Urine albumin:creatinine ratio
199481000000100	8059261000006114	Diabetes monitoring lower risk albumin excretion
199491000000103	8059281000006116	Diabetes monitoring higher risk albumin excretion
949401000000103	2426361000000116	Chronic kidney disease with glomerular filtration rate category G1

		and albuminuria category A1
949421000000107	2426411000000110	Chronic kidney disease with glomerular filtration rate category G1 and albuminuria category A2
949481000000108	2426541000000110	Chronic kidney disease with glomerular filtration rate category G1 and albuminuria category A3
949521000000108	2426671000000115	Chronic kidney disease with glomerular filtration rate category G2 and albuminuria category A1
949561000000100	2426801000000110	Chronic kidney disease with glomerular filtration rate category G2 and albuminuria category A2
949621000000109	2426851000000111	Chronic kidney disease with glomerular filtration rate category G2 and albuminuria category A3
949881000000106	2427351000000116	Chronic kidney disease with glomerular filtration rate category G3a and albuminuria category A1
949901000000109	2427431000000116	Chronic kidney disease with glomerular filtration rate category G3a and albuminuria category A2
949921000000100	2427481000000117	Chronic kidney disease with glomerular filtration rate category G3a and albuminuria category A3
950061000000103	2427781000000111	Chronic kidney disease with glomerular filtration rate category G3b and albuminuria category A1
950081000000107	2427831000000118	Chronic kidney disease with glomerular filtration rate category G3b and albuminuria category A2
950101000000101	2427881000000119	Chronic kidney disease with glomerular filtration rate category G3b and albuminuria category A3
950181000000106	2428071000000110	Chronic kidney disease with glomerular filtration rate category G4 and albuminuria category A1
950211000000107	2428121000000110	Chronic kidney disease with glomerular filtration rate category G4 and albuminuria category A2
950231000000104	2428171000000114	Chronic kidney disease with glomerular filtration rate category G4 and albuminuria category A3
950251000000106	2428261000000114	Chronic kidney disease with glomerular filtration rate category G5 and albuminuria category A1
950291000000103	2428311000000119	Chronic kidney disease with glomerular filtration rate category G5 and albuminuria category A2
950311000000102	2428361000000117	Chronic kidney disease with glomerular filtration rate category G5 and albuminuria category A3
1028811000000103	2600611000000116	Urine protein
1028271000000109	2602151000000112	Albumin / creatinine ratio
1028571000000106	2603351000000116	Protein measurement, urine, quantitative 24 hour
1030631000000106	2603501000000114	Urine dipstick for protein
420715001	2967769016	Persistent microalbuminuria associated with type 2 diabetes mellitus
420715001	2967769016	Type II diabetes mellitus with persistent microalbuminuria
658061000000102	933211000006112	DM + persist microalbuminuria

Appendix 7. SNOMED CT codes for recording body mass index

SNOMED CT Concept	SNOMED CT Description	Term
1009131000000104	2586361000000116	Sample weight
162690006	253576010	O/E - obese
162756007	253670011	O/E-height > 20% below average
162757003	253671010	O/E - height 10-20% < average
162758008	253672015	O/E -height within 10% average
162759000	253673013	O/E-height 10-20% over average
162760005	253674019	O/E -height > 20% over average
162764001	253678016	O/E - weight > 20% below ideal
162765000	253679012	O/E -weight 10-20% below ideal
162766004	253680010	O/E - weight within 10% ideal
162769006	253687013	O/E - Underweight
162863004	253847018	Body mass index index 25-29 - overweight
162864005	253848011	Body mass index 30+ - obesity
162879003	253866018	Height and weight
170794003	264752011	Initial obesity assessment
1728141000006104	1728141000006115	BMI centile
1910901000006100	1910901000006116	Estimated weight
1910911000006102	1910911000006118	Reported weight
1910921000006105	1910921000006114	Estimated height
1910931000006108	1910931000006112	Reported height
238131007	356960015	Overweight
248333004	370729019	O/E - height NOS
248333004	370729019	Standing height
248342006	370738017	Underweight
268522006	401534016	Obesity monitoring
268915006	402435016	O/E - weight 10-20% over ideal
268916007	402436015	O/E - weight greater than 20% over ideal
27113001	45352010	Body weight
27113001	45352010	O/E - weight NOS
275947003	411922013	O/E - overweight
307818003	451201014	Weight monitoring
310252000	453856012	Body mass index less than 20
35425004	59104012	Normal body mass index
400967004	1780175010	Baseline weight
408512008	2160062010	Body mass index 40+ - severely obese
412768003	2474325012	Body mass index 20-24 - normal
414916001	2535065012	Obesity
48499001	80800016	Increased body mass index
60621009	100716012	Body mass index
6497000	11777011	Decreased body mass index
713971000000102	1565211000000116	Obesity clinic administration
804271000000109	1798011000000116	Weight screening
846911000000109	2196031000000118	Baseline body mass index centile
846931000000101	2196071000000116	Baseline body mass index

908721000006107	908721000006111	[RFC] Overweight/ underweight
910251000006106	910251000006110	[RFC] Overweight
910261000006108	910261000006112	[RFC] Underweight
914721000000105	2350241000000116	Obese class I (body mass index 30.0 - 34.9)
914731000000107	2350261000000115	Obese class II (body mass index 35.0 - 39.9)
914741000000103	2350281000000112	Obese class III (body mass index equal to or greater than 40.0)
923831000006104	923831000006115	O/E - height
923851000006106	923851000006110	O/E - weight
957731000006103	957731000006119	Height weight & BP
981731000006101	981731000006117	Overweight: adult BMI 25.0; child BMI 95th percentile; or more
981741000006106	981741000006110	Underweight: adult BMI 18.5; child BMI 5th percentile; or less

Carr MJ, *et al. BMJ Qual Saf* 2022; 31:503–514. doi: 10.1136/bmjqs-2021-013613

Part A2. Drug prescription codes used to interrogate CPRD *Aurum*

Appendix 8. Product codes for prescriptions of antidiabetic medications

Product code	Term	Drug class
11441000033114	Acarbose 100mg tablets	AGi
11541000033110	Acarbose 50mg tablets	AGi
645241000033113	Glucobay 100mg tablets (Bayer Plc)	AGi
645341000033115	Glucobay 50mg tablets (Bayer Plc)	AGi
8959341000033111	Alogliptin 12.5mg tablets	DPP-4i
8959441000033117	Alogliptin 25mg tablets	DPP-4i
8959241000033118	Alogliptin 6.25mg tablets	DPP-4i
4269941000033119	Galvus 50mg tablets (Novartis Pharmaceuticals UK Ltd)	DPP-4i
4270041000033118	Galvus Tablets 100 mg	DPP-4i
4133341000033110	Januvia 100mg tablets (Merck Sharp & Dohme Ltd)	DPP-4i
7687541000033112	Januvia 25mg tablets (Merck Sharp & Dohme Ltd)	DPP-4i
7687341000033117	Januvia 50mg tablets (Merck Sharp & Dohme Ltd)	DPP-4i
6444041000033117	Linagliptin 5mg tablets	DPP-4i
6123341000033119	Onglyza 2.5mg tablets (AstraZeneca UK Ltd)	DPP-4i
5316741000033110	Onglyza 5mg tablets (AstraZeneca UK Ltd)	DPP-4i
6123241000033112	Saxagliptin 2.5mg tablets	DPP-4i
5316641000033118	Saxagliptin 5mg tablets	DPP-4i
4133241000033117	Sitagliptin 100mg tablets	DPP-4i
7687441000033111	Sitagliptin 25mg tablets	DPP-4i
7687241000033110	Sitagliptin 50mg tablets	DPP-4i
6444141000033118	Trajenta 5mg tablets (Boehringer Ingelheim Ltd)	DPP-4i
4269741000033117	Vildagliptin 50mg tablets	DPP-4i
4269841000033110	Vildagliptin Tablets 100 mg	DPP-4i
8959641000033115	Vipidia 12.5mg tablets (Takeda UK Ltd)	DPP-4i
8959741000033112	Vipidia 25mg tablets (Takeda UK Ltd)	DPP-4i
8959541000033116	Vipidia 6.25mg tablets (Takeda UK Ltd)	DPP-4i
8959841000033119	Alogliptin 12.5mg / Metformin 1g tablets	DPP4i/Metformin
4452541000033114	Eucreas 50mg/1000mg tablets (Novartis Pharmaceuticals UK Ltd)	DPP4i/Metformin
4452641000033110	Eucreas 50mg/850mg tablets (Novartis Pharmaceuticals UK Ltd)	DPP4i/Metformin
8028541000033115	Galvus Met Tablets 1 gram + 50 mg	DPP4i/Metformin
8028441000033116	Galvus Met Tablets 850 mg + 50 mg	DPP4i/Metformin
5132241000033116	Janumet 50mg/1000mg tablets (Merck Sharp & Dohme Ltd)	DPP4i/Metformin
5132141000033111	Janumet Tablets 50 mg + 500 mg	DPP4i/Metformin
8115941000033116	Jentadueto 2.5mg/1000mg tablets (Boehringer Ingelheim Ltd)	DPP4i/Metformin
8116041000033114	Jentadueto 2.5mg/850mg tablets (Boehringer Ingelheim Ltd)	DPP4i/Metformin
8242841000033114	Komboglyze 2.5mg/1000mg tablets (AstraZeneca UK Ltd)	DPP4i/Metformin
8242741000033116	Komboglyze 2.5mg/850mg tablets (AstraZeneca UK Ltd)	DPP4i/Metformin
8115741000033119	Linagliptin 2.5mg / Metformin 1g tablets	DPP4i/Metformin
8115841000033112	Linagliptin 2.5mg / Metformin 850mg tablets	DPP4i/Metformin
5576041000033115	Metformin 1g / Sitagliptin 50mg tablets	DPP4i/Metformin
8242641000033113	Saxagliptin 2.5mg / Metformin 1g tablets	DPP4i/Metformin
8242541000033112	Saxagliptin 2.5mg / Metformin 850mg tablets	DPP4i/Metformin

4452441000033113	Vildagliptin 50mg / Metformin 1g tablets	DPP4i/Metformin
4452341000033119	Vildagliptin 50mg / Metformin 850mg tablets	DPP4i/Metformin
8959941000033110	Vipdomet 12.5mg/1000mg tablets (Takeda UK Ltd)	DPP4i/Metformin
10951041000033114	Albiglutide 30mg powder and solvent for solution for injection pre-filled disposable devices	GLP-1ag
10951141000033113	Albiglutide 50mg powder and solvent for solution for injection pre-filled disposable devices	GLP-1ag
6388341000033110	Bydureon 2mg powder and solvent for prolonged-release suspension for injection vials (AstraZeneca UK Ltd)	GLP-1ag
10042641000033116	Bydureon Prolonged release suspension for injection 2 mg pen	GLP-1ag
4149641000033115	Byetta 10micrograms/0.04ml solution for injection 2.4ml pre-filled pens (AstraZeneca UK Ltd)	GLP-1ag
4149541000033116	Byetta 5micrograms/0.02ml solution for injection 1.2ml pre-filled pens (AstraZeneca UK Ltd)	GLP-1ag
10207841000033118	Dulaglutide 0.75mg/0.5ml solution for injection pre-filled disposable devices	GLP-1ag
10207941000033114	Dulaglutide 1.5mg/0.5ml solution for injection pre-filled disposable devices	GLP-1ag
10952341000033119	Eperzan 30mg powder and solvent for solution for injection pre-filled pens (GlaxoSmithKline UK Ltd)	GLP-1ag
10953041000033113	Eperzan 50mg powder and solvent for solution for injection pre-filled pens (GlaxoSmithKline UK Ltd)	GLP-1ag
4149441000033117	Exenatide 10micrograms/0.04ml solution for injection 2.4ml pre-filled disposable devices	GLP-1ag
6388241000033117	Exenatide 2mg powder and solvent for prolonged-release suspension for injection vials	GLP-1ag
4149341000033111	Exenatide 5micrograms/0.02ml solution for injection 1.2ml pre-filled disposable devices	GLP-1ag
10042541000033117	Exenatide Prolonged release suspension for injection 2 mg device	GLP-1ag
5131241000033110	Liraglutide 6mg/ml solution for injection 3ml pre-filled disposable devices	GLP-1ag
8268441000033115	Lixisenatide 10micrograms/0.2ml solution	GLP-1ag
8267341000033110	Lixisenatide 10micrograms/0.2ml solution for injection 3ml pre-filled disposable devices	GLP-1ag
8267441000033116	Lixisenatide 20micrograms/0.2ml solution for injection 3ml pre-filled disposable devices	GLP-1ag
8267541000033115	Lyxumia 10micrograms/0.2ml solution for injection 3ml pre-filled pens (Sanofi)	GLP-1ag
8268641000033118	Lyxumia 10micrograms/20micrograms treatment initiation pack (Sanofi)	GLP-1ag
8267641000033119	Lyxumia 20micrograms/0.2ml solution for injection 3ml pre-filled pens (Sanofi)	GLP-1ag
11919041000033113	Saxenda 6mg/ml solution for injection 3ml pre-filled pens (Novo Nordisk Ltd)	GLP-1ag
10208041000033112	Trulicity 0.75mg/0.5ml solution for injection pre-filled pens (Eli Lilly and Company Ltd)	GLP-1ag
10208141000033111	Trulicity 1.5mg/0.5ml solution for injection pre-filled pens (Eli Lilly and Company Ltd)	GLP-1ag
5131341000033117	Victoza 6mg/ml solution for injection 3ml pre-filled pens (Novo Nordisk Ltd)	GLP-1ag
10044541000033113	Insulin degludec 100units/ml / Liraglutide 3.6mg/ml solution for injection 3ml pre-filled disposable devices	GLP-1ag/Insulin
10044641000033114	Xultophy 100units/ml / 3.6mg/ml solution for injection 3ml pre-filled	GLP-1ag/Insulin

	pens (Novo Nordisk Ltd)	
10600641000033118	Abasaglar 100units/ml solution for injection 3ml cartridges (Eli Lilly and Company Ltd)	Insulin
10600741000033110	Abasaglar KwikPen 100units/ml solution for injection 3ml pre-filled pens (Eli Lilly and Company Ltd)	Insulin
11493341000033110	Actraphane Hm (Ge) Suspension For Injection 100 units/ml, 10 ml vial	Insulin
2952841000033116	Actrapid 100units/ml solution for injection 10ml vials (Novo Nordisk Ltd)	Insulin
7341000033112	Actrapid Mc Injection 100 units/1 ml	Insulin
2952941000033112	Actrapid NovoLet 100units/ml solution for injection (Novo Nordisk Ltd)	Insulin
2953041000033119	Actrapid Penfill 100units/ml solution for injection 3ml cartridges (Novo Nordisk Ltd)	Insulin
3345841000033110	Apidra 100units/ml solution for injection 10ml vials (Sanofi)	Insulin
3345941000033119	Apidra 100units/ml solution for injection 3ml cartridges (Sanofi)	Insulin
3963941000033117	Apidra 100units/ml solution for injection 3ml OptiClik cartridges (Sanofi)	Insulin
4258641000033112	Apidra 100units/ml solution for injection 3ml pre-filled SoloStar pens (Sanofi)	Insulin
3914341000033119	Apidra Optiset Pre-filled Disposable Pen 100 units/ml, 3 ml pen	Insulin
148841000033116	Biphasic Insulin Injection 100 units/ml	Insulin
3945141000033114	Exubera 1mg inhalation powder blisters (Pfizer Ltd)	Insulin
3945241000033119	Exubera 3mg inhalation powder blisters (Pfizer Ltd)	Insulin
12186441000033111	Fiasp 100units/ml solution for injection 10ml vials (Novo Nordisk Ltd)	Insulin
12186541000033112	Fiasp FlexTouch 100units/ml solution for injection 3ml pre-filled pens (Novo Nordisk Ltd)	Insulin
12186641000033113	Fiasp Penfill 100units/ml solution for injection 3ml cartridges (Novo Nordisk Ltd)	Insulin
727641000033113	Humaject (Humulin I) Preloaded pen 100 units/ml	Insulin
727141000033115	Humaject (Humulin M1) Preloaded pen	Insulin
727241000033110	Humaject (Humulin M2) Preloaded pen	Insulin
727441000033111	Humaject (Humulin M4) Preloaded pen	Insulin
727541000033112	Humaject (Humulin M5) Preloaded pen	Insulin
727341000033117	HumaJect M3 Pen 100units/ml suspension for injection (Eli Lilly and Company Ltd)	Insulin
727041000033119	HumaJect S Pen 100units/ml solution for injection (Eli Lilly and Company Ltd)	Insulin
720541000033111	Humalog 100units/ml solution for injection 1.5ml cartridges (Eli Lilly and Company Ltd)	Insulin
723241000033118	Humalog 100units/ml solution for injection 10ml vials (Eli Lilly and Company Ltd)	Insulin
1736841000033114	Humalog 100units/ml solution for injection 3ml cartridges (Eli Lilly and Company Ltd)	Insulin
12486241000033111	Humalog Junior KwikPen 100units/ml solution for injection 3ml pre-filled pens (Eli Lilly and Company Ltd)	Insulin
4608541000033115	Humalog KwikPen 100units/ml solution for injection 3ml pre-filled pens (Eli Lilly and Company Ltd)	Insulin
10252741000033119	Humalog KwikPen 200units/ml solution for injection 3ml pre-filled pens (Eli Lilly and Company Ltd)	Insulin
5403541000033118	Humalog Mix25 100units/ml suspension for injection 10ml vials (Eli Lilly and Company Ltd)	Insulin
1736941000033118	Humalog Mix25 100units/ml suspension for injection 3ml cartridges (Eli Lilly and Company Ltd)	Insulin

Carr MJ, *et al. BMJ Qual Saf* 2022; 31:503–514. doi: 10.1136/bmjqs-2021-013613

4608641000033119	Humalog Mix25 KwikPen 100units/ml suspension for injection 3ml pre-filled pens (Eli Lilly and Company Ltd)	Insulin
1737041000033117	Humalog Mix25 Pen 100units/ml suspension for injection 3ml pre-filled pens (Waymade Healthcare Plc)	Insulin
3922641000033111	Humalog Mix50 100units/ml suspension for injection 3ml cartridges (Eli Lilly and Company Ltd)	Insulin
4608741000033111	Humalog Mix50 KwikPen 100units/ml suspension for injection 3ml pre-filled pens (Eli Lilly and Company Ltd)	Insulin
2182141000033117	Humalog Mix50 Pen 100units/ml suspension for injection 3ml pre-filled pens (Sigma Pharmaceuticals Plc)	Insulin
2182341000033119	Humalog Pen 100units/ml solution for injection 3ml pre-filled pens (Sigma Pharmaceuticals Plc)	Insulin
725041000033113	Human Actraphane(Nova) Injection	Insulin
725941000033114	Human Actrapid Injection 100 units/ml	Insulin
4270841000033113	Human Actrapid Injection 500 units/ml, 10 ml vial	Insulin
721041000033110	Human Actrapid Penfill 100units/ml solution for injection 1.5ml cartridges (Novo Nordisk Ltd)	Insulin
720941000033117	Human Actrapid Penfill Cartridges (3 Ml) 100 units/ml	Insulin
9641000033112	Human Actrapid Preloaded pen 100 units/ml	Insulin
725141000033112	Human Initard 50/50 Insulin Injection 100 units/ml	Insulin
721641000033116	Human Insulatard Ge Injection 100 units/ml	Insulin
726641000033110	Human Insulatard Ge Penfill cartridges (3 ml) 100 units/ml	Insulin
726941000033115	Human Insulatard Ge Preloaded pen 100 units/ml	Insulin
1714041000033116	Human Insulatard Penfill 100units/ml suspension for injection 1.5ml cartridges (Novo Nordisk Ltd)	Insulin
1052941000033117	Human Mixtard 10 Injection	Insulin
1818141000033110	Human Mixtard 10 Penfill 100units/ml suspension for injection 1.5ml cartridges (Novo Nordisk Ltd)	Insulin
726141000033117	Human Mixtard 10 Penfill cartridges (3 ml)	Insulin
1061741000033116	Human Mixtard 10 Preloaded pen	Insulin
1053041000033110	Human Mixtard 20 Injection	Insulin
1818241000033115	Human Mixtard 20 Penfill 100units/ml suspension for injection 1.5ml cartridges (Novo Nordisk Ltd)	Insulin
726241000033112	Human Mixtard 20 Penfill cartridges (3 ml)	Insulin
1061841000033114	Human Mixtard 20 Preloaded pen	Insulin
722941000033116	Human Mixtard 30 Ge Injection	Insulin
1053541000033117	Human Mixtard 30 Injection (cartridges) 100 u/ml	Insulin
726041000033116	Human Mixtard 30 Injection 10 ml vial	Insulin
1818341000033113	Human Mixtard 30 Penfill 100units/ml suspension for injection 1.5ml cartridges (Novo Nordisk Ltd)	Insulin
726341000033119	Human Mixtard 30 Penfill cartridges (3 ml)	Insulin
1061641000033113	Human Mixtard 30 Preloaded pen	Insulin
1053141000033114	Human Mixtard 40 Injection	Insulin
1818441000033119	Human Mixtard 40 Penfill 100units/ml suspension for injection 1.5ml cartridges (Novo Nordisk Ltd)	Insulin
726441000033113	Human Mixtard 40 Penfill cartridges (3 ml)	Insulin
1061941000033118	Human Mixtard 40 Preloaded pen	Insulin
1053241000033119	Human Mixtard 50 Injection	Insulin
1818541000033118	Human Mixtard 50 Penfill 100units/ml suspension for injection 1.5ml	Insulin

	cartridges (Novo Nordisk Ltd)	
726541000033114	Human Mixtard 50 Penfill cartridges (3 ml)	Insulin
1062041000033112	Human Mixtard 50 Preloaded pen	Insulin
723941000033110	Human Monotard (Novo) Injection 100 units/ml	Insulin
725641000033119	Human Protaphane Injection 100u/ml	Insulin
721341000033112	Human Protaphane Penfill Injection 100 units/ml	Insulin
724941000033113	Human Ultratard Injection 100 units/ml	Insulin
725541000033115	Human Velosulin Injection 100 u/ml	Insulin
1507441000033119	Human Velosulin Injection 100 u/ml	Insulin
2868441000033119	Humulin 50/50 Injection 100 units/ml, 10 ml vial	Insulin
2868341000033113	Humulin 60/40 Injection 100 units/ml, 10 ml vial	Insulin
2868241000033115	Humulin 70/30 Injection 100 units/ml, 10 ml vial	Insulin
2868141000033110	Humulin 80/20 Injection 100 units/ml, 10 ml vial	Insulin
2868041000033111	Humulin 90/10 Injection 100 units/ml, 10 ml vial	Insulin
724041000033112	Humulin I 100units/ml suspension for injection 10ml vials (Eli Lilly and Company Ltd)	Insulin
1615941000033113	Humulin I 100units/ml suspension for injection 3ml cartridges (Eli Lilly and Company Ltd)	Insulin
719841000033112	Humulin I Cartridges (1.5 Ml) 100 units/ml	Insulin
5911541000033119	Humulin I KwikPen 100units/ml suspension for injection 3ml pre-filled pens (Eli Lilly and Company Ltd)	Insulin
2182441000033113	Humulin I Pen 100units/ml suspension for injection 3ml pre-filled pens (Eli Lilly and Company Ltd)	Insulin
2867841000033117	Humulin L Injection 100 units/ml, 10 ml vial	Insulin
725841000033118	Humulin Lente 100units/ml suspension for injection 10ml vials (Eli Lilly and Company Ltd)	Insulin
719941000033116	Humulin M1 Cartridges (1.5 Ml)	Insulin
1616141000033116	Humulin M1 Cartridges (3 Ml)	Insulin
724141000033111	Humulin M1 Injection	Insulin
1616241000033111	Humulin M2 100units/ml suspension for injection 3ml cartridges (Eli Lilly and Company Ltd)	Insulin
720041000033118	Humulin M2 Cartridges (1.5 Ml)	Insulin
724241000033116	Humulin M2 Injection	Insulin
720141000033119	Humulin M3 (Lilly) Cartridges (1.5 Ml)	Insulin
721541000033117	Humulin M3 (Lilly) Injection 100 units/ml, 10 ml vial	Insulin
725341000033110	Humulin M3 100units/ml suspension for injection 10ml vials (Eli Lilly and Company Ltd)	Insulin
1616341000033118	Humulin M3 100units/ml suspension for injection 3ml cartridges (Eli Lilly and Company Ltd)	Insulin
5910741000033117	Humulin M3 KwikPen 100units/ml suspension for injection 3ml pre-filled pens (Eli Lilly and Company Ltd)	Insulin
3333841000033113	Humulin M3 Pen 100units/ml suspension for injection 3ml pre-filled pens (Eli Lilly and Company Ltd)	Insulin
720241000033114	Humulin M4 Cartridges (1.5 Ml)	Insulin
1616441000033112	Humulin M4 Cartridges (3 Ml)	Insulin
725241000033117	Humulin M4 Injection	Insulin
722841000033112	Humulin M5 100units/ml suspension for injection 10ml vials (Eli Lilly and Company Ltd)	Insulin
721141000033114	Humulin M5 50/50 Cartridges	Insulin

Carr MJ, et al. BMJ Qual Saf 2022; 31:503–514. doi: 10.1136/bmjqs-2021-013613

720441000033110	Humulin M5 Cartridges (1.5 Ml)	Insulin
2867741000033110	Humulin N Injection 100 units/ml, 10 ml vial	Insulin
4423041000033114	Humulin R 500units/ml solution for injection 20ml vials (Imported (United States))	Insulin
2867641000033118	Humulin R Injection 100 units/ml, 10 ml vial	Insulin
12481641000033115	Humulin R KwikPen 500units/ml solution for injection 3ml pre-filled pens (Imported (United States))	Insulin
724341000033114	Humulin S 100units/ml solution for injection 10ml vials (Eli Lilly and Company Ltd)	Insulin
1616041000033115	Humulin S 100units/ml solution for injection 3ml cartridges (Eli Lilly and Company Ltd)	Insulin
720341000033116	Humulin S Cartridges (1.5 Ml) 100 units/ml	Insulin
2867941000033113	Humulin U Injection 100 units/ml, 10 ml vial	Insulin
724541000033119	Humulin Zn 100units/ml suspension for injection 10ml vials (Eli Lilly and Company Ltd)	Insulin
735841000033116	Hypurin Bovine Isophane 100units/ml suspension for injection 10ml vials (Wockhardt UK Ltd)	Insulin
2917041000033118	Hypurin Bovine Isophane 100units/ml suspension for injection 3ml cartridges (Wockhardt UK Ltd)	Insulin
735141000033110	Hypurin Bovine Isophane Injection (cartridges) 100 units/ml	Insulin
735941000033112	Hypurin Bovine Lente 100units/ml suspension for injection 10ml vials (Wockhardt UK Ltd)	Insulin
735241000033115	Hypurin Bovine Lente Injection (cartridges) 100 i.u./ml	Insulin
736041000033119	Hypurin Bovine Neutral 100units/ml solution for injection 10ml vials (Wockhardt UK Ltd)	Insulin
2916941000033119	Hypurin Bovine Neutral 100units/ml solution for injection 3ml cartridges (Wockhardt UK Ltd)	Insulin
735341000033113	Hypurin Bovine Neutral Injection (cartridges) 100 units/ml	Insulin
736141000033115	Hypurin Bovine Protamine Zinc 100units/ml suspension for injection 10ml vials (Wockhardt UK Ltd)	Insulin
2018941000033112	Hypurin Porcine 30/70 Mix 100units/ml suspension for injection 1.5ml cartridges (C P Pharmaceuticals Ltd)	Insulin
2018841000033116	Hypurin Porcine 30/70 Mix 100units/ml suspension for injection 10ml vials (Wockhardt UK Ltd)	Insulin
2917341000033116	Hypurin Porcine 30/70 Mix 100units/ml suspension for injection 3ml cartridges (Wockhardt UK Ltd)	Insulin
734741000033113	Hypurin Porcine Biphasic Isophane Injection	Insulin
734841000033115	Hypurin Porcine Biphasic Isophane Injection (cartridges)	Insulin
734941000033111	Hypurin Porcine Isophane 100units/ml suspension for injection 10ml vials (Wockhardt UK Ltd)	Insulin
2917241000033114	Hypurin Porcine Isophane 100units/ml suspension for injection 3ml cartridges (Wockhardt UK Ltd)	Insulin
735041000033111	Hypurin Porcine Isophane Injection (cartridges) 100 units/ml	Insulin
735441000033119	Hypurin Porcine Neutral 100units/ml solution for injection 10ml vials (Wockhardt UK Ltd)	Insulin
2917141000033119	Hypurin Porcine Neutral 100units/ml solution for injection 3ml cartridges (Wockhardt UK Ltd)	Insulin
735541000033118	Hypurin Porcine Neutral Injection (cartridges) 100 units/ml	Insulin
763241000033114	Initard 50/50 (Nordisk Wellcome) Injection	Insulin
2953241000033110	Insulatard 100units/ml suspension for injection 10ml vials (Novo Nordisk Ltd)	Insulin

2796741000033112	Insulatard FlexPen 100units/ml suspension for injection (Novo Nordisk Ltd)	Insulin
2644641000033113	Insulatard InnoLet 100units/ml suspension for injection 3ml pre-filled pens (Novo Nordisk Ltd)	Insulin
2953141000033115	Insulatard NovoLet 100units/ml suspension for injection (Novo Nordisk Ltd)	Insulin
2953341000033117	Insulatard Penfill 100units/ml suspension for injection 3ml cartridges (Novo Nordisk Ltd)	Insulin
9677041000033116	Insulin aspart 100units/ml solution for injection 1.6ml cartridges	Insulin
1861941000033119	Insulin aspart 100units/ml solution for injection 10ml vials	Insulin
1862041000033113	Insulin aspart 100units/ml solution for injection 3ml cartridges	Insulin
3277741000033118	Insulin aspart 100units/ml solution for injection 3ml pre-filled disposable devices	Insulin
8264341000033118	Insulin degludec 100units/ml solution for injection 3ml cartridges	Insulin
8263941000033117	Insulin degludec 100units/ml solution for injection 3ml pre-filled disposable devices	Insulin
8264041000033115	Insulin degludec 200units/ml solution for injection 3ml pre-filled disposable devices	Insulin
3137041000033115	Insulin detemir 100units/ml solution for injection 3ml cartridges	Insulin
3136941000033116	Insulin detemir 100units/ml solution for injection 3ml pre-filled disposable devices	Insulin
2798841000033115	Insulin glargine 100units/ml solution for injection 10ml vials	Insulin
2798941000033111	Insulin glargine 100units/ml solution for injection 3ml cartridges	Insulin
2799041000033119	Insulin glargine 100units/ml solution for injection 3ml pre-filled disposable devices	Insulin
10494241000033110	Insulin glargine 300units/ml solution for injection 1.5ml pre-filled disposable devices	Insulin
3345641000033114	Insulin glulisine 100units/ml solution for injection 10ml vials	Insulin
3345741000033117	Insulin glulisine 100units/ml solution for injection 3ml cartridges	Insulin
3914241000033112	Insulin glulisine 100units/ml solution for injection 3ml pre-filled disposable devices	Insulin
10673941000033110	Insulin human 100units/ml solution for injection 10ml vials	Insulin
10674041000033112	Insulin human 100units/ml solution for injection 3.15ml cartridges	Insulin
1821641000033119	Insulin isophane bovine 100units/ml suspension for injection 10ml vials	Insulin
1821741000033111	Insulin isophane bovine 100units/ml suspension for injection 3ml cartridges	Insulin
779141000033111	Insulin isophane human 100units/ml suspension for injection 10ml vials	Insulin
1821441000033116	Insulin isophane porcine 100units/ml suspension for injection 10ml vials	Insulin
1821541000033115	Insulin isophane porcine 100units/ml suspension for injection 3ml cartridges	Insulin
757641000033112	Insulin lispro 100units/ml solution for injection 1.5ml cartridges	Insulin
762441000033118	Insulin lispro 100units/ml solution for injection 10ml vials	Insulin
1739941000033110	Insulin lispro 100units/ml solution for injection 3ml cartridges	Insulin
2182241000033112	Insulin lispro 100units/ml solution for injection 3ml pre-filled disposable devices	Insulin
10252641000033111	Insulin lispro 200units/ml solution for injection 3ml pre-filled disposable devices	Insulin
12600741000033116	Insulin lispro Sanofi 100units/ml solution for injection 10ml vials (Sanofi)	Insulin
12600841000033114	Insulin lispro Sanofi 100units/ml solution for injection 3ml cartridges (Sanofi)	Insulin

Carr MJ, *et al. BMJ Qual Saf* 2022; 31:503–514. doi: 10.1136/bmjqs-2021-013613

12600941000033118	Insulin lispro Sanofi 100units/ml solution for injection 3ml pre-filled pens (Sanofi)	Insulin
1119741000033114	Insulin protamine zinc bovine 100units/ml suspension for injection 10ml vials	Insulin
12624241000033111	Insulin soluble porcine 100units/ml solution for injection 10ml vials	Insulin
759841000033115	Insulin Zinc Suspension (Human) Injection 100 units/ml, 10 ml vial	Insulin
763941000033117	Insulin Zinc Suspension Injection 100 units/ml	Insulin
764941000033119	Insulin Zinc Suspension Lente (Evans) Injection 100u/ml	Insulin
2035441000033117	Insuman Basal 100units/ml suspension for injection 3ml cartridges (Sanofi)	Insulin
2266841000033110	Insuman Basal 100units/ml suspension for injection 3ml pre-filled OptiSet pens (Sanofi)	Insulin
6389641000033111	Insuman Basal 100units/ml suspension for injection 3ml pre-filled SoloStar pens (Sanofi)	Insulin
2035241000033118	Insuman Basal 100units/ml suspension for injection 5ml vials (Sanofi)	Insulin
2158541000033117	Insuman Comb 15 100units/ml suspension for injection 3ml cartridges (Sanofi)	Insulin
2267041000033118	Insuman Comb 15 100units/ml suspension for injection 3ml pre-filled OptiSet pens (Sanofi)	Insulin
2158441000033118	Insuman Comb 15 100units/ml suspension for injection 5ml vials (Aventis Pharma)	Insulin
2035641000033115	Insuman Comb 25 100units/ml suspension for injection 3ml cartridges (Sanofi)	Insulin
2267141000033119	Insuman Comb 25 100units/ml suspension for injection 3ml pre-filled OptiSet pens (Sanofi)	Insulin
6011841000033112	Insuman Comb 25 100units/ml suspension for injection 3ml pre-filled SoloStar pens (Sanofi)	Insulin
2035541000033116	Insuman Comb 25 100units/ml suspension for injection 5ml vials (Sanofi)	Insulin
2158741000033113	Insuman Comb 50 100units/ml suspension for injection 3ml cartridges (Sanofi)	Insulin
2267241000033114	Insuman Comb 50 100units/ml suspension for injection 3ml pre-filled OptiSet pens (Sanofi)	Insulin
2158641000033116	Insuman Comb 50 100units/ml suspension for injection 5ml vials (Aventis Pharma)	Insulin
10674141000033111	Insuman Infusat 100units/ml solution for injection 10ml vials (Sanofi)	Insulin
10674341000033114	Insuman Infusat 100units/ml solution for injection 3.15ml cartridges (Sanofi)	Insulin
2034641000033119	Insuman Rapid 100units/ml solution for injection 3ml cartridges (Sanofi)	Insulin
2266941000033119	Insuman Rapid 100units/ml solution for injection 3ml pre-filled OptiSet pens (Sanofi)	Insulin
2034441000033116	Insuman Rapid 100units/ml solution for injection 5ml vials (Aventis Pharma)	Insulin
1620641000033115	Isophane Insulin (Human Pyr) Injection 100 units/ml, 10 ml vial	Insulin
1621141000033118	Isophane Insulin (Human Pyr) Penfill Cartridges (1.5 Ml) 100 units/ml	Insulin
1621241000033113	Isophane Insulin (Human Pyr) Penfill cartridges (3 ml) 100 units/ml	Insulin
1621341000033115	Isophane Insulin (Human Pyr) Preloaded pen 100 units/ml	Insulin
776041000033117	Isophane Insulin (Human, Prb) Cartridges 100 units/ml	Insulin
779641000033118	Isophane Insulin Injection (Evans) Injection 100 units/ml	Insulin
779841000033117	Isophane Insulin Injection 100 units/ml	Insulin
2780741000033111	Lantus 100units/ml solution for injection 10ml vials (Sanofi)	Insulin

2780841000033118	Lantus 100units/ml solution for injection 3ml cartridges (Sanofi)	Insulin
3869341000033114	Lantus 100units/ml solution for injection 3ml OptiClik cartridges (Sanofi)	Insulin
2780941000033114	Lantus 100units/ml solution for injection 3ml pre-filled OptiSet pens (Sanofi)	Insulin
4258541000033111	Lantus 100units/ml solution for injection 3ml pre-filled SoloStar pens (Sanofi)	Insulin
823541000033119	Lentard Mc Injection 100 units/ml, 10 ml vial	Insulin
3137241000033111	Levemir FlexPen 100units/ml solution for injection 3ml pre-filled pens (Novo Nordisk Ltd)	Insulin
4126541000033110	Levemir InnoLet 100units/ml solution for injection 3ml pre-filled pens (Novo Nordisk Ltd)	Insulin
3137141000033116	Levemir Penfill 100units/ml solution for injection 3ml cartridges (Novo Nordisk Ltd)	Insulin
2953441000033111	Mixtard 10 NovoLet 100units/ml suspension for injection (Novo Nordisk Ltd)	Insulin
2953541000033112	Mixtard 10 Penfill 100units/ml suspension for injection 3ml cartridges (Novo Nordisk Ltd)	Insulin
2953641000033113	Mixtard 20 NovoLet 100units/ml suspension for injection (Novo Nordisk Ltd)	Insulin
2953741000033116	Mixtard 20 Penfill 100units/ml suspension for injection 3ml cartridges (Novo Nordisk Ltd)	Insulin
2953941000033118	Mixtard 30 100units/ml suspension for injection 10ml vials (Novo Nordisk Ltd)	Insulin
2644741000033116	Mixtard 30 InnoLet 100units/ml suspension for injection 3ml pre-filled pens (Novo Nordisk Ltd)	Insulin
2953841000033114	Mixtard 30 NovoLet 100units/ml suspension for injection (Novo Nordisk Ltd)	Insulin
2954041000033116	Mixtard 30 Penfill 100units/ml suspension for injection 3ml cartridges (Novo Nordisk Ltd)	Insulin
2954141000033117	Mixtard 40 NovoLet 100units/ml suspension for injection (Novo Nordisk Ltd)	Insulin
2954241000033112	Mixtard 40 Penfill 100units/ml suspension for injection 3ml cartridges (Novo Nordisk Ltd)	Insulin
2954441000033113	Mixtard 50 Injection 100 units/ml, 10 ml vial	Insulin
2954341000033119	Mixtard 50 NovoLet 100units/ml suspension for injection (Novo Nordisk Ltd)	Insulin
2954541000033114	Mixtard 50 Penfill 100units/ml suspension for injection 3ml cartridges (Novo Nordisk Ltd)	Insulin
2954741000033118	Monotard 100units/ml suspension for injection 10ml vials (Novo Nordisk Ltd)	Insulin
926541000033117	Monotard Mc Insulin Zinc Susp Bp Nova Injection 100 units/1 ml	Insulin
962241000033112	Neutral Insulin (Evans) Injection 100 units/ml	Insulin
961141000033113	Neutral Insulin (Human) Injection 100 units/ml	Insulin
961041000033114	Neutral Insulin Injection 100 units/ml	Insulin
2724841000033118	NovoMix 30 FlexPen 100units/ml suspension for injection 3ml pre-filled pens (Novo Nordisk Ltd)	Insulin
2724741000033111	NovoMix 30 Penfill 100units/ml suspension for injection 3ml cartridges (Novo Nordisk Ltd)	Insulin
1862241000033117	NovoRapid 100units/ml solution for injection 10ml vials (Novo Nordisk Ltd)	Insulin
2796641000033115	NovoRapid FlexPen 100units/ml solution for injection 3ml pre-filled pens	Insulin

	(Novo Nordisk Ltd)	
6456441000033119	NovoRapid FlexTouch 100units/ml solution for injection 3ml pre-filled pens (Novo Nordisk Ltd)	Insulin
1862441000033116	NovoRapid Novolet 100units/ml solution for injection (Novo Nordisk Ltd)	Insulin
1862341000033110	NovoRapid Penfill 100units/ml solution for injection 3ml cartridges (Novo Nordisk Ltd)	Insulin
9677141000033117	NovoRapid PumpCart 100units/ml solution for injection 1.6ml cartridges (Novo Nordisk Ltd)	Insulin
1749441000033116	Pork Actrapid 100units/ml solution for injection 10ml vials (Novo Nordisk Ltd)	Insulin
763341000033116	Pork Insulatard 100units/ml suspension for injection 10ml vials (Novo Nordisk Ltd)	Insulin
915641000033117	Pork Mixtard 30 100units/ml suspension for injection 10ml vials (Novo Nordisk Ltd)	Insulin
1508341000033112	Pork Velosulin Injection 100 units/ml	Insulin
1152741000033110	Rapitard Mc (Novo) Injection 100 units/ml	Insulin
12686641000033112	Semglee 100units/ml solution for injection 3ml pre-filled pens (Mylan)	Insulin
1271241000033110	Semitard Mc (Novo) Injection 100 units/ml	Insulin
1419541000033119	Tempulin (Boots) Injection 100 units/ml	Insulin
10494441000033111	Toujeo 300units/ml solution for injection 1.5ml pre-filled SoloStar pens (Sanofi)	Insulin
8264141000033116	Tresiba FlexTouch 100units/ml solution for injection 3ml pre-filled pens (Novo Nordisk Ltd)	Insulin
8264241000033111	Tresiba FlexTouch 200units/ml solution for injection 3ml pre-filled pens (Novo Nordisk Ltd)	Insulin
8264441000033112	Tresiba Penfill 100units/ml solution for injection 3ml cartridges (Novo Nordisk Ltd)	Insulin
2954841000033111	Ultratard 100units/ml suspension for injection 10ml vials (Novo Nordisk Ltd)	Insulin
2954641000033110	Velosulin 100units/ml solution for injection 10ml vials (Novo Nordisk Ltd)	Insulin
1508441000033118	Velosulin Cartridge (Nordisk Wellcome) Injection 100 units/ml	Insulin
6391141000033118	Enyglid 0.5mg tablets (Consilient Health Ltd)	Meglitinide
6391241000033113	Enyglid 1mg tablets (Consilient Health Ltd)	Meglitinide
6391341000033115	Enyglid 2mg tablets (Consilient Health Ltd)	Meglitinide
2288341000033119	Nateglinide 120mg tablets	Meglitinide
2288441000033113	Nateglinide 180mg tablets	Meglitinide
2288241000033112	Nateglinide 60mg tablets	Meglitinide
1660041000033118	NovoNorm 1mg tablets (Novo Nordisk Ltd)	Meglitinide
1660141000033119	NovoNorm 2mg tablets (Novo Nordisk Ltd)	Meglitinide
1659941000033116	NovoNorm 500microgram tablets (Novo Nordisk Ltd)	Meglitinide
3993941000033119	Prandin 0.5mg tablets (Novo Nordisk Ltd)	Meglitinide
3994041000033117	Prandin 1mg tablets (Novo Nordisk Ltd)	Meglitinide
3994141000033118	Prandin 2mg tablets (Novo Nordisk Ltd)	Meglitinide
1672741000033112	Repaglinide 1mg tablets	Meglitinide
1672841000033119	Repaglinide 2mg tablets	Meglitinide
1672941000033110	Repaglinide 500microgram tablets	Meglitinide
2288641000033110	Starlix 120mg tablets (Novartis Pharmaceuticals UK Ltd)	Meglitinide
2288741000033118	Starlix 180mg tablets (Novartis Pharmaceuticals UK Ltd)	Meglitinide
2288541000033114	Starlix 60mg tablets (Novartis Pharmaceuticals UK Ltd)	Meglitinide

8348941000033113	Bolamyn SR 1000mg tablets (Teva UK Ltd)	Metformin
4957741000033118	Bolamyn SR 500mg tablets (Teva UK Ltd)	Metformin
7874941000033110	Diagemet XL 500mg tablets (Genus Pharmaceuticals Ltd)	Metformin
645541000033110	Glucamet Tablets 500 mg	Metformin
645641000033111	Glucamet Tablets 850 mg	Metformin
10598941000033117	Glucient SR 1000mg tablets (Consilient Health Ltd)	Metformin
6391041000033117	Glucient SR 500mg tablets (Consilient Health Ltd)	Metformin
10598841000033113	Glucient SR 750mg tablets (Consilient Health Ltd)	Metformin
5007941000033114	Glucophage 1000mg oral powder sachets (Merck Serono Ltd)	Metformin
5007841000033118	Glucophage 500mg oral powder sachets (Merck Serono Ltd)	Metformin
644941000033117	Glucophage 500mg tablets (Merck Serono Ltd)	Metformin
645041000033117	Glucophage 850mg tablets (Merck Serono Ltd)	Metformin
4945341000033110	Glucophage SR 1000mg tablets (Merck Serono Ltd)	Metformin
3228241000033112	Glucophage SR 500mg tablets (Merck Serono Ltd)	Metformin
4549241000033113	Glucophage SR 750mg tablets (Merck Serono Ltd)	Metformin
5128241000033116	Glucophage Tablets 1000 mg	Metformin
12326741000033119	Meijumet 1000mg modified-release tablets (Medreich Plc)	Metformin
12326541000033110	Meijumet 500mg modified-release tablets (Medreich Plc)	Metformin
12326641000033111	Meijumet 750mg modified-release tablets (Medreich Plc)	Metformin
6279741000033117	Metabet SR 1000mg tablets (Morningside Healthcare Ltd)	Metformin
6029841000033115	Metabet SR 500mg tablets (Morningside Healthcare Ltd)	Metformin
4945241000033117	Metformin 1g modified-release tablets	Metformin
5007741000033111	Metformin 1g oral powder sachets sugar free	Metformin
11781341000033117	Metformin 1g/5ml oral solution sugar free	Metformin
3228141000033117	Metformin 500mg modified-release tablets	Metformin
5007641000033119	Metformin 500mg oral powder sachets sugar free	Metformin
896941000033112	Metformin 500mg tablets	Metformin
5997041000033115	Metformin 500mg/5ml oral solution	Metformin
3982341000033110	Metformin 500mg/5ml oral solution sugar free	Metformin
2620241000033119	Metformin 500mg/5ml oral suspension	Metformin
4549141000033118	Metformin 750mg modified-release tablets	Metformin
897041000033113	Metformin 850mg tablets	Metformin
11781441000033111	Metformin 850mg/5ml oral solution sugar free	Metformin
3890941000033116	Metformin Hydrochloride Sugar free suspension 500 mg/5 ml	Metformin
3982441000033116	Metsol 500mg/5ml oral solution (Kappin Ltd)	Metformin
12664441000033115	Metuxtan SR 500mg tablets (Accord Healthcare Ltd)	Metformin
1017141000033110	Orabet Tablets 500 mg	Metformin
1017241000033115	Orabet Tablets 850 mg	Metformin
9230841000033114	Sukkarto SR 1000mg tablets (Morningside Healthcare Ltd)	Metformin
9230641000033113	Sukkarto SR 500mg tablets (Morningside Healthcare Ltd)	Metformin
12593341000033117	Yaltormin SR 1000mg tablets (Wockhardt UK Ltd)	Metformin
12593141000033115	Yaltormin SR 500mg tablets (Wockhardt UK Ltd)	Metformin
12593241000033110	Yaltormin SR 750mg tablets (Wockhardt UK Ltd)	Metformin
9110341000033112	Canagliflozin 100mg tablets	SGLT2i
9110441000033118	Canagliflozin 300mg tablets	SGLT2i
8199441000033112	Dapagliflozin 10mg tablets	SGLT2i
8199341000033118	Dapagliflozin 5mg tablets	SGLT2i

9336641000033119	Empagliflozin 10mg tablets	SGLT2i
9336841000033118	Empagliflozin 25mg tablets	SGLT2i
8199641000033114	Forxiga 10mg tablets (AstraZeneca UK Ltd)	SGLT2i
8199541000033113	Forxiga 5mg tablets (AstraZeneca UK Ltd)	SGLT2i
9110541000033117	Invokana 100mg tablets (Napp Pharmaceuticals Ltd)	SGLT2i
9110641000033116	Invokana 300mg tablets (Napp Pharmaceuticals Ltd)	SGLT2i
9337141000033114	Jardiance 10mg tablets (Boehringer Ingelheim Ltd)	SGLT2i
9337241000033119	Jardiance 25mg tablets (Boehringer Ingelheim Ltd)	SGLT2i
11898141000033110	Qtern 5mg/10mg tablets (AstraZeneca UK Ltd)	SGLT2i/DPP-4i
11898041000033111	Saxagliptin 5mg / Dapagliflozin 10mg tablets	SGLT2i/DPP-4i
9851541000033114	Canagliflozin 50mg / Metformin 1g tablets	SGLT2i/Metformin
9851641000033110	Canagliflozin 50mg / Metformin 850mg tablets	SGLT2i/Metformin
9106041000033110	Dapagliflozin 5mg / Metformin 1g tablets	SGLT2i/Metformin
9106141000033114	Dapagliflozin 5mg / Metformin 850mg tablets	SGLT2i/Metformin
10614441000033112	Empagliflozin 12.5mg / Metformin 1g tablets	SGLT2i/Metformin
10614341000033118	Empagliflozin 12.5mg / Metformin 850mg tablets	SGLT2i/Metformin
10614241000033111	Empagliflozin 5mg / Metformin 1g tablets	SGLT2i/Metformin
10614141000033116	Empagliflozin 5mg / Metformin 850mg tablets	SGLT2i/Metformin
10614841000033110	Synjardy 12.5mg/1000mg tablets (Boehringer Ingelheim Ltd)	SGLT2i/Metformin
10614741000033117	Synjardy 12.5mg/850mg tablets (Boehringer Ingelheim Ltd)	SGLT2i/Metformin
10614641000033114	Synjardy 5mg/1000mg tablets (Boehringer Ingelheim Ltd)	SGLT2i/Metformin
10614541000033113	Synjardy 5mg/850mg tablets (Boehringer Ingelheim Ltd)	SGLT2i/Metformin
9851841000033111	Vokanamet 50mg/1000mg tablets (Napp Pharmaceuticals Ltd)	SGLT2i/Metformin
9851741000033118	Vokanamet 50mg/850mg tablets (Napp Pharmaceuticals Ltd)	SGLT2i/Metformin
9106241000033119	Xigduo 5mg/1000mg tablets (AstraZeneca UK Ltd)	SGLT2i/Metformin
9106341000033112	Xigduo 5mg/850mg tablets (AstraZeneca UK Ltd)	SGLT2i/Metformin
59541000033116	Amaryl 1mg tablets (Zentiva)	Sulphonylurea
58841000033113	Amaryl 2mg tablets (Zentiva)	Sulphonylurea
59641000033115	Amaryl 3mg tablets (Zentiva)	Sulphonylurea
59741000033112	Amaryl 4mg tablets (Zentiva)	Sulphonylurea
10701641000033116	Bilxona 30mg modified-release tablets (Accord Healthcare Ltd)	Sulphonylurea
10701741000033113	Bilxona 60mg modified-release tablets (Accord Healthcare Ltd)	Sulphonylurea
209341000033112	Calabren Tablets 2.5 mg	Sulphonylurea
218441000033114	Calabren Tablets 5 mg	Sulphonylurea
5378141000033117	Dacadis MR 30mg tablets (Mylan)	Sulphonylurea
406841000033114	Daonil 5mg tablets (Sanofi)	Sulphonylurea
406741000033116	Daonil Tablets 2.5 mg	Sulphonylurea
462041000033119	Diabetamide 2.5mg tablets (Ashbourne Pharmaceuticals Ltd)	Sulphonylurea
462141000033115	Diabetamide 5mg tablets (Ashbourne Pharmaceuticals Ltd)	Sulphonylurea
1602941000033117	Diaglyk 80mg tablets (Ashbourne Pharmaceuticals Ltd)	Sulphonylurea
2289941000033113	Diamicron 30mg MR tablets (Servier Laboratories Ltd)	Sulphonylurea
463541000033118	Diamicron 80mg tablets (Servier Laboratories Ltd)	Sulphonylurea
8027541000033116	Diamicron M/R tablets 60 mg	Sulphonylurea
5815341000033115	Edicil MR 30mg tablets (Teva UK Ltd)	Sulphonylurea
557241000033113	Euglucon 2.5mg tablets (Aventis Pharma)	Sulphonylurea
557341000033115	Euglucon 5mg tablets (Sanofi)	Sulphonylurea
644541000033111	Glibenclamide 2.5mg tablets	Sulphonylurea

644641000033112	Glibenclamide 5mg tablets	Sulphonylurea
5968741000033115	Glibenclamide 5mg/5ml oral solution	Sulphonylurea
5968841000033113	Glibenclamide 5mg/5ml oral suspension	Sulphonylurea
644741000033115	Glibenese 5mg tablets (Pfizer Ltd)	Sulphonylurea
2289441000033115	Gliclazide 30mg modified-release tablets	Sulphonylurea
5709741000033116	Gliclazide 40mg tablets	Sulphonylurea
5869241000033114	Gliclazide 40mg/5ml oral suspension	Sulphonylurea
8298141000033113	Gliclazide 60mg modified-release tablets	Sulphonylurea
646441000033117	Gliclazide 80mg tablets	Sulphonylurea
3953041000033113	Gliclazide 80mg/5ml oral suspension	Sulphonylurea
645941000033116	Glimepiride 1mg tablets	Sulphonylurea
645841000033112	Glimepiride 2mg tablets	Sulphonylurea
646041000033114	Glimepiride 3mg tablets	Sulphonylurea
646141000033113	Glimepiride 4mg tablets	Sulphonylurea
8125241000033119	Glimepiride 6mg/5ml oral suspension	Sulphonylurea
646541000033116	Glipizide 2.5mg tablets	Sulphonylurea
644841000033113	Glipizide 5mg tablets	Sulphonylurea
646241000033118	Glyconon Tablets 500 mg	Sulphonylurea
8298241000033118	Laaglyda MR 60mg tablets (Consilient Health Ltd)	Sulphonylurea
837041000033115	Libanil Tablets 2.5 mg	Sulphonylurea
837141000033116	Libanil Tablets 5 mg	Sulphonylurea
869841000033114	Malix Tablets 2.5 mg	Sulphonylurea
869941000033118	Malix Tablets 5 mg	Sulphonylurea
920141000033112	Minodiab 2.5mg tablets (Pfizer Ltd)	Sulphonylurea
920241000033117	Minodiab 5mg tablets (Pfizer Ltd)	Sulphonylurea
4941241000033119	Nazdol MR 30mg tablets (Consilient Health Ltd)	Sulphonylurea
4522041000033113	Niddaryl 1mg tablets (Dee Pharmaceuticals Ltd)	Sulphonylurea
4522141000033112	Niddaryl 2mg tablets (Dee Pharmaceuticals Ltd)	Sulphonylurea
4522241000033117	Niddaryl 3mg tablets (Dee Pharmaceuticals Ltd)	Sulphonylurea
4522341000033110	Niddaryl 4mg tablets (Dee Pharmaceuticals Ltd)	Sulphonylurea
1153441000033112	Rastinon Tablets 500 mg	Sulphonylurea
1278041000033114	Semi-Daonil 2.5mg tablets (Sanofi)	Sulphonylurea
1450941000033113	Tolbutamide 500mg tablets	Sulphonylurea
10043341000033116	Vamju 30mg modified-release tablets (Advanz Pharma)	Sulphonylurea
10043441000033110	Vamju 60mg modified-release tablets (Advanz Pharma)	Sulphonylurea
6135341000033117	Vitile XL 30mg tablets (Actavis UK Ltd)	Sulphonylurea
5709841000033114	Zicron 40mg tablets (Bristol Laboratories Ltd)	Sulphonylurea
11918941000033116	Zicron PR 30mg tablets (Bristol Laboratories Ltd)	Sulphonylurea
5491941000033110	Amaryl M Tablets 1 mg + 250 mg	Sulphonylurea /Metformin
5492041000033116	Amaryl M Tablets 2 mg + 500 mg	Sulphonylurea /Metformin
2190941000033117	Actos 15mg tablets (Takeda UK Ltd)	TZD
2191041000033110	Actos 30mg tablets (Takeda UK Ltd)	TZD
2996041000033119	Actos 45mg tablets (Takeda UK Ltd)	TZD
2147741000033111	Avandia 4mg tablets (GlaxoSmithKline UK Ltd)	TZD
2147841000033118	Avandia 8mg tablets (GlaxoSmithKline UK Ltd)	TZD

10336941000033112	Diabiom 15mg tablets (Tillomed Laboratories Ltd)	TZD
10337041000033113	Diabiom 30mg tablets (Tillomed Laboratories Ltd)	TZD
10337141000033112	Diabiom 45mg tablets (Tillomed Laboratories Ltd)	TZD
10251341000033112	Glidipion 15mg tablets (Actavis UK Ltd)	TZD
8839341000033116	Glidipion 30mg tablets (Actavis UK Ltd)	TZD
8839441000033110	Glidipion 45mg tablets (Actavis UK Ltd)	TZD
6525941000033110	Glizofar 15mg tablets (Arrow Generics Ltd)	TZD
6526041000033117	Glizofar 30mg tablets (Arrow Generics Ltd)	TZD
6526141000033118	Glizofar 45mg tablets (Arrow Generics Ltd)	TZD
2190741000033115	Pioglitazone 15mg tablets	TZD
2190841000033113	Pioglitazone 30mg tablets	TZD
2995941000033112	Pioglitazone 45mg tablets	TZD
2147541000033115	Rosiglitazone 4mg tablets	TZD
2147641000033119	Rosiglitazone 8mg tablets	TZD
2995141000033110	Avandamet 1mg/500mg tablets (GlaxoSmithKline UK Ltd)	TZD/Metformin
3200741000033112	Avandamet 2mg/1000mg tablets (GlaxoSmithKline UK Ltd)	TZD/Metformin
2995241000033115	Avandamet 2mg/500mg tablets (GlaxoSmithKline UK Ltd)	TZD/Metformin
3200841000033119	Avandamet 4mg/1000mg tablets (GlaxoSmithKline UK Ltd)	TZD/Metformin
3984241000033114	Competact 15mg/850mg tablets (Takeda UK Ltd)	TZD/Metformin
3983941000033115	Pioglitazone 15mg / Metformin 850mg tablets	TZD/Metformin
3191341000033115	Rosiglitazone 1mg / Metformin 500mg tablets	TZD/Metformin
3200541000033116	Rosiglitazone 2mg / Metformin 1g tablets	TZD/Metformin
3191441000033114	Rosiglitazone 2mg / Metformin 500mg tablets	TZD/Metformin
3200641000033115	Rosiglitazone 4mg / Metformin 1g tablets	TZD/Metformin

Carr MJ, et al. BMJ Qual Saf 2022; 31:503–514. doi: 10.1136/bmjqs-2021-013613

Appendix 9. Product codes for prescriptions of antihypertensive medications

Product code	Term	Drug class
13241000033114	Accupro 10mg tablets	ACE inhibitor
13341000033116	Accupro 20mg tablets	ACE inhibitor
11641000033111	Accupro 40mg tablets	ACE inhibitor
13441000033110	Accupro 5mg tablets	ACE inhibitor
10841000033119	Acepril 12.5mg tablets	ACE inhibitor
10941000033110	Acepril 25mg tablets	ACE inhibitor
11041000033117	Acepril 50mg tablets	ACE inhibitor
214141000033113	Capoten 12.5mg tablets	ACE inhibitor
214241000033118	Capoten 25mg tablets	ACE inhibitor
214341000033111	Capoten 50mg tablets	ACE inhibitor
5008141000033111	Captopril 10mg/5ml oral suspension	ACE inhibitor
213441000033119	Captopril 12.5mg tablets	ACE inhibitor
5898741000033114	Captopril 12.5mg/5ml oral solution	ACE inhibitor
5898841000033116	Captopril 12.5mg/5ml oral suspension	ACE inhibitor
5401641000033118	Captopril 15mg/5ml oral suspension	ACE inhibitor
2719841000033113	Captopril 20mg/5ml oral suspension	ACE inhibitor
214441000033117	Captopril 25mg tablets	ACE inhibitor
3012741000033115	Captopril 25mg/5ml oral solution	ACE inhibitor
9108041000033112	Captopril 25mg/5ml oral solution sugar free	ACE inhibitor
9108041000033111	Captopril 25mg/5ml oral solution sugar free	ACE inhibitor
5132041000033112	Captopril 25mg/5ml oral suspension	ACE inhibitor
3940841000033110	Captopril 2mg capsules	ACE inhibitor
3851341000033117	Captopril 3mg/5ml oral solution	ACE inhibitor
5454041000033117	Captopril 4mg capsules	ACE inhibitor
214541000033116	Captopril 50mg tablets	ACE inhibitor
6001041000033115	Captopril 5mg/5ml oral solution	ACE inhibitor
9108141000033110	Captopril 5mg/5ml oral solution sugar free	ACE inhibitor
2656341000033110	Captopril 5mg/5ml oral suspension	ACE inhibitor
5898941000033112	Captopril 6.25mg/5ml oral solution	ACE inhibitor
5899041000033115	Captopril 6.25mg/5ml oral suspension	ACE inhibitor
3333941000033117	Captopril 8mg/5ml oral suspension	ACE inhibitor
5595341000033117	Captopril Oral Suspension Sugar Free 2 mg/ml	ACE inhibitor
210541000033113	Captopril Tablets 1 mg	ACE inhibitor
210741000033117	Captopril Tablets 2 mg	ACE inhibitor
215941000033111	Carace 10mg tablets	ACE inhibitor
216041000033118	Carace 2.5mg tablets	ACE inhibitor
216141000033119	Carace 20mg tablets	ACE inhibitor
216241000033114	Carace 5mg tablets	ACE inhibitor
258141000033110	Cilazapril 1mg tablets	ACE inhibitor
258241000033115	Cilazapril 2.5mg tablets	ACE inhibitor
258441000033119	Cilazapril 500microgram tablets	ACE inhibitor
258641000033117	Cilazapril 5mg tablets	ACE inhibitor
258341000033113	Cilazapril Tablets 250 micrograms	ACE inhibitor
377541000033114	Coversyl 2mg tablets	ACE inhibitor

377641000033110	Coversyl 4mg tablets	ACE inhibitor
2846641000033111	Coversyl 8mg tablets	ACE inhibitor
4454441000033119	Coversyl Arginine 10mg tablets	ACE inhibitor
4454241000033115	Coversyl Arginine 2.5mg tablets	ACE inhibitor
4454341000033113	Coversyl Arginine 5mg tablets	ACE inhibitor
2945841000033112	Ecopace 12.5mg tablets	ACE inhibitor
2945941000033116	Ecopace 25mg tablets	ACE inhibitor
2946041000033114	Ecopace 50mg tablets	ACE inhibitor
1918441000033110	Ednyt Tablets 10 mg	ACE inhibitor
1918241000033114	Ednyt Tablets 2.5 mg	ACE inhibitor
1918541000033111	Ednyt Tablets 20 mg	ACE inhibitor
1918341000033116	Ednyt Tablets 5 mg	ACE inhibitor
6012441000033118	Enalapril 1.25mg/5ml oral solution	ACE inhibitor
6012541000033117	Enalapril 1.25mg/5ml oral suspension	ACE inhibitor
522641000033110	Enalapril 10mg tablets	ACE inhibitor
5967041000033117	Enalapril 10mg/5ml oral solution	ACE inhibitor
5967141000033118	Enalapril 10mg/5ml oral suspension	ACE inhibitor
523541000033115	Enalapril 2.5mg tablets	ACE inhibitor
522741000033118	Enalapril 20mg tablets	ACE inhibitor
522841000033111	Enalapril 5mg tablets	ACE inhibitor
5992541000033111	Enalapril 5mg/5ml oral solution	ACE inhibitor
5992641000033112	Enalapril 5mg/5ml oral suspension	ACE inhibitor
3279241000033117	Enalapril 5mg/5ml oral suspension sugar free	ACE inhibitor
609141000033111	Fosinopril 10mg tablets	ACE inhibitor
609241000033116	Fosinopril 20mg tablets	ACE inhibitor
648341000033112	Gopten 1mg capsules	ACE inhibitor
648441000033118	Gopten 2mg capsules	ACE inhibitor
3014841000033118	Gopten 4mg capsules	ACE inhibitor
648541000033117	Gopten 500microgram capsules	ACE inhibitor
1739741000033112	Imidapril 10mg tablets	ACE inhibitor
2145841000033114	Imidapril 20mg tablets	ACE inhibitor
1739841000033119	Imidapril 5mg tablets	ACE inhibitor
769041000033116	Innovace 10mg tablets	ACE inhibitor
769141000033117	Innovace 2.5mg tablets	ACE inhibitor
769241000033112	Innovace 20mg tablets	ACE inhibitor
769341000033119	Innovace 5mg tablets	ACE inhibitor
1622041000033110	Kaplon 12.5mg tablets	ACE inhibitor
1622141000033114	Kaplon 25mg tablets	ACE inhibitor
1622241000033119	Kaplon 50mg tablets	ACE inhibitor
837841000033110	Lisinopril 10mg tablets	ACE inhibitor
837941000033119	Lisinopril 2.5mg tablets	ACE inhibitor
5971341000033117	Lisinopril 2.5mg/5ml oral solution	ACE inhibitor
5971441000033111	Lisinopril 2.5mg/5ml oral suspension	ACE inhibitor
838041000033116	Lisinopril 20mg tablets	ACE inhibitor
6044641000033119	Lisinopril 20mg/5ml oral solution	ACE inhibitor
6044541000033115	Lisinopril 20mg/5ml oral suspension	ACE inhibitor
5568741000033113	Lisinopril 40mg/5ml oral suspension	ACE inhibitor

838141000033117	Lisinopril 5mg tablets	ACE inhibitor
5890241000033117	Lisinopril 5mg/5ml oral solution	ACE inhibitor
10645041000033114	Lisinopril 5mg/5ml oral solution sugar free	ACE inhibitor
3152141000033115	Lisinopril 5mg/5ml oral suspension	ACE inhibitor
5971541000033112	Lisinopril 7.5mg/5ml oral solution	ACE inhibitor
5971641000033113	Lisinopril 7.5mg/5ml oral suspension	ACE inhibitor
3159941000033114	Lopace 10mg capsules	ACE inhibitor
3159741000033111	Lopace 2.5mg capsules	ACE inhibitor
3159841000033118	Lopace 5mg capsules	ACE inhibitor
938041000033113	Moexipril 15mg tablets	ACE inhibitor
938141000033112	Moexipril 7.5mg tablets	ACE inhibitor
8961341000033119	Noyada 25mg/5ml oral solution	ACE inhibitor
8961241000033112	Noyada 5mg/5ml oral solution	ACE inhibitor
998541000033110	Odrik 1mg capsules	ACE inhibitor
998641000033111	Odrik 2mg capsules	ACE inhibitor
998741000033119	Odrik 500microgram capsules	ACE inhibitor
1065941000033115	Perdix 15mg tablets	ACE inhibitor
1066041000033113	Perdix 7.5mg tablets	ACE inhibitor
5997541000033113	Perindopril Oral solution 8 mg/5 ml	ACE inhibitor
5888241000033116	Perindopril Oral suspension 4 mg/5 ml	ACE inhibitor
4153041000033114	Perindopril Oral suspension 8 mg/5 ml	ACE inhibitor
4454141000033110	Perindopril arginine 10mg tablets	ACE inhibitor
4453941000033114	Perindopril arginine 2.5mg tablets	ACE inhibitor
4454041000033111	Perindopril arginine 5mg tablets	ACE inhibitor
1067841000033115	Perindopril erbumine 2mg tablets	ACE inhibitor
1067941000033111	Perindopril erbumine 4mg tablets	ACE inhibitor
8869441000033119	Perindopril erbumine 4mg/5ml oral solution	ACE inhibitor
8276041000033117	Perindopril erbumine 4mg/5ml oral suspension	ACE inhibitor
2846741000033119	Perindopril erbumine 8mg tablets	ACE inhibitor
8275941000033110	Perindopril erbumine 8mg/5ml oral solution	ACE inhibitor
8276141000033118	Perindopril erbumine 8mg/5ml oral suspension	ACE inhibitor
8263541000033111	Perindopril tosilate 10mg tablets	ACE inhibitor
8263341000033116	Perindopril tosilate 2.5mg tablets	ACE inhibitor
8263441000033110	Perindopril tosilate 5mg tablets	ACE inhibitor
1918841000033113	Pralenal 10 tablets	ACE inhibitor
1918641000033112	Pralenal 2.5 tablets	ACE inhibitor
1918941000033117	Pralenal 20 tablets	ACE inhibitor
1918741000033115	Pralenal 5 tablets	ACE inhibitor
1149341000033118	Quinapril 10mg tablets	ACE inhibitor
1149441000033112	Quinapril 20mg tablets	ACE inhibitor
1149541000033113	Quinapril 40mg tablets	ACE inhibitor
1149241000033111	Quinapril 5mg tablets	ACE inhibitor
3283241000033113	Quinil 10mg tablets	ACE inhibitor
3283341000033115	Quinil 20mg tablets	ACE inhibitor
3283441000033114	Quinil 40mg tablets	ACE inhibitor
3283141000033118	Quinil 5mg tablets	ACE inhibitor
1151341000033117	Ramipril 1.25mg capsules	ACE inhibitor

2989541000033115	Ramipril 1.25mg tablets	ACE inhibitor
5998241000033112	Ramipril 1.25mg/5ml oral solution	ACE inhibitor
4152241000033113	Ramipril 1.25mg/5ml oral suspension	ACE inhibitor
1769841000033111	Ramipril 10mg capsules	ACE inhibitor
2989841000033118	Ramipril 10mg tablets	ACE inhibitor
5887841000033118	Ramipril 10mg/5ml oral solution	ACE inhibitor
5887941000033114	Ramipril 10mg/5ml oral suspension	ACE inhibitor
1151441000033111	Ramipril 2.5mg capsules	ACE inhibitor
2989641000033119	Ramipril 2.5mg tablets	ACE inhibitor
5890541000033115	Ramipril 2.5mg/5ml oral solution	ACE inhibitor
6517041000033110	Ramipril 2.5mg/5ml oral solution sugar free	ACE inhibitor
4805941000033114	Ramipril 2.5mg/5ml oral suspension	ACE inhibitor
1151541000033112	Ramipril 5mg capsules	ACE inhibitor
2989741000033111	Ramipril 5mg tablets	ACE inhibitor
5890341000033110	Ramipril 5mg/5ml oral solution	ACE inhibitor
2883341000033118	Ramipril 5mg/5ml oral suspension	ACE inhibitor
3890041000033117	Ramipril Powder	ACE inhibitor
2724541000033115	Ramipril Titration pack	ACE inhibitor
1387341000033114	Staril 10mg tablets	ACE inhibitor
1387441000033115	Staril 20mg tablets	ACE inhibitor
1753841000033118	Tanatril 10mg tablets	ACE inhibitor
2145741000033116	Tanatril 20mg tablets	ACE inhibitor
1753941000033114	Tanatril 5mg tablets	ACE inhibitor
2928341000033114	Tensopril 12.5mg tablets	ACE inhibitor
2928441000033115	Tensopril 25mg tablets	ACE inhibitor
2928541000033119	Tensopril 50mg tablets	ACE inhibitor
1453941000033115	Trandolapril 1mg capsules	ACE inhibitor
1454041000033118	Trandolapril 2mg capsules	ACE inhibitor
3014741000033111	Trandolapril 4mg capsules	ACE inhibitor
1454141000033119	Trandolapril 500microgram capsules	ACE inhibitor
1455541000033110	Tritace 1.25mg capsules	ACE inhibitor
2989941000033114	Tritace 1.25mg tablets	ACE inhibitor
1769941000033115	Tritace 10mg capsules	ACE inhibitor
2990241000033112	Tritace 10mg tablets	ACE inhibitor
1455641000033111	Tritace 2.5mg capsules	ACE inhibitor
2990041000033116	Tritace 2.5mg tablets	ACE inhibitor
1455741000033119	Tritace 5mg capsules	ACE inhibitor
2990141000033117	Tritace 5mg tablets	ACE inhibitor
4521441000033112	Tritace Tablet Titration Pack	ACE inhibitor
2724641000033119	Tritace titration pack capsules	ACE inhibitor
12090641000033120	Tritace titration pack tablets	ACE inhibitor
12090641000033119	Tritace titration pack tablets	ACE inhibitor
1504641000033112	Vascace 1mg tablets	ACE inhibitor
1504741000033115	Vascace 2.5mg tablets	ACE inhibitor
1504941000033117	Vascace 500microgram tablets	ACE inhibitor
1503441000033113	Vascace 5mg tablets	ACE inhibitor
1504841000033113	Vascace Tablets 250 micrograms	ACE inhibitor

1551941000033114	Zestril 10mg tablets	ACE inhibitor
1552041000033115	Zestril 2.5mg tablets	ACE inhibitor
1552141000033116	Zestril 20mg tablets	ACE inhibitor
1552241000033111	Zestril 5mg tablets	ACE inhibitor
2077541000033111	Alfuzosin 10mg modified-release tablets	Alpha blocker
38141000033118	Alfuzosin 2.5mg tablets	Alpha blocker
32541000033112	Alfuzosin 5mg modified-release tablets	Alpha blocker
4504241000033115	Alphacard MR 400microgram capsules	Alpha blocker
37641000033110	Alphavase 0.5 Tablets 500 micrograms	Alpha blocker
37741000033118	Alphavase 1 tablets	Alpha blocker
37841000033111	Alphavase 2 tablets	Alpha blocker
37941000033115	Alphavase 5 tablets	Alpha blocker
116741000033118	Baratol 25mg tablets	Alpha blocker
116841000033111	Baratol Tablets 50 mg	Alpha blocker
3893741000033111	Bazetham MR 400microgram capsules	Alpha blocker
11799841000033112	Benph 2mg tablets	Alpha blocker
11807841000033120	Benph 5mg tablets	Alpha blocker
11807841000033119	Benph 5mg tablets	Alpha blocker
3980441000033115	Besavar XL 10mg tablets	Alpha blocker
4936841000033112	Cardozin XL 4mg tablets	Alpha blocker
216741000033115	Cardura 1mg tablets	Alpha blocker
216841000033113	Cardura 2mg tablets	Alpha blocker
216941000033117	Cardura Tablets 4 mg	Alpha blocker
2274041000033118	Cardura XL 4mg tablets	Alpha blocker
2274141000033119	Cardura XL 8mg tablets	Alpha blocker
2760041000033110	Cascor 2mg tablets	Alpha blocker
2760141000033114	Cascor 4mg tablets	Alpha blocker
5403641000033117	Colixil XL 4mg tablets	Alpha blocker
4023841000033113	Contiflo XL 400microgram capsules	Alpha blocker
6065741000033115	Cositam XL 400microgram tablets	Alpha blocker
438441000033119	Dibenyline 10mg capsules	Alpha blocker
450041000033112	Dibenyline Injection 50 mg/ml, 2 ml ampoule	Alpha blocker
5330641000033110	Diffundox XL 400microgram capsules	Alpha blocker
480541000033110	Doralese Tiltab 20mg tablets	Alpha blocker
2957941000033110	Doxadura 1mg tablets	Alpha blocker
2958041000033113	Doxadura 2mg tablets	Alpha blocker
2958141000033112	Doxadura 4mg tablets	Alpha blocker
4063741000033112	Doxadura XL 4mg tablets	Alpha blocker
480641000033111	Doxazosin 1mg tablets	Alpha blocker
9173941000033112	Doxazosin 1mg/5ml oral solution	Alpha blocker
9173941000033111	Doxazosin 1mg/5ml oral solution	Alpha blocker
4432941000033119	Doxazosin 1mg/5ml oral suspension	Alpha blocker
480741000033119	Doxazosin 2mg tablets	Alpha blocker
2273841000033111	Doxazosin 4mg modified-release tablets	Alpha blocker
480841000033112	Doxazosin 4mg tablets	Alpha blocker
8870641000033119	Doxazosin 4mg/5ml oral solution	Alpha blocker
5138341000033111	Doxazosin 4mg/5ml oral suspension	Alpha blocker

2273941000033115	Doxazosin 8mg modified-release tablets	Alpha blocker
12682141000033116	Doxazosin 8mg tablets	Alpha blocker
12682141000033115	Doxazosin 8mg tablets	Alpha blocker
4502341000033119	Doxazosin Oral suspension 1 mg/5 ml	Alpha blocker
4575941000033115	Doxzogen XL 4mg tablets	Alpha blocker
6528741000033118	Faramsil 400microgram modified-release tablets	Alpha blocker
8271541000033118	Flectone XL 400microgram tablets	Alpha blocker
588741000033112	Flomax MR 400microgram capsules	Alpha blocker
3309641000033116	Flomaxtra XL 400microgram tablets	Alpha blocker
5816041000033114	Fuzatal XL 10mg tablets	Alpha blocker
5566141000033113	Galebon 400microgram modified-release capsules	Alpha blocker
743641000033117	Hypovase 1mg tablets	Alpha blocker
743741000033114	Hypovase 2mg tablets	Alpha blocker
743541000033118	Hypovase 500microgram tablets	Alpha blocker
745941000033111	Hypovase Starter Pack Tablets	Alpha blocker
743841000033116	Hypovase Tablets 5 mg	Alpha blocker
745641000033116	Hytrin 10mg tablets	Alpha blocker
745741000033113	Hytrin 2mg tablets	Alpha blocker
745841000033115	Hytrin 5mg tablets	Alpha blocker
741041000033112	Hytrin BPH tablets starter pack	Alpha blocker
740941000033119	Hytrin Bph Starter pack	Alpha blocker
743941000033112	Hytrin Bph Tablets 10 mg	Alpha blocker
744041000033114	Hytrin Bph Tablets 2 mg	Alpha blocker
744141000033113	Hytrin Bph Tablets 5 mg	Alpha blocker
741141000033111	Hytrin tablets starter pack	Alpha blocker
768541000033119	Indoramin 20mg tablets	Alpha blocker
769841000033111	Indoramin 25mg tablets	Alpha blocker
769941000033115	Indoramin Hydrochloride Tablets 50 mg	Alpha blocker
768441000033115	Indoramin Tablets 25 mg	Alpha blocker
1619941000033111	Invicorp 1 solution	Alpha blocker
1620041000033114	Invicorp 2 solution	Alpha blocker
10617741000033112	Invicorp 25micrograms/2mg/0.35ml solution	Alpha blocker
7877741000033118	Kelanu XL 10mg tablets	Alpha blocker
5809641000033111	Larbex XL 4mg tablets	Alpha blocker
6138841000033117	Losinate MR 400microgram capsules	Alpha blocker
4936541000033110	Maxtron 400microgram modified-release capsules	Alpha blocker
4011341000033111	Morvesin XL 400microgram capsules	Alpha blocker
2946441000033117	Omnic MR 400microgram capsules	Alpha blocker
4288341000033111	Oxandosin XL 4mg tablets	Alpha blocker
5811641000033114	Pamsvax XL 400microgram capsules	Alpha blocker
5817041000033111	Petyme 400microgram MR capsules	Alpha blocker
1074841000033115	Phenoxybenzamine 100mg/2ml solution	Alpha blocker
1070341000033119	Phenoxybenzamine 10mg capsules	Alpha blocker
2757941000033118	Phenoxybenzamine 25mg/5ml oral suspension	Alpha blocker
3940141000033116	Phenoxybenzamine Hydrochloride Capsules 8 mg	Alpha blocker
3108541000033116	Phentolamine 10mg/1ml solution	Alpha blocker
10617641000033120	Phentolamine 2mg/0.35ml / Aviptadil	Alpha blocker

	25micrograms/0.35ml solution	
1074241000033119	Phentolamine Mesylate Injection 10 mg/ml, 1 ml ampoule	Alpha blocker
5836341000033119	Pinexel PR 400microgram capsules	Alpha blocker
1131441000033112	Prazosin 1mg tablets	Alpha blocker
1131541000033113	Prazosin 2mg tablets	Alpha blocker
1131341000033118	Prazosin 500microgram tablets	Alpha blocker
11567441000033119	Prazosin 500micrograms/5ml oral solution	Alpha blocker
11567441000033120	Prazosin 500micrograms/5ml oral solution	Alpha blocker
1131641000033114	Prazosin 5mg tablets	Alpha blocker
1128241000033117	Prazosin Hydrochloride Starter pack	Alpha blocker
1128141000033112	Prazosin Hydrochloride Starter pack	Alpha blocker
5814441000033110	Prosurin XL 400microgram capsules	Alpha blocker
6036141000033119	Raporsin XL 4mg tablets	Alpha blocker
1180941000033115	Rogitine 10mg/1ml solution	Alpha blocker
4021141000033118	Slocinx XL 4mg tablets	Alpha blocker
8960641000033119	Solifenacin 6mg / Tamsulosin 400microgram modified-release tablets	Alpha blocker
3892641000033110	Stronazon 400microgram MR capsules	Alpha blocker
3891841000033119	Tabphyn MR 400microgram capsules	Alpha blocker
1406241000033110	Tamsulosin 400microgram modified-release capsules	Alpha blocker
3342941000033112	Tamsulosin 400microgram modified-release tablets	Alpha blocker
9201141000033116	Tamsulosin 400micrograms/5ml oral solution	Alpha blocker
9201141000033115	Tamsulosin 400micrograms/5ml oral solution	Alpha blocker
11524441000033118	Tamsulosin 400micrograms/5ml oral suspension	Alpha blocker
5844541000033118	Tamurex 400microgram modified-release capsules	Alpha blocker
1429041000033118	Terazosin 10mg tablets	Alpha blocker
1425341000033119	Terazosin 2mg tablets	Alpha blocker
1423641000033114	Terazosin 2mg tablets and Terazosin 1mg tablets	Alpha blocker
1429141000033119	Terazosin 5mg tablets	Alpha blocker
1423841000033110	Terazosin Hydrochloride Starter pack	Alpha blocker
1423741000033117	Terazosin Hydrochloride Starter pack 7 x 1 mg, 7 x 2 mg	Alpha blocker
5851641000033117	Vasran XL 10mg tablets	Alpha blocker
1543041000033110	Xatral 2.5mg tablets	Alpha blocker
1543341000033112	Xatral SR 5mg tablets	Alpha blocker
2077641000033112	Xatral XL 10mg tablets	Alpha blocker
12477141000033113	Zochek 10mg modified-release tablets	Alpha blocker
12477141000033112	Zochek 10mg modified-release tablets	Alpha blocker
3992841000033117	Zufal XL 10mg tablets	Alpha blocker
59141000033113	Amias 16mg tablets	Angiotensin receptor blocker
59241000033118	Amias 2mg tablets	Angiotensin receptor blocker
3227341000033118	Amias 32mg tablets	Angiotensin receptor blocker
59341000033111	Amias 4mg tablets	Angiotensin receptor blocker
59441000033117	Amias 8mg tablets	Angiotensin receptor

		blocker
75941000033114	Aprovel 150mg tablets	Angiotensin receptor blocker
76041000033116	Aprovel 300mg tablets	Angiotensin receptor blocker
76141000033117	Aprovel 75mg tablets	Angiotensin receptor blocker
7754841000033119	Azilsartan medoxomil 20mg tablets	Angiotensin receptor blocker
7754941000033110	Azilsartan medoxomil 40mg tablets	Angiotensin receptor blocker
7755041000033110	Azilsartan medoxomil 80mg tablets	Angiotensin receptor blocker
211541000033119	Candesartan 16mg tablets	Angiotensin receptor blocker
211641000033118	Candesartan 2mg tablets	Angiotensin receptor blocker
3227241000033111	Candesartan 32mg tablets	Angiotensin receptor blocker
211741000033110	Candesartan 4mg tablets	Angiotensin receptor blocker
211841000033117	Candesartan 8mg tablets	Angiotensin receptor blocker
2720241000033110	Cozaar 100mg tablets	Angiotensin receptor blocker
4957641000033110	Cozaar 12.5mg tablets	Angiotensin receptor blocker
5150041000033110	Cozaar 2.5mg/ml oral suspension	Angiotensin receptor blocker
370441000033112	Cozaar 25mg tablets	Angiotensin receptor blocker
370541000033113	Cozaar 50mg tablets	Angiotensin receptor blocker
437641000033118	Diovan 160mg capsules	Angiotensin receptor blocker
4425041000033113	Diovan 320mg tablets	Angiotensin receptor blocker
6528541000033114	Diovan 3mg/1ml oral solution	Angiotensin receptor blocker
437741000033110	Diovan 40mg capsules	Angiotensin receptor blocker
3201441000033114	Diovan 40mg tablets	Angiotensin receptor blocker
437841000033117	Diovan 80mg capsules	Angiotensin receptor blocker
7755141000033114	Edarbi 20mg tablets	Angiotensin receptor blocker
7755241000033119	Edarbi 40mg tablets	Angiotensin receptor blocker
7755341000033112	Edarbi 80mg tablets	Angiotensin receptor blocker
10943941000033112	Entresto 24mg/26mg tablets	Angiotensin receptor

		blocker
10944041000033114	Entresto 49mg/51mg tablets	Angiotensin receptor blocker
10944141000033113	Entresto 97mg/103mg tablets	Angiotensin receptor blocker
10944141000033112	Entresto 97mg/103mg tablets	Angiotensin receptor blocker
2036341000033115	Eprosartan 300mg tablets	Angiotensin receptor blocker
2036441000033114	Eprosartan 400mg tablets	Angiotensin receptor blocker
2036541000033110	Eprosartan 600mg tablets	Angiotensin receptor blocker
8554241000033111	Ifirmasta 150mg tablets	Angiotensin receptor blocker
8554341000033118	Ifirmasta 300mg tablets	Angiotensin receptor blocker
8554141000033116	Ifirmasta 75mg tablets	Angiotensin receptor blocker
775441000033117	Irbesartan 150mg tablets	Angiotensin receptor blocker
5970641000033117	Irbesartan 150mg/5ml oral suspension	Angiotensin receptor blocker
6167841000033112	Irbesartan 20mg oral powder sachets	Angiotensin receptor blocker
775541000033116	Irbesartan 300mg tablets	Angiotensin receptor blocker
4954341000033111	Irbesartan 300mg/5ml oral suspension	Angiotensin receptor blocker
6423941000033116	Irbesartan 30mg oral powder sachets	Angiotensin receptor blocker
6435741000033117	Irbesartan 37.5mg oral powder sachets	Angiotensin receptor blocker
775641000033115	Irbesartan 75mg tablets	Angiotensin receptor blocker
2720141000033115	Losartan 100mg tablets	Angiotensin receptor blocker
5971741000033116	Losartan 100mg/5ml oral solution	Angiotensin receptor blocker
5971841000033114	Losartan 100mg/5ml oral suspension	Angiotensin receptor blocker
4957541000033114	Losartan 12.5mg tablets	Angiotensin receptor blocker
5149941000033118	Losartan 2.5mg/ml oral suspension sugar free	Angiotensin receptor blocker
851841000033117	Losartan 25mg tablets	Angiotensin receptor blocker
851941000033113	Losartan 50mg tablets	Angiotensin receptor blocker
5971941000033118	Losartan 50mg/5ml oral solution	Angiotensin receptor blocker
5972041000033112	Losartan 50mg/5ml oral suspension	Angiotensin receptor

		blocker
2272741000033111	Micardis 20mg tablets	Angiotensin receptor blocker
1849741000033114	Micardis 40mg tablets	Angiotensin receptor blocker
1849841000033116	Micardis 80mg tablets	Angiotensin receptor blocker
2944541000033111	Olmesartan medoxomil 10mg tablets	Angiotensin receptor blocker
4273241000033118	Olmesartan medoxomil 10mg/5ml oral suspension	Angiotensin receptor blocker
2944641000033112	Olmesartan medoxomil 20mg tablets	Angiotensin receptor blocker
2944741000033115	Olmesartan medoxomil 40mg tablets	Angiotensin receptor blocker
2944841000033113	Olmetec 10mg tablets	Angiotensin receptor blocker
2944941000033117	Olmetec 20mg tablets	Angiotensin receptor blocker
2945041000033117	Olmetec 40mg tablets	Angiotensin receptor blocker
8194041000033119	Sabervel 150mg tablets	Angiotensin receptor blocker
8194141000033115	Sabervel 300mg tablets	Angiotensin receptor blocker
8193941000033116	Sabervel 75mg tablets	Angiotensin receptor blocker
10943641000033117	Sacubitril 24mg / Valsartan 26mg tablets	Angiotensin receptor blocker
10943641000033116	Sacubitril 24mg / Valsartan 26mg tablets	Angiotensin receptor blocker
10943741000033114	Sacubitril 49mg / Valsartan 51mg tablets	Angiotensin receptor blocker
10943841000033116	Sacubitril 97mg / Valsartan 103mg tablets	Angiotensin receptor blocker
2272641000033119	Telmisartan 20mg tablets	Angiotensin receptor blocker
1849541000033118	Telmisartan 40mg tablets	Angiotensin receptor blocker
1849641000033117	Telmisartan 80mg tablets	Angiotensin receptor blocker
2040841000033113	Teveten 300mg tablets	Angiotensin receptor blocker
2040941000033117	Teveten 400mg tablets	Angiotensin receptor blocker
2041041000033110	Teveten 600mg tablets	Angiotensin receptor blocker
8030141000033111	Teveten Plus Tablets 600 mg/12.5 mg	Angiotensin receptor blocker
9209341000033116	Tolura 20mg tablets	Angiotensin receptor blocker
9209441000033110	Tolura 40mg tablets	Angiotensin receptor

		blocker
9209541000033111	Tolura 80mg tablets	Angiotensin receptor blocker
9209541000033112	Tolura 80mg tablets	Angiotensin receptor blocker
1498241000033111	Valsartan 160mg capsules	Angiotensin receptor blocker
6515541000033114	Valsartan 160mg tablets	Angiotensin receptor blocker
4424941000033113	Valsartan 320mg tablets	Angiotensin receptor blocker
6528441000033113	Valsartan 3mg/ml oral solution	Angiotensin receptor blocker
1498341000033118	Valsartan 40mg capsules	Angiotensin receptor blocker
3201341000033115	Valsartan 40mg tablets	Angiotensin receptor blocker
1498441000033112	Valsartan 80mg capsules	Angiotensin receptor blocker
6515441000033113	Valsartan 80mg tablets	Angiotensin receptor blocker
3041000033115	Acebutolol 100mg capsules	Beta blocker
3141000033116	Acebutolol 200mg capsules	Beta blocker
10741000033112	Acebutolol 400mg tablets	Beta blocker
6041000033113	Acebutolol Injection 5 mg/1 ml	Beta blocker
70941000033117	Antipressan 100mg tablets	Beta blocker
69441000033112	Antipressan 25mg tablets	Beta blocker
71041000033110	Antipressan 50mg tablets	Beta blocker
75341000033110	Apsolox Tablets 160 mg	Beta blocker
75441000033116	Apsolox Tablets 20 mg	Beta blocker
75541000033115	Apsolox Tablets 40 mg	Beta blocker
75641000033119	Apsolox Tablets 80 mg	Beta blocker
1572241000033113	Atenamin Tablets 100 mg	Beta blocker
1572341000033115	Atenamin Tablets 25 mg	Beta blocker
1572441000033114	Atenamin Tablets 50 mg	Beta blocker
90541000033117	Atenix 100 tablets	Beta blocker
91841000033113	Atenix 25 tablets	Beta blocker
92541000033118	Atenix 50 tablets	Beta blocker
91041000033118	Atenolol 100mg tablets	Beta blocker
90641000033116	Atenolol 25mg tablets	Beta blocker
90441000033118	Atenolol 25mg/5ml oral solution sugar free	Beta blocker
91141000033119	Atenolol 50mg tablets	Beta blocker
89541000033111	Atenolol 5mg/10ml solution	Beta blocker
12682341000033117	Bedranol 10mg tablets	Beta blocker
12682341000033116	Bedranol 10mg tablets	Beta blocker
12682741000033116	Bedranol 160mg tablets	Beta blocker
12682441000033112	Bedranol 40mg tablets	Beta blocker
12682441000033111	Bedranol 40mg tablets	Beta blocker
12682641000033112	Bedranol 80mg tablets	Beta blocker

12682641000033113	Bedranol 80mg tablets	Beta blocker
124241000033112	Bedranol SR 160mg capsules	Beta blocker
2711841000033114	Bedranol SR 80mg capsules	Beta blocker
143941000033116	Beta-Cardone 200mg tablets	Beta blocker
144041000033119	Beta-Cardone 40mg tablets	Beta blocker
144141000033115	Beta-Cardone 80mg tablets	Beta blocker
133141000033117	Beta-Prograne 160mg modified-release capsules	Beta blocker
144341000033117	Betaloc 100mg tablets	Beta blocker
144441000033111	Betaloc 50mg tablets	Beta blocker
131141000033116	Betaloc I.V. 5mg/5ml solution	Beta blocker
144541000033112	Betaloc-SA 200mg tablets	Beta blocker
145541000033111	Betaxolol 20mg tablets	Beta blocker
144741000033116	Betim 10mg tablets	Beta blocker
2638141000033114	Bipranix 10mg tablets	Beta blocker
2638041000033110	Bipranix 5mg tablets	Beta blocker
1951641000033111	Bisoprolol 1.25mg tablets	Beta blocker
5898041000033111	Bisoprolol 1.25mg/5ml oral solution	Beta blocker
5898141000033110	Bisoprolol 1.25mg/5ml oral suspension	Beta blocker
10025241000033112	Bisoprolol 10mg / Aspirin 100mg capsules	Beta blocker
10025141000033116	Bisoprolol 10mg / Aspirin 75mg capsules	Beta blocker
10025141000033117	Bisoprolol 10mg / Aspirin 75mg capsules	Beta blocker
514741000033117	Bisoprolol 10mg tablets	Beta blocker
8536441000033115	Bisoprolol 10mg/5ml oral suspension	Beta blocker
1951741000033119	Bisoprolol 2.5mg tablets	Beta blocker
5991041000033111	Bisoprolol 2.5mg/5ml oral solution	Beta blocker
4507941000033117	Bisoprolol 2.5mg/5ml oral suspension	Beta blocker
1951841000033112	Bisoprolol 3.75mg tablets	Beta blocker
10025041000033116	Bisoprolol 5mg / Aspirin 100mg capsules	Beta blocker
10024941000033116	Bisoprolol 5mg / Aspirin 75mg capsules	Beta blocker
151341000033117	Bisoprolol 5mg tablets	Beta blocker
5898241000033115	Bisoprolol 5mg/5ml oral solution	Beta blocker
5898341000033113	Bisoprolol 5mg/5ml oral suspension	Beta blocker
5898441000033119	Bisoprolol 625micrograms/5ml oral solution	Beta blocker
5898541000033118	Bisoprolol 625micrograms/5ml oral suspension	Beta blocker
1951941000033116	Bisoprolol 7.5mg tablets	Beta blocker
4267141000033111	Bisoprolol 7.5mg/5ml oral suspension	Beta blocker
151441000033111	Bisoprolol Fumarate Tablets 10 mg	Beta blocker
153441000033112	Blocadren Tablets 10 mg	Beta blocker
158041000033114	Brevibloc Concentrate 2.5g/10ml solution	Beta blocker
157941000033111	Brevibloc Injection 10 mg/ml	Beta blocker
4202341000033110	Brevibloc Injection 10 mg/ml, 10 ml vial	Beta blocker
5655241000033110	Brevibloc Premixed 100mg/10ml solution	Beta blocker
5656241000033115	Brevibloc Premixed 2.5g/250ml infusion bags	Beta blocker
1952241000033119	Cardicor 1.25mg tablets	Beta blocker
1952741000033113	Cardicor 10mg tablets	Beta blocker
1952341000033112	Cardicor 2.5mg tablets	Beta blocker
1952441000033118	Cardicor 3.75mg tablets	Beta blocker

1952541000033117	Cardicor 5mg tablets	Beta blocker
1952641000033116	Cardicor 7.5mg tablets	Beta blocker
208841000033114	Carteolol Hydrochloride Tablets 10 mg	Beta blocker
218141000033118	Cartrol Tablets 10 mg	Beta blocker
209941000033111	Carvedilol 12.5mg tablets	Beta blocker
210041000033115	Carvedilol 25mg tablets	Beta blocker
1581241000033110	Carvedilol 3.125mg tablets	Beta blocker
5288741000033116	Carvedilol 5mg/5ml oral suspension	Beta blocker
1581341000033117	Carvedilol 6.25mg tablets	Beta blocker
210141000033116	Carvedilol Tablets 50 mg	Beta blocker
236141000033110	Celectol 200mg tablets	Beta blocker
233241000033114	Celectol 400mg tablets	Beta blocker
236041000033111	Celiprolol 200mg tablets	Beta blocker
233341000033116	Celiprolol 400mg tablets	Beta blocker
3984541000033111	Congescor 1.25mg tablets	Beta blocker
3984641000033112	Congescor 2.5mg tablets	Beta blocker
375241000033119	Corgard 40mg tablets	Beta blocker
375341000033112	Corgard 80mg tablets	Beta blocker
514141000033116	Emcor 10mg tablets	Beta blocker
513941000033117	Emcor LS 5mg tablets	Beta blocker
4202241000033117	Esmolol 100mg/10ml solution	Beta blocker
12206941000033120	Esmolol 2.5g powder for solution	Beta blocker
12206941000033119	Esmolol 2.5g powder for solution for infusion vials	Beta blocker
544641000033119	Esmolol 2.5g/10ml solution	Beta blocker
5655541000033112	Esmolol 2.5g/250ml infusion bags	Beta blocker
544541000033115	Esmolol Hydrochloride Injection 10 mg/ml	Beta blocker
556941000033118	Eucardic 12.5mg tablets	Beta blocker
557041000033117	Eucardic 25mg tablets	Beta blocker
1608941000033115	Eucardic 3.125mg tablets	Beta blocker
1609041000033112	Eucardic 6.25mg tablets	Beta blocker
557141000033118	Eucardic Tablets 50 mg	Beta blocker
660741000033115	Half Beta-Prograne 80mg modified-release capsules	Beta blocker
658241000033117	Half Inderal LA 80mg capsules	Beta blocker
660941000033117	Half Propanix La M/R capsules 80 mg	Beta blocker
660541000033111	Half-Betadur Cr M/R capsules 80 mg	Beta blocker
5052641000033111	Hypoloc 5mg tablets	Beta blocker
768741000033110	Inderal 10mg tablets	Beta blocker
759641000033116	Inderal 1mg/1ml solution	Beta blocker
768841000033117	Inderal 40mg tablets	Beta blocker
768941000033113	Inderal 80mg tablets	Beta blocker
756541000033113	Inderal LA 160mg capsules	Beta blocker
803641000033114	Kerlone 20mg tablets	Beta blocker
814041000033119	Labetalol 100mg tablets	Beta blocker
811341000033119	Labetalol 100mg/20ml solution	Beta blocker
818641000033117	Labetalol 200mg tablets	Beta blocker
818741000033114	Labetalol 400mg tablets	Beta blocker
818841000033116	Labetalol 50mg tablets	Beta blocker

6487941000033119	Labetalol 50mg/5ml oral solution	Beta blocker
814141000033115	Labrocol Tablets 100 mg	Beta blocker
814241000033110	Labrocol Tablets 200 mg	Beta blocker
814341000033117	Labrocol Tablets 400 mg	Beta blocker
850841000033110	Lopresor 100mg tablets	Beta blocker
850941000033119	Lopresor 50mg tablets	Beta blocker
844241000033116	Lopresor Injection 1 mg/ml	Beta blocker
5333041000033112	Lopresor Retard M/R tablets 200 mg	Beta blocker
851041000033112	Lopresor SR 200mg tablets	Beta blocker
894741000033117	Mepranix 100 Tablets 100 mg	Beta blocker
894641000033114	Mepranix 50 Tablets 50 mg	Beta blocker
897741000033111	Metoprolol 100mg tablets	Beta blocker
2616041000033116	Metoprolol 12.5mg/5ml oral solution	Beta blocker
4815641000033117	Metoprolol 12.5mg/5ml oral suspension	Beta blocker
891641000033111	Metoprolol 200mg modified-release tablets	Beta blocker
5575541000033119	Metoprolol 25mg/5ml oral solution	Beta blocker
897841000033118	Metoprolol 50mg tablets	Beta blocker
5972841000033117	Metoprolol 50mg/5ml oral solution	Beta blocker
5972941000033113	Metoprolol 50mg/5ml oral suspension	Beta blocker
887141000033113	Metoprolol 5mg/5ml solution	Beta blocker
890741000033112	Metoprolol Fumarate S/r tablets 95 mg	Beta blocker
903641000033115	Metoprolol Tartrate Tablets 200 mg	Beta blocker
891841000033112	Metoros Ls Sr tablets 95 mg	Beta blocker
891741000033119	Metoros Sr tablets 190 mg	Beta blocker
939241000033113	Monocor 10mg tablets	Beta blocker
939141000033118	Monocor 5mg tablets	Beta blocker
5814141000033119	Nadolol 20mg/5ml oral solution	Beta blocker
10650041000033116	Nadolol 20mg/5ml oral suspension	Beta blocker
7871341000033114	Nadolol 30mg/5ml oral solution	Beta blocker
10650141000033116	Nadolol 30mg/5ml oral suspension	Beta blocker
10650141000033117	Nadolol 30mg/5ml oral suspension	Beta blocker
956341000033113	Nadolol 40mg tablets	Beta blocker
5378241000033112	Nadolol 40mg/5ml oral suspension	Beta blocker
956541000033118	Nadolol 80mg tablets	Beta blocker
1796141000033112	Nebilet 5mg tablets	Beta blocker
8961141000033117	Nebivolol 10mg tablets	Beta blocker
5565341000033115	Nebivolol 2.5mg tablets	Beta blocker
1796041000033113	Nebivolol 5mg tablets	Beta blocker
1026041000033118	Oxprenolol 160mg modified-release tablets	Beta blocker
1026141000033119	Oxprenolol 20mg tablets	Beta blocker
1026241000033114	Oxprenolol 40mg tablets	Beta blocker
1026341000033116	Oxprenolol 80mg tablets	Beta blocker
1023841000033116	Oxprenolol Hydrochloride Injection 2 mg/amp	Beta blocker
1027241000033112	Oxprenolol Hydrochloride Tablets 160 mg	Beta blocker
1025141000033114	Oxyprenix-160-Sr Sr tablets 160 mg	Beta blocker
3163141000033117	Pindolol 10mg / Clopamide 5mg tablets	Beta blocker
1091141000033117	Pindolol 15mg tablets	Beta blocker

1091241000033112	Pindolol 5mg tablets	Beta blocker
1124341000033119	Probeta La M/R capsules 160 mg	Beta blocker
1130341000033114	Propanix 10 Tablets 10 mg	Beta blocker
1127941000033110	Propanix 160 Sr capsules 160 mg	Beta blocker
1130641000033118	Propanix 160 Tablets 160 mg	Beta blocker
1130441000033115	Propanix 40 Tablets 40 mg	Beta blocker
1130541000033119	Propanix 80 Tablets 80 mg	Beta blocker
1124441000033113	Propanix La M/R capsules 160 mg	Beta blocker
1135041000033117	Propranolol 10mg tablets	Beta blocker
1125341000033118	Propranolol 10mg/5ml oral solution sugar free	Beta blocker
1124041000033116	Propranolol 160mg modified-release capsules	Beta blocker
1135141000033118	Propranolol 160mg tablets	Beta blocker
1119641000033117	Propranolol 1mg/1ml solution	Beta blocker
1135241000033113	Propranolol 40mg tablets	Beta blocker
1750241000033113	Propranolol 40mg/5ml oral solution	Beta blocker
4955841000033115	Propranolol 40mg/5ml oral solution sugar free	Beta blocker
5998141000033117	Propranolol 40mg/5ml oral suspension	Beta blocker
1125541000033113	Propranolol 50mg/5ml oral solution sugar free	Beta blocker
1125441000033112	Propranolol 5mg/5ml oral solution sugar free	Beta blocker
1130041000033112	Propranolol 80mg modified-release capsules	Beta blocker
1135341000033115	Propranolol 80mg tablets	Beta blocker
4957141000033117	Propranolol 80mg/5ml oral solution	Beta blocker
1111441000033118	Propranolol Hydrochloride Capsules 160 mg	Beta blocker
1112341000033115	Propranolol Hydrochloride Capsules 80 mg	Beta blocker
1128041000033113	Propranolol Hydrochloride M/R tablets 160 mg	Beta blocker
2839841000033118	Rapranol SR 160mg capsules	Beta blocker
2839941000033114	Rapranol SR 80mg capsules	Beta blocker
1268141000033119	Sectral 100mg capsules	Beta blocker
1268241000033114	Sectral 200mg capsules	Beta blocker
1278241000033118	Sectral 400mg tablets	Beta blocker
2929141000033117	Slo-Pro 160mg capsules	Beta blocker
1347641000033111	Slow-Pren Tablets 160 mg	Beta blocker
1347441000033114	Slow-Trasicor 160mg tablets	Beta blocker
2802041000033113	Soloc 10mg tablets	Beta blocker
2801941000033119	Soloc 5mg tablets	Beta blocker
1366041000033112	Sotacor 160mg tablets	Beta blocker
1358541000033113	Sotacor 40mg/4ml solution	Beta blocker
1366141000033111	Sotacor 80mg tablets	Beta blocker
1367141000033113	Sotalol 160mg tablets	Beta blocker
1367241000033118	Sotalol 200mg tablets	Beta blocker
3953441000033116	Sotalol 25mg/5ml oral solution	Beta blocker
9776441000033119	Sotalol 25mg/5ml oral suspension	Beta blocker
9776441000033120	Sotalol 25mg/5ml oral suspension	Beta blocker
1367441000033117	Sotalol 40mg tablets	Beta blocker
2908541000033114	Sotalol 40mg/4ml solution	Beta blocker
1367541000033116	Sotalol 80mg tablets	Beta blocker
1358641000033114	Sotalol Hydrochloride Injection 10 mg/ml	Beta blocker

2267541000033111	Syprol 10mg/5ml oral solution	Beta blocker
4955941000033111	Syprol 40mg/5ml oral solution	Beta blocker
2267641000033112	Syprol 50mg/5ml oral solution	Beta blocker
2267441000033110	Syprol 5mg/5ml oral solution	Beta blocker
1426441000033116	Tenormin 100mg tablets	Beta blocker
1428741000033112	Tenormin 25mg tablets	Beta blocker
1424541000033110	Tenormin 25mg/5ml syrup	Beta blocker
1420741000033114	Tenormin 5mg/10ml solution	Beta blocker
1426541000033115	Tenormin LS 50mg tablets	Beta blocker
1444541000033113	Timolol 10mg tablets	Beta blocker
1453041000033116	Totamol Tablets 100 mg	Beta blocker
1449641000033110	Totamol Tablets 25 mg	Beta blocker
1453141000033117	Totamol Tablets 50 mg	Beta blocker
1469041000033113	Trandate 100mg tablets	Beta blocker
1461441000033115	Trandate 100mg/20ml solution	Beta blocker
1469141000033112	Trandate 200mg tablets	Beta blocker
1469241000033117	Trandate 400mg tablets	Beta blocker
1469341000033110	Trandate 50mg tablets	Beta blocker
1469641000033119	Trasicor 20mg tablets	Beta blocker
1469741000033111	Trasicor 40mg tablets	Beta blocker
1469841000033118	Trasicor 80mg tablets	Beta blocker
1469541000033115	Trasicor Tablets 160 mg	Beta blocker
1505241000033113	Vasaten Tablets 100 mg	Beta blocker
1505341000033115	Vasaten Tablets 50 mg	Beta blocker
1523941000033114	Viskaldix tablets	Beta blocker
1525141000033111	Visken 15mg tablets	Beta blocker
1524041000033111	Visken 5mg tablets	Beta blocker
2866141000033114	Vivacor 10mg tablets	Beta blocker
2866041000033110	Vivacor 5mg tablets	Beta blocker
14041000033115	Adalat 10mg capsules	Calcium channel blocker
14141000033116	Adalat 5mg capsules	Calcium channel blocker
14241000033111	Adalat I C Coronary injection 100 mcg/ml	Calcium channel blocker
1766341000033113	Adalat LA 20mg tablets	Calcium channel blocker
17141000033112	Adalat LA 30mg tablets	Calcium channel blocker
17241000033117	Adalat LA 60mg tablets	Calcium channel blocker
19441000033113	Adalat retard 10mg tablets	Calcium channel blocker
19341000033119	Adalat retard 20mg tablets	Calcium channel blocker
5891941000033115	Adanif XL 30mg tablets	Calcium channel blocker
5892041000033114	Adanif XL 60mg tablets	Calcium channel blocker
1820541000033114	Adipine LA 30 M/R tablets 30 mg	Calcium channel blocker
1820441000033113	Adipine LA 60 M/R tablets 60 mg	Calcium channel blocker
18341000033114	Adipine MR 10 tablets	Calcium channel blocker
18241000033116	Adipine MR 20 tablets	Calcium channel blocker
3225441000033117	Adipine XL 30mg tablets	Calcium channel blocker
3225541000033116	Adipine XL 60mg tablets	Calcium channel blocker
13841000033113	Adizem Capsules 60 mg	Calcium channel blocker
19741000033118	Adizem Continus Tablets 120 mg	Calcium channel blocker

18141000033111	Adizem-60 M/R tablets 60 mg	Calcium channel blocker
17341000033110	Adizem-SR 120mg capsules	Calcium channel blocker
19641000033110	Adizem-SR 120mg tablets	Calcium channel blocker
17441000033116	Adizem-SR 180mg capsules	Calcium channel blocker
17641000033119	Adizem-SR 90mg capsules	Calcium channel blocker
17541000033115	Adizem-Sr M/R capsules 300 mg	Calcium channel blocker
19541000033114	Adizem-Sr Tablets 60 mg	Calcium channel blocker
17741000033111	Adizem-XL 120mg capsules	Calcium channel blocker
17841000033118	Adizem-XL 180mg capsules	Calcium channel blocker
2653341000033118	Adizem-XL 200mg capsules	Calcium channel blocker
17941000033114	Adizem-XL 240mg capsules	Calcium channel blocker
18041000033112	Adizem-XL 300mg capsules	Calcium channel blocker
4897741000033118	Amlodipine 1.5mg/5ml oral suspension	Calcium channel blocker
3038141000033110	Amlodipine 10mg tablets	Calcium channel blocker
7740641000033112	Amlodipine 10mg/5ml oral solution	Calcium channel blocker
10387441000033111	Amlodipine 10mg/5ml oral solution sugar free	Calcium channel blocker
10387441000033112	Amlodipine 10mg/5ml oral solution sugar free	Calcium channel blocker
3963741000033115	Amlodipine 10mg/5ml oral suspension	Calcium channel blocker
3038041000033111	Amlodipine 5mg tablets	Calcium channel blocker
5490241000033113	Amlodipine 5mg/5ml oral solution	Calcium channel blocker
4508041000033119	Amlodipine 5mg/5ml oral solution sugar free	Calcium channel blocker
5888741000033110	Amlodipine 5mg/5ml oral suspension	Calcium channel blocker
12682241000033110	Amlodipine 5mg/5ml oral suspension sugar free	Calcium channel blocker
60741000033111	Amlodipine Besilate Tablets 10 mg	Calcium channel blocker
57241000033113	Amlodipine Besilate Tablets 5 mg	Calcium channel blocker
3890141000033118	Amlodipine Powder	Calcium channel blocker
3188841000033118	Amlostin 10mg tablets	Calcium channel blocker
3188741000033111	Amlostin 5mg tablets	Calcium channel blocker
61841000033111	Angiopine 10 capsules	Calcium channel blocker
65841000033113	Angiopine 40 La M/R tablets 40 mg	Calcium channel blocker
61741000033118	Angiopine Capsules 5 mg	Calcium channel blocker
66141000033114	Angiopine MR 10mg tablets	Calcium channel blocker
65241000033114	Angiopine MR 20mg tablets	Calcium channel blocker
71141000033114	Angiozem 60mg modified-release tablets	Calcium channel blocker
66041000033110	Angiozem CR 120mg tablets	Calcium channel blocker
65941000033117	Angiozem CR 90mg tablets	Calcium channel blocker
65441000033110	Angitil SR 120 capsules	Calcium channel blocker
65541000033111	Angitil SR 180 capsules	Calcium channel blocker
65341000033116	Angitil SR 90 capsules	Calcium channel blocker
1570441000033110	Angitil XL 240 capsules	Calcium channel blocker
1570541000033111	Angitil XL 300 capsules	Calcium channel blocker
2779341000033110	Bi-Carzem SR 120mg capsules	Calcium channel blocker
2779141000033112	Bi-Carzem SR 60mg capsules	Calcium channel blocker
2779241000033117	Bi-Carzem SR 90mg capsules	Calcium channel blocker
5816841000033119	Bi-Carzem XL 240mg capsules	Calcium channel blocker
5816941000033110	Bi-Carzem XL 300mg capsules	Calcium channel blocker
163541000033117	Britiazim Tablets 60 mg	Calcium channel blocker

2929041000033116	Cabren 10mg modified-release tablets	Calcium channel blocker
2928841000033117	Cabren 2.5mg modified-release tablets	Calcium channel blocker
2928941000033113	Cabren 5mg modified-release tablets	Calcium channel blocker
210241000033111	Calanif Capsules 10 mg	Calcium channel blocker
210341000033118	Calanif Capsules 5 mg	Calcium channel blocker
1580941000033112	Calazem M/R tablets 60 mg	Calcium channel blocker
2295641000033116	Calchan MR 10 tablets	Calcium channel blocker
2295741000033113	Calchan MR 20 tablets	Calcium channel blocker
199241000033119	Calcicard CR 120mg tablets	Calcium channel blocker
199341000033112	Calcicard CR 90mg tablets	Calcium channel blocker
216641000033112	Calcicard Tablets 60 mg	Calcium channel blocker
176741000033114	Cardene 20mg capsules	Calcium channel blocker
176841000033116	Cardene 30mg capsules	Calcium channel blocker
177241000033117	Cardene Capsules	Calcium channel blocker
198941000033118	Cardene SR 30mg capsules	Calcium channel blocker
199041000033110	Cardene SR 45mg capsules	Calcium channel blocker
1580841000033116	Cardilate MR 10mg tablets	Calcium channel blocker
199141000033114	Cardilate MR 20mg tablets	Calcium channel blocker
3154941000033110	Cardioplen XL 10mg tablets	Calcium channel blocker
4152841000033112	Cardioplen XL 2.5mg tablets	Calcium channel blocker
3154841000033119	Cardioplen XL 5mg tablets	Calcium channel blocker
11072441000033111	Clevidipine 25mg/50ml emulsion for infusion vials	Calcium channel blocker
11072441000033112	Clevidipine 25mg/50ml emulsion for infusion vials	Calcium channel blocker
11072541000033112	Cleviprex 25mg/50ml emulsion	Calcium channel blocker
286241000033112	Clinium Tablets 120 mg	Calcium channel blocker
336641000033114	Coracten SR 10mg capsules	Calcium channel blocker
364241000033112	Coracten SR 20mg capsules	Calcium channel blocker
1724941000033113	Coracten XL 30mg capsules	Calcium channel blocker
1725041000033113	Coracten XL 60mg capsules	Calcium channel blocker
374841000033119	Cordilox 120mg tablets	Calcium channel blocker
374941000033110	Cordilox 160mg tablets	Calcium channel blocker
375041000033110	Cordilox 40mg tablets	Calcium channel blocker
375141000033114	Cordilox 80mg tablets	Calcium channel blocker
333841000033118	Cordilox Injection 2.5 mg/ml, 2 ml ampoule	Calcium channel blocker
2129041000033118	Cordilox MR 240mg tablets	Calcium channel blocker
2051241000033116	Coroday MR 20mg tablets	Calcium channel blocker
1602341000033116	Dilcardia SR 120mg capsules	Calcium channel blocker
1602441000033110	Dilcardia SR 60mg capsules	Calcium channel blocker
1602541000033111	Dilcardia SR 90mg capsules	Calcium channel blocker
1727641000033113	Dilcardia Xl M/R capsules 120 mg	Calcium channel blocker
1727741000033116	Dilcardia Xl M/R capsules 180 mg	Calcium channel blocker
1727841000033114	Dilcardia Xl M/R capsules 240 mg	Calcium channel blocker
451841000033110	Diltiazem 120mg modified-release capsules	Calcium channel blocker
459641000033113	Diltiazem 120mg modified-release tablets	Calcium channel blocker
451941000033119	Diltiazem 180mg modified-release capsules	Calcium channel blocker
453941000033115	Diltiazem 200mg modified-release capsules	Calcium channel blocker
453741000033118	Diltiazem 240mg modified-release capsules	Calcium channel blocker

Carr MJ, *et al. BMJ Qual Saf* 2022; 31:503–514. doi: 10.1136/bmjqs-2021-013613

452041000033113	Diltiazem 300mg modified-release capsules	Calcium channel blocker
1800341000033113	Diltiazem 360mg modified-release capsules	Calcium channel blocker
452941000033114	Diltiazem 60mg modified-release capsules	Calcium channel blocker
454041000033118	Diltiazem 60mg modified-release tablets	Calcium channel blocker
5991941000033112	Diltiazem 60mg/5ml oral solution	Calcium channel blocker
4953741000033114	Diltiazem 60mg/5ml oral suspension	Calcium channel blocker
452141000033112	Diltiazem 90mg modified-release capsules	Calcium channel blocker
458841000033115	Diltiazem 90mg modified-release tablets	Calcium channel blocker
451741000033117	Diltiazem Hydrochloride M/R capsules 300 mg	Calcium channel blocker
468841000033112	Diltiazem Hydrochloride Tablets 60 mg	Calcium channel blocker
452841000033118	Dilzem SR 120 capsules	Calcium channel blocker
452641000033119	Dilzem SR 60 capsules	Calcium channel blocker
452741000033111	Dilzem SR 90 capsules	Calcium channel blocker
453041000033116	Dilzem XL 120 capsules	Calcium channel blocker
453141000033117	Dilzem XL 180 capsules	Calcium channel blocker
453241000033112	Dilzem XL 240 capsules	Calcium channel blocker
2675041000033119	Disogram SR 120mg capsules	Calcium channel blocker
2675341000033117	Disogram SR 180mg capsules	Calcium channel blocker
2675641000033113	Disogram SR 240mg capsules	Calcium channel blocker
2676041000033111	Disogram SR 300mg capsules	Calcium channel blocker
2674841000033110	Disogram SR 60mg capsules	Calcium channel blocker
2674941000033119	Disogram SR 90mg capsules	Calcium channel blocker
2104741000033113	Ethimil MR 240mg tablets	Calcium channel blocker
4504141000033110	Felendil Xl M/R tablets 10 mg	Calcium channel blocker
4503941000033114	Felendil Xl M/R tablets 2.5 mg	Calcium channel blocker
4504041000033111	Felendil Xl M/R tablets 5 mg	Calcium channel blocker
569041000033115	Felodipine 10mg modified-release tablets	Calcium channel blocker
568041000033111	Felodipine 2.5mg modified-release tablets	Calcium channel blocker
569141000033116	Felodipine 5mg modified-release tablets	Calcium channel blocker
3177541000033111	Felogen XL 10mg tablets	Calcium channel blocker
3177441000033110	Felogen XL 5mg tablets	Calcium channel blocker
2980041000033115	Felotens XL 10mg tablets	Calcium channel blocker
4429241000033115	Felotens XL 2.5mg tablets	Calcium channel blocker
2979941000033119	Felotens XL 5mg tablets	Calcium channel blocker
5576341000033118	Folpik XL 10mg tablets	Calcium channel blocker
5576441000033112	Folpik XL 2.5mg tablets	Calcium channel blocker
5576541000033113	Folpik XL 5mg tablets	Calcium channel blocker
605941000033110	Fortipine LA 40 tablets	Calcium channel blocker
660641000033112	Half Securon SR 120mg tablets	Calcium channel blocker
2739041000033110	Horizem SR 120mg capsules	Calcium channel blocker
2739141000033114	Horizem SR 90mg capsules	Calcium channel blocker
738141000033119	Hypolar Retard 20 tablets	Calcium channel blocker
2776541000033111	Hypolar XL 30 tablets	Calcium channel blocker
784641000033112	Isradipine 2.5mg tablets	Calcium channel blocker
788041000033110	Istin 10mg tablets	Calcium channel blocker
787941000033112	Istin 5mg tablets	Calcium channel blocker
9118441000033116	Kenzem SR 120mg capsules	Calcium channel blocker

9118241000033116	Kenzem SR 60mg capsules	Calcium channel blocker
9118241000033117	Kenzem SR 60mg capsules	Calcium channel blocker
9118341000033110	Kenzem SR 90mg capsules	Calcium channel blocker
817541000033119	Lacidipine 2mg tablets	Calcium channel blocker
817641000033118	Lacidipine 4mg tablets	Calcium channel blocker
825941000033112	Lercanidipine 10mg tablets	Calcium channel blocker
3908741000033114	Lercanidipine 20mg tablets	Calcium channel blocker
835341000033111	Lidoflazine Tablets 120 mg	Calcium channel blocker
919741000033112	Mibefradil Dihydrochloride Tablets 100 mg	Calcium channel blocker
919841000033119	Mibefradil Dihydrochloride Tablets 50 mg	Calcium channel blocker
10217641000033116	Molap 4mg tablets	Calcium channel blocker
937041000033114	Motens 2mg tablets	Calcium channel blocker
937141000033113	Motens 4mg tablets	Calcium channel blocker
4521741000033117	Neofel XL 10mg tablets	Calcium channel blocker
4521541000033113	Neofel XL 2.5mg tablets	Calcium channel blocker
4521641000033114	Neofel XL 5mg tablets	Calcium channel blocker
5817441000033119	Neozipine XL 30mg tablets	Calcium channel blocker
5817541000033118	Neozipine XL 60mg tablets	Calcium channel blocker
9697741000033114	Nicardipine 10mg/10ml solution	Calcium channel blocker
967741000033115	Nicardipine 20mg capsules	Calcium channel blocker
967141000033119	Nicardipine 30mg capsules	Calcium channel blocker
971441000033112	Nicardipine 30mg modified-release capsules	Calcium channel blocker
971541000033113	Nicardipine 45mg modified-release capsules	Calcium channel blocker
12372341000033116	Nidef 30mg modified-release tablets	Calcium channel blocker
12372341000033117	Nidef 30mg modified-release tablets	Calcium channel blocker
12372441000033111	Nidef 60mg modified-release tablets	Calcium channel blocker
12372441000033112	Nidef 60mg modified-release tablets	Calcium channel blocker
967241000033114	Nifedipine 10mg capsules	Calcium channel blocker
971141000033116	Nifedipine 10mg modified-release capsules	Calcium channel blocker
971841000033110	Nifedipine 10mg modified-release tablets	Calcium channel blocker
5973741000033117	Nifedipine 10mg/5ml oral suspension	Calcium channel blocker
5402541000033112	Nifedipine 2.5mg/5ml oral suspension	Calcium channel blocker
974141000033119	Nifedipine 20mg modified-release capsules	Calcium channel blocker
970941000033113	Nifedipine 20mg modified-release tablets	Calcium channel blocker
2639341000033117	Nifedipine 20mg/ml oral drops	Calcium channel blocker
1778741000033118	Nifedipine 30mg modified-release capsules	Calcium channel blocker
971241000033111	Nifedipine 30mg modified-release tablets	Calcium channel blocker
972441000033116	Nifedipine 40mg modified-release tablets	Calcium channel blocker
967341000033116	Nifedipine 5mg capsules	Calcium channel blocker
5056241000033112	Nifedipine 5mg/5ml oral suspension	Calcium channel blocker
1778841000033111	Nifedipine 60mg modified-release capsules	Calcium channel blocker
971341000033118	Nifedipine 60mg modified-release tablets	Calcium channel blocker
977641000033117	Nifedipine Tablets 10 mg	Calcium channel blocker
977341000033113	Nifedipine Tablets 10 mg	Calcium channel blocker
974941000033117	Nifedipine Tablets 20 mg	Calcium channel blocker
1745041000033110	Nifedipress MR 10 tablets	Calcium channel blocker
1745141000033114	Nifedipress MR 20 tablets	Calcium channel blocker

2068941000033111	Nifedotard 20 MR M/R tablets 20 mg	Calcium channel blocker
971741000033117	Nifelease M/R tablets 20 mg	Calcium channel blocker
971041000033115	Nifensar Xl M/R tablets 20 mg	Calcium channel blocker
2189541000033117	Nifopress Retard 20mg tablets	Calcium channel blocker
970741000033110	Nimodipine 10mg/50ml solution for infusion vials	Calcium channel blocker
977441000033119	Nimodipine 30mg tablets	Calcium channel blocker
971941000033119	Nimodrel Mr M/R tablets 10 mg	Calcium channel blocker
972041000033113	Nimodrel Mr M/R tablets 20 mg	Calcium channel blocker
3952641000033110	Nimodrel XL 30mg tablets	Calcium channel blocker
3952741000033118	Nimodrel XL 60mg tablets	Calcium channel blocker
970841000033117	Nimotop 0.02% solution	Calcium channel blocker
977741000033114	Nimotop 30mg tablets	Calcium channel blocker
972141000033112	Nisoldipine 10mg modified-release tablets	Calcium channel blocker
972241000033117	Nisoldipine 20mg modified-release tablets	Calcium channel blocker
972341000033110	Nisoldipine 30mg modified-release tablets	Calcium channel blocker
1659641000033111	Nivaten Retard M/R tablets 10 mg	Calcium channel blocker
1776541000033110	Nivaten Retard M/R tablets 20 mg	Calcium channel blocker
1661641000033116	Optil 60mg modified-release tablets	Calcium channel blocker
1747141000033117	Optil Sr 120 M/R capsules 120 mg	Calcium channel blocker
1747241000033112	Optil Sr 180 M/R capsules 180 mg	Calcium channel blocker
1747041000033116	Optil Sr 90 M/R capsules 90 mg	Calcium channel blocker
1747341000033119	Optil Xl 240 M/R capsules 240 mg	Calcium channel blocker
1747441000033113	Optil Xl 300 M/R capsules 300 mg	Calcium channel blocker
1039741000033114	Parmid	Calcium channel blocker
1041441000033117	Parmid	Calcium channel blocker
1035141000033112	Parmid	Calcium channel blocker
9121741000033113	Parmid XL 10mg tablets	Calcium channel blocker
9121741000033112	Parmid XL 10mg tablets	Calcium channel blocker
9121541000033116	Parmid XL 2.5mg tablets	Calcium channel blocker
9121541000033117	Parmid XL 2.5mg tablets	Calcium channel blocker
9121641000033116	Parmid XL 5mg tablets	Calcium channel blocker
1094041000033119	Plendil 10mg modified-release tablets	Calcium channel blocker
1093941000033116	Plendil 2.5mg modified-release tablets	Calcium channel blocker
1094141000033115	Plendil 5mg modified-release tablets	Calcium channel blocker
1109841000033118	Posicor Tablets 100 mg	Calcium channel blocker
1109941000033114	Posicor Tablets 50 mg	Calcium channel blocker
1140241000033117	Prescal 2.5mg tablets	Calcium channel blocker
2845841000033119	Ranvera MR 240mg tablets	Calcium channel blocker
4590741000033116	Retalzem 60 modified-release tablets	Calcium channel blocker
1274741000033119	Securon 120mg tablets	Calcium channel blocker
1271741000033116	Securon IV 5mg/2ml solution	Calcium channel blocker
1275141000033117	Securon SR 240mg tablets	Calcium channel blocker
1274841000033112	Securon Tablets 160 mg	Calcium channel blocker
1274941000033116	Securon Tablets 40 mg	Calcium channel blocker
1275041000033116	Securon Tablets 80 mg	Calcium channel blocker
1697641000033111	Slofedipine 20mg tablets	Calcium channel blocker
1752941000033115	Slofedipine XL 30mg tablets	Calcium channel blocker

1753041000033113	Slofedipine XL 60 tablets	Calcium channel blocker
1346441000033111	Slozem 120mg capsules	Calcium channel blocker
1346541000033112	Slozem 180mg capsules	Calcium channel blocker
1346641000033113	Slozem 240mg capsules	Calcium channel blocker
2261241000033118	Slozem 300mg capsules	Calcium channel blocker
1401341000033110	Syscor MR 10 tablets	Calcium channel blocker
1401441000033116	Syscor MR 20 tablets	Calcium channel blocker
1401541000033115	Syscor MR 30 tablets	Calcium channel blocker
1421241000033110	Tensipine MR 10 tablets	Calcium channel blocker
1421341000033117	Tensipine MR 20 tablets	Calcium channel blocker
1443841000033113	Tildiem 60mg modified-release tablets	Calcium channel blocker
1441841000033112	Tildiem LA 200 capsules	Calcium channel blocker
1441741000033119	Tildiem LA 300 capsules	Calcium channel blocker
1442741000033113	Tildiem Retard 120mg tablets	Calcium channel blocker
1442841000033115	Tildiem Retard 90mg tablets	Calcium channel blocker
1442541000033117	Tildiem S/r tablets 120 mg	Calcium channel blocker
1442641000033116	Tildiem S/r tablets 90 mg	Calcium channel blocker
1487641000033112	Unipine Xl M/R tablets 30 mg	Calcium channel blocker
1489541000033117	Univer 120mg modified-release capsules	Calcium channel blocker
1489641000033116	Univer 180mg modified-release capsules	Calcium channel blocker
1489741000033113	Univer 240mg modified-release capsules	Calcium channel blocker
2955041000033115	Valni 20 Retard tablets	Calcium channel blocker
4451941000033110	Valni XL 30mg tablets	Calcium channel blocker
4452041000033116	Valni XL 60mg tablets	Calcium channel blocker
3034441000033111	Vascalpha 10mg modified-release tablets	Calcium channel blocker
3034341000033117	Vascalpha 5mg modified-release tablets	Calcium channel blocker
2890641000033116	Vera-Til SR 120mg tablets	Calcium channel blocker
2890741000033113	Vera-Til SR 240mg tablets	Calcium channel blocker
1511141000033118	Verapamil 120mg modified-release capsules	Calcium channel blocker
1509041000033119	Verapamil 120mg modified-release tablets	Calcium channel blocker
1512941000033111	Verapamil 120mg tablets	Calcium channel blocker
1513141000033119	Verapamil 160mg tablets	Calcium channel blocker
1511241000033113	Verapamil 180mg modified-release capsules	Calcium channel blocker
1511341000033115	Verapamil 240mg modified-release capsules	Calcium channel blocker
1509141000033115	Verapamil 240mg modified-release tablets	Calcium channel blocker
1513241000033114	Verapamil 40mg tablets	Calcium channel blocker
1509841000033114	Verapamil 40mg/5ml oral solution sugar free	Calcium channel blocker
4260341000033110	Verapamil 5mg/2ml solution for injection ampoules	Calcium channel blocker
1513341000033116	Verapamil 80mg tablets	Calcium channel blocker
1508641000033116	Verapamil Hydrochloride Injection 2.5 mg/ml	Calcium channel blocker
1511741000033119	Verapamil Hydrochloride M/R capsules 240 mg	Calcium channel blocker
1513041000033118	Verapamil Hydrochloride Tablets 240 mg	Calcium channel blocker
1833541000033117	Verapress MR 240mg tablets	Calcium channel blocker
1754841000033116	Vertab SR 240 tablets	Calcium channel blocker
1520141000033118	Viazem XL 120mg capsules	Calcium channel blocker
1520241000033113	Viazem XL 180mg capsules	Calcium channel blocker
1520341000033115	Viazem XL 240mg capsules	Calcium channel blocker

1520441000033114	Viazem XL 300mg capsules	Calcium channel blocker
1520541000033110	Viazem XL 360mg capsules	Calcium channel blocker
1550841000033111	Zanidip 10mg tablets	Calcium channel blocker
3908841000033116	Zanidip 20mg tablets	Calcium channel blocker
2955141000033116	Zemret 180 XL capsules	Calcium channel blocker
2955241000033111	Zemret 240 XL capsules	Calcium channel blocker
2955341000033118	Zemret 300 XL capsules	Calcium channel blocker
1833041000033110	Zemtard 120 XL capsules	Calcium channel blocker
1833141000033114	Zemtard 180 XL capsules	Calcium channel blocker
1833241000033119	Zemtard 240 XL capsules	Calcium channel blocker
1833341000033112	Zemtard 300 XL capsules	Calcium channel blocker
2952341000033113	Zildil 90mg modified-release capsules	Calcium channel blocker
2952441000033119	Zildil SR 120mg capsules	Calcium channel blocker
2952241000033115	Zildil SR 60mg capsules	Calcium channel blocker
2641041000033116	Zolvera 40mg/5ml oral solution	Calcium channel blocker
40941000033118	Aldomet 250mg tablets	Central-acting
41041000033111	Aldomet 500mg tablets	Central-acting
31341000033114	Aldomet Injection 50 mg/ml	Central-acting
36841000033113	Aldomet Suspension 250 mg/5 ml	Central-acting
40841000033114	Aldomet Tablets 125 mg	Central-acting
215341000033112	Catapres 100microgram tablets	Central-acting
196241000033111	Catapres 150micrograms/1ml solution	Central-acting
215441000033118	Catapres 300microgram tablets	Central-acting
177441000033116	Catapres Capsules 0.25 mg	Central-acting
177641000033119	Catapres Perlongets M/R capsules 250 micrograms	Central-acting
12107141000033117	Catapres TTS 1 patches	Central-acting
12117941000033118	Catapres TTS 2 patches	Central-acting
12118541000033113	Catapres TTS 3 patches	Central-acting
285841000033118	Clonidine 100microgram tablets	Central-acting
12106941000033117	Clonidine 100micrograms/24hours transdermal patches	Central-acting
12569041000033115	Clonidine 100micrograms/5ml oral suspension	Central-acting
275641000033110	Clonidine 150micrograms/1ml solution	Central-acting
5289041000033110	Clonidine 200micrograms/24hours transdermal patches	Central-acting
261841000033118	Clonidine 250microgram modified-release capsules	Central-acting
285941000033114	Clonidine 25microgram tablets	Central-acting
287241000033110	Clonidine 300microgram tablets	Central-acting
12107041000033116	Clonidine 300micrograms/24hours transdermal patches	Central-acting
5900141000033118	Clonidine 50micrograms/5ml oral solution	Central-acting
12684041000033114	Clonidine 50micrograms/5ml oral solution sugar free	Central-acting
5900241000033113	Clonidine 50micrograms/5ml oral suspension	Central-acting
2776441000033110	Clonidine Hydrochloride Transdermal patches	Central-acting
431241000033112	Decaserpyl Plus Tablets	Central-acting
430941000033114	Decaserpyl Tablets 10 mg	Central-acting
431041000033116	Decaserpyl Tablets 100 micrograms	Central-acting
431141000033117	Decaserpyl Tablets 5 mg	Central-acting
464341000033111	Dixarit 25microgram tablets	Central-acting
481341000033111	Dopamet Tablets 125 mg	Central-acting

481441000033117	Dopamet Tablets 250 mg	Central-acting
481541000033116	Dopamet Tablets 500 mg	Central-acting
10740841000033116	Guanfacine 1mg modified-release tablets	Central-acting
10740941000033112	Guanfacine 2mg modified-release tablets	Central-acting
10741041000033119	Guanfacine 3mg modified-release tablets	Central-acting
10741141000033115	Guanfacine 4mg modified-release tablets	Central-acting
742441000033111	Hydromet Tablets	Central-acting
743441000033119	Hypercal Tablets 2 mg	Central-acting
10741241000033110	Intuniv 1mg modified-release tablets	Central-acting
10741341000033117	Intuniv 2mg modified-release tablets	Central-acting
10741441000033111	Intuniv 3mg modified-release tablets	Central-acting
10741541000033112	Intuniv 4mg modified-release tablets	Central-acting
872541000033114	Medomet Capsules 250 mg	Central-acting
895141000033115	Medomet Tablets 250 mg	Central-acting
895241000033110	Medomet Tablets 500 mg	Central-acting
902441000033114	Methoserpidine Tablets 10 mg	Central-acting
903941000033110	Methoserpidine Tablets 5 mg	Central-acting
897341000033110	Methyldopa 125mg tablets	Central-acting
897441000033116	Methyldopa 250mg tablets	Central-acting
892341000033112	Methyldopa 250mg/5ml oral suspension	Central-acting
897541000033115	Methyldopa 500mg tablets	Central-acting
873941000033114	Methyldopa Capsules 250 mg	Central-acting
878541000033112	Methyldopa Injection 50 mg/ml	Central-acting
938541000033115	Moxonidine 200microgram tablets	Central-acting
2635641000033111	Moxonidine 300microgram tablets	Central-acting
938641000033119	Moxonidine 400microgram tablets	Central-acting
1080841000033119	Physiotens 200microgram tablets	Central-acting
2635741000033119	Physiotens 300microgram tablets	Central-acting
1080941000033110	Physiotens 400microgram tablets	Central-acting
11241000033113	Accuretic 10mg/12.5mg tablets	Combined
3162941000033114	Acebutolol 200mg / Hydrochlorothiazide 12.5mg tablets	Combined
13141000033119	Acezide 25mg/50mg tablets	Combined
9159341000033112	Actelsar HCT 40mg/12.5mg tablets	Combined
9159441000033118	Actelsar HCT 80mg/12.5mg tablets	Combined
9159541000033117	Actelsar HCT 80mg/25mg tablets	Combined
9159541000033116	Actelsar HCT 80mg/25mg tablets	Combined
18441000033115	Adizem-Xl Plus M/R capsules	Combined
40241000033110	Aldactide 25 tablets	Combined
40341000033117	Aldactide 50 tablets	Combined
60341000033110	Amil-Co 5mg/50mg tablets	Combined
57341000033115	Amilmaxco 5mg/50mg tablets	Combined
1903541000033116	Amiloride 2.5mg / Cyclopenthiazide 250microgram tablets	Combined
1845641000033119	Amiloride 5mg / Bumetanide 1mg tablets	Combined
4021641000033111	Amlodipine 10mg / Valsartan 160mg tablets	Combined
4021541000033110	Amlodipine 5mg / Valsartan 160mg tablets	Combined
4021441000033114	Amlodipine 5mg / Valsartan 80mg tablets	Combined

90741000033113	Atenix Co 100 tablets	Combined
92441000033119	Atenix Co 50 tablets	Combined
92341000033113	Atenixco 50 Tablets 12.5 mg	Combined
3246941000033118	Atenolol 50mg / Nifedipine 20mg modified-release capsules	Combined
124341000033119	Beta-Adalat modified-release capsules	Combined
172341000033112	Burinex A 5mg/1mg tablets	Combined
216441000033110	Capozide 25mg/50mg tablets	Combined
209241000033119	Capozide LS 12.5mg/25mg tablets	Combined
1893341000033116	Capto-Co Tablets 12.5 mg/25 mg	Combined
1893441000033110	Capto-Co Tablets 25 mg/50 mg	Combined
209841000033115	Carace 10 Plus tablets	Combined
217041000033116	Carace 20 Plus tablets	Combined
3136041000033117	Caralpha 10mg/12.5mg tablets	Combined
3136141000033118	Caralpha 20mg/12.5mg tablets	Combined
5393041000033115	Chlorothiazide And Spironolactone Capsules 250 mg + 25 mg	Combined
372841000033114	Co-Betaloc SA tablets	Combined
372741000033116	Co-Betaloc tablets	Combined
3152541000033112	Co-Diovan 160mg/12.5mg tablets	Combined
3152641000033113	Co-Diovan 160mg/25mg tablets	Combined
3190841000033119	Co-Diovan 80mg/12.5mg tablets	Combined
6028541000033112	Co-Tritace Tablets 10 mg + 12.5 mg	Combined
6028641000033113	Co-Tritace Tablets 10 mg + 25 mg	Combined
376741000033115	Co-amilofruse 10mg/80mg tablets	Combined
376841000033113	Co-amilofruse 2.5mg/20mg tablets	Combined
376641000033112	Co-amilofruse 5mg/40mg tablets	Combined
5710441000033117	Co-amilofruse 5mg/40mg/5ml oral suspension	Combined
376941000033117	Co-amilozide 2.5mg/25mg tablets	Combined
369141000033118	Co-amilozide 5mg/50mg tablets	Combined
351941000033111	Co-amilozide 5mg/50mg/5ml oral solution	Combined
377041000033116	Co-flumactone 25mg/25mg tablets	Combined
377141000033117	Co-flumactone 50mg/50mg tablets	Combined
377341000033119	Co-prenozide 160mg/0.25mg modified-release tablets	Combined
376541000033111	Co-tenidone 100mg/25mg tablets	Combined
376441000033110	Co-tenidone 50mg/12.5mg tablets	Combined
370241000033111	Co-triamterzide 50mg/25mg tablets	Combined
1893141000033119	Co-zidocapt 12.5mg/25mg tablets	Combined
1893241000033114	Co-zidocapt 25mg/50mg tablets	Combined
2216141000033115	CoAprovel 150mg/12.5mg tablets	Combined
2216241000033110	CoAprovel 300mg/12.5mg tablets	Combined
3995341000033119	CoAprovel 300mg/25mg tablets	Combined
375441000033118	Corgaretic 40mg tablets	Combined
375541000033117	Corgaretic 80mg tablets	Combined
4454641000033117	Coversyl Arginine Plus 5mg/1.25mg tablets	Combined
2739541000033117	Coversyl Plus tablets	Combined
4424841000033117	Cozaar-Comp 100mg/12.5mg tablets	Combined

3346941000033113	Cozaar-Comp 100mg/25mg tablets	Combined
370841000033110	Cozaar-Comp 50mg/12.5mg tablets	Combined
492341000033115	Dyazide 50mg/25mg tablets	Combined
491741000033113	Dytide capsules	Combined
3162241000033117	Enalapril 20mg / Hydrochlorothiazide 12.5mg tablets	Combined
4021941000033116	Exforge 10mg/160mg tablets	Combined
4021841000033112	Exforge 5mg/160mg tablets	Combined
4021741000033119	Exforge 5mg/80mg tablets	Combined
8029241000033113	Exforge Hct Tablets 5 mg/160 mg/12.5 mg	Combined
8029341000033115	Exforge Hct Tablets 5 mg/160 mg/25 mg	Combined
8027941000033110	Exforge Tablets 10 mg/320 mg	Combined
8027841000033119	Exforge Tablets 5 mg/320 mg	Combined
3163341000033119	Felodipine 2.5mg modified-release / Ramipril 2.5mg tablets	Combined
3163241000033112	Felodipine 5mg modified-release / Ramipril 5mg tablets	Combined
1611441000033117	Froop Co 5mg/40mg tablets	Combined
615241000033116	Fru-Co 5mg/40mg tablets	Combined
614641000033117	Frumil 40mg/5mg tablets	Combined
615641000033118	Frumil Forte 10mg/80mg tablets	Combined
614341000033113	Frumil LS 20mg/2.5mg tablets	Combined
615041000033112	Frusene 50mg/40mg tablets	Combined
756641000033114	Inderetic 80mg/2.5mg capsules	Combined
756741000033117	Inderex 160mg/5mg modified-release capsules	Combined
770141000033115	Innozide 20mg/12.5mg tablets	Combined
3161141000033116	Irbesartan 150mg / Hydrochlorothiazide 12.5mg tablets	Combined
3161241000033111	Irbesartan 300mg / Hydrochlorothiazide 12.5mg tablets	Combined
3995241000033112	Irbesartan 300mg / Hydrochlorothiazide 25mg tablets	Combined
796741000033111	Kalspare Ls Tablets	Combined
796541000033115	Kalspare tablets	Combined
793741000033117	Kalten capsules	Combined
808841000033119	Lasilactone 20mg/50mg capsules	Combined
816241000033119	Lasipressin Tablets	Combined
818941000033112	Lasoride 5mg/40mg tablets	Combined
3279541000033115	Lisicostad 10mg/12.5mg tablets	Combined
3279641000033119	Lisicostad 20mg/12.5mg tablets	Combined
3160841000033117	Lisinopril 10mg / Hydrochlorothiazide 12.5mg tablets	Combined
3160941000033113	Lisinopril 20mg / Hydrochlorothiazide 12.5mg tablets	Combined
849941000033112	Lopresoretic Tablets	Combined
4424741000033110	Losartan 100mg / Hydrochlorothiazide 12.5mg tablets	Combined
3346841000033117	Losartan 100mg / Hydrochlorothiazide 25mg tablets	Combined
3161741000033117	Losartan 50mg / Hydrochlorothiazide 12.5mg tablets	Combined
3161341000033118	Metoprolol 100mg / Hydrochlorothiazide 12.5mg tablets	Combined
2864241000033110	MicardisPlus 40mg/12.5mg tablets	Combined
2864341000033117	MicardisPlus 80mg/12.5mg tablets	Combined
4548841000033118	MicardisPlus 80mg/25mg tablets	Combined
935341000033118	Moducren tablets	Combined
935441000033112	Moduret 25 tablets	Combined

935541000033113	Moduretic 5mg/50mg tablets	Combined
937341000033111	Monozide 10 tablets	Combined
956741000033114	Navispare 2.5mg/250microgram tablets	Combined
5005841000033114	Olmesartan medoxomil 20mg / Amlodipine 5mg tablets	Combined
3908941000033112	Olmesartan medoxomil 20mg / Hydrochlorothiazide 12.5mg tablets	Combined
3909041000033115	Olmesartan medoxomil 20mg / Hydrochlorothiazide 25mg tablets	Combined
5006041000033111	Olmesartan medoxomil 40mg / Amlodipine 10mg tablets	Combined
5005941000033118	Olmesartan medoxomil 40mg / Amlodipine 5mg tablets	Combined
5566341000033111	Olmesartan medoxomil 40mg / Hydrochlorothiazide 12.5mg tablets	Combined
3909141000033116	Olmetec Plus 20mg/12.5mg tablets	Combined
3909241000033111	Olmetec Plus 20mg/25mg tablets	Combined
5566441000033117	Olmetec Plus 40mg/12.5mg tablets	Combined
4454541000033118	Perindopril arginine 5mg / Indapamide 1.25mg tablets	Combined
9105341000033116	Perindopril erbumine 4mg / Amlodipine 10mg tablets	Combined
9105241000033114	Perindopril erbumine 4mg / Amlodipine 5mg tablets	Combined
3161641000033114	Perindopril erbumine 4mg / Indapamide 1.25mg tablets	Combined
9105541000033111	Perindopril erbumine 8mg / Amlodipine 10mg tablets	Combined
9105541000033112	Perindopril erbumine 8mg / Amlodipine 10mg tablets	Combined
9105441000033110	Perindopril erbumine 8mg / Amlodipine 5mg tablets	Combined
8263641000033112	Perindopril tosilate 5mg / Indapamide 1.25mg tablets	Combined
1132841000033118	Prestim Forte Tablets	Combined
1132741000033111	Prestim tablets	Combined
3160641000033118	Quinapril 10mg / Hydrochlorothiazide 12.5mg tablets	Combined
1274641000033111	Secadrex 200mg/12.5mg tablets	Combined
5006141000033110	Sevikar 20mg/5mg tablets	Combined
5006341000033113	Sevikar 40mg/10mg tablets	Combined
5006241000033115	Sevikar 40mg/5mg tablets	Combined
6180641000033112	Sevikar HCT 20mg/5mg/12.5mg tablets	Combined
6180841000033113	Sevikar HCT 40mg/10mg/12.5mg tablets	Combined
6181041000033110	Sevikar HCT 40mg/10mg/25mg tablets	Combined
6180741000033115	Sevikar HCT 40mg/5mg/12.5mg tablets	Combined
6180941000033117	Sevikar HCT 40mg/5mg/25mg tablets	Combined
1366241000033116	Sotazide Tablets	Combined
1373041000033111	Spiro-Co 25 Tablets	Combined
1372941000033118	Spiro-Co 50 Tablets	Combined
9278541000033112	Spironolactone 3mg / Chlorothiazide 30mg capsules	Combined
7681541000033117	Spironolactone 4mg / Chlorothiazide 40mg capsules	Combined
3162541000033115	Spironolactone 50mg / Furosemide 20mg capsules	Combined
1374441000033117	Spiroprop Tablets	Combined
2955841000033110	Synuretic 25 tablets	Combined
2955741000033117	Synuretic 50 tablets	Combined
1406341000033117	Tarka modified-release capsules	Combined
3162641000033119	Telmisartan 40mg / Hydrochlorothiazide 12.5mg tablets	Combined
3162741000033111	Telmisartan 80mg / Hydrochlorothiazide 12.5mg tablets	Combined

4548741000033111	Telmisartan 80mg / Hydrochlorothiazide 25mg tablets	Combined
1414241000033117	Tenben 25mg/1.25mg capsules	Combined
1425541000033114	Tenchlor 100mg/25mg tablets	Combined
1425441000033113	Tenchlor 50mg/12.5mg tablets	Combined
1414941000033114	Tenif 50mg/20mg modified-release capsules	Combined
1426241000033117	Tenoret 50mg/12.5mg tablets	Combined
1426341000033110	Tenoretic 100mg/25mg tablets	Combined
5376441000033114	Timolol 10mg / Amiloride 2.5mg / Hydrochlorothiazide 25mg tablets	Combined
3346641000033118	Timolol 10mg / Bendroflumethiazide 2.5mg tablets	Combined
1451041000033115	Tolerzide Tablets	Combined
9292641000033116	Tolucombi 40mg/12.5mg tablets	Combined
9292741000033113	Tolucombi 80mg/12.5mg tablets	Combined
9292741000033112	Tolucombi 80mg/12.5mg tablets	Combined
9292841000033116	Tolucombi 80mg/25mg tablets	Combined
9292841000033115	Tolucombi 80mg/25mg tablets	Combined
1469941000033114	Trasidrex modified-release tablets	Combined
1470341000033112	Triam-Co 50mg/25mg tablets	Combined
1468141000033118	Triamaxco 50mg/25mg tablets	Combined
3161941000033119	Triamterene 50mg / Benzthiazide 25mg capsules	Combined
3162341000033110	Triamterene 50mg / Chlortalidone 50mg tablets	Combined
3092741000033119	Triamterene 50mg / Furosemide 40mg tablets	Combined
1846841000033111	Triamterene And Frusemide Tablets 50 mg + 40 mg	Combined
8264941000033119	Triapin 2.5mg/2.5mg modified-release tablets	Combined
1834341000033110	Triapin 5mg/5mg modified-release tablets	Combined
1834241000033117	Triapin Mite Tablets	Combined
3161441000033112	Valsartan 160mg / Hydrochlorothiazide 12.5mg tablets	Combined
3161541000033113	Valsartan 160mg / Hydrochlorothiazide 25mg tablets	Combined
3190741000033112	Valsartan 80mg / Hydrochlorothiazide 12.5mg tablets	Combined
1505441000033114	Vasetic 5/50 Tablets	Combined
3163041000033116	Verapamil 180mg modified-release / Trandolapril 2mg capsules	Combined
1551841000033118	Zestoretic 10 tablets	Combined
1552341000033118	Zestoretic 20 tablets	Combined
1557741000033119	Zida-Co 5/50 Tablets	Combined
40441000033111	Aldactone 100mg tablets	Diuretics
40541000033112	Aldactone 25mg tablets	Diuretics
40641000033113	Aldactone 50mg tablets	Diuretics
12312641000033118	Alkapamid XL 1.5mg tablets	Diuretics
2101641000033119	Amilamont 5mg/5ml oral solution sugar free	Diuretics
57141000033118	Amiloride 5mg tablets	Diuretics
53841000033117	Amiloride 5mg/5ml oral solution sugar free	Diuretics
61241000033112	Amilospare-5 Tablets 5 mg	Diuretics
2956041000033112	Amoride 5mg tablets	Diuretics
74541000033119	Aprinox 2.5mg tablets	Diuretics
74641000033118	Aprinox 5mg tablets	Diuretics
77541000033112	Arelix Capsules 6 mg	Diuretics

5453941000033119	Bendroflumethiazide 1.25mg/5ml oral suspension	Diuretics
3161041000033115	Bendroflumethiazide 2.5mg / Potassium chloride 573mg	Diuretics
3162841000033118	Bendroflumethiazide 2.5mg / Potassium chloride 630mg	Diuretics
3083141000033112	Bendroflumethiazide 2.5mg tablets	Diuretics
3151841000033117	Bendroflumethiazide 2.5mg/5ml oral suspension	Diuretics
3083241000033117	Bendroflumethiazide 5mg tablets	Diuretics
5401741000033110	Bendroflumethiazide 5mg/5ml oral suspension	Diuretics
141341000033119	Berkamil Tablets 5 mg	Diuretics
143741000033119	Berkozide Tablets 2.5 mg	Diuretics
143841000033112	Berkozide Tablets 5 mg	Diuretics
172441000033118	Bumetanide 1mg tablets	Diuretics
169041000033112	Bumetanide 1mg/5ml oral solution sugar free	Diuretics
3858741000033112	Bumetanide 2mg/4ml solution	Diuretics
1845741000033111	Bumetanide 500microgram / Potassium chloride 573mg	Diuretics
172541000033117	Bumetanide 5mg tablets	Diuretics
10713941000033112	Bumetanide 5mg/5ml oral suspension	Diuretics
10713941000033113	Bumetanide 5mg/5ml oral suspension	Diuretics
167341000033118	Bumetanide Injection 500 micrograms/ml	Diuretics
171941000033116	Bumetanide K Tablets	Diuretics
172741000033113	Burinex 1mg tablets	Diuretics
172841000033115	Burinex 5mg tablets	Diuretics
167541000033113	Burinex Injection 500 micrograms/ml	Diuretics
172941000033111	Burinex K modified-release tablets	Diuretics
169141000033111	Burinex Liquid 1 mg/5 ml	Diuretics
9121841000033116	Cardide SR 1.5mg tablets	Diuretics
9121841000033115	Cardide SR 1.5mg tablets	Diuretics
234841000033110	Centyl K modified-release tablets	Diuretics
234641000033114	Centyl Tablets 2.5 mg	Diuretics
234741000033117	Centyl Tablets 5 mg	Diuretics
3086241000033111	Chlortalidone 50mg tablets	Diuretics
395041000033117	Cyclopenthiazide 500microgram tablets	Diuretics
428741000033112	Delvas Tablets	Diuretics
466641000033112	Diatensec Tablets 50 mg	Diuretics
470541000033117	Diumide-K Continus tablets	Diuretics
9808241000033110	Diuresal 500mg tablets	Diuretics
450241000033116	Diuresal Injection 10 mg/ml	Diuretics
470641000033116	Diuresal Tablets 40 mg	Diuretics
470741000033113	Diurexan 20mg tablets	Diuretics
482941000033115	Dryptal Injection 20 mg/2 ml	Diuretics
483041000033113	Dryptal Injection 50 mg/5 ml	Diuretics
484741000033114	Dryptal Tablets 40 mg	Diuretics
485141000033111	Dryptal Tablets 500 mg	Diuretics
491641000033116	Dytac 50mg capsules	Diuretics
523141000033112	Enduron Tablets 5 mg	Diuretics
3198841000033117	Eplerenone 25mg tablets	Diuretics
3198941000033113	Eplerenone 50mg tablets	Diuretics
546241000033113	Esidrex K Tablets	Diuretics

546041000033117	Esidrex Tablets 25 mg	Diuretics
546141000033118	Esidrex Tablets 50 mg	Diuretics
4571241000033110	Ethibide XL 1.5mg tablets	Diuretics
615541000033119	Froop 40mg tablets	Diuretics
615141000033111	Frusid 40mg tablets	Diuretics
1731541000033115	Frusol 20mg/5ml oral solution	Diuretics
1731641000033119	Frusol 40mg/5ml oral solution	Diuretics
1731741000033111	Frusol 50mg/5ml oral solution	Diuretics
3162441000033116	Furosemide 20mg / Potassium chloride 750mg	Diuretics
3092241000033113	Furosemide 20mg tablets	Diuretics
3091841000033115	Furosemide 20mg/2ml solution	Diuretics
3091941000033111	Furosemide 20mg/5ml oral solution sugar free	Diuretics
3954441000033119	Furosemide 250mg/25ml solution	Diuretics
3161841000033110	Furosemide 40mg / Potassium chloride 600mg	Diuretics
3092341000033115	Furosemide 40mg tablets	Diuretics
3092041000033117	Furosemide 40mg/5ml oral solution sugar free	Diuretics
3092441000033114	Furosemide 500mg tablets	Diuretics
3092141000033118	Furosemide 50mg/5ml oral solution sugar free	Diuretics
3091741000033113	Furosemide 50mg/5ml solution	Diuretics
3924941000033113	Furosemide 5mg/5ml oral solution	Diuretics
5993341000033112	Furosemide 5mg/5ml oral suspension	Diuretics
3092541000033110	Furosemide 80mg/8ml solution	Diuretics
3092641000033111	Furosemide 80mg/8ml solution	Diuretics
742141000033115	Hydrenox Tablets 50 mg	Diuretics
744941000033110	Hydrochlorothiazide 25mg tablets	Diuretics
745441000033118	Hydrochlorothiazide 50mg tablets	Diuretics
2746441000033111	Hydrochlorothiazide 50mg/5ml oral solution	Diuretics
5969841000033119	Hydrochlorothiazide 5mg/5ml oral solution	Diuretics
5969941000033110	Hydrochlorothiazide 5mg/5ml oral suspension	Diuretics
745041000033110	Hydroflumethiazide Tablets 50 mg	Diuretics
742541000033112	Hydrosaluric 25mg tablets	Diuretics
742641000033113	Hydrosaluric 50mg tablets	Diuretics
742841000033114	Hygroton 50mg tablets	Diuretics
742941000033118	Hygroton K Tablets	Diuretics
742741000033116	Hygroton Tablets 100 mg	Diuretics
743041000033111	Hygroton-K Tablets	Diuretics
746141000033119	Hypertane-50 Tablets	Diuretics
765741000033116	Indapamide 1.5mg modified-release tablets	Diuretics
768641000033118	Indapamide 2.5mg tablets	Diuretics
8197041000033112	Indapamide 2.5mg/5ml oral suspension	Diuretics
5053141000033113	Indipam XL 1.5mg tablets	Diuretics
3199041000033116	Inspra 25mg tablets	Diuretics
3199141000033117	Inspra 50mg tablets	Diuretics
816141000033114	Lasikal modified-release tablets	Diuretics
816641000033116	Lasix + K Tablets	Diuretics
816341000033112	Lasix 20mg tablets	Diuretics
811841000033111	Lasix 20mg/2ml solution	Diuretics

816441000033118	Lasix 40mg tablets	Diuretics
816541000033117	Lasix 500mg tablets	Diuretics
812041000033114	Lasix 5mg/5ml oral solution	Diuretics
811941000033115	Lasix Injection 10 mg/ml	Diuretics
811141000033117	Lasix Injection 250 mg/25 ml	Diuretics
811241000033112	Lasix Injection 50 mg/5 ml	Diuretics
5128741000033110	Mapemid XL 1.5mg tablets	Diuretics
896841000033116	Metenix 5mg tablets	Diuretics
902741000033119	Methyclothiazide Tablets 5 mg	Diuretics
8196441000033118	Metolazone 2.5mg tablets	Diuretics
903541000033116	Metolazone 5mg tablets	Diuretics
898341000033114	Metolazone Tablets 500 micrograms	Diuretics
921541000033114	Midamor Tablets 5 mg	Diuretics
933541000033112	Moduretic Solution	Diuretics
955641000033113	Natramid Tablets 2.5 mg	Diuretics
955341000033117	Natrilix 2.5mg tablets	Diuretics
953141000033119	Natrilix SR 1.5mg tablets	Diuretics
955441000033111	Navidrex 500microgram tablets	Diuretics
955541000033112	Navidrex-K Tablets	Diuretics
965841000033117	Neo-Bendromax Tablets 2.5 mg	Diuretics
965941000033113	Neo-Bendromax Tablets 5 mg	Diuretics
6045641000033115	Neo-Naclex 2.5mg tablets	Diuretics
965341000033114	Neo-Naclex 5mg tablets	Diuretics
967041000033118	Neo-Naclex-K modified-release tablets	Diuretics
965441000033115	Nephril 1mg tablets	Diuretics
769741000033118	Nindaxa 2.5 tablets	Diuretics
987441000033112	Normetic Tablets	Diuretics
1009041000033117	Opumide Tablets 2.5 mg	Diuretics
1083941000033117	Piretanide Capsules 6 mg	Diuretics
1110141000033119	Polythiazide 1mg tablets	Diuretics
5566041000033114	Rawel XL 1.5mg tablets	Diuretics
1257941000033113	Saluric Tablets 500 mg	Diuretics
1373441000033119	Spiretic Tablets 100 mg	Diuretics
1373541000033118	Spiretic Tablets 25 mg	Diuretics
1367941000033110	Spiroctan Capsules 100 mg	Diuretics
1373641000033117	Spiroctan Tablets 25 mg	Diuretics
1373741000033114	Spiroctan Tablets 50 mg	Diuretics
1370541000033113	Spiroctan-M Injection 20 mg/ml	Diuretics
1373841000033116	Spirolone Tablets 100 mg	Diuretics
1373941000033112	Spirolone Tablets 25 mg	Diuretics
1374041000033114	Spirolone Tablets 50 mg	Diuretics
1374141000033113	Spironolactone 100mg tablets	Diuretics
5999141000033111	Spironolactone 100mg/5ml oral solution	Diuretics
2011541000033114	Spironolactone 100mg/5ml oral suspension	Diuretics
5999241000033116	Spironolactone 10mg/5ml oral solution	Diuretics
1371241000033116	Spironolactone 10mg/5ml oral suspension	Diuretics
5975441000033113	Spironolactone 15mg/5ml oral suspension	Diuretics

5975541000033114	Spironolactone 250mg/5ml oral solution	Diuretics
5975641000033110	Spironolactone 250mg/5ml oral suspension	Diuretics
1374241000033118	Spironolactone 25mg tablets	Diuretics
5890141000033112	Spironolactone 25mg/5ml oral solution	Diuretics
1371041000033112	Spironolactone 25mg/5ml oral suspension	Diuretics
1374341000033111	Spironolactone 50mg tablets	Diuretics
5890441000033116	Spironolactone 50mg/5ml oral solution	Diuretics
1371341000033114	Spironolactone 50mg/5ml oral suspension	Diuretics
5999341000033114	Spironolactone 5mg/5ml oral solution	Diuretics
1371141000033111	Spironolactone 5mg/5ml oral suspension	Diuretics
1368341000033110	Spironolactone Capsules 100 mg	Diuretics
1374541000033116	Spirospare 100 tablets	Diuretics
1372841000033114	Spirospare-25 Tablets 25 mg	Diuretics
4656741000033119	Tensaid XL 1.5mg tablets	Diuretics
1450141000033111	Torasemide 10mg tablets	Diuretics
1450241000033116	Torasemide 2.5mg tablets	Diuretics
1450341000033114	Torasemide 5mg tablets	Diuretics
1446341000033113	Torasemide Injection 10 mg/2 ml	Diuretics
1446441000033119	Torasemide Injection 20 mg/4 ml	Diuretics
1449841000033111	Torem 10mg tablets	Diuretics
1449941000033115	Torem 2.5mg tablets	Diuretics
1450041000033112	Torem 5mg tablets	Diuretics
1446141000033110	Torem Injection 10 mg/2 ml	Diuretics
1446241000033115	Torem Injection 20 mg/4 ml	Diuretics
1455341000033115	Triamterene 50mg capsules	Diuretics
2955941000033119	Urizide 2.5mg tablets	Diuretics
1495441000033116	Urizide 5mg tablets	Diuretics
5816741000033112	Varbim XL 1.5mg tablets	Diuretics
1543941000033111	Xipamide 20mg tablets	Diuretics
1544141000033112	Xuret Tablets 500 micrograms	Diuretics
8196541000033117	Zaroxolyn 2.5mg tablets	Diuretics
8196641000033116	Zaroxolyn 5mg tablets	Diuretics
655241000033111	Guanethidine 10mg/1ml solution	Peripheral adrenergic inhibitor
654741000033118	Guanethidine Monosulfate 1+0.2 Eye drops	Peripheral adrenergic inhibitor
654841000033111	Guanethidine Monosulfate 3+0.5 Eye drops	Peripheral adrenergic inhibitor
654641000033110	Guanethidine Monosulfate Eye drops 5 %	Peripheral adrenergic inhibitor
655541000033113	Guanethidine Monosulfate Tablets 10 mg	Peripheral adrenergic inhibitor
655641000033114	Guanethidine Monosulfate Tablets 25 mg	Peripheral adrenergic inhibitor
780741000033110	Ismelin 10mg/1ml solution	Peripheral adrenergic inhibitor
1164941000033114	Reserpine Tablets 0.1 mg	Peripheral adrenergic inhibitor

1165041000033114	Reserpine Tablets 0.25 mg	Peripheral adrenergic inhibitor
1276541000033115	Serpasil Esidrex Tablets	Peripheral adrenergic inhibitor
1276341000033110	Serpasil Tablets 0.1 mg	Peripheral adrenergic inhibitor
1276441000033116	Serpasil Tablets 0.25 mg	Peripheral adrenergic inhibitor
73441000033117	Apresoline 20mg powder	Vasodilators
74341000033114	Apresoline 25mg tablets	Vasodilators
74441000033115	Apresoline Tablets 50 mg	Vasodilators
6468341000033117	Diazoxide 100mg/5ml oral solution	Vasodilators
6468441000033111	Diazoxide 100mg/5ml oral suspension	Vasodilators
5966941000033118	Diazoxide 10mg/5ml oral solution	Vasodilators
5966841000033114	Diazoxide 10mg/5ml oral suspension	Vasodilators
8182141000033114	Diazoxide 12.5mg/5ml oral suspension	Vasodilators
2623841000033114	Diazoxide 250mg/5ml oral suspension	Vasodilators
448841000033117	Diazoxide 300mg/20ml solution	Vasodilators
464441000033117	Diazoxide 50mg tablets	Vasodilators
9173841000033115	Diazoxide 50mg/5ml oral suspension	Vasodilators
4119841000033113	Diazoxide Oral Suspension	Vasodilators
555141000033110	Eudemine 300mg/20ml solution	Vasodilators
556641000033113	Eudemine 50mg tablets	Vasodilators
4456241000033111	Hydralazine 10mg/5ml oral suspension	Vasodilators
734141000033114	Hydralazine 20mg powder for solution	Vasodilators
741941000033113	Hydralazine 25mg tablets	Vasodilators
11072741000033116	Hydralazine 25mg/5ml oral solution	Vasodilators
742041000033119	Hydralazine 50mg tablets	Vasodilators
3940641000033114	Hydralazine Hydrochloride Dispersible tablets 10 mg	Vasodilators
1618441000033111	Hydralazine Hydrochloride Tablets 12.5 mg	Vasodilators
850441000033112	Loniten 10mg tablets	Vasodilators
850541000033113	Loniten 2.5mg tablets	Vasodilators
850641000033114	Loniten 5mg tablets	Vasodilators
920641000033119	Minoxidil 10mg tablets	Vasodilators
920841000033118	Minoxidil 2.5mg tablets	Vasodilators
920941000033114	Minoxidil 5mg tablets	Vasodilators
969341000033112	Nipride Infusion 50 mg	Vasodilators
7859441000033119	Proglycem 250mg/5ml oral suspension	Vasodilators
1357141000033115	Sodium nitroprusside 50mg powder	Vasodilators

Carr MJ, *et al. BMJ Qual Saf* 2022; 31:503–514. doi: 10.1136/bmjqs-2021-013613

Appendix 10. Product codes for prescriptions of lipid-lowering medications

Product code	Term	Drug class
2906741000033119	Ezetimibe 10mg tablets	Ezetimibe
2906841000033112	Ezetrol 10mg tablets (Merck Sharp & Dohme Ltd)	Ezetimibe
145841000033113	Bezafibrate 200mg tablets	Fibrate
145941000033117	Bezafibrate 400mg modified-release tablets	Fibrate
1578041000033115	Bezagen XL 400mg tablets (Mylan)	Fibrate
144941000033118	Bezalip 200mg tablets (Teva UK Ltd)	Fibrate
145041000033118	Bezalip Mono 400mg modified-release tablets (Teva UK Ltd)	Fibrate
258541000033118	Ciprofibrate 100mg tablets	Fibrate
564541000033112	Fenofibrate 200mg capsules	Fibrate
565741000033115	Fenofibrate Capsules 100 mg	Fibrate
2214741000033113	Fenofibrate micronised 160mg tablets	Fibrate
3188941000033114	Fenofibrate micronised 200mg capsules	Fibrate
1862541000033115	Fenofibrate micronised 267mg capsules	Fibrate
564641000033113	Fenofibrate micronised 67mg capsules	Fibrate
2801141000033116	Fenogal 200mg capsules (Genus Pharmaceuticals Ltd)	Fibrate
6453641000033112	Fibrazate XL 400mg tablets (Sandoz Ltd)	Fibrate
627841000033118	Gemfibrozil 300mg capsules	Fibrate
634541000033110	Gemfibrozil 600mg tablets	Fibrate
828041000033118	Lipantil Capsules 100 mg	Fibrate
1862741000033111	Lipantil Micro 200 capsules (Mylan)	Fibrate
1862641000033119	Lipantil Micro 267 capsules (Mylan)	Fibrate
836941000033116	Lipantil Micro 67 capsules (Mylan)	Fibrate
827441000033114	Lipantil Micro Capsules 200 mg	Fibrate
2055241000033119	Liparol 400 XL tablets (Ashbourne Pharmaceuticals Ltd)	Fibrate
838941000033115	Lopid 300mg capsules (Pfizer Ltd)	Fibrate
851541000033119	Lopid 600mg tablets (Pfizer Ltd)	Fibrate
936941000033113	Modalim 100mg tablets (Lexon (UK) Ltd)	Fibrate
2214841000033115	Supralip 160mg tablets (Mylan)	Fibrate
2192041000033117	Zimbacol XL 400mg tablets (Archimedes Pharma UK Ltd)	Fibrate
2667641000033114	*Nicotinic Acid Granules	Other
2667541000033113	*Nicotinic Acid Liquid	Other
2667841000033110	*Nicotinic Acid Pills (Sucrose)	Other
2667741000033117	*Nicotinic Acid Powder	Other
2667441000033112	*Nicotinic Acid Tablets	Other
2841000033117	Acipimox 250mg capsules	Other
10647141000033116	Alirocumab 150mg/1ml solution for injection pre-filled disposable devices	Other
10647041000033115	Alirocumab 75mg/1ml solution for injection pre-filled disposable devices	Other
4310941000033117	Cholestagel 625mg tablets (Sanofi)	Other
244041000033116	Cholestyramine Powder 4g	Other
2908141000033117	Cholestyramine Sugar Free Powder 4 grams/sachet	Other
4310841000033113	Colesevelam 625mg tablets	Other
328341000033119	Colestid 5g granules sachets plain (Pfizer Ltd)	Other

359441000033119	Colestid Orange 5g granules sachets (Pfizer Ltd)	Other
12688241000033113	Colestipol 1g tablets	Other
328141000033117	Colestipol 5g granules sachets sugar free	Other
359541000033118	Colestipol Hydrochloride Sachets (orange) 5 grams/sachet	Other
3086341000033118	Colestyramine 4g oral powder sachets	Other
3086441000033112	Colestyramine 4g oral powder sachets sugar free	Other
10213541000033113	Dualtis 1000mg capsules (Mylan)	Other
3281941000033115	Eicosapentaenoic acid 170mg / Docosahexaenoic acid 115mg capsules	Other
3282041000033114	Eicosapentaenoic acid 170mg/g / Docosahexaenoic acid 115mg/g oral liquid	Other
3282141000033113	Eicosapentaenoic acid 460mg / Docosahexaenoic acid 380mg capsules	Other
10618041000033112	Evolocumab 140mg/1ml solution for injection pre-filled disposable devices	Other
10617841000033118	Evolocumab 140mg/1ml solution for injection pre-filled syringes	Other
8886541000033111	Lojuxta 10mg capsules (Amryt Pharma)	Other
8886641000033112	Lojuxta 20mg capsules (Amryt Pharma)	Other
8886441000033110	Lojuxta 5mg capsules (Amryt Pharma)	Other
8886241000033114	Lomitapide 10mg capsules	Other
8886341000033116	Lomitapide 20mg capsules	Other
8886141000033119	Lomitapide 5mg capsules	Other
858241000033113	MaxEPA 1g capsules (Seven Seas Ltd)	Other
863241000033112	MaxEPA liquid (Seven Seas Ltd)	Other
859141000033112	Maxepa Emulsion	Other
9118541000033115	Nebbaro 1000mg capsules (Zentiva)	Other
2994441000033118	Niaspan 1g modified-release tablets (Abbott Laboratories Ltd)	Other
2994241000033119	Niaspan 500mg modified-release tablets (Abbott Laboratories Ltd)	Other
2994341000033112	Niaspan 750mg modified-release tablets (Abbott Laboratories Ltd)	Other
2994641000033116	Niaspan titration pack (Abbott Laboratories Ltd)	Other
974641000033112	Nicotinic Acid Tablets 100 mg	Other
974741000033115	Nicotinic Acid Tablets 25 mg	Other
2994541000033117	Nicotinic Acid Titration Starter Pack M/R tablets 7 x 375 mg, 7 x 500 mg, 7 x 750 mg	Other
4805841000033118	Nicotinic acid 1g / Laropiprant 20mg modified-release tablets	Other
2994141000033114	Nicotinic acid 1g modified-release tablets	Other
2849841000033117	Nicotinic acid 500mg modified-release tablets	Other
974841000033113	Nicotinic acid 50mg tablets	Other
2994041000033110	Nicotinic acid 750mg modified-release tablets	Other
1003341000033117	Olbetam 250mg capsules (Pfizer Ltd)	Other
2762241000033115	Omacor capsules (Mylan)	Other
10647341000033118	Praluent 150mg/1ml solution for injection pre-filled pens (Sanofi)	Other
10647241000033111	Praluent 75mg/1ml solution for injection pre-filled pens (Sanofi)	Other
8275241000033118	Prestylon 1g capsules (Teva UK Ltd)	Other
1148741000033114	Questran 4g oral powder sachets (Cheplapharm Arzneimittel GmbH)	Other
1148841000033116	Questran Light 4g oral powder sachets (Cheplapharm Arzneimittel GmbH)	Other
10617941000033114	Repatha 140mg/1ml solution for injection pre-filled syringes (Amgen	Other

	Ltd)	
10618141000033111	Repatha SureClick 140mg/1ml solution for injection pre-filled pens (Amgen Ltd)	Other
8127541000033114	Teromeg 1000mg capsules (Advanz Pharma)	Other
5244841000033110	Tredaptive 1000mg/20mg modified-release tablets (Merck Sharp & Dohme Ltd)	Other
6468641000033113	Atorvastatin 10mg chewable tablets sugar free	Statin
91941000033117	Atorvastatin 10mg tablets	Statin
5897841000033117	Atorvastatin 10mg/5ml oral solution	Statin
5897741000033110	Atorvastatin 10mg/5ml oral suspension	Statin
6468841000033114	Atorvastatin 20mg chewable tablets sugar free	Statin
92041000033111	Atorvastatin 20mg tablets	Statin
5990541000033112	Atorvastatin 20mg/5ml oral solution	Statin
3868741000033118	Atorvastatin 20mg/5ml oral suspension	Statin
7861441000033110	Atorvastatin 30mg tablets	Statin
92141000033110	Atorvastatin 40mg tablets	Statin
7861541000033111	Atorvastatin 60mg tablets	Statin
2261041000033114	Atorvastatin 80mg tablets	Statin
233741000033115	Cerivastatin 100microgram tablets	Statin
233841000033113	Cerivastatin 200microgram tablets	Statin
233941000033117	Cerivastatin 300microgram tablets	Statin
1916841000033114	Cerivastatin 400microgram tablets	Statin
2290041000033115	Cerivastatin 800microgram tablets	Statin
12486541000033113	Cholesterol 2% / Simvastatin 2% cream	Statin
2891841000033113	Crestor 10mg tablets (AstraZeneca UK Ltd)	Statin
2891941000033117	Crestor 20mg tablets (AstraZeneca UK Ltd)	Statin
2892041000033111	Crestor 40mg tablets (AstraZeneca UK Ltd)	Statin
3836141000033113	Crestor 5mg tablets (AstraZeneca UK Ltd)	Statin
5711841000033112	Dorisin XL 80mg tablets (Aspire Pharma Ltd)	Statin
578641000033114	Fluvastatin 20mg capsules	Statin
578741000033117	Fluvastatin 40mg capsules	Statin
2189041000033110	Fluvastatin 80mg modified-release tablets	Statin
819941000033119	Lescol 20mg capsules (Novartis Pharmaceuticals UK Ltd)	Statin
820041000033116	Lescol 40mg capsules (Novartis Pharmaceuticals UK Ltd)	Statin
2189141000033114	Lescol XL 80mg tablets (Novartis Pharmaceuticals UK Ltd)	Statin
6469041000033110	Lipitor 10mg chewable tablets (Upjohn UK Ltd)	Statin
836241000033113	Lipitor 10mg tablets (Upjohn UK Ltd)	Statin
6469141000033114	Lipitor 20mg chewable tablets (Upjohn UK Ltd)	Statin
836341000033115	Lipitor 20mg tablets (Upjohn UK Ltd)	Statin
836441000033114	Lipitor 40mg tablets (Upjohn UK Ltd)	Statin
2261141000033113	Lipitor 80mg tablets (Upjohn UK Ltd)	Statin
836641000033111	Lipobay 100microgram tablets (Bayer Plc)	Statin
836741000033119	Lipobay 200microgram tablets (Bayer Plc)	Statin
836841000033112	Lipobay 300microgram tablets (Bayer Plc)	Statin
1916741000033116	Lipobay 400microgram tablets (Bayer Plc)	Statin
2290141000033116	Lipobay 800microgram tablets (Bayer Plc)	Statin
834841000033118	Lipostat 10mg tablets (Bristol-Myers Squibb Pharmaceuticals Ltd)	Statin

Carr MJ, et al. BMJ Qual Saf 2022; 31:503–514. doi: 10.1136/bmjqs-2021-013613

834941000033114	Lipostat 20mg tablets (Bristol-Myers Squibb Pharmaceuticals Ltd)	Statin
836541000033110	Lipostat 40mg tablets (Bristol-Myers Squibb Pharmaceuticals Ltd)	Statin
5007141000033112	Luvinsta XL 80mg tablets (Actavis UK Ltd)	Statin
8962441000033117	Nandovar XL 80mg tablets (Sandoz Ltd)	Statin
5560541000033119	Pinmactil 80mg modified-release tablets (Mylan)	Statin
1130141000033111	Pravastatin 10mg tablets	Statin
1130241000033116	Pravastatin 20mg tablets	Statin
1136541000033116	Pravastatin 40mg tablets	Statin
5711041000033117	Pravastatin 40mg/5ml oral suspension	Statin
8493841000033111	Pravastatin 5mg/5ml oral solution	Statin
10618641000033118	Pravastatin 5mg/5ml oral suspension	Statin
3140841000033113	Ranzolont 10mg tablets (Ranbaxy (UK) Ltd)	Statin
3140941000033117	Ranzolont 20mg tablets (Ranbaxy (UK) Ltd)	Statin
3141041000033110	Ranzolont 40mg tablets (Ranbaxy (UK) Ltd)	Statin
2891541000033111	Rosuvastatin 10mg tablets	Statin
2891641000033112	Rosuvastatin 20mg tablets	Statin
2891741000033115	Rosuvastatin 40mg tablets	Statin
3836041000033114	Rosuvastatin 5mg tablets	Statin
2973941000033116	Simvador 10mg tablets (Dexcel-Pharma Ltd)	Statin
2974041000033119	Simvador 20mg tablets (Dexcel-Pharma Ltd)	Statin
2974141000033115	Simvador 40mg tablets (Dexcel-Pharma Ltd)	Statin
4941641000033116	Simvador 80mg tablets (Dexcel-Pharma Ltd)	Statin
1336841000033110	Simvastatin 10mg tablets	Statin
1336941000033119	Simvastatin 20mg tablets	Statin
5888441000033115	Simvastatin 20mg/5ml oral solution	Statin
3304141000033110	Simvastatin 20mg/5ml oral suspension	Statin
5817741000033114	Simvastatin 20mg/5ml oral suspension sugar free	Statin
1337541000033111	Simvastatin 40mg tablets	Statin
5888541000033119	Simvastatin 40mg/5ml oral solution	Statin
4152341000033115	Simvastatin 40mg/5ml oral suspension	Statin
5817841000033116	Simvastatin 40mg/5ml oral suspension sugar free	Statin
2066241000033118	Simvastatin 80mg tablets	Statin
5808641000033119	Stefluvin XL 80mg tablets (Zentiva)	Statin
1562841000033119	Zocor 10mg tablets (Merck Sharp & Dohme Ltd)	Statin
1562941000033110	Zocor 20mg tablets (Merck Sharp & Dohme Ltd)	Statin
1562241000033118	Zocor 40mg tablets (Merck Sharp & Dohme Ltd)	Statin
2066341000033111	Zocor 80mg tablets (Merck Sharp & Dohme Ltd)	Statin
3292741000033111	Inegy 10mg/20mg tablets (Merck Sharp & Dohme Ltd)	Statin/Ezetimibe
3292841000033118	Inegy 10mg/40mg tablets (Merck Sharp & Dohme Ltd)	Statin/Ezetimibe
3292941000033114	Inegy 10mg/80mg tablets (Merck Sharp & Dohme Ltd)	Statin/Ezetimibe
3292441000033116	Simvastatin 20mg / Ezetimibe 10mg tablets	Statin/Ezetimibe
3292541000033115	Simvastatin 40mg / Ezetimibe 10mg tablets	Statin/Ezetimibe
3292641000033119	Simvastatin 80mg / Ezetimibe 10mg tablets	Statin/Ezetimibe
11241941000033116	Cholib 145mg/20mg tablets (Mylan)	Statin/Fibrate
11242041000033110	Cholib 145mg/40mg tablets (Mylan)	Statin/Fibrate
11241741000033119	Fenofibrate 145mg / Simvastatin 20mg tablets	Statin/Fibrate
11241841000033112	Fenofibrate 145mg / Simvastatin 40mg tablets	Statin/Fibrate

Appendix 11. Product codes for prescriptions of antiplatelet medications

Product code	Term	Drug class
71241000033119	Angettes 75 tablets (Bristol-Myers Squibb Pharmaceuticals Ltd)	Aspirin
86841000033118	Aspav dispersible tablets (Actavis UK Ltd)	Aspirin
3162141000033112	Aspirin 150mg / Isosorbide mononitrate 60mg modified-release tablets	Aspirin
2013441000033114	Aspirin 150mg suppositories	Aspirin
82841000033117	Aspirin 300mg dispersible tablets	Aspirin
83141000033116	Aspirin 300mg gastro-resistant tablets	Aspirin
84441000033114	Aspirin 300mg modified-release tablets	Aspirin
2013341000033115	Aspirin 300mg suppositories	Aspirin
86641000033119	Aspirin 300mg tablets	Aspirin
3934341000033112	Aspirin 500mg / Codeine 8mg dispersible tablets sugar free	Aspirin
3849841000033110	Aspirin 500mg / Papaveretum 7.71mg dispersible tablets sugar free	Aspirin
3162041000033113	Aspirin 75mg / Isosorbide mononitrate 60mg modified-release tablets	Aspirin
82941000033113	Aspirin 75mg dispersible tablets	Aspirin
83041000033115	Aspirin 75mg gastro-resistant tablets	Aspirin
87041000033110	Aspirin 75mg tablets	Aspirin
3836841000033119	Aspirin 900mg / Metoclopramide 10mg oral powder sachets sugar free	Aspirin
5331041000033113	Aspirin And Vitamin C Effervescent tablets 330 mg + 200 mg	Aspirin
82641000033118	Aspirin Chewable tablets 227 mg	Aspirin
87141000033114	Aspirin Dispersible Low Dose Tablets 75 mg	Aspirin
85741000033118	Aspirin E/C Tablets 300 mg	Aspirin
88041000033114	Aspirin E/C Tablets 600 mg	Aspirin
84941000033116	Aspirin E/c tablets 324 mg	Aspirin
83241000033111	Aspirin E/c tablets 600 mg	Aspirin
84341000033115	Aspirin M/R tablets 100 mg	Aspirin
86941000033114	Aspirin Paracetamol And Codeine Tablets	Aspirin
4043341000033119	Aspirin Powder Sachets Lactose Free 75 mg	Aspirin
5151341000033116	Aspirin Protect E/c tablets 100 mg	Aspirin
87441000033118	Aspirin Tablets 500 mg	Aspirin
87541000033117	Aspirin Tablets 600 mg	Aspirin
2085241000033111	Aspirin powder	Aspirin
4431841000033118	Aspro Clear 300mg effervescent tablets (Bayer Plc)	Aspirin
10025241000033112	Bisoprolol 10mg / Aspirin 100mg capsules	Aspirin
10025141000033117	Bisoprolol 10mg / Aspirin 75mg capsules	Aspirin
10025041000033116	Bisoprolol 5mg / Aspirin 100mg capsules	Aspirin
10024941000033116	Bisoprolol 5mg / Aspirin 75mg capsules	Aspirin
212341000033117	Caprin 300mg gastro-resistant tablets (Pinewood Healthcare)	Aspirin
189841000033119	Caprin 75mg gastro-resistant tablets (Wockhardt UK Ltd)	Aspirin
371041000033112	Co-codaprin 8mg/400mg dispersible tablets	Aspirin
370941000033119	Co-codaprin 8mg/400mg tablets	Aspirin
3934441000033118	Codis 500 dispersible tablets (Reckitt Benckiser Healthcare (UK) Ltd)	Aspirin
11716941000033112	Danamep 75mg dispersible tablets (Ecogen Europe Ltd)	Aspirin
452541000033115	Disprin CV 300mg modified-release tablets (Reckitt Benckiser Healthcare (UK) Ltd)	Aspirin

452441000033116	Disprin Cv M/R tablets 100 mg	Aspirin
1729141000033110	Enprin 75mg gastro-resistant tablets (Galpharm International Ltd)	Aspirin
1739641000033115	Imazin XL forte tablets (Napp Pharmaceuticals Ltd)	Aspirin
1739541000033116	Imazin XL tablets (Napp Pharmaceuticals Ltd)	Aspirin
818541000033118	Laboprin Dl Tablets	Aspirin
12684641000033115	Mandaprin 75mg dispersible tablets (M & A Pharmachem Ltd)	Aspirin
4431941000033114	Maximum Strength Aspro Clear 500mg effervescent tablets (Bayer Plc)	Aspirin
2273741000033118	Micropirin 75mg gastro-resistant tablets (Dexcel-Pharma Ltd)	Aspirin
2086841000033117	MigraMax oral powder sachets (Zentiva)	Aspirin
993341000033113	Nu-Seals 300 gastro-resistant tablets (Alliance Pharmaceuticals Ltd)	Aspirin
991441000033115	Nu-Seals 75 gastro-resistant tablets (Alliance Pharmaceuticals Ltd)	Aspirin
993441000033119	Nu-Seals Aspirin E/c tablets 600 mg	Aspirin
1041641000033115	Paynocil Tablets	Aspirin
1101541000033119	PostMI 75 EC tablets (Ashbourne Pharmaceuticals Ltd)	Aspirin
1098041000033111	PostMI 75 dispersible tablets (Ashbourne Pharmaceuticals Ltd)	Aspirin
1101441000033115	Postmi 300 E/c tablets 300 mg	Aspirin
1367341000033111	Solprin Tablets 300 mg	Aspirin
1365441000033112	Soluble Aspirin Paediatric Tablets 75 mg	Aspirin
1365341000033118	Soluble Aspirin Tablets 300 mg	Aspirin
1571541000033117	Asasantin Retard capsules (Boehringer Ingelheim Ltd)	Aspirin/Other
12352041000033117	Atransipar 200mg/25mg modified-release capsules (Par Laboratories Europe Ltd)	Aspirin/Other
3160741000033110	Dipyridamole 200mg modified-release / Aspirin 25mg capsules	Aspirin/Other
8619141000033116	Molita 200mg/25mg modified-release capsules (Dr Reddy's Laboratories (UK) Ltd)	Aspirin/Other
8031441000033112	Clopidogrel 1mg/ml oral suspension	Clopidogrel
4519641000033119	Clopidogrel 300mg tablets	Clopidogrel
8300741000033118	Clopidogrel 4mg oral powder sachets	Clopidogrel
1583541000033113	Clopidogrel 75mg tablets	Clopidogrel
8301241000033117	Clopidogrel 75mg/5ml oral solution	Clopidogrel
6122841000033118	Clopidogrel 75mg/5ml oral suspension	Clopidogrel
5242341000033112	Grepid 75mg tablets (Kent Pharmaceuticals Ltd)	Clopidogrel
4519741000033111	Plavix 300mg tablets (Sanofi)	Clopidogrel
1667041000033119	Plavix 75mg tablets (Sanofi)	Clopidogrel
1941000033118	Abciximab 10mg/5ml solution for injection vials	Other
2677141000033112	Aggrastat 12.5mg/250ml infusion bags (Correvio UK Ltd)	Other
4129741000033110	Aggrastat 12.5mg/50ml concentrate for solution for infusion vials (Correvio UK Ltd)	Other
1863441000033113	Aggrastat Injection 250 micrograms/ml	Other
9165341000033117	Attia 200mg modified-release capsules (Dr Reddy's Laboratories (UK) Ltd)	Other
11422741000033114	Brilique 60mg tablets (AstraZeneca UK Ltd)	Other
12371141000033111	Brilique 90mg orodispersible tablets (AstraZeneca UK Ltd)	Other
6059441000033117	Brilique 90mg tablets (AstraZeneca UK Ltd)	Other
10602141000033112	Cangrelor 50mg powder for solution for injection vials	Other
469641000033119	Dipyridamole 100mg tablets	Other
5889941000033115	Dipyridamole 100mg/5ml oral solution	Other

5052341000033115	Dipyridamole 100mg/5ml oral suspension	Other
447141000033112	Dipyridamole 10mg/2ml solution for injection ampoules	Other
454141000033119	Dipyridamole 200mg modified-release capsules	Other
5992041000033118	Dipyridamole 200mg/5ml oral solution	Other
5020341000033117	Dipyridamole 200mg/5ml oral suspension	Other
11072641000033113	Dipyridamole 200mg/5ml oral suspension sugar free	Other
5401541000033119	Dipyridamole 250mg/5ml oral solution	Other
469741000033111	Dipyridamole 25mg tablets	Other
1728341000033118	Dipyridamole 50mg/5ml oral suspension sugar free	Other
1602841000033113	Dipyridamole Sugar-free suspension 100 mg/5 ml	Other
4956741000033115	Efient 10mg tablets (Daiichi Sankyo UK Ltd)	Other
4956641000033112	Efient 5mg tablets (Daiichi Sankyo UK Ltd)	Other
1843641000033115	Eptifibatide 20mg/10ml solution for injection vials	Other
1843741000033112	Eptifibatide 75mg/100ml solution for infusion vials	Other
1843841000033119	Integrilin 20mg/10ml solution for injection vials (GlaxoSmithKline UK Ltd)	Other
1843941000033110	Integrilin 75mg/100ml solution for infusion vials (GlaxoSmithKline UK Ltd)	Other
10602241000033117	Kengrexal 50mg powder for concentrate for solution for injection / infusion vials (Chiesi Ltd)	Other
10333041000033118	Ofcram PR 200mg capsules (Advanz Pharma)	Other
1065441000033113	Persantin 100mg tablets (Boehringer Ingelheim Ltd)	Other
1052141000033119	Persantin 10mg/2ml solution for injection ampoules (Boehringer Ingelheim Ltd)	Other
1065541000033114	Persantin 25mg tablets (Boehringer Ingelheim Ltd)	Other
1054941000033114	Persantin Retard 200mg capsules (Boehringer Ingelheim Ltd)	Other
4956541000033111	Prasugrel 10mg tablets	Other
4956441000033110	Prasugrel 5mg tablets	Other
2956241000033116	Pyridantin 100mg tablets (Dr Reddy's Laboratories (UK) Ltd)	Other
2956141000033111	Pyridantin 25mg tablets (Dr Reddy's Laboratories (UK) Ltd)	Other
1159041000033118	ReoPro 10mg/5ml solution for injection vials (Janssen-Cilag Ltd)	Other
11422441000033119	Ticagrelor 60mg tablets	Other
12371041000033112	Ticagrelor 90mg orodispersible tablets sugar free	Other
6059341000033111	Ticagrelor 90mg tablets	Other
2677041000033113	Tirofiban 12.5mg/250ml infusion bags	Other
4129641000033118	Tirofiban 12.5mg/50ml solution for infusion vials	Other
1863341000033119	Tirofiban Injection 250 micrograms/ml	Other
12195441000033115	Trolactin 200mg modified-release capsules (Actavis UK Ltd)	Other

Carr MJ, *et al. BMJ Qual Saf* 2022; 31:503–514. doi: 10.1136/bmjqs-2021-013613

Part B1. Read codes used to interrogate CPRD *GOLD*

Appendix 12. Read codes for diagnoses of type 2 diabetes

Read code	Term
C100112	Non-insulin dependent diabetes mellitus
C107400	NIDDM with peripheral circulatory disorder
C109.00	Non-insulin dependent diabetes mellitus
C109.11	NIDDM - Non-insulin dependent diabetes mellitus
C109.12	Type 2 diabetes mellitus
C109.13	Type II diabetes mellitus
C109000	Non-insulin-dependent diabetes mellitus with renal comps
C109011	Type II diabetes mellitus with renal complications
C109012	Type 2 diabetes mellitus with renal complications
C109100	Non-insulin-dependent diabetes mellitus with ophthalm comps
C109111	Type II diabetes mellitus with ophthalmic complications
C109112	Type 2 diabetes mellitus with ophthalmic complications
C109200	Non-insulin-dependent diabetes mellitus with neuro comps
C109211	Type II diabetes mellitus with neurological complications
C109212	Type 2 diabetes mellitus with neurological complications
C109300	Non-insulin-dependent diabetes mellitus with multiple comps
C109312	Type 2 diabetes mellitus with multiple complications
C109400	Non-insulin dependent diabetes mellitus with ulcer
C109411	Type II diabetes mellitus with ulcer
C109412	Type 2 diabetes mellitus with ulcer
C109500	Non-insulin dependent diabetes mellitus with gangrene
C109511	Type II diabetes mellitus with gangrene
C109512	Type 2 diabetes mellitus with gangrene
C109600	Non-insulin-dependent diabetes mellitus with retinopathy
C109611	Type II diabetes mellitus with retinopathy
C109612	Type 2 diabetes mellitus with retinopathy
C109700	Non-insulin dependent diabetes mellitus - poor control
C109711	Type II diabetes mellitus - poor control
C109712	Type 2 diabetes mellitus - poor control
C109900	Non-insulin-dependent diabetes mellitus without complication
C109911	Type II diabetes mellitus without complication
C109912	Type 2 diabetes mellitus without complication
C109A00	Non-insulin dependent diabetes mellitus with mononeuropathy
C109A11	Type II diabetes mellitus with mononeuropathy
C109B00	Non-insulin dependent diabetes mellitus with polyneuropathy
C109B11	Type II diabetes mellitus with polyneuropathy
C109B12	Type 2 diabetes mellitus with polyneuropathy
C109C00	Non-insulin dependent diabetes mellitus with nephropathy
C109C11	Type II diabetes mellitus with nephropathy
C109C12	Type 2 diabetes mellitus with nephropathy
C109D00	Non-insulin dependent diabetes mellitus with hypoglyca coma
C109D11	Type II diabetes mellitus with hypoglycaemic coma

Carr MJ, *et al. BMJ Qual Saf* 2022; 31:503–514. doi: 10.1136/bmjqs-2021-013613

C109D12	Type 2 diabetes mellitus with hypoglycaemic coma
C109E00	Non-insulin depend diabetes mellitus with diabetic cataract
C109E11	Type II diabetes mellitus with diabetic cataract
C109E12	Type 2 diabetes mellitus with diabetic cataract
C109F00	Non-insulin-dependent d m with peripheral angiopath
C109F11	Type II diabetes mellitus with peripheral angiopathy
C109F12	Type 2 diabetes mellitus with peripheral angiopathy
C109G00	Non-insulin dependent diabetes mellitus with arthropathy
C109G11	Type II diabetes mellitus with arthropathy
C109G12	Type 2 diabetes mellitus with arthropathy
C109H00	Non-insulin dependent d m with neuropathic arthropathy
C109H11	Type II diabetes mellitus with neuropathic arthropathy
C109H12	Type 2 diabetes mellitus with neuropathic arthropathy
C109J00	Insulin treated Type 2 diabetes mellitus
C109J11	Insulin treated non-insulin dependent diabetes mellitus
C109J12	Insulin treated Type II diabetes mellitus
C109K00	Hyperosmolar non-ketotic state in type 2 diabetes mellitus
C10F.00	Type 2 diabetes mellitus
C10F.11	Type II diabetes mellitus
C10F000	Type 2 diabetes mellitus with renal complications
C10F011	Type II diabetes mellitus with renal complications
C10F100	Type 2 diabetes mellitus with ophthalmic complications
C10F111	Type II diabetes mellitus with ophthalmic complications
C10F200	Type 2 diabetes mellitus with neurological complications
C10F211	Type II diabetes mellitus with neurological complications
C10F300	Type 2 diabetes mellitus with multiple complications
C10F311	Type II diabetes mellitus with multiple complications
C10F400	Type 2 diabetes mellitus with ulcer
C10F411	Type II diabetes mellitus with ulcer
C10F500	Type 2 diabetes mellitus with gangrene
C10F511	Type II diabetes mellitus with gangrene
C10F600	Type 2 diabetes mellitus with retinopathy
C10F611	Type II diabetes mellitus with retinopathy
C10F700	Type 2 diabetes mellitus - poor control
C10F711	Type II diabetes mellitus - poor control
C10F900	Type 2 diabetes mellitus without complication
C10F911	Type II diabetes mellitus without complication
C10FA00	Type 2 diabetes mellitus with mononeuropathy
C10FA11	Type II diabetes mellitus with mononeuropathy
C10FB00	Type 2 diabetes mellitus with polyneuropathy
C10FB11	Type II diabetes mellitus with polyneuropathy
C10FC00	Type 2 diabetes mellitus with nephropathy
C10FC11	Type II diabetes mellitus with nephropathy
C10FD00	Type 2 diabetes mellitus with hypoglycaemic coma
C10FD11	Type II diabetes mellitus with hypoglycaemic coma
C10FE00	Type 2 diabetes mellitus with diabetic cataract
C10FE11	Type II diabetes mellitus with diabetic cataract

C10FF00	Type 2 diabetes mellitus with peripheral angiopathy
C10FF11	Type II diabetes mellitus with peripheral angiopathy
C10FG00	Type 2 diabetes mellitus with arthropathy
C10FG11	Type II diabetes mellitus with arthropathy
C10FH00	Type 2 diabetes mellitus with neuropathic arthropathy
C10FH11	Type II diabetes mellitus with neuropathic arthropathy
C10FJ00	Insulin treated Type 2 diabetes mellitus
C10FJ11	Insulin treated Type II diabetes mellitus
C10FK00	Hyperosmolar non-ketotic state in type 2 diabetes mellitus
C10FK11	Hyperosmolar non-ketotic state in type II diabetes mellitus
C10FL00	Type 2 diabetes mellitus with persistent proteinuria
C10FL11	Type II diabetes mellitus with persistent proteinuria
C10FM00	Type 2 diabetes mellitus with persistent microalbuminuria
C10FM11	Type II diabetes mellitus with persistent microalbuminuria
C10FN00	Type 2 diabetes mellitus with ketoacidosis
C10FN11	Type II diabetes mellitus with ketoacidosis
C10FP00	Type 2 diabetes mellitus with ketoacidotic coma
C10FP11	Type II diabetes mellitus with ketoacidotic coma
C10FQ00	Type 2 diabetes mellitus with exudative maculopathy
C10FQ11	Type II diabetes mellitus with exudative maculopathy
C10FR00	Type 2 diabetes mellitus with gastroparesis

Appendix 13. Read codes for haemoglobin A1c monitoring

Read code	Term
423..00	Haemoglobin estimation
423..11	Hb estimation
42V2.00	Haemoglobin A
42V2000	Haemoglobin A quantitation
42VA.00	Haemoglobin variant test
42W..00	Hb. A1C - diabetic control
42W..00	Test request : Haemoglobin A1c
42W..11	Glycosylated Hb
42W..12	Glycated haemoglobin
42W1.00	Hb. A1C < 7% - good control
42W2.00	Hb. A1C 7-10% - borderline
42W3.00	Hb. A1C > 10% - bad control
42W4.00	HbA1c level (DCCT aligned)
42W5.00	Haemoglobin A1c level - IFCC standardised
42WZ.00	Hb. A1C - diabetic control NOS
42c..00	HbA1 - diabetic control
42c0.00	HbA1 < 7% - good control
42c1.00	HbA1 7 - 10% - borderline control
42c2.00	HbA1 > 10% - bad control
42c3.00	HbA1 level (DCCT aligned)
44T1200	Random blood sugar raised
44T2.00	Fasting blood sugar
44TB.00	Haemoglobin A1c level
44TB000	Haemoglobin A1c (diagnostic reference range)
44TB100	Haemoglobin A1c (monitoring ranges)
44TC.00	Haemoglobin A1 level
44TJ.00	Blood glucose level
44TK.00	Fasting blood glucose level
44TL.00	Total glycosylated haemoglobin level
44U..00	Blood glucose result
44U..11	Blood sugar result
44U5.00	Blood glucose 7-9.9 mmol/L
44U6.00	Blood glucose 10-13.9 mmol/L
66Ae.00	HBA1c target
66Ae000	HbA1c target level - IFCC standardised
C109711	Type II diabetes mellitus - poor control

Carr MJ, *et al. BMJ Qual Saf* 2022; 31:503–514. doi: 10.1136/bmjqs-2021-013613

Appendix 14. Read codes for blood pressure monitoring

Read code	Term
246..00	O/E - blood pressure reading
246..11	O/E - BP reading
246..12	O/E - blood pressure
2462.00	O/E - BP reading low
2468.00	O/E - BP reading:postural drop
2469.00	Systolic blood pressure
246A.00	Diastolic blood pressure
246C.00	Lying blood pressure reading
246D.00	Standing blood pressure reading
246E.00	Sitting blood pressure reading
246G.00	O/E - BP labile
246N.00	Standing systolic blood pressure
246P.00	Standing diastolic blood pressure
246Q.00	Sitting systolic blood pressure
246R.00	Sitting diastolic blood pressure
246S.00	Lying systolic blood pressure
246T.00	Lying diastolic blood pressure
246V.00	Average 24 hour diastolic blood pressure
246W.00	Average 24 hour systolic blood pressure
246X.00	Average day interval diastolic blood pressure
246Y.00	Average day interval systolic blood pressure
246Z.00	O/E-blood pressure reading NOS
246a.00	Average night interval diastolic blood pressure
246b.00	Average night interval systolic blood pressure
246c.00	Average home diastolic blood pressure
246d.00	Average home systolic blood pressure
246e.00	Ambulatory systolic blood pressure
246f.00	Ambulatory diastolic blood pressure
246g.00	Self measured blood pressure reading
246i.00	Diastolic blood pressure centile
246j.00	Systolic blood pressure centile
246l.00	Average systolic blood pressure
246m.00	Average diastolic blood pressure
246n.00	Baseline blood pressure
246n000	Baseline diastolic blood pressure
246n100	Baseline systolic blood pressure
315B.00	Ambulatory blood pressure recording
662L.00	24 hr blood pressure monitoring
662Q.00	Borderline blood pressure
662V.00	Blood pressure monitoring
662j.00	Blood pressure recorded by patient at home
7P1B200	Application of ambulatory blood pressure monitor
8A59.00	Self-monitoring of blood pressure
9OD..11	BP screen administration

G20..11	High blood pressure
R1y2.00	[D]Raised blood pressure reading
R1y3.00	[D]Low blood pressure reading
R1y3.99	Nonspecific low BP reading [D]
ZV70B00	[V]Examination of blood pressure

Appendix 15. Read codes for cholesterol monitoring

Read code	Term
44O8.00	Plasma VLDL triglyceride level
44OB.00	Serum VLDL triglyceride level
44OE.00	Plasma total cholesterol level
44P..00	Test request : Serum cholesterol
44P..00	Serum cholesterol
44P1.00	Serum cholesterol normal
44P2.00	Serum cholesterol borderline
44P3.00	Serum cholesterol raised
44P4.00	Serum cholesterol very high
44P5.00	Serum HDL cholesterol level
44P5.00	Test request : Serum HDL cholesterol level
44P6.00	Serum LDL cholesterol level
44P7.00	Serum VLDL cholesterol level
44P8.00	Serum HDL:non-HDL cholesterol ratio
44P9.00	Serum cholesterol studies
44PB.00	Serum fasting HDL cholesterol level
44PC.00	Serum random HDL cholesterol level
44PD.00	Serum fasting LDL cholesterol level
44PE.00	Serum random LDL cholesterol level
44PF.00	Total cholesterol:HDL ratio
44PG.00	HDL : total cholesterol ratio
44PH.00	Total cholesterol measurement
44PI.00	Calculated LDL cholesterol level
44PJ.00	Serum total cholesterol level
44PK.00	Serum fasting total cholesterol
44PL.00	Non HDL cholesterol level
44PL000	Serum non high density lipoprotein cholesterol level
44PL100	Estimated serum non-HDL cholesterol level
44PZ.00	Serum cholesterol NOS
44Q..00	Test request : Serum triglycerides
44Q..00	Serum triglycerides
44Q1.00	Serum triglycerides normal
44Q2.00	Serum triglycerides borderline
44Q3.00	Serum triglycerides raised
44Q4.00	Serum fasting triglyceride level
44Q5.00	Serum random triglyceride level
44QZ.00	Serum triglycerides NOS
44d2.00	Plasma random HDL cholesterol level
44d3.00	Plasma fasting HDL cholesterol level
44d4.00	Plasma random LDL cholesterol level
44d5.00	Plasma fasting LDL cholesterol level
44dA.00	Plasma HDL cholesterol level
44dB.00	Plasma LDL cholesterol level
44e..00	Plasma triglyceride level

44e0.00	Plasma random triglyceride level
44e1.00	Plasma fasting triglyceride level
44l2.00	Cholesterol/HDL ratio
44lF.00	Serum cholesterol/HDL ratio
44lG.00	Plasma cholesterol/HDL ratio
44lH.00	Serum cholesterol/LDL ratio
44lI.00	Plasma cholesterol/LDL ratio
44lJ.00	Serum cholesterol/VLDL ratio
44lK.00	Plasma cholesterol/VLDL ratio
44lzY00	Serum HDL cholesterol:triglyceride ratio
4I3O.00	Fluid sample cholesterol level
4I3W.00	Fluid sample triglyceride level
4QA1.00	Triglyceride level
662a.00	Pre-treatment serum cholesterol level
6879.12	Lipid screen
C320.13	Low density lipoproteinaemia
C320300	Low-density-lipoprotein-type (LDL) hyperlipoproteinaemia
C321.12	Very low density lipoprotinaemia

Appendix 16. Read codes for serum creatinine monitoring

Read code	Term
1Z1a.00	CKD G4A1 - chronic kidney disease with glomerular filtration rate category G4 and albuminuria category A1
1Z1b.00	CKD G4A2 - chronic kidney disease with glomerular filtration rate category G4 and albuminuria category A2
1Z1c.00	CKD G4A3 - chronic kidney disease with glomerular filtration rate category G4 and albuminuria category A3
1Z1d.00	CKD G5A1 - chronic kidney disease with glomerular filtration rate category G5 and albuminuria category A1
1Z1e.00	CKD G5A2 - chronic kidney disease with glomerular filtration rate category G5 and albuminuria category A2
1Z1f.00	CKD G5A3 - chronic kidney disease with glomerular filtration rate category G5 and albuminuria category A3
1Z1M.00	CKD G1A1 - chronic kidney disease with glomerular filtration rate category G1 and albuminuria category A1
1Z1N.00	CKD G1A2 - chronic kidney disease with glomerular filtration rate category G1 and albuminuria category A2
1Z1P.00	CKD G1A3 - chronic kidney disease with glomerular filtration rate category G1 and albuminuria category A3
1Z1Q.00	CKD G2A1 - chronic kidney disease with glomerular filtration rate category G2 and albuminuria category A1
1Z1R.00	CKD G2A2 - chronic kidney disease with glomerular filtration rate category G2 and albuminuria category A2
1Z1S.00	CKD G2A3 - chronic kidney disease with glomerular filtration rate category G2 and albuminuria category A3
1Z1T.00	CKD G3aA1 - chronic kidney disease with glomerular filtration rate category G3a and albuminuria category A1
1Z1V.00	CKD G3aA2 - chronic kidney disease with glomerular filtration rate category G3a and albuminuria category A2
1Z1W.00	CKD G3aA3 - chronic kidney disease with glomerular filtration rate category G3a and albuminuria category A3
1Z1X.00	CKD G3bA1 - chronic kidney disease with glomerular filtration rate category G3b and albuminuria category A1
1Z1Y.00	CKD G3bA2 - chronic kidney disease with glomerular filtration rate category G3b and albuminuria category A2
1Z1Z.00	CKD G3bA3 - chronic kidney disease with glomerular filtration rate category G3b and albuminuria category A3
44J3.00	Serum creatinine level
44J3.00	Test request : Creatinine
44J3000	Serum creatinine abnormal
44J3100	Serum creatinine low
44J3200	Serum creatinine normal
44J3300	Serum creatinine raised
44J3z00	Serum creatinine NOS
44JC.00	Corrected plasma creatinine level
44JD.00	Corrected serum creatinine level
44JF.00	Plasma creatinine level
4513.00	Creatinine clearance-glom filt

4513.11	Creatinine clearance
4513000	Creatinine clearance-glomerular filtration normal
4513100	Creatinine clearance-glomerular filtration abnormal
4517.00	Creatinine clearance test
4517.00	Test request : Creatinine clearance test
451A.00	Estimated creatinine clearance
451A000	Estimated creatinine clearance (Cockcroft-Gault formula)
451E.00	GFR (glomerular filtration rate) calculated by abbreviated Modification of Diet in Renal Disease Study Group calculation
451F.00	Glomerular filtration rate
451G.00	GFR (glomerular filtration rate) calculated by abbreviated Modification of Diet in Renal Disease Study Group calculation adjusted for African American origin
451K.00	Estimated glomerular filtration rate using Chronic Kidney Disease Epidemiology Collaboration formula
451M.00	eGFR (estimated glomerular filtration rate) using cystatin C Chronic Kidney Disease Epidemiology Collaboration equation per 1.73 square metres
451N.00	eGFR (estimated glomerular filtration rate) using creatinine Chronic Kidney Disease Epidemiology Collaboration equation per 1.73 square metres
46M7.00	Urine creatinine level
46MD.00	24 hour urine creatinine output
46N7.00	Urine protein/creatinine index
4Q40.00	Creatinine level
7P14000	Glomerular filtration rate testing
K014.00	Nephrotic syndrome, minor glomerular abnormality
K015.00	Nephrotic syndrome, focal and segmental glomerular lesions
K0A1000	Rapidly progressive nephritic syndrome, minor glomerular abnormality
Kyu0C00	[X]Unspecified nephritic syndrome, diffuse concentric glomerulonephritis
Kyu0D00	[X]Isolated proteinuria, with oth specif morpholog changes
Kyu0E00	[X]Isolated proteinuria, with unspecified morphological changes

Carr MJ, *et al. BMJ Qual Saf* 2022; 31:503–514. doi: 10.1136/bmjqs-2021-013613

Appendix 17. Read codes for urine albumin monitoring

Read code	Term
1Z1M.00	CKD G1A1 - chronic kidney disease with glomerular filtration rate category G1 and albuminuria category A1
1Z1N.00	CKD G1A2 - chronic kidney disease with glomerular filtration rate category G1 and albuminuria category A2
1Z1P.00	CKD G1A3 - chronic kidney disease with glomerular filtration rate category G1 and albuminuria category A3
1Z1Q.00	CKD G2A1 - chronic kidney disease with glomerular filtration rate category G2 and albuminuria category A1
1Z1R.00	CKD G2A2 - chronic kidney disease with glomerular filtration rate category G2 and albuminuria category A2
1Z1S.00	CKD G2A3 - chronic kidney disease with glomerular filtration rate category G2 and albuminuria category A3
1Z1T.00	CKD G3aA1 - chronic kidney disease with glomerular filtration rate category G3a and albuminuria category A1
1Z1V.00	CKD G3aA2 - chronic kidney disease with glomerular filtration rate category G3a and albuminuria category A2
1Z1W.00	CKD G3aA3 - chronic kidney disease with glomerular filtration rate category G3a and albuminuria category A3
1Z1X.00	CKD G3bA1 - chronic kidney disease with glomerular filtration rate category G3b and albuminuria category A1
1Z1Y.00	CKD G3bA2 - chronic kidney disease with glomerular filtration rate category G3b and albuminuria category A2
1Z1Z.00	CKD G3bA3 - chronic kidney disease with glomerular filtration rate category G3b and albuminuria category A3
1Z1a.00	CKD G4A1 - chronic kidney disease with glomerular filtration rate category G4 and albuminuria category A1
1Z1b.00	CKD G4A2 - chronic kidney disease with glomerular filtration rate category G4 and albuminuria category A2
1Z1c.00	CKD G4A3 - chronic kidney disease with glomerular filtration rate category G4 and albuminuria category A3
1Z1d.00	CKD G5A1 - chronic kidney disease with glomerular filtration rate category G5 and albuminuria category A1
1Z1e.00	CKD G5A2 - chronic kidney disease with glomerular filtration rate category G5 and albuminuria category A2
1Z1f.00	CKD G5A3 - chronic kidney disease with glomerular filtration rate category G5 and albuminuria category A3
44J6.00	Albumin excretion rate
44J7.00	Albumin/creatinine ratio
44JG.00	Overnight albumin excretion rate
4618.00	Urine dipstick test
467..00	Urine protein test
4672.00	Urine protein test negative
4673.00	Urine protein test = trace
4674.00	Urine protein test = +
4675.00	Urine protein test = ++
4676.00	Urine protein test = +++
4677.00	Urine protein test = ++++

Carr MJ, *et al. BMJ Qual Saf* 2022; 31:503–514. doi: 10.1136/bmjqs-2021-013613

4678.00	Proteinuria
4679.00	Urine dipstick for protein
467A.00	24 hour urine protein output
467E.00	Urine protein level
467H.00	Random urine protein level
467Z.00	Urine protein test NOS
46N..00	Urine protein
46N4.00	Test request : Urinary Microalbumin
46N4.00	Urine albumin level
46N5.00	24 hour urine protein excretion test
46N5.00	Test request : 24 hour urine protein excretion test
46N6.00	24 hour urine albumin output
46N8.00	Urine microalbumin profile
46TC.00	Urine albumin:creatinine ratio
46TC.00	Test request : Urine albumin:creatinine ratio
46TD.00	Urine microalbumin/creatinine ratio
46W..00	Urine microalbumin level
46W0.00	Urine microalbumin positive
46W1.00	Urine microalbumin negative
46W2.00	Microalbumin excretion rate
66Ak.00	Diabetes monitoring lower risk albumin excretion
66Al.00	Diabetes monitoring higher risk albumin excretion
C10FM00	Persistent microalbuminuria associated with type 2 diabetes mellitus
C10FM11	Type II diabetes mellitus with persistent microalbuminuria
C10yz99	DM + persist microalbuminuria
R110.00	[D]Proteinuria
R110000	Albuminuria
R110300	Microalbuminuria
R110z00	[D]Proteinuria NOS

Carr MJ, *et al. BMJ Qual Saf* 2022; 31:503–514. doi: 10.1136/bmjqs-2021-013613

Appendix 18. Read codes for recording body mass index

Read code	Term
222A.00	O/E - obese
229..00	Standing height
2291.00	O/E-height > 20% below average
2292.00	O/E - height 10-20% < average
2293.00	O/E -height within 10% average
2294.00	O/E-height 10-20% over average
2295.00	O/E -height > 20% over average
229Z.00	O/E - height NOS
22A..00	Body weight
22A1.00	O/E - weight > 20% below ideal
22A2.00	O/E -weight 10-20% below ideal
22A3.00	O/E - weight within 10% ideal
22A4.00	O/E - weight 10-20% over ideal
22A4.11	O/E - overweight
22A5.00	O/E - weight greater than 20% over ideal
22A5.11	O/E - obese
22A6.00	O/E - Underweight
22A7.00	Baseline weight
22AA.00	Overweight
22AZ.00	O/E - weight NOS
22K..00	Body mass index
22K1.00	Normal body mass index
22K2.00	Increased body mass index
22K3.00	Decreased body mass index
22K4.00	Body mass index index 25-29 - overweight
22K5.00	Body mass index 30+ - obesity
22K6.00	Body mass index less than 20
22K7.00	Body mass index 40+ - severely obese
22K8.00	Body mass index 20-24 - normal
22K9000	Baseline body mass index centile
22KB.00	Baseline body mass index
22KC.00	Obese class I (body mass index 30.0 - 34.9)
22KD.00	Obese class II (body mass index 35.0 - 39.9)
22KE.00	Obese class III (body mass index equal to or greater than 40.0)
22Z..00	Height and weight
4I5..00	Sample weight
66C..00	Obesity monitoring
66C..11	Weight monitoring
66C1.00	Initial obesity assessment
6878.11	Weight screening
9OK..11	Obesity clinic administration
C380.00	Obesity
R034800	Underweight

Part B2. Drug prescription codes used to interrogate CPRD *GOLD*

Appendix 19. Product codes for prescriptions of antidiabetic medications

Product code	Term	Drug class
5174	Acarbose 100mg tablets	AGi
479	Acarbose 50mg tablets	AGi
71020	Acarbose 50mg tablets (Actavis UK Ltd)	AGi
9105	Glucobay 100mg tablets (Bayer Plc)	AGi
5621	Glucobay 50mg tablets (Bayer Plc)	AGi
60328	Alogliptin 12.5mg tablets	DPP-4i
59177	Alogliptin 25mg tablets	DPP-4i
68258	Alogliptin 25mg tablets (Colorama Pharmaceuticals Ltd)	DPP-4i
69459	Alogliptin 25mg tablets (Ennogen Healthcare Ltd)	DPP-4i
59809	Alogliptin 6.25mg tablets	DPP-4i
39149	Galvus 50mg tablets (Novartis Pharmaceuticals UK Ltd)	DPP-4i
35462	Januvia 100mg tablets (Merck Sharp & Dohme Ltd)	DPP-4i
50124	Januvia 25mg tablets (Merck Sharp & Dohme Ltd)	DPP-4i
50087	Januvia 50mg tablets (Merck Sharp & Dohme Ltd)	DPP-4i
46665	Linagliptin 5mg tablets	DPP-4i
45821	Onglyza 2.5mg tablets (AstraZeneca UK Ltd)	DPP-4i
41431	Onglyza 5mg tablets (AstraZeneca UK Ltd)	DPP-4i
74051	Onglyza 5mg tablets (Sigma Pharmaceuticals Plc)	DPP-4i
45775	Saxagliptin 2.5mg tablets	DPP-4i
41204	Saxagliptin 5mg tablets	DPP-4i
35022	Sitagliptin 100mg tablets	DPP-4i
73150	Sitagliptin 100mg tablets (Waymade Healthcare Plc)	DPP-4i
48533	Sitagliptin 25mg tablets	DPP-4i
48401	Sitagliptin 50mg tablets	DPP-4i
72539	Sitagliptin 50mg/5ml oral solution	DPP-4i
46716	Trajenta 5mg tablets (Boehringer Ingelheim Ltd)	DPP-4i
37875	Vildagliptin 50mg tablets	DPP-4i
60681	Vipidia 12.5mg tablets (Takeda UK Ltd)	DPP-4i
60682	Vipidia 25mg tablets (Takeda UK Ltd)	DPP-4i
62326	Vipidia 6.25mg tablets (Takeda UK Ltd)	DPP-4i
60497	Alogliptin 12.5mg / Metformin 1g tablets	DPP-4i/Metformin
38551	Eucreas 50mg/1000mg tablets (Novartis Pharmaceuticals UK Ltd)	DPP-4i/Metformin
39203	Eucreas 50mg/850mg tablets (Novartis Pharmaceuticals UK Ltd)	DPP-4i/Metformin
43684	Janumet 50mg/1000mg tablets (Merck Sharp & Dohme Ltd)	DPP-4i/Metformin
50682	Jentadueto 2.5mg/1000mg tablets (Boehringer Ingelheim Ltd)	DPP-4i/Metformin
54150	Jentadueto 2.5mg/850mg tablets (Boehringer Ingelheim Ltd)	DPP-4i/Metformin
56965	Komboglyze 2.5mg/1000mg tablets (AstraZeneca UK Ltd)	DPP-4i/Metformin
58865	Komboglyze 2.5mg/850mg tablets (AstraZeneca UK Ltd)	DPP-4i/Metformin
52445	Linagliptin 2.5mg / Metformin 1g tablets	DPP-4i/Metformin
52449	Linagliptin 2.5mg / Metformin 850mg tablets	DPP-4i/Metformin
43619	Metformin 1g / Sitagliptin 50mg tablets	DPP-4i/Metformin
54891	Saxagliptin 2.5mg / Metformin 1g tablets	DPP-4i/Metformin

54973	Saxagliptin 2.5mg / Metformin 850mg tablets	DPP-4i/Metformin
37902	Vildagliptin 50mg / Metformin 1g tablets	DPP-4i/Metformin
37874	Vildagliptin 50mg / Metformin 850mg tablets	DPP-4i/Metformin
59385	Vipdomet 12.5mg/1000mg tablets (Takeda UK Ltd)	DPP-4i/Metformin
69947	Albiglutide 30mg powder and solvent for solution for injection pre-filled disposable devices	GLP-1ag
62661	Bydureon 2mg powder and solvent for prolonged-release suspension for injection pre-filled pen (AstraZeneca UK Ltd)	GLP-1ag
46469	Bydureon 2mg powder and solvent for prolonged-release suspension for injection vials (AstraZeneca UK Ltd)	GLP-1ag
64622	Bydureon 2mg powder and solvent for prolonged-release suspension for injection vials (Lexon (UK) Ltd)	GLP-1ag
35150	Byetta 10micrograms/0.04ml solution for injection 2.4ml pre-filled disposable devices (AstraZeneca UK Ltd)	GLP-1ag
35144	Byetta 5micrograms/0.02ml solution for injection 1.2ml pre-filled disposable devices (AstraZeneca UK Ltd)	GLP-1ag
63785	Dulaglutide 0.75mg/0.5ml solution for injection pre-filled disposable devices	GLP-1ag
63823	Dulaglutide 1.5mg/0.5ml solution for injection pre-filled disposable devices	GLP-1ag
35149	Exenatide 10micrograms/0.04ml solution for injection 2.4ml pre-filled disposable devices	GLP-1ag
62904	Exenatide 2mg powder and solvent for prolonged-release suspension for injection pre-filled disposable devices	GLP-1ag
46458	Exenatide 2mg powder and solvent for prolonged-release suspension for injection vials	GLP-1ag
35251	Exenatide 5micrograms/0.02ml solution for injection 1.2ml pre-filled disposable devices	GLP-1ag
40693	Liraglutide 6mg/ml solution for injection 3ml pre-filled disposable devices	GLP-1ag
55459	Lixisenatide 10micrograms/0.2ml solution for injection 3ml pre-filled disposable devices	GLP-1ag
55723	Lixisenatide 10micrograms/0.2ml solution for injection 3ml pre-filled disposable devices and Lixisenatide 20micrograms/0.2ml solution for injection 3ml pre-filled disposable devices	GLP-1ag
55413	Lixisenatide 20micrograms/0.2ml solution for injection 3ml pre-filled disposable devices	GLP-1ag
55728	Lyxumia 10micrograms/0.2ml solution for injection 3ml pre-filled pen (Sanofi)	GLP-1ag
55767	Lyxumia 10micrograms/20micrograms treatment initiation pack (Sanofi)	GLP-1ag
55729	Lyxumia 20micrograms/0.2ml solution for injection 3ml pre-filled pen (Sanofi)	GLP-1ag
69548	Saxenda 6mg/ml solution for injection 3ml pre-filled pen (Novo Nordisk Ltd)	GLP-1ag
63401	Trulicity 0.75mg/0.5ml solution for injection pre-filled pen (Eli Lilly and Company Ltd)	GLP-1ag
63336	Trulicity 1.5mg/0.5ml solution for injection pre-filled pen (Eli Lilly and Company Ltd)	GLP-1ag
40642	Victoza 6mg/ml solution for injection 3ml pre-filled pen (Novo Nordisk Ltd)	GLP-1ag
63562	Insulin degludec 100units/ml / Liraglutide 3.6mg/ml solution for injection 3ml pre-filled disposable devices	GLP-1ag/Insulin
62899	Xultophy 100units/ml / 3.6mg/ml solution for injection 3ml pre-filled pen (Novo Nordisk Ltd)	GLP-1ag/Insulin
64987	Abasaglar 100units/ml solution for injection 3ml cartridges (Eli Lilly and Company Ltd)	Insulin
64723	Abasaglar KwikPen 100units/ml solution for injection 3ml pre-filled pen (Eli Lilly and Company Ltd)	Insulin

1588	Actrapid 100iu/ml Injection (Novo Nordisk Ltd)	Insulin
7349	Actrapid 100units/ml solution for injection 10ml vials (Novo Nordisk Ltd)	Insulin
74824	Actrapid 100units/ml solution for injection 10ml vials (Waymade Healthcare Plc)	Insulin
1594	Actrapid NovoLet 100units/ml solution for injection (Novo Nordisk Ltd)	Insulin
56502	Actrapid Penfill 100units/ml solution for injection 3ml cartridges (Novo Nordisk Ltd)	Insulin
30209	Actrapid mc 100unit/ml Injection (Arun Products Ltd)	Insulin
1592	Actrapid penfill 100 100iu/ml Penfill (Novo Nordisk Ltd)	Insulin
19491	Apidra 100units/ml solution for injection 10ml vials (Sanofi)	Insulin
29953	Apidra 100units/ml solution for injection 3ml OptiClik cartridges (Sanofi)	Insulin
14345	Apidra 100units/ml solution for injection 3ml cartridges (Sanofi)	Insulin
21583	Apidra 100units/ml solution for injection 3ml pre-filled OptiSet pen (Sanofi)	Insulin
74392	Apidra 100units/ml solution for injection 3ml pre-filled SoloStar pen (Lexon (UK) Ltd)	Insulin
36920	Apidra 100units/ml solution for injection 3ml pre-filled SoloStar pen (Sanofi)	Insulin
31465	Exubera 1mg inhalation powder blisters (Pfizer Ltd)	Insulin
31467	Exubera 3mg inhalation powder blisters (Pfizer Ltd)	Insulin
69715	Fiasp 100units/ml solution for injection 10ml vials (Novo Nordisk Ltd)	Insulin
69823	Fiasp FlexTouch 100units/ml solution for injection 3ml pre-filled pen (Novo Nordisk Ltd)	Insulin
70055	Fiasp Penfill 100units/ml solution for injection 3ml cartridges (Novo Nordisk Ltd)	Insulin
7793	HumaJect M3 Pen 100units/ml suspension for injection (Eli Lilly and Company Ltd)	Insulin
9565	HumaJect S Pen 100units/ml solution for injection (Eli Lilly and Company Ltd)	Insulin
8118	Humaject i 100iu/ml Pen (Eli Lilly and Company Ltd)	Insulin
10915	Humaject m1 100iu/ml M1 pen (Eli Lilly and Company Ltd)	Insulin
10910	Humaject m2 100iu/ml M2 pen (Eli Lilly and Company Ltd)	Insulin
17809	Humaject m4 100iu/ml M4 pen (Eli Lilly and Company Ltd)	Insulin
22155	Humaject m5 100iu/ml M5 pen (Eli Lilly and Company Ltd)	Insulin
322	Humalog 100units/ml solution for injection 1.5ml cartridges (Eli Lilly and Company Ltd)	Insulin
57529	Humalog 100units/ml solution for injection 10ml vials (Dowelhurst Ltd)	Insulin
18224	Humalog 100units/ml solution for injection 10ml vials (Eli Lilly and Company Ltd)	Insulin
7318	Humalog 100units/ml solution for injection 3ml cartridges (Eli Lilly and Company Ltd)	Insulin
73282	Humalog Junior KwikPen 100units/ml solution for injection 3ml pre-filled pen (Eli Lilly and Company Ltd)	Insulin
55603	Humalog KwikPen 100units/ml solution for injection 3ml pre-filled pen (DE Pharmaceuticals)	Insulin
38986	Humalog KwikPen 100units/ml solution for injection 3ml pre-filled pen (Eli Lilly and Company Ltd)	Insulin
74389	Humalog KwikPen 100units/ml solution for injection 3ml pre-filled pen (Sigma Pharmaceuticals Plc)	Insulin
57564	Humalog KwikPen 100units/ml solution for injection 3ml pre-filled pen (Waymade Healthcare Plc)	Insulin
63464	Humalog KwikPen 200units/ml solution for injection 3ml pre-filled pen (Eli Lilly and Company Ltd)	Insulin

42395	Humalog Mix25 100units/ml suspension for injection 10ml vials (Eli Lilly and Company Ltd)	Insulin
10243	Humalog Mix25 100units/ml suspension for injection 3ml cartridges (Eli Lilly and Company Ltd)	Insulin
71395	Humalog Mix25 100units/ml suspension for injection 3ml cartridges (Sigma Pharmaceuticals Plc)	Insulin
69583	Humalog Mix25 100units/ml suspension for injection 3ml cartridges (Waymade Healthcare Plc)	Insulin
39006	Humalog Mix25 KwikPen 100units/ml suspension for injection 3ml pre-filled pen (Eli Lilly and Company Ltd)	Insulin
68031	Humalog Mix25 KwikPen 100units/ml suspension for injection 3ml pre-filled pen (Sigma Pharmaceuticals Plc)	Insulin
74071	Humalog Mix25 KwikPen 100units/ml suspension for injection 3ml pre-filled pen (Waymade Healthcare Plc)	Insulin
14270	Humalog Mix25 Pen 100units/ml suspension for injection 3ml pre-filled pen (Eli Lilly and Company Ltd)	Insulin
71430	Humalog Mix25 Pen 100units/ml suspension for injection 3ml pre-filled pen (Waymade Healthcare Plc)	Insulin
18593	Humalog Mix50 100units/ml suspension for injection 3ml cartridges (Eli Lilly and Company Ltd)	Insulin
74388	Humalog Mix50 100units/ml suspension for injection 3ml cartridges (Sigma Pharmaceuticals Plc)	Insulin
52522	Humalog Mix50 KwikPen 100units/ml suspension for injection 3ml pre-filled pen (DE Pharmaceuticals)	Insulin
39086	Humalog Mix50 KwikPen 100units/ml suspension for injection 3ml pre-filled pen (Eli Lilly and Company Ltd)	Insulin
74391	Humalog Mix50 KwikPen 100units/ml suspension for injection 3ml pre-filled pen (Sigma Pharmaceuticals Plc)	Insulin
57622	Humalog Mix50 KwikPen 100units/ml suspension for injection 3ml pre-filled pen (Waymade Healthcare Plc)	Insulin
10001	Humalog Mix50 Pen 100units/ml suspension for injection 3ml pre-filled pen (Eli Lilly and Company Ltd)	Insulin
10264	Humalog Pen 100units/ml solution for injection 3ml pre-filled pen (Eli Lilly and Company Ltd)	Insulin
4715	Humalog mix 25 25/75 100units/ml Injection (Eli Lilly and Company Ltd)	Insulin
56115	Human Actrapid Penfill 100units/ml solution for injection 1.5ml cartridges (Novo Nordisk Ltd)	Insulin
72559	Human Insulatard Penfill 100units/ml suspension for injection 1.5ml cartridges (Novo Nordisk Ltd)	Insulin
50691	Human Mixtard 20 Penfill 100units/ml suspension for injection 1.5ml cartridges (Novo Nordisk Ltd)	Insulin
52722	Human Mixtard 30 Penfill 100units/ml suspension for injection 1.5ml cartridges (Novo Nordisk Ltd)	Insulin
12818	Human Mixtard 50 100units/ml suspension for injection 10ml vials (Novo Nordisk Ltd)	Insulin
1649	Human actraphane 100iu/ml Injection (Novo Nordisk Ltd)	Insulin
34097	Human initard 50/50 100unit/ml Injection (Novo Nordisk Ltd)	Insulin
7772	Human protaphane 100unit/ml Injection (Novo Nordisk Ltd)	Insulin
7771	Human protaphane penfill 100 100unit/ml Penfill (Novo Nordisk Ltd)	Insulin
14918	Humulin I 100units/ml suspension for injection 10ml vials (Eli Lilly and Company Ltd)	Insulin

Carr MJ, et al. BMJ Qual Saf 2022; 31:503–514. doi: 10.1136/bmjqs-2021-013613

74845	Humulin I 100units/ml suspension for injection 10ml vials (Waymade Healthcare Plc)	Insulin
14357	Humulin I 100units/ml suspension for injection 3ml cartridges (Eli Lilly and Company Ltd)	Insulin
71361	Humulin I 100units/ml suspension for injection 3ml cartridges (Waymade Healthcare Plc)	Insulin
43950	Humulin I KwikPen 100units/ml suspension for injection 3ml pre-filled pen (Eli Lilly and Company Ltd)	Insulin
10229	Humulin I Pen 100units/ml suspension for injection 3ml pre-filled pen (Eli Lilly and Company Ltd)	Insulin
10547	Humulin Lente 100units/ml suspension for injection 10ml vials (Eli Lilly and Company Ltd)	Insulin
4093	Humulin M2 100units/ml suspension for injection 3ml cartridges (Eli Lilly and Company Ltd)	Insulin
19513	Humulin M3 100units/ml suspension for injection 10ml vials (Eli Lilly and Company Ltd)	Insulin
57620	Humulin M3 100units/ml suspension for injection 10ml vials (Mawdsley-Brooks & Company Ltd)	Insulin
60933	Humulin M3 100units/ml suspension for injection 10ml vials (Sigma Pharmaceuticals Plc)	Insulin
10277	Humulin M3 100units/ml suspension for injection 3ml cartridges (Eli Lilly and Company Ltd)	Insulin
74842	Humulin M3 100units/ml suspension for injection 3ml cartridges (Mawdsley-Brooks & Company Ltd)	Insulin
67324	Humulin M3 100units/ml suspension for injection 3ml cartridges (Waymade Healthcare Plc)	Insulin
43991	Humulin M3 KwikPen 100units/ml suspension for injection 3ml pre-filled pen (Eli Lilly and Company Ltd)	Insulin
16160	Humulin M3 Pen 100units/ml suspension for injection 3ml pre-filled pen (Eli Lilly and Company Ltd)	Insulin
8841	Humulin M5 100units/ml suspension for injection 10ml vials (Eli Lilly and Company Ltd)	Insulin
62276	Humulin R 500units/ml solution for injection 20ml vials (Imported (United States))	Insulin
72479	Humulin R KwikPen 500units/ml solution for injection 3ml pre-filled pen (Imported (United States))	Insulin
21235	Humulin S 100units/ml solution for injection 10ml vials (Eli Lilly and Company Ltd)	Insulin
14944	Humulin S 100units/ml solution for injection 3ml cartridges (Eli Lilly and Company Ltd)	Insulin
7537	Humulin Zn 100units/ml suspension for injection 10ml vials (Eli Lilly and Company Ltd)	Insulin
4760	Humulin i 100unit/ml Injection (Eli Lilly and Company Ltd)	Insulin
4199	Humulin m1 100unit/ml M1 injection (Eli Lilly and Company Ltd)	Insulin
4198	Humulin m3 100unit/ml M3 injection (Eli Lilly and Company Ltd)	Insulin
11107	Humulin m4 100unit/ml M4 injection (Eli Lilly and Company Ltd)	Insulin
1840	Humulin s 100unit/ml Injection (Eli Lilly and Company Ltd)	Insulin
71137	Hypurin Bovine Isophane 100units/ml suspension for injection 10ml vials (Waymade Healthcare Plc)	Insulin
14340	Hypurin Bovine Isophane 100units/ml suspension for injection 10ml vials (Wockhardt UK Ltd)	Insulin

28588	Hypurin Bovine Isophane 100units/ml suspension for injection 3ml cartridges (Wockhardt UK Ltd)	Insulin
17712	Hypurin Bovine Lente 100units/ml suspension for injection 10ml vials (Wockhardt UK Ltd)	Insulin
14339	Hypurin Bovine Neutral 100units/ml solution for injection 10ml vials (Wockhardt UK Ltd)	Insulin
23231	Hypurin Bovine Neutral 100units/ml solution for injection 3ml cartridges (Wockhardt UK Ltd)	Insulin
9503	Hypurin Bovine Protamine Zinc 100units/ml suspension for injection 10ml vials (Wockhardt UK Ltd)	Insulin
9618	Hypurin Porcine 30/70 Mix 100units/ml suspension for injection 1.5ml cartridges (C P Pharmaceuticals Ltd)	Insulin
24800	Hypurin Porcine 30/70 Mix 100units/ml suspension for injection 10ml vials (Wockhardt UK Ltd)	Insulin
20995	Hypurin Porcine 30/70 Mix 100units/ml suspension for injection 3ml cartridges (Wockhardt UK Ltd)	Insulin
13819	Hypurin Porcine Isophane 100units/ml suspension for injection 1.5ml cartridges (C P Pharmaceuticals Ltd)	Insulin
28183	Hypurin Porcine Isophane 100units/ml suspension for injection 10ml vials (Wockhardt UK Ltd)	Insulin
14933	Hypurin Porcine Isophane 100units/ml suspension for injection 3ml cartridges (Wockhardt UK Ltd)	Insulin
26098	Hypurin Porcine Neutral 100units/ml solution for injection 10ml vials (Wockhardt UK Ltd)	Insulin
14930	Hypurin Porcine Neutral 100units/ml solution for injection 3ml cartridges (Wockhardt UK Ltd)	Insulin
13516	Hypurin bovine isophane 100unit/ml Injection (C P Pharmaceuticals Ltd)	Insulin
12297	Hypurin bovine neutral 100unit/ml Injection (C P Pharmaceuticals Ltd)	Insulin
13622	Hypurin porcine neutral 100unit/ml Injection (C P Pharmaceuticals Ltd)	Insulin
63679	Hypurin soluble 100iu/ml Injection (C P Pharmaceuticals Ltd)	Insulin
14506	INSULIN BOVINE PROTAMINE ZINC 100 I/U INJ	Insulin
20195	INSULIN BOVINE PROTAMINE ZINC 40 I/U INJ	Insulin
13550	INSULIN BP 100 I/U	Insulin
20671	INSULIN HUM/ACTRAPHANE	Insulin
20672	INSULIN HUM/ACTRAPID	Insulin
32053	INSULIN HUMALOG MIX 25	Insulin
25006	INSULIN HUMAN ACTRAPID (NEUTRAL)	Insulin
321	INSULIN HUMAN ACTRAPID (NEUTRAL) 40 I/U INJ	Insulin
27911	INSULIN HUMAN ACTRAPID PENFILL	Insulin
2373	INSULIN HUMAN VELOSULIN 100 I/U INJ	Insulin
1839	INSULIN HUMULIN I (ISOPHANE) 100 I/U INJ	Insulin
10566	INSULIN HUMULIN M CARTRIDGE 100 I/U	Insulin
22161	INSULIN HUMULIN M1 VIAL	Insulin
22094	INSULIN HUMULIN M2 VIAL	Insulin
10546	INSULIN HUMULIN M4 100 I/U INJ	Insulin
10545	INSULIN HUMULIN M4 CARTRIDGE 100 I/U	Insulin
19707	INSULIN HUMULIN S (NEUTRAL SOLUBLE)	Insulin
7861	INSULIN HUMULIN S (NEUTRAL) CARTRIDGE 100 I/U	Insulin
14504	INSULIN HYPURIN PROTAMINE ZINC 100 I/U INJ	Insulin

Carr MJ, et al. BMJ Qual Saf 2022; 31:503–514. doi: 10.1136/bmjqs-2021-013613

16209	INSULIN HYPURIN SOLUBLE 100 I/U INJ	Insulin
24866	INSULIN INSULATARD (LEO RETARD) 40 I/U INJ	Insulin
15624	INSULIN ISOPHANE (HIGHLY PURIFIED) 100 I/U INJ	Insulin
7783	INSULIN ISOPHANE (HUMAN) 100 I/U INJ	Insulin
10691	INSULIN ISOPHANE (NPH) 100 I/U INJ	Insulin
23003	INSULIN ISOPHANE (NPH) 40 I/U	Insulin
22823	INSULIN ISOPHANE (PURIFIED) 100 I/U INJ	Insulin
8376	INSULIN ISOPHANE 100 I/U	Insulin
24722	INSULIN ISOPHANE 50%/NEUTRAL 50% 100 I/U INJ	Insulin
8354	INSULIN ISOPHANE 70%/NEUTRAL 30% 100 I/U INJ	Insulin
2808	INSULIN LENTARD INJ	Insulin
7959	INSULIN MIXTARD 30/70 40 I/U INJ	Insulin
15040	INSULIN MONOPHANE (ISOPHANE) 100 I/U INJ	Insulin
7757	INSULIN NEULENTE (ZINC SUSP)(PURIFIED) 100 I/U INJ	Insulin
7763	INSULIN NEUPHANE (ISOPHANE)(PURIFIED) 100 I/U INJ	Insulin
7764	INSULIN NEUSULIN (NEUTRAL)(PURIFIED) 100 I/U INJ	Insulin
7765	INSULIN NEUTRAL (HUMAN) 100 I/U INJ	Insulin
18645	INSULIN NEUTRAL (PURIFIED) 100 I/U INJ	Insulin
1645	INSULIN NOVO ACTRAPID MC 100 I/U INJ	Insulin
19829	INSULIN NOVO MONOTARD MC	Insulin
1643	INSULIN NOVO MONOTARD MC 100 I/U INJ	Insulin
4248	INSULIN NOVO ULTRATARD MC 100 I/U INJ	Insulin
22806	INSULIN PORK ACTRAPID	Insulin
21945	INSULIN PORK INSULATARD	Insulin
24845	INSULIN PUR-IN ISOPHANE 100 I/U INJ	Insulin
28978	INSULIN PUR-IN MIX 15/85 100 I/U INJ	Insulin
31267	INSULIN PUR-IN MIX 50/50 100 I/U INJ	Insulin
12060	INSULIN QUICKSOL (SOLUBLE NEUTRAL) 100 I/U INJ	Insulin
8839	INSULIN SEMITARD 100 I/U INJ	Insulin
8838	INSULIN SEMITARD 40 I/U INJ	Insulin
9079	INSULIN SOLUBLE 100 I/U INJ	Insulin
20196	INSULIN SOLUBLE 40 I/U INJ	Insulin
18301	INSULIN SOLUBLE INJ I/U^2	Insulin
24485	INSULIN ZINC ANIMAL SUSPENSION	Insulin
28723	INSULIN ZINC BOVINE SUSPENSION	Insulin
12244	INSULIN ZINC BOVINE susp 100 I/U INJ	Insulin
8646	INSULIN ZINC CRYSTALLINE susp 100 I/U INJ	Insulin
30861	INSULIN ZINC HUMAN SUSPENSION	Insulin
22496	INSULIN ZINC LENTE PURIFIED SUSPENSION	Insulin
26784	INSULIN ZINC SEMILENTE SUSP BP 100 I/U INJ	Insulin
8895	Initard 50/50 100unit/ml Injection (Novo Nordisk Ltd)	Insulin
1886	Insulatard 100iu/ml GE injection (Novo Nordisk Ltd)	Insulin
33966	Insulatard 100unit/ml Injection (Novo Nordisk Ltd)	Insulin
14928	Insulatard 100units/ml suspension for injection 10ml vials (Novo Nordisk Ltd)	Insulin
71118	Insulatard 100units/ml suspension for injection 10ml vials (Waymade Healthcare Plc)	Insulin
5891	Insulatard FlexPen 100units/ml suspension for injection (Novo Nordisk Ltd)	Insulin

10208	Insulatard InnoLet 100units/ml suspension for injection 3ml pre-filled pen (Novo Nordisk Ltd)	Insulin
71428	Insulatard InnoLet 100units/ml suspension for injection 3ml pre-filled pen (Waymade Healthcare Plc)	Insulin
1595	Insulatard NovoLet 100units/ml suspension for injection (Novo Nordisk Ltd)	Insulin
14290	Insulatard Penfill 100units/ml suspension for injection 3ml cartridges (Novo Nordisk Ltd)	Insulin
52748	Insulatard Penfill 100units/ml suspension for injection 3ml cartridges (Waymade Healthcare Plc)	Insulin
9737	Insulatard innolet 100iu/ml Injection (Novo Nordisk Ltd)	Insulin
1593	Insulatard penfill 100 100iu/ml Penfill (Novo Nordisk Ltd)	Insulin
62180	Insulin aspart 100units/ml solution for injection 1.6ml cartridges	Insulin
29567	Insulin aspart 100units/ml solution for injection 10ml vials	Insulin
16142	Insulin aspart 100units/ml solution for injection 3ml cartridges	Insulin
19877	Insulin aspart 100units/ml solution for injection 3ml pre-filled disposable devices	Insulin
24795	Insulin aspart biphasic 30/70 100units/ml suspension for injection 3ml cartridges	Insulin
23099	Insulin aspart biphasic 30/70 100units/ml suspension for injection 3ml pre-filled disposable devices	Insulin
6447	Insulin aspart human pyr 100 iu/ml Injection	Insulin
13416	Insulin biphasic 100 units/ml Injection	Insulin
10067	Insulin biphasic aspart human pyr 30:70; 100 units/ml Injection	Insulin
33167	Insulin biphasic isophane human crb 25:75; 100 units/ml Injection	Insulin
54462	Insulin biphasic isophane human emp 25:75; 100 units/ml Injection	Insulin
13837	Insulin biphasic isophane human prb 10:90; 100 units/ml Injection	Insulin
14644	Insulin biphasic isophane human prb 20:80; 100 units/ml Injection	Insulin
29837	Insulin biphasic isophane human prb 25:75; 100 units/ml Injection	Insulin
9341	Insulin biphasic isophane human prb 30:70; 100 units/ml Injection	Insulin
21374	Insulin biphasic isophane human prb 40:60; 100 units/ml Injection	Insulin
21110	Insulin biphasic isophane human prb 50:50; 100 units/ml Injection	Insulin
14649	Insulin biphasic isophane human pyr 10:90; 100 units/ml Injection	Insulin
11055	Insulin biphasic isophane human pyr 20:80; 100 units/ml Injection	Insulin
11056	Insulin biphasic isophane human pyr 30:70; 100 units/ml Injection	Insulin
21395	Insulin biphasic isophane human pyr 40:60; 100 units/ml Injection	Insulin
66335	Insulin biphasic isophane porcine 50:50; 100 units/ml Injection	Insulin
5250	Insulin biphasic lispro human prb 25:75; 100 units/ml Injection	Insulin
27177	Insulin biphasic lispro human prb 50:50; 100 units/ml Injection	Insulin
55907	Insulin degludec 100units/ml solution for injection 3ml cartridges	Insulin
55687	Insulin degludec 100units/ml solution for injection 3ml pre-filled disposable devices	Insulin
56691	Insulin degludec 200units/ml solution for injection 3ml pre-filled disposable devices	Insulin
10184	Insulin detemir 100 iu/ml Solution for injection	Insulin
14301	Insulin detemir 100units/ml solution for injection 3ml cartridges	Insulin
14330	Insulin detemir 100units/ml solution for injection 3ml pre-filled disposable devices	Insulin
5953	Insulin glargine 100iu/ml Injection	Insulin
10259	Insulin glargine 100units/ml solution for injection 10ml vials	Insulin

7393	Insulin glargine 100units/ml solution for injection 3ml cartridges	Insulin
7400	Insulin glargine 100units/ml solution for injection 3ml pre-filled disposable devices	Insulin
64460	Insulin glargine 300units/ml solution for injection 1.5ml pre-filled disposable devices	Insulin
28442	Insulin glulisine 100unit/ml Solution for injection	Insulin
28101	Insulin glulisine 100units/ml solution for injection 10ml vials	Insulin
14299	Insulin glulisine 100units/ml solution for injection 3ml cartridges	Insulin
21590	Insulin glulisine 100units/ml solution for injection 3ml pre-filled disposable devices	Insulin
60951	Insulin human 100units/ml solution for injection 10ml vials	Insulin
67429	Insulin human 100units/ml solution for injection 3.15ml cartridges	Insulin
36355	Insulin human 1mg inhalation powder blisters	Insulin
36356	Insulin human 3mg inhalation powder blisters	Insulin
53710	Insulin human 500units/ml solution for injection 20ml vials	Insulin
25736	Insulin isophane biphasic human 10/90 100units/ml suspension for injection 3ml cartridges	Insulin
56857	Insulin isophane biphasic human 15/85 100units/ml suspension for injection 3ml cartridges	Insulin
25735	Insulin isophane biphasic human 20/80 100units/ml suspension for injection 3ml cartridges	Insulin
36194	Insulin isophane biphasic human 25/75 100units/ml suspension for injection 3ml cartridges	Insulin
44378	Insulin isophane biphasic human 25/75 100units/ml suspension for injection 3ml pre-filled disposable devices	Insulin
42954	Insulin isophane biphasic human 25/75 100units/ml suspension for injection 5ml vials	Insulin
21232	Insulin isophane biphasic human 30/70 100units/ml suspension for injection 10ml vials	Insulin
16152	Insulin isophane biphasic human 30/70 100units/ml suspension for injection 3ml cartridges	Insulin
19878	Insulin isophane biphasic human 30/70 100units/ml suspension for injection 3ml pre-filled disposable devices	Insulin
21422	Insulin isophane biphasic human 40/60 100units/ml suspension for injection 3ml cartridges	Insulin
22697	Insulin isophane biphasic human 50/50 100units/ml suspension for injection 1.5ml cartridges	Insulin
28096	Insulin isophane biphasic human 50/50 100units/ml suspension for injection 3ml cartridges	Insulin
41120	Insulin isophane biphasic human 50/50 100units/ml suspension for injection 3ml pre-filled disposable devices	Insulin
33232	Insulin isophane biphasic human 50/50 100units/ml suspension for injection 5ml vials	Insulin
14619	Insulin isophane biphasic porcine 30/70 100units/ml suspension for injection 1.5ml cartridges	Insulin
27280	Insulin isophane biphasic porcine 30/70 100units/ml suspension for injection 10ml vials	Insulin
36031	Insulin isophane biphasic porcine 30/70 100units/ml suspension for injection 3ml cartridges	Insulin
15484	Insulin isophane bovine 100units/ml suspension for injection 1.5ml cartridges	Insulin
18590	Insulin isophane bovine 100units/ml suspension for injection 10ml vials	Insulin

Carr MJ, *et al. BMJ Qual Saf* 2022; 31:503–514. doi: 10.1136/bmjqs-2021-013613

36066	Insulin isophane bovine 100units/ml suspension for injection 3ml cartridges	Insulin
10175	Insulin isophane human 100units/ml suspension for injection 1.5ml cartridges	Insulin
55517	Insulin isophane human 100units/ml suspension for injection 10ml vials	Insulin
10207	Insulin isophane human 100units/ml suspension for injection 3ml cartridges	Insulin
25812	Insulin isophane human 100units/ml suspension for injection 3ml pre-filled disposable devices	Insulin
59500	Insulin isophane human 100units/ml suspension for injection 5ml vials	Insulin
15961	Insulin isophane human crb 100iu/ml Injection	Insulin
13729	Insulin isophane human emp 100unit/ml Injection	Insulin
11080	Insulin isophane human prb 100iu/ml Injection	Insulin
14925	Insulin isophane human vial 100unit/ml Sterile suspension injection	Insulin
4247	Insulin isophane porcine 100units/ml suspension for injection 1.5ml cartridges	Insulin
7350	Insulin isophane porcine 100units/ml suspension for injection 10ml vials	Insulin
30686	Insulin isophane porcine 100units/ml suspension for injection 3ml cartridges	Insulin
5214	Insulin lispro 100units/ml solution for injection 1.5ml cartridges	Insulin
26060	Insulin lispro 100units/ml solution for injection 10ml vials	Insulin
14313	Insulin lispro 100units/ml solution for injection 3ml cartridges	Insulin
14362	Insulin lispro 100units/ml solution for injection 3ml pre-filled disposable devices	Insulin
74479	Insulin lispro 100units/ml solution for injection 3ml pre-filled pen (Sanofi)	Insulin
43953	Insulin lispro biphasic 25/75 100units/ml suspension for injection 10ml vials	Insulin
28185	Insulin lispro biphasic 25/75 100units/ml suspension for injection 3ml cartridges	Insulin
31258	Insulin lispro biphasic 25/75 100units/ml suspension for injection 3ml pre-filled disposable devices	Insulin
36146	Insulin lispro biphasic 50/50 100units/ml suspension for injection 3ml cartridges	Insulin
35701	Insulin lispro biphasic 50/50 100units/ml suspension for injection 3ml pre-filled disposable devices	Insulin
14505	Insulin protamine zinc bovine 100units/ml suspension for injection 10ml vials	Insulin
10572	Insulin soluble bovine 100unit/ml Injection	Insulin
18592	Insulin soluble bovine 100units/ml solution for injection 10ml vials	Insulin
14938	Insulin soluble bovine cartridge 100unit/ml Solution for injection	Insulin
27402	Insulin soluble human 100units/ml solution for injection 10ml vials	Insulin
16129	Insulin soluble human 100units/ml solution for injection 3ml cartridges	Insulin
36430	Insulin soluble human 100units/ml solution for injection 3ml pre-filled disposable devices	Insulin
26621	Insulin soluble human crb 100iu/ml Injection	Insulin
15710	Insulin soluble human emp 100unit/ml Injection	Insulin
12654	Insulin soluble human prb 100unit/ml Injection	Insulin
12638	Insulin soluble human pyr 100unit/ml Injection	Insulin
4129	Insulin soluble porcine 100units/ml solution for injection 1.5ml cartridges	Insulin
27396	Insulin soluble porcine 100units/ml solution for injection 10ml vials	Insulin
25479	Insulin soluble porcine 100units/ml solution for injection 3ml cartridges	Insulin
18931	Insulin zinc crystalline human 100units/ml suspension for injection 10ml vials	Insulin
12035	Insulin zinc mixed bovine 100units/ml suspension for injection 10ml vials	Insulin
16700	Insulin zinc mixed bovine vial 100unit/ml Sterile suspension injection	Insulin
18461	Insulin zinc mixed human 100units/ml suspension for injection 10ml vials	Insulin
9376	Insulin zinc suspension crystalline human pyr 100unit/ml long acting Injection	Insulin
41834	Insulin zinc suspension lente 100iu/ml Injection (Celltech Pharma Europe Ltd)	Insulin

26498	Insulin zinc suspension mixed bovine and porcine 100unit/ml Injection	Insulin
8322	Insulin zinc suspension mixed human pyr 100unit/ml Injection	Insulin
44251	Insulin zinc suspension mixed porcine 100unit/ml Injection	Insulin
27461	Insuman Basal 100units/ml suspension for injection 3ml cartridges (Sanofi)	Insulin
23992	Insuman Basal 100units/ml suspension for injection 3ml pre-filled OptiSet pen (Sanofi)	Insulin
46001	Insuman Basal 100units/ml suspension for injection 3ml pre-filled SoloStar pen (Sanofi)	Insulin
35468	Insuman Basal 100units/ml suspension for injection 5ml vials (Sanofi)	Insulin
45158	Insuman Comb 15 100units/ml suspension for injection 3ml cartridges (Sanofi)	Insulin
30819	Insuman Comb 15 100units/ml suspension for injection 3ml pre-filled OptiSet pen (Sanofi)	Insulin
24993	Insuman Comb 25 100units/ml suspension for injection 3ml cartridges (Sanofi)	Insulin
25133	Insuman Comb 25 100units/ml suspension for injection 3ml pre-filled OptiSet pen (Sanofi)	Insulin
44480	Insuman Comb 25 100units/ml suspension for injection 3ml pre-filled SoloStar pen (Sanofi)	Insulin
24002	Insuman Comb 25 100units/ml suspension for injection 5ml vials (Sanofi)	Insulin
35253	Insuman Comb 50 100units/ml suspension for injection 3ml cartridges (Sanofi)	Insulin
31205	Insuman Comb 50 100units/ml suspension for injection 3ml pre-filled OptiSet pen (Sanofi)	Insulin
22983	Insuman Rapid 100units/ml solution for injection 3ml cartridges (Sanofi)	Insulin
23993	Insuman Rapid 100units/ml solution for injection 3ml pre-filled OptiSet pen (Sanofi)	Insulin
5501	Insuman basal 100iu/ml Injection (Aventis Pharma)	Insulin
20422	Insuman comb 15 100iu/ml Injection (Aventis Pharma)	Insulin
15199	Insuman comb 25 100iu/ml Injection (Aventis Pharma)	Insulin
21554	Insuman comb 50 100iu/ml Injection (Aventis Pharma)	Insulin
22945	Insuman rapid 100iu/ml Injection (Aventis Pharma)	Insulin
38422	Isophane 100iu/ml Injection (Celltech Pharma Europe Ltd)	Insulin
30236	Isophane insulin 100iu/ml Injection	Insulin
6057	Lantus 100iu/ml Injection (Aventis Pharma)	Insulin
66316	Lantus 100units/ml solution for injection 10ml vials (Mawdsley-Brooks & Company Ltd)	Insulin
7402	Lantus 100units/ml solution for injection 10ml vials (Sanofi)	Insulin
10225	Lantus 100units/ml solution for injection 3ml OptiClik cartridges (Sanofi)	Insulin
50633	Lantus 100units/ml solution for injection 3ml cartridges (Necessity Supplies Ltd)	Insulin
7266	Lantus 100units/ml solution for injection 3ml cartridges (Sanofi)	Insulin
71351	Lantus 100units/ml solution for injection 3ml cartridges (Waymade Healthcare Plc)	Insulin
74862	Lantus 100units/ml solution for injection 3ml pre-filled OptiSet pen (Mawdsley-Brooks & Company Ltd)	Insulin
7237	Lantus 100units/ml solution for injection 3ml pre-filled OptiSet pen (Sanofi)	Insulin
56495	Lantus 100units/ml solution for injection 3ml pre-filled OptiSet pen (Waymade Healthcare Plc)	Insulin
49831	Lantus 100units/ml solution for injection 3ml pre-filled SoloStar pen (Necessity Supplies Ltd)	Insulin
36853	Lantus 100units/ml solution for injection 3ml pre-filled SoloStar pen (Sanofi)	Insulin
67230	Lantus 100units/ml solution for injection 3ml pre-filled SoloStar pen (Waymade	Insulin

	Healthcare Plc)	
4784	Lentard mc 100unit/ml Injection (Novo Nordisk Ltd)	Insulin
6958	Levemir FlexPen 100units/ml solution for injection 3ml pre-filled pen (Novo Nordisk Ltd)	Insulin
55618	Levemir FlexPen 100units/ml solution for injection 3ml pre-filled pen (Waymade Healthcare Plc)	Insulin
35260	Levemir InnoLet 100units/ml solution for injection 3ml pre-filled pen (Novo Nordisk Ltd)	Insulin
6965	Levemir Penfill 100units/ml solution for injection 3ml cartridges (Novo Nordisk Ltd)	Insulin
2456	Mixtard 10 NovoLet 100units/ml suspension for injection (Novo Nordisk Ltd)	Insulin
10245	Mixtard 10 Penfill 100units/ml suspension for injection 3ml cartridges (Novo Nordisk Ltd)	Insulin
5255	Mixtard 10 penfill 100 100iu/ml Penfill (Novo Nordisk Ltd)	Insulin
2455	Mixtard 20 NovoLet 100units/ml suspension for injection (Novo Nordisk Ltd)	Insulin
7319	Mixtard 20 Penfill 100units/ml suspension for injection 3ml cartridges (Novo Nordisk Ltd)	Insulin
3551	Mixtard 20 penfill 100 100iu/ml Penfill (Novo Nordisk Ltd)	Insulin
2929	Mixtard 30 100iu/ml GE injection (Novo Nordisk Ltd)	Insulin
7300	Mixtard 30 100units/ml suspension for injection 10ml vials (Novo Nordisk Ltd)	Insulin
60938	Mixtard 30 100units/ml suspension for injection 10ml vials (Waymade Healthcare Plc)	Insulin
5845	Mixtard 30 InnoLet 100units/ml suspension for injection 3ml pre-filled pen (Novo Nordisk Ltd)	Insulin
67267	Mixtard 30 InnoLet 100units/ml suspension for injection 3ml pre-filled pen (Waymade Healthcare Plc)	Insulin
2221	Mixtard 30 NovoLet 100units/ml suspension for injection (Novo Nordisk Ltd)	Insulin
7231	Mixtard 30 Penfill 100units/ml suspension for injection 3ml cartridges (Novo Nordisk Ltd)	Insulin
71340	Mixtard 30 Penfill 100units/ml suspension for injection 3ml cartridges (Waymade Healthcare Plc)	Insulin
2454	Mixtard 30 penfill 100 100iu/ml Penfill (Novo Nordisk Ltd)	Insulin
1805	Mixtard 30/70 100unit/ml Injection (Novo Nordisk Ltd)	Insulin
2812	Mixtard 40 NovoLet 100units/ml suspension for injection (Novo Nordisk Ltd)	Insulin
10244	Mixtard 40 Penfill 100units/ml suspension for injection 3ml cartridges (Novo Nordisk Ltd)	Insulin
3550	Mixtard 40 penfill 100 100iu/ml Penfill (Novo Nordisk Ltd)	Insulin
5933	Mixtard 50 NovoLet 100units/ml suspension for injection (Novo Nordisk Ltd)	Insulin
13277	Mixtard 50 Penfill 100units/ml suspension for injection 3ml cartridges (Novo Nordisk Ltd)	Insulin
67266	Mixtard 50 Penfill 100units/ml suspension for injection 3ml cartridges (Waymade Healthcare Plc)	Insulin
4790	Mixtard 50 penfill 100 100iu/ml Penfill (Novo Nordisk Ltd)	Insulin
1587	Monotard 100units/ml suspension for injection 10ml vials (Novo Nordisk Ltd)	Insulin
34031	Monotard mc 100unit/ml Injection (Novo Nordisk Ltd)	Insulin
47856	Neuphane 100unit/ml Injection (Wellcome Medical Division)	Insulin
47360	Neutral insulin 100unit/ml Injection (Celltech Pharma Europe Ltd)	Insulin
24593	Neutral insulin bovine 100unit/ml Injection	Insulin
7228	NovoMix 30 FlexPen 100units/ml suspension for injection 3ml pre-filled pen (Novo Nordisk Ltd)	Insulin

71369	NovoMix 30 FlexPen 100units/ml suspension for injection 3ml pre-filled pen (Sigma Pharmaceuticals Plc)	Insulin
74680	NovoMix 30 Penfill 100units/ml suspension for injection 3ml cartridges (DE Pharmaceuticals)	Insulin
74020	NovoMix 30 Penfill 100units/ml suspension for injection 3ml cartridges (Mawdsley-Brooks & Company Ltd)	Insulin
7267	NovoMix 30 Penfill 100units/ml suspension for injection 3ml cartridges (Novo Nordisk Ltd)	Insulin
56489	NovoMix 30 Penfill 100units/ml suspension for injection 3ml cartridges (Waymade Healthcare Plc)	Insulin
6209	NovoRapid 100units/ml solution for injection 10ml vials (Novo Nordisk Ltd)	Insulin
67313	NovoRapid 100units/ml solution for injection 10ml vials (Sigma Pharmaceuticals Plc)	Insulin
67231	NovoRapid FlexPen 100units/ml solution for injection 3ml pre-filled pen (Dowelhurst Ltd)	Insulin
53118	NovoRapid FlexPen 100units/ml solution for injection 3ml pre-filled pen (Mawdsley-Brooks & Company Ltd)	Insulin
5892	NovoRapid FlexPen 100units/ml solution for injection 3ml pre-filled pen (Novo Nordisk Ltd)	Insulin
59533	NovoRapid FlexPen 100units/ml solution for injection 3ml pre-filled pen (Sigma Pharmaceuticals Plc)	Insulin
74179	NovoRapid FlexPen 100units/ml solution for injection 3ml pre-filled pen (Waymade Healthcare Plc)	Insulin
46666	NovoRapid FlexTouch 100units/ml solution for injection 3ml pre-filled pen (Novo Nordisk Ltd)	Insulin
11337	NovoRapid Novolet 100units/ml solution for injection (Novo Nordisk Ltd)	Insulin
53251	NovoRapid Penfill 100units/ml solution for injection 3ml cartridges (DE Pharmaceuticals)	Insulin
49108	NovoRapid Penfill 100units/ml solution for injection 3ml cartridges (Necessity Supplies Ltd)	Insulin
5021	NovoRapid Penfill 100units/ml solution for injection 3ml cartridges (Novo Nordisk Ltd)	Insulin
51743	NovoRapid Penfill 100units/ml solution for injection 3ml cartridges (Sigma Pharmaceuticals Plc)	Insulin
61845	NovoRapid PumpCart 100units/ml solution for injection 1.6ml cartridges (Novo Nordisk Ltd)	Insulin
6061	Novomix 30 30/70 100units/ml Injection (Novo Nordisk Ltd)	Insulin
17336	Novopen 100unit/ml Injection device (Novo Nordisk Ltd)	Insulin
41959	Penject 100unit/ml Injection device (Hypoguard Ltd)	Insulin
3439	Penmix 10/90 Pen (Novo Nordisk Ltd)	Insulin
3396	Penmix 10/90 Penfill (Novo Nordisk Ltd)	Insulin
2220	Penmix 20/80 Pen (Novo Nordisk Ltd)	Insulin
10484	Penmix 20/80 Penfill (Novo Nordisk Ltd)	Insulin
27614	Penmix 30/70 100iu/ml Injection (Novo Nordisk Ltd)	Insulin
1806	Penmix 30/70 100iu/ml Penfill (Novo Nordisk Ltd)	Insulin
21347	Penmix 40/60 100iu/ml Injection (Novo Nordisk Ltd)	Insulin
10887	Penmix 40/60 100iu/ml Penfill (Novo Nordisk Ltd)	Insulin
17731	Penmix 50/50 100iu/ml Injection (Novo Nordisk Ltd)	Insulin
8203	Penmix 50/50 100iu/ml Penfill (Novo Nordisk Ltd)	Insulin
9521	Pork Actrapid 100units/ml solution for injection 10ml vials (Novo Nordisk Ltd)	Insulin

1843	Pork Insulatard 100units/ml suspension for injection 10ml vials (Novo Nordisk Ltd)	Insulin
67279	Pork Insulatard 100units/ml suspension for injection 10ml vials (Waymade Healthcare Plc)	Insulin
2459	Pork Mixtard 30 100units/ml suspension for injection 10ml vials (Novo Nordisk Ltd)	Insulin
1842	Pork velosulin 100unit/ml Injection (Novo Nordisk Ltd)	Insulin
22058	Pur-in mix 15/85 Injection (C P Pharmaceuticals Ltd)	Insulin
26403	Pur-in mix 25/75 Injection (C P Pharmaceuticals Ltd)	Insulin
24846	Pur-in neutral 100unit/ml Injection (C P Pharmaceuticals Ltd)	Insulin
74970	Pur-n Isophane 100unit/ml Injection (C P Pharmaceuticals Ltd)	Insulin
4163	Rapitard MC 100unit/ml Injection (Novo Nordisk Ltd)	Insulin
12299	Semitard mc 100unit/ml Injection (Novo Nordisk Ltd)	Insulin
16682	Tempulin 100unit/ml Injection (Knoll Ltd)	Insulin
64354	Toujeo 300units/ml solution for injection 1.5ml pre-filled SoloStar pen (Sanofi)	Insulin
55462	Tresiba FlexTouch 100units/ml solution for injection 3ml pre-filled pen (Novo Nordisk Ltd)	Insulin
55234	Tresiba FlexTouch 200units/ml solution for injection 3ml pre-filled pen (Novo Nordisk Ltd)	Insulin
55910	Tresiba Penfill 100units/ml solution for injection 3ml cartridges (Novo Nordisk Ltd)	Insulin
1844	Ultratard 100units/ml suspension for injection 10ml vials (Novo Nordisk Ltd)	Insulin
4706	Velosulin 100units/ml solution for injection 10ml vials (Novo Nordisk Ltd)	Insulin
36513	Velosulin cartridge 100unit/ml Injection (Novo Nordisk Ltd)	Insulin
52203	Enyglid 0.5mg tablets (Consilient Health Ltd)	Meglitinide
5678	Nateglinide 120mg tablets	Meglitinide
5989	Nateglinide 180mg tablets	Meglitinide
11483	Nateglinide 60mg tablets	Meglitinide
11321	NovoNorm 1mg tablets (Novo Nordisk Ltd)	Meglitinide
74860	NovoNorm 1mg tablets (Waymade Healthcare Plc)	Meglitinide
11366	NovoNorm 2mg tablets (Novo Nordisk Ltd)	Meglitinide
11316	NovoNorm 500microgram tablets (Novo Nordisk Ltd)	Meglitinide
61925	NovoNorm 500microgram tablets (Waymade Healthcare Plc)	Meglitinide
36948	Prandin 0.5mg tablets (Novo Nordisk Ltd)	Meglitinide
36774	Prandin 1mg tablets (Novo Nordisk Ltd)	Meglitinide
35561	Prandin 2mg tablets (Novo Nordisk Ltd)	Meglitinide
9707	Repaglinide 1mg tablets	Meglitinide
9748	Repaglinide 2mg tablets	Meglitinide
9865	Repaglinide 500microgram tablets	Meglitinide
15955	Starlix 120mg tablets (Novartis Pharmaceuticals UK Ltd)	Meglitinide
27125	Starlix 180mg tablets (Novartis Pharmaceuticals UK Ltd)	Meglitinide
23945	Starlix 60mg tablets (Novartis Pharmaceuticals UK Ltd)	Meglitinide
57147	Bolamyn SR 1000mg tablets (Teva UK Ltd)	Metformin
39560	Bolamyn SR 500mg tablets (Teva UK Ltd)	Metformin
52221	Diagemet XL 500mg tablets (Thornton & Ross Ltd)	Metformin
55270	Duformin 500mg Tablet (Dumex Ltd)	Metformin
25678	Glucamet 500mg Tablet (Opus Pharmaceuticals Ltd)	Metformin
26258	Glucamet 850mg Tablet (Opus Pharmaceuticals Ltd)	Metformin

64939	Glucient SR 1000mg tablets (Consilient Health Ltd)	Metformin
47939	Glucient SR 500mg tablets (Consilient Health Ltd)	Metformin
70477	Glucient SR 750mg tablets (Consilient Health Ltd)	Metformin
40007	Glucophage 1000mg oral powder sachets (Merck Serono Ltd)	Metformin
40110	Glucophage 500mg oral powder sachets (Merck Serono Ltd)	Metformin
7166	Glucophage 500mg tablets (Merck Serono Ltd)	Metformin
7610	Glucophage 850mg tablets (Merck Serono Ltd)	Metformin
39729	Glucophage SR 1000mg tablets (Merck Serono Ltd)	Metformin
66136	Glucophage SR 1000mg tablets (Waymade Healthcare Plc)	Metformin
52634	Glucophage SR 500mg tablets (DE Pharmaceuticals)	Metformin
50570	Glucophage SR 500mg tablets (Lexon (UK) Ltd)	Metformin
49502	Glucophage SR 500mg tablets (Mawdsley-Brooks & Company Ltd)	Metformin
16044	Glucophage SR 500mg tablets (Merck Serono Ltd)	Metformin
59620	Glucophage SR 500mg tablets (Waymade Healthcare Plc)	Metformin
38400	Glucophage SR 750mg tablets (Merck Serono Ltd)	Metformin
20810	METFORMIN	Metformin
16213	METFORMIN 250 MG TAB	Metformin
7815	METFORMIN 800 MG TAB	Metformin
3252	METFORMIN HCl 500 MG TAB	Metformin
2928	METFORMIN HCl 850 MG TAB	Metformin
72107	Meijumet 500mg modified-release tablets (Medreich Plc)	Metformin
51080	Metabet SR 1000mg tablets (Actavis UK Ltd)	Metformin
46989	Metabet SR 1000mg tablets (Morningside Healthcare Ltd)	Metformin
53774	Metabet SR 500mg tablets (Actavis UK Ltd)	Metformin
45581	Metabet SR 500mg tablets (Morningside Healthcare Ltd)	Metformin
54442	Metformin (roi) 1000mg Tablet	Metformin
735	Metformin 100mg/ml Oral solution	Metformin
39598	Metformin 1g modified-release tablets	Metformin
49738	Metformin 1g modified-release tablets (A A H Pharmaceuticals Ltd)	Metformin
62824	Metformin 1g modified-release tablets (Actavis UK Ltd)	Metformin
72001	Metformin 1g modified-release tablets (DE Pharmaceuticals)	Metformin
65923	Metformin 1g modified-release tablets (Mawdsley-Brooks & Company Ltd)	Metformin
60074	Metformin 1g modified-release tablets (Waymade Healthcare Plc)	Metformin
40233	Metformin 1g oral powder sachets sugar free	Metformin
69221	Metformin 1g oral powder sachets sugar free (J M McGill Ltd)	Metformin
63307	Metformin 1g/5ml oral solution	Metformin
68589	Metformin 1g/5ml oral solution sugar free	Metformin
74021	Metformin 250mg/5ml oral solution	Metformin
72695	Metformin 500mg Tablet (Celltech Pharma Europe Ltd)	Metformin
72052	Metformin 500mg Tablet (Lagap)	Metformin
34135	Metformin 500mg Tablet (M & A Pharmachem Ltd)	Metformin
7048	Metformin 500mg modified-release tablets	Metformin
51135	Metformin 500mg modified-release tablets (A A H Pharmaceuticals Ltd)	Metformin
60968	Metformin 500mg modified-release tablets (Actavis UK Ltd)	Metformin
68203	Metformin 500mg modified-release tablets (Almus Pharmaceuticals Ltd)	Metformin
62144	Metformin 500mg modified-release tablets (DE Pharmaceuticals)	Metformin
53478	Metformin 500mg modified-release tablets (Kent Pharmaceuticals Ltd)	Metformin

62265	Metformin 500mg modified-release tablets (Mawdsley-Brooks & Company Ltd)	Metformin
65694	Metformin 500mg modified-release tablets (Waymade Healthcare Plc)	Metformin
39988	Metformin 500mg oral powder sachets sugar free	Metformin
23	Metformin 500mg tablets	Metformin
34323	Metformin 500mg tablets (A A H Pharmaceuticals Ltd)	Metformin
33087	Metformin 500mg tablets (Actavis UK Ltd)	Metformin
55711	Metformin 500mg tablets (Alliance Healthcare (Distribution) Ltd)	Metformin
48149	Metformin 500mg tablets (Almus Pharmaceuticals Ltd)	Metformin
51527	Metformin 500mg tablets (Boston Healthcare Ltd)	Metformin
50970	Metformin 500mg tablets (Bristol Laboratories Ltd)	Metformin
72046	Metformin 500mg tablets (DE Pharmaceuticals)	Metformin
34004	Metformin 500mg tablets (IVAX Pharmaceuticals UK Ltd)	Metformin
57457	Metformin 500mg tablets (Milpharm Ltd)	Metformin
34598	Metformin 500mg tablets (Mylan)	Metformin
52442	Metformin 500mg tablets (Pfizer Ltd)	Metformin
71012	Metformin 500mg tablets (Phoenix Healthcare Distribution Ltd)	Metformin
73892	Metformin 500mg tablets (Relonchem Ltd)	Metformin
34917	Metformin 500mg tablets (Teva UK Ltd)	Metformin
55739	Metformin 500mg tablets (Tillomed Laboratories Ltd)	Metformin
73285	Metformin 500mg tablets (Waymade Healthcare Plc)	Metformin
34504	Metformin 500mg tablets (Wockhardt UK Ltd)	Metformin
71198	Metformin 500mg tablets (Zanza Laboratories Ltd)	Metformin
53867	Metformin 500mg tablets (Zentiva)	Metformin
44250	Metformin 500mg/5ml Oral solution (Hillcross Pharmaceuticals Ltd)	Metformin
58051	Metformin 500mg/5ml oral solution	Metformin
11990	Metformin 500mg/5ml oral solution sugar free	Metformin
68214	Metformin 500mg/5ml oral solution sugar free (A A H Pharmaceuticals Ltd)	Metformin
73460	Metformin 500mg/5ml oral solution sugar free (Actavis UK Ltd)	Metformin
73808	Metformin 500mg/5ml oral solution sugar free (Actavis UK Ltd)	Metformin
73525	Metformin 500mg/5ml oral solution sugar free (Alliance Healthcare (Distribution) Ltd)	Metformin
71890	Metformin 500mg/5ml oral solution sugar free (Almus Pharmaceuticals Ltd)	Metformin
73303	Metformin 500mg/5ml oral solution sugar free (Colonis Pharma Ltd)	Metformin
70744	Metformin 500mg/5ml oral solution sugar free (Focus Pharmaceuticals Ltd)	Metformin
68389	Metformin 500mg/5ml oral solution sugar free (Pinewood Healthcare)	Metformin
43270	Metformin 500mg/5ml oral solution sugar free (Rosemont Pharmaceuticals Ltd)	Metformin
73673	Metformin 500mg/5ml oral solution sugar free (Sigma Pharmaceuticals Plc)	Metformin
58607	Metformin 500mg/5ml oral solution sugar free (Zentiva)	Metformin
60286	Metformin 500mg/5ml oral suspension	Metformin
38355	Metformin 750mg modified-release tablets	Metformin
74734	Metformin 850mg capsules	Metformin
93	Metformin 850mg tablets	Metformin
33674	Metformin 850mg tablets (A A H Pharmaceuticals Ltd)	Metformin
34836	Metformin 850mg tablets (Actavis UK Ltd)	Metformin
54898	Metformin 850mg tablets (Almus Pharmaceuticals Ltd)	Metformin
34020	Metformin 850mg tablets (IVAX Pharmaceuticals UK Ltd)	Metformin
62605	Metformin 850mg tablets (Kent Pharmaceuticals Ltd)	Metformin

50821	Metformin 850mg tablets (Pfizer Ltd)	Metformin
63045	Metformin 850mg tablets (Relonchem Ltd)	Metformin
34742	Metformin 850mg tablets (Teva UK Ltd)	Metformin
34697	Metformin 850mg tablets (Wockhardt UK Ltd)	Metformin
68636	Metformin 850mg/5ml oral solution sugar free	Metformin
31146	Metsol 500mg/5ml oral solution (Kappin Ltd)	Metformin
27501	Orabet 500mg Tablet (Lagap)	Metformin
42161	Orabet 500mg Tablet (Sandoz Ltd)	Metformin
61043	Sukkarto SR 1000mg tablets (Morningside Healthcare Ltd)	Metformin
61559	Sukkarto SR 500mg tablets (Morningside Healthcare Ltd)	Metformin
73254	Yaltormin SR 1000mg tablets (Wockhardt UK Ltd)	Metformin
73252	Yaltormin SR 500mg tablets (Wockhardt UK Ltd)	Metformin
73511	Yaltormin SR 750mg tablets (Wockhardt UK Ltd)	Metformin
60211	Canagliflozin 100mg tablets	SGLT2i
60073	Canagliflozin 100mg tablets	SGLT2i
60386	Canagliflozin 300mg tablets	SGLT2i
54182	Dapagliflozin 10mg tablets	SGLT2i
54265	Dapagliflozin 5mg tablets	SGLT2i
61756	Empagliflozin 10mg tablets	SGLT2i
62172	Empagliflozin 25mg tablets	SGLT2i
54203	Forxiga 10mg tablets (AstraZeneca UK Ltd)	SGLT2i
63516	Forxiga 10mg tablets (Waymade Healthcare Plc)	SGLT2i
54480	Forxiga 5mg tablets (AstraZeneca UK Ltd)	SGLT2i
60066	Invokana 100mg tablets (Janssen-Cilag Ltd)	SGLT2i
60430	Invokana 100mg tablets (Napp Pharmaceuticals Ltd)	SGLT2i
60379	Invokana 300mg tablets (Napp Pharmaceuticals Ltd)	SGLT2i
62760	Jardiance 10mg tablets (Boehringer Ingelheim Ltd)	SGLT2i
64217	Jardiance 25mg tablets (Boehringer Ingelheim Ltd)	SGLT2i
69654	Qtern 5mg/10mg tablets (AstraZeneca UK Ltd)	SGLT2i/DPP-4i
69540	Saxagliptin 5mg / Dapagliflozin 10mg tablets	SGLT2i/DPP-4i
63929	Canagliflozin 50mg / Metformin 1g tablets	SGLT2i/Metformin
64743	Canagliflozin 50mg / Metformin 850mg tablets	SGLT2i/Metformin
60012	Dapagliflozin 5mg / Metformin 1g tablets	SGLT2i/Metformin
63031	Dapagliflozin 5mg / Metformin 850mg tablets	SGLT2i/Metformin
65066	Empagliflozin 12.5mg / Metformin 1g tablets	SGLT2i/Metformin
66855	Empagliflozin 12.5mg / Metformin 850mg tablets	SGLT2i/Metformin
65057	Empagliflozin 5mg / Metformin 1g tablets	SGLT2i/Metformin
65344	Empagliflozin 5mg / Metformin 850mg tablets	SGLT2i/Metformin
66008	Synjardy 12.5mg/1000mg tablets (Boehringer Ingelheim Ltd)	SGLT2i/Metformin
70463	Synjardy 12.5mg/850mg tablets (Boehringer Ingelheim Ltd)	SGLT2i/Metformin
65083	Synjardy 5mg/1000mg tablets (Boehringer Ingelheim Ltd)	SGLT2i/Metformin
69370	Synjardy 5mg/850mg tablets (Boehringer Ingelheim Ltd)	SGLT2i/Metformin
66854	Vokanamet 50mg/1000mg tablets (Napp Pharmaceuticals Ltd)	SGLT2i/Metformin
60643	Xigduo 5mg/1000mg tablets (AstraZeneca UK Ltd)	SGLT2i/Metformin
65059	Xigduo 5mg/850mg tablets (AstraZeneca UK Ltd)	SGLT2i/Metformin
67056	Amaryl 1mg tablets (Lexon (UK) Ltd)	Sulphonylurea
7332	Amaryl 1mg tablets (Zentiva)	Sulphonylurea

Carr MJ, *et al. BMJ Qual Saf* 2022; 31:503–514. doi: 10.1136/bmjqs-2021-013613

7284	Amaryl 2mg tablets (Zentiva)	Sulphonylurea
7409	Amaryl 3mg tablets (Zentiva)	Sulphonylurea
11284	Amaryl 4mg tablets (Zentiva)	Sulphonylurea
70432	Bilxona 30mg modified-release tablets (Actavis UK Ltd)	Sulphonylurea
70490	Bilxona 60mg modified-release tablets (Actavis UK Ltd)	Sulphonylurea
16602	Calabren 2.5mg Tablet (Berk Pharmaceuticals Ltd)	Sulphonylurea
26218	Calabren 5mg Tablet (Berk Pharmaceuticals Ltd)	Sulphonylurea
22614	DAONIL 10 MG TAB	Sulphonylurea
45831	Dacadis MR 30mg tablets (Mylan)	Sulphonylurea
57601	Daonil 5mg tablets (Dowelhurst Ltd)	Sulphonylurea
7744	Daonil 5mg tablets (Sanofi)	Sulphonylurea
4862	Diabetamide 2.5mg tablets (Ashbourne Pharmaceuticals Ltd)	Sulphonylurea
21832	Diabetamide 5mg tablets (Ashbourne Pharmaceuticals Ltd)	Sulphonylurea
21892	Diaglyk 80mg tablets (Ashbourne Pharmaceuticals Ltd)	Sulphonylurea
11695	Diamicron 30mg MR tablets (Servier Laboratories Ltd)	Sulphonylurea
1964	Diamicron 80mg tablets (Servier Laboratories Ltd)	Sulphonylurea
33562	Duclazide 80mg Tablet (Dumex Ltd)	Sulphonylurea
44473	Edicil MR 30mg tablets (Teva UK Ltd)	Sulphonylurea
8976	Euglucon 2.5mg tablets (Aventis Pharma)	Sulphonylurea
13331	Euglucon 5mg tablets (Sanofi)	Sulphonylurea
41898	GLIBENCLAMIDE	Sulphonylurea
75008	Glibenclamide 2.5mg Tablet (Berk Pharmaceuticals Ltd)	Sulphonylurea
2219	Glibenclamide 2.5mg tablets	Sulphonylurea
34676	Glibenclamide 2.5mg tablets (A A H Pharmaceuticals Ltd)	Sulphonylurea
34706	Glibenclamide 2.5mg tablets (IVAX Pharmaceuticals UK Ltd)	Sulphonylurea
41593	Glibenclamide 2.5mg tablets (Teva UK Ltd)	Sulphonylurea
34507	Glibenclamide 2.5mg tablets (Wockhardt UK Ltd)	Sulphonylurea
1254	Glibenclamide 5mg tablets	Sulphonylurea
41559	Glibenclamide 5mg tablets (A A H Pharmaceuticals Ltd)	Sulphonylurea
41558	Glibenclamide 5mg tablets (Teva UK Ltd)	Sulphonylurea
34563	Glibenclamide 5mg tablets (Wockhardt UK Ltd)	Sulphonylurea
21424	Glibenclamide 5mg/5ml oral suspension	Sulphonylurea
12513	Glibenese 5mg tablets (Pfizer Ltd)	Sulphonylurea
58882	Gliclazide 120mg/5ml oral suspension	Sulphonylurea
69669	Gliclazide 160mg/5ml oral suspension	Sulphonylurea
5627	Gliclazide 30mg modified-release tablets	Sulphonylurea
53288	Gliclazide 30mg modified-release tablets (A A H Pharmaceuticals Ltd)	Sulphonylurea
57830	Gliclazide 30mg modified-release tablets (Alliance Healthcare (Distribution) Ltd)	Sulphonylurea
68415	Gliclazide 30mg modified-release tablets (Phoenix Healthcare Distribution Ltd)	Sulphonylurea
43065	Gliclazide 40mg tablets	Sulphonylurea
61957	Gliclazide 40mg tablets (A A H Pharmaceuticals Ltd)	Sulphonylurea
72852	Gliclazide 40mg tablets (Accord Healthcare Ltd)	Sulphonylurea
71781	Gliclazide 40mg tablets (Teva UK Ltd)	Sulphonylurea
15374	Gliclazide 40mg/5ml oral suspension	Sulphonylurea
56437	Gliclazide 60mg modified-release tablets	Sulphonylurea
42790	Gliclazide 80mg Tablet (Merck Generics (UK) Ltd)	Sulphonylurea
45215	Gliclazide 80mg Tablet (Neo Laboratories Ltd)	Sulphonylurea

32	Gliclazide 80mg tablets	Sulphonylurea
17343	Gliclazide 80mg tablets (A A H Pharmaceuticals Ltd)	Sulphonylurea
51955	Gliclazide 80mg tablets (Accord Healthcare Ltd)	Sulphonylurea
31212	Gliclazide 80mg tablets (Actavis UK Ltd)	Sulphonylurea
63048	Gliclazide 80mg tablets (Alliance Healthcare (Distribution) Ltd)	Sulphonylurea
56008	Gliclazide 80mg tablets (Almus Pharmaceuticals Ltd)	Sulphonylurea
54764	Gliclazide 80mg tablets (Arrow Generics Ltd)	Sulphonylurea
68819	Gliclazide 80mg tablets (Bristol Laboratories Ltd)	Sulphonylurea
34932	Gliclazide 80mg tablets (Genus Pharmaceuticals Ltd)	Sulphonylurea
34399	Gliclazide 80mg tablets (IVAX Pharmaceuticals UK Ltd)	Sulphonylurea
67781	Gliclazide 80mg tablets (Milpharm Ltd)	Sulphonylurea
29939	Gliclazide 80mg tablets (Mylan)	Sulphonylurea
36856	Gliclazide 80mg tablets (Sandoz Ltd)	Sulphonylurea
48056	Gliclazide 80mg tablets (Sovereign Medical Ltd)	Sulphonylurea
60495	Gliclazide 80mg tablets (Teva UK Ltd)	Sulphonylurea
21564	Gliclazide 80mg tablets (Wockhardt UK Ltd)	Sulphonylurea
47074	Gliclazide 80mg/5ml oral suspension	Sulphonylurea
55862	Gliclazide Oral solution	Sulphonylurea
5276	Glimepiride 1mg tablets	Sulphonylurea
40365	Glimepiride 1mg tablets (Actavis UK Ltd)	Sulphonylurea
71477	Glimepiride 1mg tablets (Alliance Healthcare (Distribution) Ltd)	Sulphonylurea
71667	Glimepiride 1mg tablets (Teva UK Ltd)	Sulphonylurea
5353	Glimepiride 2mg tablets	Sulphonylurea
66399	Glimepiride 2mg tablets (A A H Pharmaceuticals Ltd)	Sulphonylurea
62014	Glimepiride 2mg tablets (Accord Healthcare Ltd)	Sulphonylurea
71476	Glimepiride 2mg tablets (Alliance Healthcare (Distribution) Ltd)	Sulphonylurea
6337	Glimepiride 3mg tablets	Sulphonylurea
71665	Glimepiride 3mg tablets (Alliance Healthcare (Distribution) Ltd)	Sulphonylurea
5316	Glimepiride 4mg tablets	Sulphonylurea
61311	Glimepiride 4mg tablets (Sigma Pharmaceuticals Plc)	Sulphonylurea
68675	Glimepiride 4mg tablets (Somex Pharma)	Sulphonylurea
68289	Glimepiride 4mg tablets (Waymade Healthcare Plc)	Sulphonylurea
547	Glipizide 2.5mg tablets	Sulphonylurea
5636	Glipizide 5mg tablets	Sulphonylurea
34802	Glipizide 5mg tablets (IVAX Pharmaceuticals UK Ltd)	Sulphonylurea
29326	Glipizide 5mg tablets (Mylan)	Sulphonylurea
44304	Glyconon 500mg Tablet (DDSA Pharmaceuticals Ltd)	Sulphonylurea
62034	Laaglyda MR 60mg tablets (Consilient Health Ltd)	Sulphonylurea
25636	Libanil 2.5mg Tablet (Approved Prescription Services Ltd)	Sulphonylurea
31474	Libanil 5mg Tablet (Approved Prescription Services Ltd)	Sulphonylurea
28708	Malix 2.5mg Tablet (Lagap)	Sulphonylurea
30460	Malix 5mg Tablet (Lagap)	Sulphonylurea
17706	Minodiab 2.5mg tablets (Pfizer Ltd)	Sulphonylurea
17698	Minodiab 5mg tablets (Pfizer Ltd)	Sulphonylurea
47894	Nazdol MR 30mg tablets (Consilient Health Ltd)	Sulphonylurea
40425	Nazdol MR 30mg tablets (Teva UK Ltd)	Sulphonylurea
44738	Niddaryl 1mg tablets (Dee Pharmaceuticals Ltd)	Sulphonylurea

Carr MJ, et al. BMJ Qual Saf 2022; 31:503–514. doi: 10.1136/bmjqs-2021-013613

21870	RASTINON	Sulphonylurea
12455	Rastinon 500mg Tablet (Hoechst Marion Roussel)	Sulphonylurea
7912	Semi-Daonil 2.5mg tablets (Sanofi)	Sulphonylurea
22636	TOLBUTAMIDE 1 GM TAB	Sulphonylurea
16211	TOLBUTAMIDE 100 MG TAB	Sulphonylurea
9108	TOLBUTAMIDE 250 MG TAB	Sulphonylurea
1965	Tolbutamide 500mg tablets	Sulphonylurea
34957	Tolbutamide 500mg tablets (A A H Pharmaceuticals Ltd)	Sulphonylurea
33673	Tolbutamide 500mg tablets (Actavis UK Ltd)	Sulphonylurea
46927	Tolbutamide 500mg tablets (Teva UK Ltd)	Sulphonylurea
11946	Tolbutamide 50mg/ml Injection	Sulphonylurea
63131	Ziclaseg 30mg modified-release tablets (Lupin Healthcare (UK) Ltd)	Sulphonylurea
43465	Zicron 40mg tablets (Bristol Laboratories Ltd)	Sulphonylurea
56376	Rosiglitazone 4mg with glimepiride 4mg tablet	Sulphonylurea/TZD
20287	Actos 15mg tablets (Takeda UK Ltd)	TZD
20889	Actos 30mg tablets (Takeda UK Ltd)	TZD
19472	Actos 45mg tablets (Takeda UK Ltd)	TZD
48120	Avandia 2mg Tablet (GlaxoSmithKline UK Ltd)	TZD
9662	Avandia 4mg tablets (GlaxoSmithKline UK Ltd)	TZD
15232	Avandia 8mg tablets (GlaxoSmithKline UK Ltd)	TZD
68934	Diabiom 30mg tablets (Tillomed Laboratories Ltd)	TZD
64900	Glidipion 30mg tablets (Actavis UK Ltd)	TZD
548	Pioglitazone 15mg tablets	TZD
56208	Pioglitazone 15mg tablets (A A H Pharmaceuticals Ltd)	TZD
65563	Pioglitazone 15mg tablets (Alliance Healthcare (Distribution) Ltd)	TZD
9699	Pioglitazone 30mg tablets	TZD
48139	Pioglitazone 30mg tablets (A A H Pharmaceuticals Ltd)	TZD
62426	Pioglitazone 30mg tablets (Accord Healthcare Ltd)	TZD
57659	Pioglitazone 30mg tablets (Actavis UK Ltd)	TZD
65562	Pioglitazone 30mg tablets (Alliance Healthcare (Distribution) Ltd)	TZD
69885	Pioglitazone 30mg tablets (Consilient Health Ltd)	TZD
63421	Pioglitazone 30mg tablets (Teva UK Ltd)	TZD
10051	Pioglitazone 45mg tablets	TZD
63046	Pioglitazone 45mg tablets (A A H Pharmaceuticals Ltd)	TZD
72163	Pioglitazone 45mg tablets (Alliance Healthcare (Distribution) Ltd)	TZD
63107	Pioglitazone 45mg tablets (Waymade Healthcare Plc)	TZD
37617	Rosiglitazone 2mg tablet	TZD
469	Rosiglitazone 4mg tablets	TZD
5227	Rosiglitazone 8mg tablets	TZD
17580	Avandamet 1mg/500mg tablets (GlaxoSmithKline UK Ltd)	TZD/Metformin
14164	Avandamet 2mg/1000mg tablets (GlaxoSmithKline UK Ltd)	TZD/Metformin
6855	Avandamet 2mg/500mg tablets (GlaxoSmithKline UK Ltd)	TZD/Metformin
7325	Avandamet 4mg/1000mg tablets (GlaxoSmithKline UK Ltd)	TZD/Metformin
31077	Competact 15mg/850mg tablets (Takeda UK Ltd)	TZD/Metformin
30316	Metformin with pioglitazone 850mg + 15mg Tablet	TZD/Metformin
11760	Metformin with rosiglitazone 1000mg + 2mg Tablet	TZD/Metformin
11737	Metformin with rosiglitazone 1000mg + 4mg Tablet	TZD/Metformin

Carr MJ, *et al. BMJ Qual Saf* 2022; 31:503–514. doi: 10.1136/bmjqs-2021-013613

11609	Metformin with rosiglitazone 500mg + 1mg Tablet	TZD/Metformin
11610	Metformin with rosiglitazone 500mg + 2mg Tablet	TZD/Metformin
18220	Pioglitazone 15mg / Metformin 850mg tablets	TZD/Metformin
11604	Rosiglitazone 1mg / Metformin 500mg tablets	TZD/Metformin
11717	Rosiglitazone 2mg / Metformin 1g tablets	TZD/Metformin
11601	Rosiglitazone 2mg / Metformin 500mg tablets	TZD/Metformin
7375	Rosiglitazone 4mg / Metformin 1g tablets	TZD/Metformin

Carr MJ, *et al. BMJ Qual Saf* 2022; 31:503–514. doi: 10.1136/bmjqs-2021-013613

Appendix 20. Product codes for prescriptions of antihypertensive medications

Product code	Term	Drug class
14477	Accupro 10mg tablets (Pfizer Ltd)	ACE inhibitor
14478	Accupro 20mg tablets (Pfizer Ltd)	ACE inhibitor
15096	Accupro 40mg tablets (Pfizer Ltd)	ACE inhibitor
7314	Accupro 5mg tablets (Pfizer Ltd)	ACE inhibitor
18269	Acepril 12.5mg tablets (Bristol-Myers Squibb Pharmaceuticals Ltd)	ACE inhibitor
3069	Acepril 25mg tablets (Bristol-Myers Squibb Pharmaceuticals Ltd)	ACE inhibitor
18325	Acepril 50mg tablets (Bristol-Myers Squibb Pharmaceuticals Ltd)	ACE inhibitor
25051	CAPOZIDE	ACE inhibitor
8923	CAPTOPRIL 100 MG TAB	ACE inhibitor
217	CAPTOPRIL 4 MG/ML LIQ	ACE inhibitor
24693	CARACE (SPECIAL COMPLIANCE PACK)	ACE inhibitor
22004	CARACE (SPECIAL COMPLIANCE PACK)	ACE inhibitor
23382	CARACE (SPECIAL COMPLIANCE PACK)	ACE inhibitor
3310	Capoten 12.5mg tablets (Bristol-Myers Squibb Pharmaceuticals Ltd)	ACE inhibitor
56509	Capoten 12.5mg tablets (Dowelhurst Ltd)	ACE inhibitor
1144	Capoten 25mg tablets (Bristol-Myers Squibb Pharmaceuticals Ltd)	ACE inhibitor
3839	Capoten 50mg tablets (Bristol-Myers Squibb Pharmaceuticals Ltd)	ACE inhibitor
25998	Captomex 12.5mg tablets (Actavis UK Ltd)	ACE inhibitor
28820	Captomex 25mg tablets (Actavis UK Ltd)	ACE inhibitor
24482	Captomex 50mg tablets (Actavis UK Ltd)	ACE inhibitor
66597	Captopril 10mg/5ml oral suspension	ACE inhibitor
33646	Captopril 12.5mg Tablet (Generics (UK) Ltd)	ACE inhibitor
34544	Captopril 12.5mg Tablet (IVAX Pharmaceuticals UK Ltd)	ACE inhibitor
1121	Captopril 12.5mg tablets	ACE inhibitor
46951	Captopril 12.5mg tablets (A A H Pharmaceuticals Ltd)	ACE inhibitor
41633	Captopril 12.5mg tablets (Actavis UK Ltd)	ACE inhibitor
70994	Captopril 12.5mg tablets (Sandoz Ltd)	ACE inhibitor
46957	Captopril 12.5mg tablets (Tillomed Laboratories Ltd)	ACE inhibitor
58195	Captopril 12.5mg/5ml oral solution	ACE inhibitor
35302	Captopril 12.5mg/5ml oral suspension	ACE inhibitor
69600	Captopril 1mg/5ml oral suspension	ACE inhibitor
74417	Captopril 20mg/5ml oral suspension	ACE inhibitor
74627	Captopril 25mg Tablet (C P Pharmaceuticals Ltd)	ACE inhibitor
43507	Captopril 25mg Tablet (Generics (UK) Ltd)	ACE inhibitor
34562	Captopril 25mg Tablet (IVAX Pharmaceuticals UK Ltd)	ACE inhibitor
34936	Captopril 25mg Tablet (Lagap)	ACE inhibitor
1143	Captopril 25mg tablets	ACE inhibitor
43649	Captopril 25mg tablets (A A H Pharmaceuticals Ltd)	ACE inhibitor
41617	Captopril 25mg tablets (Actavis UK Ltd)	ACE inhibitor
37655	Captopril 25mg tablets (Teva UK Ltd)	ACE inhibitor
52499	Captopril 25mg/5ml oral solution	ACE inhibitor
64739	Captopril 25mg/5ml oral solution (Special Order)	ACE inhibitor
59915	Captopril 25mg/5ml oral solution sugar free	ACE inhibitor
39512	Captopril 25mg/5ml oral suspension	ACE inhibitor

54544	Captopril 25mg/5ml oral suspension	ACE inhibitor
52293	Captopril 2mg capsules	ACE inhibitor
15958	Captopril 2mg tablets	ACE inhibitor
36742	Captopril 2mg/5ml oral suspension	ACE inhibitor
17633	Captopril 3mg/5ml oral solution	ACE inhibitor
69599	Captopril 500micrograms/5ml oral suspension	ACE inhibitor
34719	Captopril 50mg Tablet (Generics (UK) Ltd)	ACE inhibitor
34937	Captopril 50mg Tablet (IVAX Pharmaceuticals UK Ltd)	ACE inhibitor
1807	Captopril 50mg tablets	ACE inhibitor
73659	Captopril 50mg tablets (Kent Pharmaceuticals Ltd)	ACE inhibitor
41743	Captopril 50mg tablets (Teva UK Ltd)	ACE inhibitor
33336	Captopril 5mg/5ml Oral suspension (Eldon Laboratories)	ACE inhibitor
46851	Captopril 5mg/5ml oral solution	ACE inhibitor
59699	Captopril 5mg/5ml oral solution sugar free	ACE inhibitor
17624	Captopril 5mg/5ml oral suspension	ACE inhibitor
44527	Captopril 5mg/ml oral solution sugar free	ACE inhibitor
43432	Captopril 6.25mg tablets	ACE inhibitor
28486	Captopril 6.25mg/5ml oral suspension	ACE inhibitor
71277	Captopril 7.5mg/5ml oral suspension	ACE inhibitor
45228	Captopril capsules	ACE inhibitor
69192	Captopril oral solution	ACE inhibitor
16701	Carace 10mg tablets (Bristol-Myers Squibb Pharmaceuticals Ltd)	ACE inhibitor
10882	Carace 2.5mg tablets (Bristol-Myers Squibb Pharmaceuticals Ltd)	ACE inhibitor
12313	Carace 20mg tablets (Bristol-Myers Squibb Pharmaceuticals Ltd)	ACE inhibitor
14387	Carace 5mg tablets (Bristol-Myers Squibb Pharmaceuticals Ltd)	ACE inhibitor
12574	Cilazapril 1mg tablets	ACE inhibitor
12412	Cilazapril 2.5mg tablets	ACE inhibitor
15605	Cilazapril 250micrograms tablets	ACE inhibitor
12411	Cilazapril 500microgram tablets	ACE inhibitor
13026	Cilazapril 5mg tablets	ACE inhibitor
56506	Coversyl 2mg tablets (Dowelhurst Ltd)	ACE inhibitor
5612	Coversyl 2mg tablets (Servier Laboratories Ltd)	ACE inhibitor
67269	Coversyl 2mg tablets (Waymade Healthcare Plc)	ACE inhibitor
56508	Coversyl 4mg tablets (Dowelhurst Ltd)	ACE inhibitor
5800	Coversyl 4mg tablets (Servier Laboratories Ltd)	ACE inhibitor
14960	Coversyl 8mg tablets (Servier Laboratories Ltd)	ACE inhibitor
38026	Coversyl Arginine 10mg tablets (Servier Laboratories Ltd)	ACE inhibitor
38034	Coversyl Arginine 2.5mg tablets (Servier Laboratories Ltd)	ACE inhibitor
51807	Coversyl Arginine 5mg tablets (DE Pharmaceuticals)	ACE inhibitor
37965	Coversyl Arginine 5mg tablets (Servier Laboratories Ltd)	ACE inhibitor
50347	Coversyl Arginine 5mg tablets (Waymade Healthcare Plc)	ACE inhibitor
27890	ENALAPRIL MALEATE	ACE inhibitor
3509	ENALAPRIL MALEATE 40 MG TAB	ACE inhibitor
56850	Ecopace 12.5mg tablets (AMCo)	ACE inhibitor
32514	Ecopace 25mg tablets (AMCo)	ACE inhibitor
36753	Ednyt 10mg Tablet (Dominion Pharma)	ACE inhibitor
44657	Ednyt 2.5mg Tablet (Dominion Pharma)	ACE inhibitor

22439	Ednyt 20mg Tablet (Dominion Pharma)	ACE inhibitor
33057	Ednyt 5mg Tablet (Dominion Pharma)	ACE inhibitor
72017	Enalapril 1.25mg/5ml oral solution	ACE inhibitor
58751	Enalapril 1.25mg/5ml oral suspension	ACE inhibitor
73617	Enalapril 1.5mg/5ml oral suspension	ACE inhibitor
1299	Enalapril 10mg tablets	ACE inhibitor
32241	Enalapril 10mg tablets (A A H Pharmaceuticals Ltd)	ACE inhibitor
19208	Enalapril 10mg tablets (Actavis UK Ltd)	ACE inhibitor
52010	Enalapril 10mg tablets (Alliance Healthcare (Distribution) Ltd)	ACE inhibitor
63322	Enalapril 10mg tablets (Almus Pharmaceuticals Ltd)	ACE inhibitor
55903	Enalapril 10mg tablets (Dexcel-Pharma Ltd)	ACE inhibitor
68496	Enalapril 10mg tablets (Kent Pharmaceuticals Ltd)	ACE inhibitor
34952	Enalapril 10mg tablets (Mylan)	ACE inhibitor
61133	Enalapril 10mg tablets (Phoenix Healthcare Distribution Ltd)	ACE inhibitor
41746	Enalapril 10mg tablets (Sandoz Ltd)	ACE inhibitor
42894	Enalapril 10mg tablets (Teva UK Ltd)	ACE inhibitor
13755	Enalapril 10mg wafer	ACE inhibitor
66895	Enalapril 10mg/5ml oral suspension	ACE inhibitor
64062	Enalapril 1mg/5ml oral suspension	ACE inhibitor
448	Enalapril 2.5mg tablets	ACE inhibitor
41417	Enalapril 2.5mg tablets (A A H Pharmaceuticals Ltd)	ACE inhibitor
71737	Enalapril 2.5mg tablets (Almus Pharmaceuticals Ltd)	ACE inhibitor
73389	Enalapril 2.5mg tablets (DE Pharmaceuticals)	ACE inhibitor
64877	Enalapril 2.5mg tablets (Dexcel-Pharma Ltd)	ACE inhibitor
41694	Enalapril 2.5mg tablets (IVAX Pharmaceuticals UK Ltd)	ACE inhibitor
28127	Enalapril 2.5mg tablets (Teva UK Ltd)	ACE inhibitor
43563	Enalapril 2.5mg tablets (Zentiva)	ACE inhibitor
20188	Enalapril 2.5mg wafer	ACE inhibitor
57882	Enalapril 2.5mg/5ml oral suspension	ACE inhibitor
1904	Enalapril 20mg tablets	ACE inhibitor
33078	Enalapril 20mg tablets (A A H Pharmaceuticals Ltd)	ACE inhibitor
31716	Enalapril 20mg tablets (Actavis UK Ltd)	ACE inhibitor
53719	Enalapril 20mg tablets (Alliance Healthcare (Distribution) Ltd)	ACE inhibitor
71668	Enalapril 20mg tablets (Almus Pharmaceuticals Ltd)	ACE inhibitor
34768	Enalapril 20mg tablets (IVAX Pharmaceuticals UK Ltd)	ACE inhibitor
34712	Enalapril 20mg tablets (Kent Pharmaceuticals Ltd)	ACE inhibitor
59996	Enalapril 20mg tablets (Milpharm Ltd)	ACE inhibitor
34453	Enalapril 20mg tablets (Mylan)	ACE inhibitor
34798	Enalapril 20mg tablets (Sandoz Ltd)	ACE inhibitor
42902	Enalapril 20mg tablets (Teva UK Ltd)	ACE inhibitor
34953	Enalapril 20mg tablets (Zentiva)	ACE inhibitor
24041	Enalapril 20mg wafer	ACE inhibitor
74237	Enalapril 25mg/5ml oral solution	ACE inhibitor
50780	Enalapril 2mg/5ml oral solution	ACE inhibitor
57378	Enalapril 2mg/5ml oral suspension	ACE inhibitor
50334	Enalapril 4mg/5ml oral suspension	ACE inhibitor
34400	Enalapril 5mg Tablet (Dowelhurst Ltd)	ACE inhibitor

196	Enalapril 5mg tablets	ACE inhibitor
35794	Enalapril 5mg tablets (A A H Pharmaceuticals Ltd)	ACE inhibitor
62860	Enalapril 5mg tablets (DE Pharmaceuticals)	ACE inhibitor
53915	Enalapril 5mg tablets (Dexcel-Pharma Ltd)	ACE inhibitor
42908	Enalapril 5mg tablets (IVAX Pharmaceuticals UK Ltd)	ACE inhibitor
45217	Enalapril 5mg tablets (Kent Pharmaceuticals Ltd)	ACE inhibitor
60143	Enalapril 5mg tablets (Medreich Plc)	ACE inhibitor
46974	Enalapril 5mg tablets (Mylan)	ACE inhibitor
43411	Enalapril 5mg tablets (Sandoz Ltd)	ACE inhibitor
42901	Enalapril 5mg tablets (Teva UK Ltd)	ACE inhibitor
22708	Enalapril 5mg wafer	ACE inhibitor
37080	Enalapril 5mg/5ml oral solution	ACE inhibitor
50863	Enalapril 5mg/5ml oral solution (Drug Tariff Special Order)	ACE inhibitor
37087	Enalapril 5mg/5ml oral suspension	ACE inhibitor
52882	Enalapril 5mg/5ml oral suspension sugar free	ACE inhibitor
16708	Enalapril titration pack	ACE inhibitor
633	Fosinopril 10mg tablets	ACE inhibitor
5861	Fosinopril 20mg tablets	ACE inhibitor
8025	Gopten 1mg capsules (Abbott Laboratories Ltd)	ACE inhibitor
8026	Gopten 2mg capsules (Abbott Laboratories Ltd)	ACE inhibitor
29130	Gopten 4mg capsules (Abbott Laboratories Ltd)	ACE inhibitor
16710	Gopten 500microgram capsules (Abbott Laboratories Ltd)	ACE inhibitor
65389	Gopten 500microgram capsules (Waymade Healthcare Plc)	ACE inhibitor
12858	Imidapril 10mg tablets	ACE inhibitor
18219	Imidapril 20mg tablets	ACE inhibitor
16924	Imidapril 5mg tablets	ACE inhibitor
8830	Innovace 10mg tablets (Merck Sharp & Dohme Ltd)	ACE inhibitor
8106	Innovace 2.5mg tablets (Merck Sharp & Dohme Ltd)	ACE inhibitor
8105	Innovace 20mg tablets (Merck Sharp & Dohme Ltd)	ACE inhibitor
8800	Innovace 5mg tablets (Merck Sharp & Dohme Ltd)	ACE inhibitor
15085	Innovace Titration pack (Merck Sharp & Dohme Ltd)	ACE inhibitor
27871	Innovace melt 10mg Wafer (Merck Sharp & Dohme Ltd)	ACE inhibitor
29530	Innovace melt 2.5mg Wafer (Merck Sharp & Dohme Ltd)	ACE inhibitor
31587	Innovace melt 20mg Wafer (Merck Sharp & Dohme Ltd)	ACE inhibitor
11197	Innovace melt 5mg Wafer (Merck Sharp & Dohme Ltd)	ACE inhibitor
21943	Kaplon 12.5mg tablets (Teva UK Ltd)	ACE inhibitor
26995	Kaplon 25mg tablets (Teva UK Ltd)	ACE inhibitor
32048	Kaplon 50mg tablets (Teva UK Ltd)	ACE inhibitor
71562	Lisinopril 10mg Tablet (Niche Generics Ltd)	ACE inhibitor
65	Lisinopril 10mg tablets	ACE inhibitor
43416	Lisinopril 10mg tablets (A A H Pharmaceuticals Ltd)	ACE inhibitor
55639	Lisinopril 10mg tablets (Accord Healthcare Ltd)	ACE inhibitor
45300	Lisinopril 10mg tablets (Actavis UK Ltd)	ACE inhibitor
53271	Lisinopril 10mg tablets (Alliance Healthcare (Distribution) Ltd)	ACE inhibitor
47159	Lisinopril 10mg tablets (Almus Pharmaceuticals Ltd)	ACE inhibitor
54288	Lisinopril 10mg tablets (Arrow Generics Ltd)	ACE inhibitor
73672	Lisinopril 10mg tablets (Aurobindo Pharma Ltd)	ACE inhibitor

54928	Lisinopril 10mg tablets (Bristol Laboratories Ltd)	ACE inhibitor
63030	Lisinopril 10mg tablets (DE Pharmaceuticals)	ACE inhibitor
60010	Lisinopril 10mg tablets (Kent Pharmaceuticals Ltd)	ACE inhibitor
33977	Lisinopril 10mg tablets (Mylan)	ACE inhibitor
58863	Lisinopril 10mg tablets (Phoenix Healthcare Distribution Ltd)	ACE inhibitor
54037	Lisinopril 10mg tablets (Relonchem Ltd)	ACE inhibitor
32597	Lisinopril 10mg tablets (Sandoz Ltd)	ACE inhibitor
65102	Lisinopril 10mg tablets (Sigma Pharmaceuticals Plc)	ACE inhibitor
19223	Lisinopril 10mg tablets (Teva UK Ltd)	ACE inhibitor
58871	Lisinopril 10mg tablets (Waymade Healthcare Plc)	ACE inhibitor
57048	Lisinopril 10mg tablets (Zentiva)	ACE inhibitor
62564	Lisinopril 10mg/5ml oral solution	ACE inhibitor
63824	Lisinopril 10mg/5ml oral suspension	ACE inhibitor
277	Lisinopril 2.5mg tablets	ACE inhibitor
43412	Lisinopril 2.5mg tablets (A A H Pharmaceuticals Ltd)	ACE inhibitor
55896	Lisinopril 2.5mg tablets (Actavis UK Ltd)	ACE inhibitor
65536	Lisinopril 2.5mg tablets (Alliance Healthcare (Distribution) Ltd)	ACE inhibitor
58451	Lisinopril 2.5mg tablets (Almus Pharmaceuticals Ltd)	ACE inhibitor
67194	Lisinopril 2.5mg tablets (Bristol Laboratories Ltd)	ACE inhibitor
73716	Lisinopril 2.5mg tablets (Crescent Pharma Ltd)	ACE inhibitor
65985	Lisinopril 2.5mg tablets (DE Pharmaceuticals)	ACE inhibitor
58461	Lisinopril 2.5mg tablets (Kent Pharmaceuticals Ltd)	ACE inhibitor
65983	Lisinopril 2.5mg tablets (Lupin Healthcare (UK) Ltd)	ACE inhibitor
67075	Lisinopril 2.5mg tablets (Mawdsley-Brooks & Company Ltd)	ACE inhibitor
58682	Lisinopril 2.5mg tablets (Mylan)	ACE inhibitor
72038	Lisinopril 2.5mg tablets (Relonchem Ltd)	ACE inhibitor
43566	Lisinopril 2.5mg tablets (Sandoz Ltd)	ACE inhibitor
30921	Lisinopril 2.5mg tablets (Teva UK Ltd)	ACE inhibitor
66772	Lisinopril 2.5mg tablets (Waymade Healthcare Plc)	ACE inhibitor
60097	Lisinopril 2.5mg tablets (Zentiva)	ACE inhibitor
56279	Lisinopril 2.5mg/5ml oral solution	ACE inhibitor
58258	Lisinopril 2.5mg/5ml oral suspension	ACE inhibitor
69	Lisinopril 20mg tablets	ACE inhibitor
43413	Lisinopril 20mg tablets (A A H Pharmaceuticals Ltd)	ACE inhibitor
55002	Lisinopril 20mg tablets (Accord Healthcare Ltd)	ACE inhibitor
45324	Lisinopril 20mg tablets (Actavis UK Ltd)	ACE inhibitor
59111	Lisinopril 20mg tablets (Alliance Healthcare (Distribution) Ltd)	ACE inhibitor
67795	Lisinopril 20mg tablets (Almus Pharmaceuticals Ltd)	ACE inhibitor
61262	Lisinopril 20mg tablets (Bristol Laboratories Ltd)	ACE inhibitor
66622	Lisinopril 20mg tablets (DE Pharmaceuticals)	ACE inhibitor
63559	Lisinopril 20mg tablets (Kent Pharmaceuticals Ltd)	ACE inhibitor
34696	Lisinopril 20mg tablets (Mylan)	ACE inhibitor
53551	Lisinopril 20mg tablets (Phoenix Healthcare Distribution Ltd)	ACE inhibitor
69074	Lisinopril 20mg tablets (Relonchem Ltd)	ACE inhibitor
46979	Lisinopril 20mg tablets (Sandoz Ltd)	ACE inhibitor
55588	Lisinopril 20mg tablets (Sigma Pharmaceuticals Plc)	ACE inhibitor
19198	Lisinopril 20mg tablets (Teva UK Ltd)	ACE inhibitor

51433	Lisinopril 20mg tablets (Tillomed Laboratories Ltd)	ACE inhibitor
69269	Lisinopril 20mg tablets (Waymade Healthcare Plc)	ACE inhibitor
34799	Lisinopril 20mg tablets (Zentiva)	ACE inhibitor
78	Lisinopril 5mg tablets	ACE inhibitor
43418	Lisinopril 5mg tablets (A A H Pharmaceuticals Ltd)	ACE inhibitor
58294	Lisinopril 5mg tablets (Accord Healthcare Ltd)	ACE inhibitor
45337	Lisinopril 5mg tablets (Actavis UK Ltd)	ACE inhibitor
55456	Lisinopril 5mg tablets (Alliance Healthcare (Distribution) Ltd)	ACE inhibitor
45816	Lisinopril 5mg tablets (Almus Pharmaceuticals Ltd)	ACE inhibitor
53820	Lisinopril 5mg tablets (Arrow Generics Ltd)	ACE inhibitor
74155	Lisinopril 5mg tablets (Aurobindo Pharma Ltd)	ACE inhibitor
68247	Lisinopril 5mg tablets (Bristol Laboratories Ltd)	ACE inhibitor
66558	Lisinopril 5mg tablets (DE Pharmaceuticals)	ACE inhibitor
65416	Lisinopril 5mg tablets (Lupin Healthcare (UK) Ltd)	ACE inhibitor
70667	Lisinopril 5mg tablets (Mawdsley-Brooks & Company Ltd)	ACE inhibitor
34471	Lisinopril 5mg tablets (Mylan)	ACE inhibitor
52088	Lisinopril 5mg tablets (Phoenix Healthcare Distribution Ltd)	ACE inhibitor
60309	Lisinopril 5mg tablets (Relonchem Ltd)	ACE inhibitor
46975	Lisinopril 5mg tablets (Sandoz Ltd)	ACE inhibitor
68094	Lisinopril 5mg tablets (Sigma Pharmaceuticals Plc)	ACE inhibitor
19204	Lisinopril 5mg tablets (Teva UK Ltd)	ACE inhibitor
59109	Lisinopril 5mg tablets (Tillomed Laboratories Ltd)	ACE inhibitor
72336	Lisinopril 5mg tablets (Waymade Healthcare Plc)	ACE inhibitor
60232	Lisinopril 5mg tablets (Zentiva)	ACE inhibitor
11987	Lisinopril 5mg/5ml oral solution	ACE inhibitor
64902	Lisinopril 5mg/5ml oral solution sugar free	ACE inhibitor
37778	Lisinopril 5mg/5ml oral suspension	ACE inhibitor
54283	Lisinopril 5mg/5ml oral suspension (Special Order)	ACE inhibitor
20975	Lisinopril 7.5mg/5ml oral suspension	ACE inhibitor
54512	Lisinopril Oral solution	ACE inhibitor
41573	Lisopress 10mg tablets (Teva UK Ltd)	ACE inhibitor
41538	Lisopress 2.5mg tablets (Teva UK Ltd)	ACE inhibitor
41522	Lisopress 20mg tablets (Teva UK Ltd)	ACE inhibitor
41532	Lisopress 5mg tablets (Teva UK Ltd)	ACE inhibitor
32934	Lopace 10mg capsules (Discovery Pharmaceuticals)	ACE inhibitor
29627	Lopace 2.5mg capsules (Discovery Pharmaceuticals)	ACE inhibitor
28586	Lopace 5mg capsules (Discovery Pharmaceuticals)	ACE inhibitor
17120	Moexipril 15mg tablets	ACE inhibitor
15121	Moexipril 7.5mg tablets	ACE inhibitor
60349	Noyada 25mg/5ml oral solution (Martindale Pharmaceuticals Ltd)	ACE inhibitor
60823	Noyada 5mg/5ml oral solution (Martindale Pharmaceuticals Ltd)	ACE inhibitor
31810	Odrik 1mg capsules (Aventis Pharma)	ACE inhibitor
28902	Odrik 2mg capsules (Aventis Pharma)	ACE inhibitor
31307	Odrik 500microgram capsules (Aventis Pharma)	ACE inhibitor
2927	PERINDOPRIL/TERT-BUTYLAMINE 2 MG TAB	ACE inhibitor
28725	Perdix 15mg tablets (UCB Pharma Ltd)	ACE inhibitor
28724	Perdix 7.5mg tablets (UCB Pharma Ltd)	ACE inhibitor

Carr MJ, *et al. BMJ Qual Saf* 2022; 31:503–514. doi: 10.1136/bmjqs-2021-013613

72295	Perindopril 2mg Tablet (Neo Laboratories Ltd)	ACE inhibitor
50402	Perindopril 2mg Tablet (Servier Laboratories Ltd)	ACE inhibitor
37971	Perindopril arginine 10mg tablets	ACE inhibitor
37964	Perindopril arginine 2.5mg tablets	ACE inhibitor
37930	Perindopril arginine 5mg tablets	ACE inhibitor
72941	Perindopril erbumine 1mg/5ml oral suspension	ACE inhibitor
593	Perindopril erbumine 2mg tablets	ACE inhibitor
45319	Perindopril erbumine 2mg tablets (A A H Pharmaceuticals Ltd)	ACE inhibitor
67789	Perindopril erbumine 2mg tablets (Accord Healthcare Ltd)	ACE inhibitor
43813	Perindopril erbumine 2mg tablets (Actavis UK Ltd)	ACE inhibitor
59972	Perindopril erbumine 2mg tablets (Alliance Healthcare (Distribution) Ltd)	ACE inhibitor
68759	Perindopril erbumine 2mg tablets (Aurobindo Pharma Ltd)	ACE inhibitor
49491	Perindopril erbumine 2mg tablets (Consilient Health Ltd)	ACE inhibitor
66060	Perindopril erbumine 2mg tablets (DE Pharmaceuticals)	ACE inhibitor
70917	Perindopril erbumine 2mg tablets (Glenmark Pharmaceuticals Europe Ltd)	ACE inhibitor
58843	Perindopril erbumine 2mg tablets (Kent Pharmaceuticals Ltd)	ACE inhibitor
48049	Perindopril erbumine 2mg tablets (Mylan)	ACE inhibitor
56516	Perindopril erbumine 2mg tablets (Sandoz Ltd)	ACE inhibitor
56473	Perindopril erbumine 2mg tablets (Sigma Pharmaceuticals Plc)	ACE inhibitor
58874	Perindopril erbumine 2mg tablets (Somex Pharma)	ACE inhibitor
54899	Perindopril erbumine 2mg tablets (Teva UK Ltd)	ACE inhibitor
64602	Perindopril erbumine 2mg tablets (Waymade Healthcare Plc)	ACE inhibitor
97	Perindopril erbumine 4mg tablets	ACE inhibitor
33095	Perindopril erbumine 4mg tablets (A A H Pharmaceuticals Ltd)	ACE inhibitor
61270	Perindopril erbumine 4mg tablets (Accord Healthcare Ltd)	ACE inhibitor
48214	Perindopril erbumine 4mg tablets (Actavis UK Ltd)	ACE inhibitor
68021	Perindopril erbumine 4mg tablets (Alliance Healthcare (Distribution) Ltd)	ACE inhibitor
38510	Perindopril erbumine 4mg tablets (Apotex UK Ltd)	ACE inhibitor
59770	Perindopril erbumine 4mg tablets (Aurobindo Pharma Ltd)	ACE inhibitor
56162	Perindopril erbumine 4mg tablets (Consilient Health Ltd)	ACE inhibitor
57801	Perindopril erbumine 4mg tablets (Glenmark Pharmaceuticals Europe Ltd)	ACE inhibitor
56472	Perindopril erbumine 4mg tablets (Kent Pharmaceuticals Ltd)	ACE inhibitor
68381	Perindopril erbumine 4mg tablets (Mawdsley-Brooks & Company Ltd)	ACE inhibitor
65273	Perindopril erbumine 4mg tablets (Mylan)	ACE inhibitor
75024	Perindopril erbumine 4mg tablets (Phoenix Healthcare Distribution Ltd)	ACE inhibitor
75021	Perindopril erbumine 4mg tablets (Ranbaxy (UK) Ltd)	ACE inhibitor
48180	Perindopril erbumine 4mg tablets (Sandoz Ltd)	ACE inhibitor
60065	Perindopril erbumine 4mg tablets (Sigma Pharmaceuticals Plc)	ACE inhibitor
38285	Perindopril erbumine 4mg tablets (Teva UK Ltd)	ACE inhibitor
61117	Perindopril erbumine 4mg/5ml oral solution	ACE inhibitor
11983	Perindopril erbumine 4mg/5ml oral suspension	ACE inhibitor
6078	Perindopril erbumine 8mg tablets	ACE inhibitor
35731	Perindopril erbumine 8mg tablets (A A H Pharmaceuticals Ltd)	ACE inhibitor
59790	Perindopril erbumine 8mg tablets (Accord Healthcare Ltd)	ACE inhibitor
71004	Perindopril erbumine 8mg tablets (Accord Healthcare Ltd)	ACE inhibitor
57701	Perindopril erbumine 8mg tablets (Actavis UK Ltd)	ACE inhibitor
61693	Perindopril erbumine 8mg tablets (Aurobindo Pharma Ltd)	ACE inhibitor

54733	Perindopril erbumine 8mg tablets (Consilient Health Ltd)	ACE inhibitor
69016	Perindopril erbumine 8mg tablets (DE Pharmaceuticals)	ACE inhibitor
70916	Perindopril erbumine 8mg tablets (Glenmark Pharmaceuticals Europe Ltd)	ACE inhibitor
54942	Perindopril erbumine 8mg tablets (Mylan)	ACE inhibitor
53058	Perindopril erbumine 8mg tablets (Sandoz Ltd)	ACE inhibitor
45938	Perindopril erbumine 8mg tablets (Teva UK Ltd)	ACE inhibitor
54986	Perindopril erbumine 8mg/5ml oral suspension	ACE inhibitor
43012	Perindopril erbumine oral solution	ACE inhibitor
56079	Perindopril tosilate 10mg tablets	ACE inhibitor
57944	Perindopril tosilate 2.5mg tablets	ACE inhibitor
57333	Perindopril tosilate 5mg tablets	ACE inhibitor
23252	Pralenal 10 tablets (Opus Pharmaceuticals Ltd)	ACE inhibitor
42723	Pralenal 5 tablets (Opus Pharmaceuticals Ltd)	ACE inhibitor
3929	Quinapril 10mg tablets	ACE inhibitor
5159	Quinapril 20mg tablets	ACE inhibitor
38854	Quinapril 20mg/5ml oral solution	ACE inhibitor
9731	Quinapril 40mg tablets	ACE inhibitor
61292	Quinapril 40mg tablets (Mylan)	ACE inhibitor
6765	Quinapril 5mg tablets	ACE inhibitor
38899	Quinil 10mg tablets (Tillomed Laboratories Ltd)	ACE inhibitor
46365	Quinil 20mg tablets (Tillomed Laboratories Ltd)	ACE inhibitor
42285	Quinil 40mg tablets (Tillomed Laboratories Ltd)	ACE inhibitor
40355	Quinil 5mg tablets (Tillomed Laboratories Ltd)	ACE inhibitor
22882	RAMIPRIL	ACE inhibitor
65443	Ramipril 1.25mg Tablet (Sovereign Medical Ltd)	ACE inhibitor
147	Ramipril 1.25mg capsules	ACE inhibitor
55299	Ramipril 1.25mg capsules (A A H Pharmaceuticals Ltd)	ACE inhibitor
45264	Ramipril 1.25mg capsules (Actavis UK Ltd)	ACE inhibitor
56704	Ramipril 1.25mg capsules (Alliance Healthcare (Distribution) Ltd)	ACE inhibitor
67741	Ramipril 1.25mg capsules (Almus Pharmaceuticals Ltd)	ACE inhibitor
52399	Ramipril 1.25mg capsules (Kent Pharmaceuticals Ltd)	ACE inhibitor
70072	Ramipril 1.25mg capsules (Mawdsley-Brooks & Company Ltd)	ACE inhibitor
32857	Ramipril 1.25mg capsules (Teva UK Ltd)	ACE inhibitor
57073	Ramipril 1.25mg capsules (Waymade Healthcare Plc)	ACE inhibitor
34698	Ramipril 1.25mg capsules (Zentiva)	ACE inhibitor
761	Ramipril 1.25mg tablets	ACE inhibitor
57658	Ramipril 1.25mg tablets (A A H Pharmaceuticals Ltd)	ACE inhibitor
71025	Ramipril 1.25mg tablets (APC Pharmaceuticals & Chemicals (Europe) Ltd)	ACE inhibitor
56148	Ramipril 1.25mg tablets (Kent Pharmaceuticals Ltd)	ACE inhibitor
57235	Ramipril 1.25mg tablets (Sandoz Ltd)	ACE inhibitor
61985	Ramipril 1.25mg tablets (Teva UK Ltd)	ACE inhibitor
62039	Ramipril 1.25mg tablets (Zentiva)	ACE inhibitor
45340	Ramipril 10mg Capsule (Actavis UK Ltd)	ACE inhibitor
34583	Ramipril 10mg Capsule (Dexcel-Pharma Ltd)	ACE inhibitor
34893	Ramipril 10mg Capsule (IVAX Pharmaceuticals UK Ltd)	ACE inhibitor
34877	Ramipril 10mg Capsule (Sovereign Medical Ltd)	ACE inhibitor
82	Ramipril 10mg capsules	ACE inhibitor

Carr MJ, *et al. BMJ Qual Saf* 2022; 31:503–514. doi: 10.1136/bmjqs-2021-013613

34943	Ramipril 10mg capsules (A A H Pharmaceuticals Ltd)	ACE inhibitor
49164	Ramipril 10mg capsules (Actavis UK Ltd)	ACE inhibitor
56356	Ramipril 10mg capsules (Alliance Healthcare (Distribution) Ltd)	ACE inhibitor
61339	Ramipril 10mg capsules (Almus Pharmaceuticals Ltd)	ACE inhibitor
56169	Ramipril 10mg capsules (Arrow Generics Ltd)	ACE inhibitor
59788	Ramipril 10mg capsules (Bristol Laboratories Ltd)	ACE inhibitor
69288	Ramipril 10mg capsules (Brown & Burk UK Ltd)	ACE inhibitor
66669	Ramipril 10mg capsules (DE Pharmaceuticals)	ACE inhibitor
34357	Ramipril 10mg capsules (Genus Pharmaceuticals Ltd)	ACE inhibitor
52407	Ramipril 10mg capsules (Kent Pharmaceuticals Ltd)	ACE inhibitor
68480	Ramipril 10mg capsules (Mawdsley-Brooks & Company Ltd)	ACE inhibitor
34651	Ramipril 10mg capsules (Mylan)	ACE inhibitor
56763	Ramipril 10mg capsules (Phoenix Healthcare Distribution Ltd)	ACE inhibitor
34710	Ramipril 10mg capsules (Sandoz Ltd)	ACE inhibitor
56855	Ramipril 10mg capsules (Sigma Pharmaceuticals Plc)	ACE inhibitor
33894	Ramipril 10mg capsules (Teva UK Ltd)	ACE inhibitor
57346	Ramipril 10mg capsules (Waymade Healthcare Plc)	ACE inhibitor
72842	Ramipril 10mg capsules (Wockhardt UK Ltd)	ACE inhibitor
34657	Ramipril 10mg capsules (Zentiva)	ACE inhibitor
756	Ramipril 10mg tablets	ACE inhibitor
40384	Ramipril 10mg tablets (A A H Pharmaceuticals Ltd)	ACE inhibitor
70709	Ramipril 10mg tablets (Actavis UK Ltd)	ACE inhibitor
53612	Ramipril 10mg tablets (Alliance Healthcare (Distribution) Ltd)	ACE inhibitor
56038	Ramipril 10mg tablets (Pfizer Ltd)	ACE inhibitor
63010	Ramipril 10mg tablets (Phoenix Healthcare Distribution Ltd)	ACE inhibitor
66162	Ramipril 10mg tablets (Teva UK Ltd)	ACE inhibitor
74066	Ramipril 10mg tablets (Tillomed Laboratories Ltd)	ACE inhibitor
50509	Ramipril 10mg/5ml oral solution	ACE inhibitor
35007	Ramipril 10mg/5ml oral suspension	ACE inhibitor
654	Ramipril 2.5/5mg/10mg capsule	ACE inhibitor
38308	Ramipril 2.5/5mg/10mg tablet	ACE inhibitor
34732	Ramipril 2.5mg Capsule (Dexcel-Pharma Ltd)	ACE inhibitor
709	Ramipril 2.5mg capsules	ACE inhibitor
34528	Ramipril 2.5mg capsules (A A H Pharmaceuticals Ltd)	ACE inhibitor
47998	Ramipril 2.5mg capsules (Actavis UK Ltd)	ACE inhibitor
51714	Ramipril 2.5mg capsules (Alliance Healthcare (Distribution) Ltd)	ACE inhibitor
48053	Ramipril 2.5mg capsules (Almus Pharmaceuticals Ltd)	ACE inhibitor
54298	Ramipril 2.5mg capsules (Arrow Generics Ltd)	ACE inhibitor
53621	Ramipril 2.5mg capsules (Bristol Laboratories Ltd)	ACE inhibitor
71491	Ramipril 2.5mg capsules (Ennogen Pharma Ltd)	ACE inhibitor
34432	Ramipril 2.5mg capsules (Genus Pharmaceuticals Ltd)	ACE inhibitor
59557	Ramipril 2.5mg capsules (Kent Pharmaceuticals Ltd)	ACE inhibitor
74618	Ramipril 2.5mg capsules (Mawdsley-Brooks & Company Ltd)	ACE inhibitor
34567	Ramipril 2.5mg capsules (Mylan)	ACE inhibitor
59603	Ramipril 2.5mg capsules (Phoenix Healthcare Distribution Ltd)	ACE inhibitor
33811	Ramipril 2.5mg capsules (Ranbaxy (UK) Ltd)	ACE inhibitor
34505	Ramipril 2.5mg capsules (Sandoz Ltd)	ACE inhibitor

54620	Ramipril 2.5mg capsules (Sigma Pharmaceuticals Plc)	ACE inhibitor
34490	Ramipril 2.5mg capsules (Teva UK Ltd)	ACE inhibitor
56013	Ramipril 2.5mg capsules (Waymade Healthcare Plc)	ACE inhibitor
72341	Ramipril 2.5mg capsules (Wockhardt UK Ltd)	ACE inhibitor
34431	Ramipril 2.5mg capsules (Zentiva)	ACE inhibitor
6314	Ramipril 2.5mg tablets	ACE inhibitor
71068	Ramipril 2.5mg tablets (APC Pharmaceuticals & Chemicals (Europe) Ltd)	ACE inhibitor
61499	Ramipril 2.5mg tablets (Actavis UK Ltd)	ACE inhibitor
63442	Ramipril 2.5mg tablets (Teva UK Ltd)	ACE inhibitor
62918	Ramipril 2.5mg/5ml oral solution	ACE inhibitor
47021	Ramipril 2.5mg/5ml oral solution sugar free	ACE inhibitor
73459	Ramipril 2.5mg/5ml oral solution sugar free (A A H Pharmaceuticals Ltd)	ACE inhibitor
64055	Ramipril 2.5mg/5ml oral solution sugar free (Waymade Healthcare Plc)	ACE inhibitor
11937	Ramipril 2.5mg/5ml oral suspension	ACE inhibitor
34589	Ramipril 5mg Capsule (Dexcel-Pharma Ltd)	ACE inhibitor
34652	Ramipril 5mg Capsule (Sovereign Medical Ltd)	ACE inhibitor
74632	Ramipril 5mg Tablet (Sovereign Medical Ltd)	ACE inhibitor
80	Ramipril 5mg capsules	ACE inhibitor
34540	Ramipril 5mg capsules (A A H Pharmaceuticals Ltd)	ACE inhibitor
48008	Ramipril 5mg capsules (Actavis UK Ltd)	ACE inhibitor
54941	Ramipril 5mg capsules (Alliance Healthcare (Distribution) Ltd)	ACE inhibitor
61067	Ramipril 5mg capsules (Almus Pharmaceuticals Ltd)	ACE inhibitor
51701	Ramipril 5mg capsules (Bristol Laboratories Ltd)	ACE inhibitor
68192	Ramipril 5mg capsules (Brown & Burk UK Ltd)	ACE inhibitor
65936	Ramipril 5mg capsules (DE Pharmaceuticals)	ACE inhibitor
65749	Ramipril 5mg capsules (Ennogen Pharma Ltd)	ACE inhibitor
34390	Ramipril 5mg capsules (Genus Pharmaceuticals Ltd)	ACE inhibitor
56129	Ramipril 5mg capsules (Kent Pharmaceuticals Ltd)	ACE inhibitor
67719	Ramipril 5mg capsules (Mawdsley-Brooks & Company Ltd)	ACE inhibitor
34429	Ramipril 5mg capsules (Mylan)	ACE inhibitor
60730	Ramipril 5mg capsules (Phoenix Healthcare Distribution Ltd)	ACE inhibitor
34539	Ramipril 5mg capsules (Sandoz Ltd)	ACE inhibitor
52197	Ramipril 5mg capsules (Sigma Pharmaceuticals Plc)	ACE inhibitor
34412	Ramipril 5mg capsules (Teva UK Ltd)	ACE inhibitor
55798	Ramipril 5mg capsules (Waymade Healthcare Plc)	ACE inhibitor
34382	Ramipril 5mg capsules (Zentiva)	ACE inhibitor
6288	Ramipril 5mg tablets	ACE inhibitor
65599	Ramipril 5mg tablets (A A H Pharmaceuticals Ltd)	ACE inhibitor
68372	Ramipril 5mg tablets (Actavis UK Ltd)	ACE inhibitor
71040	Ramipril 5mg tablets (Pfizer Ltd)	ACE inhibitor
57864	Ramipril 5mg tablets (Sigma Pharmaceuticals Plc)	ACE inhibitor
62958	Ramipril 5mg tablets (Teva UK Ltd)	ACE inhibitor
62036	Ramipril 5mg tablets (Waymade Healthcare Plc)	ACE inhibitor
61694	Ramipril 5mg tablets (Zentiva)	ACE inhibitor
45554	Ramipril 5mg/5ml oral solution	ACE inhibitor
46890	Ramipril 5mg/5ml oral suspension	ACE inhibitor
66329	Ramipril oral solution	ACE inhibitor

4571	Staril 10mg tablets (Bristol-Myers Squibb Pharmaceuticals Ltd)	ACE inhibitor
13589	Staril 20mg tablets (Bristol-Myers Squibb Pharmaceuticals Ltd)	ACE inhibitor
67307	Staril 20mg tablets (Dowelhurst Ltd)	ACE inhibitor
31288	TRITACE	ACE inhibitor
24214	TRITACE 1.25 MG TAB	ACE inhibitor
29964	TRITACE 2.5 MG TAB	ACE inhibitor
12815	Tanatril 10mg tablets (Mitsubishi Tanabe Pharma Europe Ltd)	ACE inhibitor
32560	Tanatril 20mg tablets (Mitsubishi Tanabe Pharma Europe Ltd)	ACE inhibitor
6408	Tanatril 5mg tablets (Mitsubishi Tanabe Pharma Europe Ltd)	ACE inhibitor
20849	Tensopril 12.5mg tablets (Teva UK Ltd)	ACE inhibitor
30039	Tensopril 25mg tablets (Teva UK Ltd)	ACE inhibitor
23478	Tensopril 50mg tablets (Teva UK Ltd)	ACE inhibitor
4103	Trandolapril 1mg capsules	ACE inhibitor
74209	Trandolapril 1mg capsules (Actavis UK Ltd)	ACE inhibitor
5047	Trandolapril 2mg capsules	ACE inhibitor
66623	Trandolapril 2mg capsules (A A H Pharmaceuticals Ltd)	ACE inhibitor
9948	Trandolapril 4mg capsules	ACE inhibitor
54345	Trandolapril 4mg capsules (Arrow Generics Ltd)	ACE inhibitor
65570	Trandolapril 4mg capsules (Teva UK Ltd)	ACE inhibitor
7419	Trandolapril 500microgram capsules	ACE inhibitor
60757	Trandolapril 500microgram capsules (Teva UK Ltd)	ACE inhibitor
42081	Tritace 1.25mg Tablet (Sterwin Medicines)	ACE inhibitor
9646	Tritace 1.25mg capsules (Aventis Pharma)	ACE inhibitor
6261	Tritace 1.25mg tablets (Sanofi)	ACE inhibitor
39355	Tritace 10mg Tablet (Sterwin Medicines)	ACE inhibitor
9693	Tritace 10mg capsules (Sanofi)	ACE inhibitor
9915	Tritace 10mg tablets (Sanofi)	ACE inhibitor
5275	Tritace 2.5mg capsules (Sanofi)	ACE inhibitor
6364	Tritace 2.5mg tablets (Sanofi)	ACE inhibitor
73484	Tritace 5mg Tablet (Sterwin Medicines)	ACE inhibitor
5735	Tritace 5mg capsules (Sanofi)	ACE inhibitor
6362	Tritace 5mg tablets (Sanofi)	ACE inhibitor
6200	Tritace titration pack capsules (Sanofi)	ACE inhibitor
23642	Vascace 0.25mg Tablet (Roche Products Ltd)	ACE inhibitor
16212	Vascace 1mg tablets (Roche Products Ltd)	ACE inhibitor
16197	Vascace 2.5mg tablets (Roche Products Ltd)	ACE inhibitor
21053	Vascace 500microgram tablets (Roche Products Ltd)	ACE inhibitor
16196	Vascace 5mg tablets (Roche Products Ltd)	ACE inhibitor
6806	Zestril 10mg tablets (AstraZeneca UK Ltd)	ACE inhibitor
3720	Zestril 2.5mg tablets (AstraZeneca UK Ltd)	ACE inhibitor
57588	Zestril 2.5mg tablets (Mawdsley-Brooks & Company Ltd)	ACE inhibitor
8268	Zestril 20mg tablets (AstraZeneca UK Ltd)	ACE inhibitor
56510	Zestril 20mg tablets (Sigma Pharmaceuticals Plc)	ACE inhibitor
6807	Zestril 5mg tablets (AstraZeneca UK Ltd)	ACE inhibitor
56505	Zestril 5mg tablets (Lexon (UK) Ltd)	ACE inhibitor
5485	Alfuzosin 10mg modified-release tablets	Alpha blocker
71090	Alfuzosin 10mg modified-release tablets (Mawdsley-Brooks & Company Ltd)	Alpha blocker

62539	Alfuzosin 10mg modified-release tablets (Phoenix Healthcare Distribution Ltd)	Alpha blocker
69082	Alfuzosin 10mg modified-release tablets (Sigma Pharmaceuticals Plc)	Alpha blocker
2088	Alfuzosin 2.5mg tablets	Alpha blocker
71036	Alfuzosin 2.5mg tablets (Sandoz Ltd)	Alpha blocker
64443	Alfuzosin 2.5mg tablets (Sigma Pharmaceuticals Plc)	Alpha blocker
56793	Alfuzosin 2.5mg tablets (Teva UK Ltd)	Alpha blocker
2120	Alfuzosin 5mg modified-release tablets	Alpha blocker
42820	Alfuzosin xl 10mg Tablet (Hillcross Pharmaceuticals Ltd)	Alpha blocker
35466	Alphacard MR 400microgram capsules (Ratiopharm UK Ltd)	Alpha blocker
26238	Alphavase 1 tablets (Ashbourne Pharmaceuticals Ltd)	Alpha blocker
13610	Alphavase 2 tablets (Ashbourne Pharmaceuticals Ltd)	Alpha blocker
19823	Alphavase 5 tablets (Ashbourne Pharmaceuticals Ltd)	Alpha blocker
26237	Alphavase 500microgram Tablet (Ashbourne Pharmaceuticals Ltd)	Alpha blocker
11394	Baratol 25mg Tablet (Shire Pharmaceuticals Ltd)	Alpha blocker
40256	Baratol 25mg tablets (Amdipharm Plc)	Alpha blocker
16198	Baratol 50mg Tablet (Shire Pharmaceuticals Ltd)	Alpha blocker
35312	Bazetham MR 400microgram capsules (Teva UK Ltd)	Alpha blocker
47563	Besavar XL 10mg tablets (Actavis UK Ltd)	Alpha blocker
35639	Besavar XL 10mg tablets (Zentiva)	Alpha blocker
46066	Cardozin XL 4mg tablets (Almus Pharmaceuticals Ltd)	Alpha blocker
38461	Cardozin XL 4mg tablets (Arrow Generics Ltd)	Alpha blocker
36023	Cardozin xl 4mg Tablet (Hillcross Pharmaceuticals Ltd)	Alpha blocker
37243	Cardozin xl 4mg Tablet (Teva UK Ltd)	Alpha blocker
4449	Cardura 1mg tablets (Pfizer Ltd)	Alpha blocker
4802	Cardura 2mg tablets (Pfizer Ltd)	Alpha blocker
8086	Cardura 4mg Tablet (Pfizer Ltd)	Alpha blocker
71405	Cardura XL 4mg tablets (DE Pharmaceuticals)	Alpha blocker
755	Cardura XL 4mg tablets (Pfizer Ltd)	Alpha blocker
74000	Cardura XL 4mg tablets (Waymade Healthcare Plc)	Alpha blocker
5618	Cardura XL 8mg tablets (Pfizer Ltd)	Alpha blocker
25487	Cascor 2mg tablets (Ranbaxy (UK) Ltd)	Alpha blocker
25551	Cascor 4mg tablets (Ranbaxy (UK) Ltd)	Alpha blocker
43695	Colixil XL 4mg tablets (Sandoz Ltd)	Alpha blocker
35925	Contiflo XL 400microgram capsules (Ranbaxy (UK) Ltd)	Alpha blocker
53084	Cositam XL 400microgram tablets (Consilient Health Ltd)	Alpha blocker
21049	DIBENYLINE 2.5 MG CAP	Alpha blocker
29765	DIBENYLINE 5 MG TAB	Alpha blocker
8942	Dibenyline 10mg capsules (Mercury Pharma Group Ltd)	Alpha blocker
35058	Diffundox XL 400microgram capsules (Zentiva)	Alpha blocker
2117	Doralese Tiltab 20mg tablets (Chemidex Pharma Ltd)	Alpha blocker
7549	Doxadura 1mg tablets (Discovery Pharmaceuticals)	Alpha blocker
7547	Doxadura 2mg tablets (Discovery Pharmaceuticals)	Alpha blocker
10088	Doxadura 4mg tablets (Discovery Pharmaceuticals)	Alpha blocker
35272	Doxadura XL 4mg tablets (Discovery Pharmaceuticals)	Alpha blocker
119	Doxazosin 1mg tablets	Alpha blocker
34715	Doxazosin 1mg tablets (A A H Pharmaceuticals Ltd)	Alpha blocker

48150	Doxazosin 1mg tablets (Actavis UK Ltd)	Alpha blocker
55916	Doxazosin 1mg tablets (Alliance Healthcare (Distribution) Ltd)	Alpha blocker
62019	Doxazosin 1mg tablets (Almus Pharmaceuticals Ltd)	Alpha blocker
60319	Doxazosin 1mg tablets (Bristol Laboratories Ltd)	Alpha blocker
55906	Doxazosin 1mg tablets (Dexcel-Pharma Ltd)	Alpha blocker
41543	Doxazosin 1mg tablets (IVAX Pharmaceuticals UK Ltd)	Alpha blocker
59209	Doxazosin 1mg tablets (Kent Pharmaceuticals Ltd)	Alpha blocker
65853	Doxazosin 1mg tablets (Mawdsley-Brooks & Company Ltd)	Alpha blocker
34601	Doxazosin 1mg tablets (Mylan)	Alpha blocker
45328	Doxazosin 1mg tablets (Sandoz Ltd)	Alpha blocker
63314	Doxazosin 1mg tablets (Sovereign Medical Ltd)	Alpha blocker
73637	Doxazosin 1mg tablets (Sterwin Medicines)	Alpha blocker
34342	Doxazosin 1mg tablets (Teva UK Ltd)	Alpha blocker
64233	Doxazosin 1mg tablets (Waymade Healthcare Plc)	Alpha blocker
72348	Doxazosin 1mg/5ml oral solution	Alpha blocker
20369	Doxazosin 1mg/5ml oral suspension	Alpha blocker
493	Doxazosin 2mg tablets	Alpha blocker
34625	Doxazosin 2mg tablets (A A H Pharmaceuticals Ltd)	Alpha blocker
56145	Doxazosin 2mg tablets (Actavis UK Ltd)	Alpha blocker
50467	Doxazosin 2mg tablets (Alliance Healthcare (Distribution) Ltd)	Alpha blocker
63158	Doxazosin 2mg tablets (Almus Pharmaceuticals Ltd)	Alpha blocker
61066	Doxazosin 2mg tablets (Bristol Laboratories Ltd)	Alpha blocker
69319	Doxazosin 2mg tablets (DE Pharmaceuticals)	Alpha blocker
45583	Doxazosin 2mg tablets (Dexcel-Pharma Ltd)	Alpha blocker
40891	Doxazosin 2mg tablets (IVAX Pharmaceuticals UK Ltd)	Alpha blocker
66065	Doxazosin 2mg tablets (Kent Pharmaceuticals Ltd)	Alpha blocker
58276	Doxazosin 2mg tablets (Medreich Plc)	Alpha blocker
33094	Doxazosin 2mg tablets (Mylan)	Alpha blocker
62351	Doxazosin 2mg tablets (Phoenix Healthcare Distribution Ltd)	Alpha blocker
57074	Doxazosin 2mg tablets (Sigma Pharmaceuticals Plc)	Alpha blocker
73116	Doxazosin 2mg tablets (Sovereign Medical Ltd)	Alpha blocker
19193	Doxazosin 2mg tablets (Teva UK Ltd)	Alpha blocker
72346	Doxazosin 2mg/5ml oral solution	Alpha blocker
57784	Doxazosin 2mg/5ml oral suspension	Alpha blocker
582	Doxazosin 4mg modified-release tablets	Alpha blocker
1294	Doxazosin 4mg tablets	Alpha blocker
57448	Doxazosin 4mg tablets (A A H Pharmaceuticals Ltd)	Alpha blocker
51685	Doxazosin 4mg tablets (Actavis UK Ltd)	Alpha blocker
61283	Doxazosin 4mg tablets (Alliance Healthcare (Distribution) Ltd)	Alpha blocker
62158	Doxazosin 4mg tablets (Almus Pharmaceuticals Ltd)	Alpha blocker
53322	Doxazosin 4mg tablets (Bristol Laboratories Ltd)	Alpha blocker
60200	Doxazosin 4mg tablets (DE Pharmaceuticals)	Alpha blocker
59862	Doxazosin 4mg tablets (Dexcel-Pharma Ltd)	Alpha blocker
19216	Doxazosin 4mg tablets (IVAX Pharmaceuticals UK Ltd)	Alpha blocker
68161	Doxazosin 4mg tablets (Mawdsley-Brooks & Company Ltd)	Alpha blocker
54785	Doxazosin 4mg tablets (Medreich Plc)	Alpha blocker
34553	Doxazosin 4mg tablets (Mylan)	Alpha blocker

58325	Doxazosin 4mg tablets (Phoenix Healthcare Distribution Ltd)	Alpha blocker
45342	Doxazosin 4mg tablets (Sandoz Ltd)	Alpha blocker
74831	Doxazosin 4mg tablets (Sigma Pharmaceuticals Plc)	Alpha blocker
68022	Doxazosin 4mg tablets (Sovereign Medical Ltd)	Alpha blocker
40678	Doxazosin 4mg tablets (Teva UK Ltd)	Alpha blocker
65159	Doxazosin 4mg tablets (Waymade Healthcare Plc)	Alpha blocker
61123	Doxazosin 4mg/5ml oral solution	Alpha blocker
35603	Doxazosin 4mg/5ml oral suspension	Alpha blocker
5496	Doxazosin 8mg modified-release tablets	Alpha blocker
74567	Doxazosin 8mg tablets	Alpha blocker
69757	Doxazosin 8mg/5ml oral suspension	Alpha blocker
45265	Doxazosin sr 4mg Tablet (Generics (UK) Ltd)	Alpha blocker
47807	Doxazosin xl 4mg Tablet (Hillcross Pharmaceuticals Ltd)	Alpha blocker
53033	Doxzogen XL 4mg tablets (Mylan)	Alpha blocker
58517	Faramsil 400microgram modified-release tablets (Sandoz Ltd)	Alpha blocker
70733	Flectone XL 400microgram tablets (Teva UK Ltd)	Alpha blocker
460	Flomax MR 400microgram capsules (Astellas Pharma Ltd)	Alpha blocker
42936	Flomax Relief MR 400microgram capsules (Sanofi)	Alpha blocker
10134	Flomaxtra XL 400microgram tablets (Astellas Pharma Ltd)	Alpha blocker
39373	Fuzatal XL 10mg tablets (Teva UK Ltd)	Alpha blocker
54497	Galebon 400microgram modified-release capsules (Consilient Health Ltd)	Alpha blocker
19566	HYPOVASE	Alpha blocker
24211	HYPOVASE B.D. STARTER PACK	Alpha blocker
1292	Hypovase 1mg tablets (Pfizer Ltd)	Alpha blocker
5183	Hypovase 2mg tablets (Pfizer Ltd)	Alpha blocker
4111	Hypovase 500microgram tablets (Pfizer Ltd)	Alpha blocker
8198	Hypovase 5mg Tablet (Pfizer Ltd)	Alpha blocker
8863	Hypovase benign prostatic hyperplasia 1mg Tablet (Pfizer Ltd)	Alpha blocker
23459	Hypovase benign prostatic hyperplasia 2mg Tablet (Pfizer Ltd)	Alpha blocker
25047	Hypovase benign prostatic hyperplasia 500microgram Tablet (Pfizer Ltd)	Alpha blocker
26693	Hypovase benign prostatic hyperplasia bd BD Starter pack (Pfizer Ltd)	Alpha blocker
12518	Hypovase tablets B.D. starter pack (Pfizer Ltd)	Alpha blocker
2347	Hytrin 10mg Tablet (Abbott Laboratories Ltd)	Alpha blocker
37428	Hytrin 10mg tablets (AMCo)	Alpha blocker
8077	Hytrin 2mg Tablet (Abbott Laboratories Ltd)	Alpha blocker
36649	Hytrin 2mg tablets (AMCo)	Alpha blocker
71419	Hytrin 2mg tablets (Waymade Healthcare Plc)	Alpha blocker
2346	Hytrin 5mg Tablet (Abbott Laboratories Ltd)	Alpha blocker
36780	Hytrin 5mg tablets (AMCo)	Alpha blocker
74037	Hytrin 5mg tablets (Waymade Healthcare Plc)	Alpha blocker
2348	Hytrin bph 10mg Tablet (Amdipharm Plc)	Alpha blocker
16201	Hytrin bph 2mg Tablet (Amdipharm Plc)	Alpha blocker
5337	Hytrin bph 5mg Tablet (Amdipharm Plc)	Alpha blocker
2816	Indoramin 20mg tablets	Alpha blocker
5815	Indoramin 25mg tablets	Alpha blocker
9019	Indoramin 50mg Tablet	Alpha blocker
55005	Kelanu XL 10mg tablets (Pfizer Ltd)	Alpha blocker

42462	Kirtacap mr 400microgram Capsule (Consilient Health Ltd)	Alpha blocker
45040	Larbex XL 4mg tablets (Teva UK Ltd)	Alpha blocker
36282	Morvesin XL 400microgram capsules (Sandoz Ltd)	Alpha blocker
6008	Omnic 400microgram Modified-release capsule (Paines & Byrne Ltd)	Alpha blocker
27403	Omnic MR 400microgram capsules (Astellas Pharma Ltd)	Alpha blocker
25004	PHENOXYBENZAMINE HCl 2.5 MG CAP	Alpha blocker
21466	PHENOXYBENZAMINE HCl 5 MG CAP	Alpha blocker
26783	PHENOXYBENZAMINE HCl 5 MG TAB	Alpha blocker
28441	Pamsvax XL 400microgram capsules (Actavis UK Ltd)	Alpha blocker
46206	Pamsvax XL 400microgram capsules (Almus Pharmaceuticals Ltd)	Alpha blocker
24369	Petyme 400microgram MR capsules (Teva UK Ltd)	Alpha blocker
45584	Phenoxybenzamine 100mg/2ml solution for infusion ampoules	Alpha blocker
7759	Phenoxybenzamine 10mg capsules	Alpha blocker
62300	Phenoxybenzamine 10mg capsules (AMCo)	Alpha blocker
40810	Phenoxybenzamine 10mg/5ml oral suspension	Alpha blocker
54918	Phenoxybenzamine oral liquid	Alpha blocker
12545	Phentolamine 10mg/1ml solution for injection ampoules	Alpha blocker
44553	Pinexel PR 400microgram capsules (Wockhardt UK Ltd)	Alpha blocker
60316	Prazosin 1mg Tablet (Approved Prescription Services Ltd)	Alpha blocker
591	Prazosin 1mg tablets	Alpha blocker
41721	Prazosin 1mg tablets (A A H Pharmaceuticals Ltd)	Alpha blocker
46922	Prazosin 1mg tablets (IVAX Pharmaceuticals UK Ltd)	Alpha blocker
445	Prazosin 1mg tablets and Prazosin 500microgram tablets	Alpha blocker
726	Prazosin 2mg tablets	Alpha blocker
41651	Prazosin 500microgram Tablet (Approved Prescription Services Ltd)	Alpha blocker
1455	Prazosin 500microgram tablets	Alpha blocker
41652	Prazosin 500microgram tablets (A A H Pharmaceuticals Ltd)	Alpha blocker
43547	Prazosin 500microgram tablets (IVAX Pharmaceuticals UK Ltd)	Alpha blocker
3715	Prazosin 5mg tablets	Alpha blocker
55826	Prazosin 5mg tablets (A A H Pharmaceuticals Ltd)	Alpha blocker
31109	Prosurin XL 400microgram capsules (Mylan)	Alpha blocker
46526	Raporsin XL 4mg tablets (Actavis UK Ltd)	Alpha blocker
23010	Rogitine 10mg/1ml solution for injection ampoules (Alliance Pharmaceuticals Ltd)	Alpha blocker
36740	Slocinx XL 4mg tablets (Zentiva)	Alpha blocker
34080	Stronazon 400microgram MR capsules (Actavis UK Ltd)	Alpha blocker
14932	Tabphyn MR 400microgram capsules (Kyowa Kirin Ltd)	Alpha blocker
68584	Tabphyn MR 400microgram capsules (Thornton & Ross Ltd)	Alpha blocker
634	Tamsulosin 400microgram modified-release capsules	Alpha blocker
53964	Tamsulosin 400microgram modified-release capsules (A A H Pharmaceuticals Ltd)	Alpha blocker
62553	Tamsulosin 400microgram modified-release capsules (Alliance Healthcare (Distribution) Ltd)	Alpha blocker
52159	Tamsulosin 400microgram modified-release capsules (Focus Pharmaceuticals Ltd)	Alpha blocker
73300	Tamsulosin 400microgram modified-release capsules (Sigma Pharmaceuticals Plc)	Alpha blocker
74539	Tamsulosin 400microgram modified-release capsules (Waymade Healthcare	Alpha blocker

	Plc)	
7056	Tamsulosin 400microgram modified-release tablets	Alpha blocker
71955	Tamsulosin 400microgram modified-release tablets (A A H Pharmaceuticals Ltd)	Alpha blocker
52055	Tamsulosin 400microgram oral powder sachets	Alpha blocker
65124	Tamsulosin 400micrograms/5ml oral solution	Alpha blocker
69622	Tamsulosin 400micrograms/5ml oral suspension	Alpha blocker
51665	Tamurex 400microgram modified-release capsules (Somex Pharma)	Alpha blocker
4875	Terazosin 10mg tablets	Alpha blocker
3470	Terazosin 1mg tablets	Alpha blocker
4637	Terazosin 2mg tablets	Alpha blocker
57145	Terazosin 2mg tablets (A A H Pharmaceuticals Ltd)	Alpha blocker
4694	Terazosin 2mg tablets and Terazosin 1mg tablets	Alpha blocker
3924	Terazosin 5mg tablets	Alpha blocker
72644	Terazosin 5mg tablets (A A H Pharmaceuticals Ltd)	Alpha blocker
65442	Terazosin 5mg tablets (Mylan)	Alpha blocker
44268	Vasran XL 10mg tablets (Ranbaxy (UK) Ltd)	Alpha blocker
57549	Xatral 2.5mg tablets (Necessity Supplies Ltd)	Alpha blocker
5624	Xatral 2.5mg tablets (Sanofi)	Alpha blocker
58985	Xatral SR 5mg tablets (Sanofi-Synthelabo Ltd)	Alpha blocker
5179	Xatral XL 10mg tablets (Sanofi)	Alpha blocker
9422	Xatral sr 5mg Tablet (Sanofi-Synthelabo Ltd)	Alpha blocker
74334	Zochek 10mg modified-release tablets (Aurobindo Pharma Ltd)	Alpha blocker
36439	Zufal XL 10mg tablets (Teva UK Ltd)	Alpha blocker
5117	Amias 16mg tablets (Takeda UK Ltd)	Angiotensin receptor blocker
4155	Amias 2mg tablets (Takeda UK Ltd)	Angiotensin receptor blocker
31072	Amias 32mg tablets (Takeda UK Ltd)	Angiotensin receptor blocker
4685	Amias 4mg tablets (Takeda UK Ltd)	Angiotensin receptor blocker
5013	Amias 8mg tablets (Takeda UK Ltd)	Angiotensin receptor blocker
9196	Aprovel 150mg tablets (Sanofi)	Angiotensin receptor blocker
11348	Aprovel 300mg tablets (Sanofi)	Angiotensin receptor blocker
7338	Aprovel 75mg tablets (Sanofi)	Angiotensin receptor blocker
56606	Azilsartan medoxomil 40mg tablets	Angiotensin receptor blocker
51368	Azilsartan medoxomil 80mg tablets	Angiotensin receptor blocker
4741	Candesartan 16mg tablets	Angiotensin receptor blocker
52208	Candesartan 16mg tablets (A A H Pharmaceuticals Ltd)	Angiotensin receptor blocker
57977	Candesartan 16mg tablets (Alliance Healthcare (Distribution) Ltd)	Angiotensin receptor blocker

54414	Candesartan 16mg tablets (Consilient Health Ltd)	Angiotensin receptor blocker
68751	Candesartan 16mg tablets (Genesis Pharmaceuticals Ltd)	Angiotensin receptor blocker
65228	Candesartan 16mg tablets (Mawdsley-Brooks & Company Ltd)	Angiotensin receptor blocker
66624	Candesartan 16mg tablets (Sandoz Ltd)	Angiotensin receptor blocker
53680	Candesartan 16mg tablets (Teva UK Ltd)	Angiotensin receptor blocker
67929	Candesartan 16mg tablets (Tillomed Laboratories Ltd)	Angiotensin receptor blocker
62035	Candesartan 16mg tablets (Waymade Healthcare Plc)	Angiotensin receptor blocker
529	Candesartan 2mg tablets	Angiotensin receptor blocker
65479	Candesartan 2mg tablets (A A H Pharmaceuticals Ltd)	Angiotensin receptor blocker
57266	Candesartan 2mg tablets (Actavis UK Ltd)	Angiotensin receptor blocker
73811	Candesartan 2mg tablets (Ranbaxy (UK) Ltd)	Angiotensin receptor blocker
59802	Candesartan 2mg tablets (Teva UK Ltd)	Angiotensin receptor blocker
7043	Candesartan 32mg tablets	Angiotensin receptor blocker
54326	Candesartan 32mg tablets (Teva UK Ltd)	Angiotensin receptor blocker
531	Candesartan 4mg tablets	Angiotensin receptor blocker
68718	Candesartan 4mg tablets (A A H Pharmaceuticals Ltd)	Angiotensin receptor blocker
58646	Candesartan 4mg tablets (Actavis UK Ltd)	Angiotensin receptor blocker
72215	Candesartan 4mg tablets (Consilient Health Ltd)	Angiotensin receptor blocker
64359	Candesartan 4mg tablets (DE Pharmaceuticals)	Angiotensin receptor blocker
51647	Candesartan 4mg tablets (Mawdsley-Brooks & Company Ltd)	Angiotensin receptor blocker
73528	Candesartan 4mg tablets (Milpharm Ltd)	Angiotensin receptor blocker
69802	Candesartan 4mg tablets (Mylan)	Angiotensin receptor blocker
72924	Candesartan 4mg tablets (Phoenix Healthcare Distribution Ltd)	Angiotensin receptor blocker
62140	Candesartan 4mg tablets (Sandoz Ltd)	Angiotensin receptor blocker
53755	Candesartan 4mg tablets (Teva UK Ltd)	Angiotensin receptor blocker
73813	Candesartan 4mg tablets (Waymade Healthcare Plc)	Angiotensin receptor blocker

Carr MJ, *et al. BMJ Qual Saf* 2022; 31:503–514. doi: 10.1136/bmjqs-2021-013613

71080	Candesartan 4mg tablets (Zentiva)	Angiotensin receptor blocker
70455	Candesartan 4mg/5ml oral suspension	Angiotensin receptor blocker
4818	Candesartan 8mg tablets	Angiotensin receptor blocker
51519	Candesartan 8mg tablets (A A H Pharmaceuticals Ltd)	Angiotensin receptor blocker
57273	Candesartan 8mg tablets (Actavis UK Ltd)	Angiotensin receptor blocker
59690	Candesartan 8mg tablets (Consilient Health Ltd)	Angiotensin receptor blocker
70805	Candesartan 8mg tablets (Crescent Pharma Ltd)	Angiotensin receptor blocker
51117	Candesartan 8mg tablets (DE Pharmaceuticals)	Angiotensin receptor blocker
68647	Candesartan 8mg tablets (Genesis Pharmaceuticals Ltd)	Angiotensin receptor blocker
66958	Candesartan 8mg tablets (Sandoz Ltd)	Angiotensin receptor blocker
50185	Candesartan 8mg tablets (Teva UK Ltd)	Angiotensin receptor blocker
57026	Candesartan 8mg tablets (Waymade Healthcare Plc)	Angiotensin receptor blocker
52559	Candesartan 8mg tablets (Zentiva)	Angiotensin receptor blocker
14965	Cozaar 100mg tablets (Merck Sharp & Dohme Ltd)	Angiotensin receptor blocker
52427	Cozaar 100mg tablets (Necessity Supplies Ltd)	Angiotensin receptor blocker
40571	Cozaar 12.5mg tablets (Merck Sharp & Dohme Ltd)	Angiotensin receptor blocker
41232	Cozaar 2.5mg/ml oral suspension (Merck Sharp & Dohme Ltd)	Angiotensin receptor blocker
4226	Cozaar 25mg tablets (Merck Sharp & Dohme Ltd)	Angiotensin receptor blocker
5723	Cozaar 50mg tablets (Merck Sharp & Dohme Ltd)	Angiotensin receptor blocker
45600	Diovan 160mg Tablet (Novartis Pharmaceuticals UK Ltd)	Angiotensin receptor blocker
74013	Diovan 160mg capsules (DE Pharmaceuticals)	Angiotensin receptor blocker
6518	Diovan 160mg capsules (Novartis Pharmaceuticals UK Ltd)	Angiotensin receptor blocker
39199	Diovan 320mg tablets (Novartis Pharmaceuticals UK Ltd)	Angiotensin receptor blocker
70628	Diovan 3mg/1ml oral solution (Novartis Pharmaceuticals UK Ltd)	Angiotensin receptor blocker
11251	Diovan 40mg capsules (Novartis Pharmaceuticals UK Ltd)	Angiotensin receptor blocker
24359	Diovan 40mg tablets (Novartis Pharmaceuticals UK Ltd)	Angiotensin receptor blocker

11252	Diovan 80mg capsules (Novartis Pharmaceuticals UK Ltd)	Angiotensin receptor blocker
51897	Edarbi 20mg tablets (Takeda UK Ltd)	Angiotensin receptor blocker
66261	Entresto 24mg/26mg tablets (Novartis Pharmaceuticals UK Ltd)	Angiotensin receptor blocker
66205	Entresto 49mg/51mg tablets (Novartis Pharmaceuticals UK Ltd)	Angiotensin receptor blocker
66829	Entresto 97mg/103mg tablets (Novartis Pharmaceuticals UK Ltd)	Angiotensin receptor blocker
6939	Eprosartan 300mg tablets	Angiotensin receptor blocker
13123	Eprosartan 400mg tablets	Angiotensin receptor blocker
12836	Eprosartan 600mg tablets	Angiotensin receptor blocker
63337	Eprosartan 600mg tablets (A A H Pharmaceuticals Ltd)	Angiotensin receptor blocker
31160	IRBESARTAN	Angiotensin receptor blocker
1293	Irbesartan 150mg tablets	Angiotensin receptor blocker
58108	Irbesartan 150mg tablets (A A H Pharmaceuticals Ltd)	Angiotensin receptor blocker
58201	Irbesartan 150mg tablets (Actavis UK Ltd)	Angiotensin receptor blocker
72000	Irbesartan 150mg tablets (Alliance Healthcare (Distribution) Ltd)	Angiotensin receptor blocker
71096	Irbesartan 150mg tablets (Dr Reddy's Laboratories (UK) Ltd)	Angiotensin receptor blocker
70431	Irbesartan 150mg tablets (Lupin Healthcare (UK) Ltd)	Angiotensin receptor blocker
60597	Irbesartan 150mg tablets (Teva UK Ltd)	Angiotensin receptor blocker
2971	Irbesartan 300mg tablets	Angiotensin receptor blocker
62415	Irbesartan 300mg tablets (A A H Pharmaceuticals Ltd)	Angiotensin receptor blocker
55017	Irbesartan 300mg tablets (Accord Healthcare Ltd)	Angiotensin receptor blocker
63411	Irbesartan 300mg tablets (Alliance Healthcare (Distribution) Ltd)	Angiotensin receptor blocker
63717	Irbesartan 300mg tablets (DE Pharmaceuticals)	Angiotensin receptor blocker
59393	Irbesartan 300mg tablets (Sandoz Ltd)	Angiotensin receptor blocker
52972	Irbesartan 300mg tablets (Sigma Pharmaceuticals Plc)	Angiotensin receptor blocker
61781	Irbesartan 300mg tablets (Teva UK Ltd)	Angiotensin receptor blocker
70955	Irbesartan 300mg/5ml Oral suspension (Martindale Pharmaceuticals Ltd)	Angiotensin receptor blocker

36939	Irbesartan 300mg/5ml oral suspension	Angiotensin receptor blocker
828	Irbesartan 75mg tablets	Angiotensin receptor blocker
65065	Irbesartan 75mg tablets (A A H Pharmaceuticals Ltd)	Angiotensin receptor blocker
71215	Irbesartan 75mg tablets (Actavis UK Ltd)	Angiotensin receptor blocker
71019	Irbesartan 75mg tablets (Dr Reddy's Laboratories (UK) Ltd)	Angiotensin receptor blocker
624	Losartan 100mg tablets	Angiotensin receptor blocker
49588	Losartan 100mg tablets (A A H Pharmaceuticals Ltd)	Angiotensin receptor blocker
61288	Losartan 100mg tablets (Accord Healthcare Ltd)	Angiotensin receptor blocker
54404	Losartan 100mg tablets (Actavis UK Ltd)	Angiotensin receptor blocker
61053	Losartan 100mg tablets (Alliance Healthcare (Distribution) Ltd)	Angiotensin receptor blocker
70765	Losartan 100mg tablets (Almus Pharmaceuticals Ltd)	Angiotensin receptor blocker
55446	Losartan 100mg tablets (Bristol Laboratories Ltd)	Angiotensin receptor blocker
60506	Losartan 100mg tablets (Dexcel-Pharma Ltd)	Angiotensin receptor blocker
57028	Losartan 100mg tablets (Mylan)	Angiotensin receptor blocker
56970	Losartan 100mg tablets (Pfizer Ltd)	Angiotensin receptor blocker
67902	Losartan 100mg tablets (Sandoz Ltd)	Angiotensin receptor blocker
47006	Losartan 100mg tablets (Teva UK Ltd)	Angiotensin receptor blocker
69667	Losartan 100mg/5ml oral solution	Angiotensin receptor blocker
52658	Losartan 100mg/5ml oral suspension	Angiotensin receptor blocker
39944	Losartan 12.5mg tablets	Angiotensin receptor blocker
52886	Losartan 12.5mg tablets (A A H Pharmaceuticals Ltd)	Angiotensin receptor blocker
58967	Losartan 12.5mg tablets (Alliance Healthcare (Distribution) Ltd)	Angiotensin receptor blocker
68340	Losartan 12.5mg tablets (Consilient Health Ltd)	Angiotensin receptor blocker
62388	Losartan 12.5mg tablets (DE Pharmaceuticals)	Angiotensin receptor blocker
59340	Losartan 12.5mg tablets (Dexcel-Pharma Ltd)	Angiotensin receptor blocker
66114	Losartan 12.5mg tablets (Mawdsley-Brooks & Company Ltd)	Angiotensin receptor blocker

Carr MJ, *et al. BMJ Qual Saf* 2022; 31:503–514. doi: 10.1136/bmjqs-2021-013613

64888	Losartan 12.5mg tablets (Sigma Pharmaceuticals Plc)	Angiotensin receptor blocker
40711	Losartan 2.5mg/ml oral suspension sugar free	Angiotensin receptor blocker
520	Losartan 25mg tablets	Angiotensin receptor blocker
50971	Losartan 25mg tablets (A A H Pharmaceuticals Ltd)	Angiotensin receptor blocker
58274	Losartan 25mg tablets (Accord Healthcare Ltd)	Angiotensin receptor blocker
54740	Losartan 25mg tablets (Actavis UK Ltd)	Angiotensin receptor blocker
69858	Losartan 25mg tablets (Alliance Healthcare (Distribution) Ltd)	Angiotensin receptor blocker
61495	Losartan 25mg tablets (Aptil Pharma Ltd)	Angiotensin receptor blocker
51186	Losartan 25mg tablets (Arrow Generics Ltd)	Angiotensin receptor blocker
58649	Losartan 25mg tablets (Bristol Laboratories Ltd)	Angiotensin receptor blocker
74904	Losartan 25mg tablets (DE Pharmaceuticals)	Angiotensin receptor blocker
48398	Losartan 25mg tablets (Dexcel-Pharma Ltd)	Angiotensin receptor blocker
74589	Losartan 25mg tablets (Genesis Pharmaceuticals Ltd)	Angiotensin receptor blocker
49492	Losartan 25mg tablets (Mylan)	Angiotensin receptor blocker
63222	Losartan 25mg tablets (Pfizer Ltd)	Angiotensin receptor blocker
55718	Losartan 25mg tablets (Phoenix Healthcare Distribution Ltd)	Angiotensin receptor blocker
59271	Losartan 25mg tablets (Sandoz Ltd)	Angiotensin receptor blocker
63918	Losartan 25mg tablets (Teva UK Ltd)	Angiotensin receptor blocker
59086	Losartan 25mg tablets (Wockhardt UK Ltd)	Angiotensin receptor blocker
61754	Losartan 25mg/5ml oral suspension	Angiotensin receptor blocker
1780	Losartan 50mg tablets	Angiotensin receptor blocker
56104	Losartan 50mg tablets (A A H Pharmaceuticals Ltd)	Angiotensin receptor blocker
54049	Losartan 50mg tablets (Accord Healthcare Ltd)	Angiotensin receptor blocker
51601	Losartan 50mg tablets (Actavis UK Ltd)	Angiotensin receptor blocker
54735	Losartan 50mg tablets (Alliance Healthcare (Distribution) Ltd)	Angiotensin receptor blocker
70325	Losartan 50mg tablets (Almus Pharmaceuticals Ltd)	Angiotensin receptor blocker

59750	Losartan 50mg tablets (Aptil Pharma Ltd)	Angiotensin receptor blocker
66551	Losartan 50mg tablets (Bristol Laboratories Ltd)	Angiotensin receptor blocker
74243	Losartan 50mg tablets (Consilient Health Ltd)	Angiotensin receptor blocker
54843	Losartan 50mg tablets (Dexcel-Pharma Ltd)	Angiotensin receptor blocker
55296	Losartan 50mg tablets (Mylan)	Angiotensin receptor blocker
71910	Losartan 50mg tablets (Necessity Supplies Ltd)	Angiotensin receptor blocker
59351	Losartan 50mg tablets (Pfizer Ltd)	Angiotensin receptor blocker
65094	Losartan 50mg tablets (Sandoz Ltd)	Angiotensin receptor blocker
54057	Losartan 50mg tablets (Teva UK Ltd)	Angiotensin receptor blocker
68603	Losartan 50mg tablets (Wockhardt UK Ltd)	Angiotensin receptor blocker
52659	Losartan 50mg/5ml oral solution	Angiotensin receptor blocker
59903	Losartan 50mg/5ml oral suspension	Angiotensin receptor blocker
17686	Micardis 20mg tablets (Boehringer Ingelheim Ltd)	Angiotensin receptor blocker
13821	Micardis 40mg tablets (Boehringer Ingelheim Ltd)	Angiotensin receptor blocker
17545	Micardis 80mg tablets (Boehringer Ingelheim Ltd)	Angiotensin receptor blocker
6217	Olmesartan medoxomil 10mg tablets	Angiotensin receptor blocker
39786	Olmesartan medoxomil 10mg/5ml oral suspension	Angiotensin receptor blocker
6285	Olmesartan medoxomil 20mg tablets	Angiotensin receptor blocker
6351	Olmesartan medoxomil 40mg tablets	Angiotensin receptor blocker
14983	Olmetec 10mg tablets (Daiichi Sankyo UK Ltd)	Angiotensin receptor blocker
18910	Olmetec 20mg tablets (Daiichi Sankyo UK Ltd)	Angiotensin receptor blocker
20117	Olmetec 40mg tablets (Daiichi Sankyo UK Ltd)	Angiotensin receptor blocker
63385	Sabervel 75mg tablets (Aspire Pharma Ltd)	Angiotensin receptor blocker
66702	Sacubitril 24mg / Valsartan 26mg tablets	Angiotensin receptor blocker
66197	Sacubitril 49mg / Valsartan 51mg tablets	Angiotensin receptor blocker
66931	Sacubitril 97mg / Valsartan 103mg tablets	Angiotensin receptor blocker

Carr MJ, *et al. BMJ Qual Saf* 2022; 31:503–514. doi: 10.1136/bmjqs-2021-013613

6243	Telmisartan 20mg tablets	Angiotensin receptor blocker
61177	Telmisartan 20mg tablets (Sigma Pharmaceuticals Plc)	Angiotensin receptor blocker
70251	Telmisartan 20mg tablets (Teva UK Ltd)	Angiotensin receptor blocker
5988	Telmisartan 40mg tablets	Angiotensin receptor blocker
65274	Telmisartan 40mg tablets (Actavis UK Ltd)	Angiotensin receptor blocker
12874	Telmisartan 80mg tablets	Angiotensin receptor blocker
9745	Teveten 300mg tablets (Mylan)	Angiotensin receptor blocker
16285	Teveten 400mg tablets (Abbott Healthcare Products Ltd)	Angiotensin receptor blocker
16371	Teveten 600mg tablets (Mylan)	Angiotensin receptor blocker
4645	Valsartan 160mg capsules	Angiotensin receptor blocker
68948	Valsartan 160mg capsules (Actavis UK Ltd)	Angiotensin receptor blocker
55187	Valsartan 160mg capsules (Arrow Generics Ltd)	Angiotensin receptor blocker
67663	Valsartan 160mg capsules (Dexcel-Pharma Ltd)	Angiotensin receptor blocker
53833	Valsartan 160mg capsules (Mylan)	Angiotensin receptor blocker
61442	Valsartan 160mg capsules (Teva UK Ltd)	Angiotensin receptor blocker
55821	Valsartan 160mg capsules (Teva UK Ltd)	Angiotensin receptor blocker
60076	Valsartan 160mg capsules (Waymade Healthcare Plc)	Angiotensin receptor blocker
44778	Valsartan 160mg tablets	Angiotensin receptor blocker
37573	Valsartan 320mg tablets	Angiotensin receptor blocker
59029	Valsartan 3mg/ml oral solution	Angiotensin receptor blocker
575	Valsartan 40mg capsules	Angiotensin receptor blocker
54726	Valsartan 40mg capsules (Teva UK Ltd)	Angiotensin receptor blocker
58669	Valsartan 40mg capsules (Teva UK Ltd)	Angiotensin receptor blocker
14943	Valsartan 40mg tablets	Angiotensin receptor blocker
74055	Valsartan 40mg/5ml oral solution	Angiotensin receptor blocker
74057	Valsartan 40mg/5ml oral suspension	Angiotensin receptor blocker

3222	Valsartan 80mg capsules	Angiotensin receptor blocker
59448	Valsartan 80mg capsules (A A H Pharmaceuticals Ltd)	Angiotensin receptor blocker
71028	Valsartan 80mg capsules (Arrow Generics Ltd)	Angiotensin receptor blocker
58910	Valsartan 80mg capsules (Sigma Pharmaceuticals Plc)	Angiotensin receptor blocker
38395	Valsartan 80mg tablets	Angiotensin receptor blocker
24677	ATENOLOL	Beta blocker
25037	ATENOLOL	Beta blocker
8172	Acebutolol 100mg capsules	Beta blocker
65438	Acebutolol 100mg capsules (A A H Pharmaceuticals Ltd)	Beta blocker
8113	Acebutolol 200mg capsules	Beta blocker
72545	Acebutolol 200mg capsules (A A H Pharmaceuticals Ltd)	Beta blocker
7620	Acebutolol 400mg tablets	Beta blocker
45309	Acebutolol 400mg tablets (A A H Pharmaceuticals Ltd)	Beta blocker
28048	Angilol 10mg Tablet (DDSA Pharmaceuticals Ltd)	Beta blocker
31833	Angilol 80mg Tablet (DDSA Pharmaceuticals Ltd)	Beta blocker
24195	Antipressan 100mg tablets (Teva UK Ltd)	Beta blocker
26211	Antipressan 25mg tablets (Teva UK Ltd)	Beta blocker
24191	Antipressan 50mg tablets (Teva UK Ltd)	Beta blocker
33836	Apsolol 160mg Tablet (Approved Prescription Services Ltd)	Beta blocker
27964	Apsolol 40mg Tablet (Approved Prescription Services Ltd)	Beta blocker
25644	Apsolox 80mg Tablet (Approved Prescription Services Ltd)	Beta blocker
29398	Atenamin 100mg Tablet (OPD Pharm)	Beta blocker
20728	Atenamin 25mg Tablet (OPD Pharm)	Beta blocker
21133	Atenamin 50mg Tablet (OPD Pharm)	Beta blocker
20502	Atenix 100 tablets (Ashbourne Pharmaceuticals Ltd)	Beta blocker
17322	Atenix 25 tablets (Ashbourne Pharmaceuticals Ltd)	Beta blocker
10191	Atenix 50 tablets (Ashbourne Pharmaceuticals Ltd)	Beta blocker
24	Atenolol 100mg tablets	Beta blocker
33085	Atenolol 100mg tablets (A A H Pharmaceuticals Ltd)	Beta blocker
46931	Atenolol 100mg tablets (Actavis UK Ltd)	Beta blocker
71026	Atenolol 100mg tablets (Almus Pharmaceuticals Ltd)	Beta blocker
31934	Atenolol 100mg tablets (IVAX Pharmaceuticals UK Ltd)	Beta blocker
46908	Atenolol 100mg tablets (Kent Pharmaceuticals Ltd)	Beta blocker
33079	Atenolol 100mg tablets (Mylan)	Beta blocker
34754	Atenolol 100mg tablets (Sandoz Ltd)	Beta blocker
19191	Atenolol 100mg tablets (Teva UK Ltd)	Beta blocker
33184	Atenolol 100mg tablets (Wockhardt UK Ltd)	Beta blocker
72810	Atenolol 10mg/5ml oral solution	Beta blocker
26	Atenolol 25mg tablets	Beta blocker
33657	Atenolol 25mg tablets (A A H Pharmaceuticals Ltd)	Beta blocker
59982	Atenolol 25mg tablets (Accord Healthcare Ltd)	Beta blocker
44858	Atenolol 25mg tablets (Actavis UK Ltd)	Beta blocker
50702	Atenolol 25mg tablets (Alliance Healthcare (Distribution) Ltd)	Beta blocker

Carr MJ, et al. BMJ Qual Saf 2022; 31:503–514. doi: 10.1136/bmjqs-2021-013613

47870	Atenolol 25mg tablets (Almus Pharmaceuticals Ltd)	Beta blocker
53826	Atenolol 25mg tablets (Boston Healthcare Ltd)	Beta blocker
49953	Atenolol 25mg tablets (Bristol Laboratories Ltd)	Beta blocker
52310	Atenolol 25mg tablets (Crescent Pharma Ltd)	Beta blocker
70135	Atenolol 25mg tablets (DE Pharmaceuticals)	Beta blocker
19172	Atenolol 25mg tablets (IVAX Pharmaceuticals UK Ltd)	Beta blocker
31536	Atenolol 25mg tablets (Kent Pharmaceuticals Ltd)	Beta blocker
34492	Atenolol 25mg tablets (Mylan)	Beta blocker
34585	Atenolol 25mg tablets (Sandoz Ltd)	Beta blocker
53802	Atenolol 25mg tablets (Sigma Pharmaceuticals Plc)	Beta blocker
51998	Atenolol 25mg tablets (Strides Shasun (UK) Ltd)	Beta blocker
29368	Atenolol 25mg tablets (Teva UK Ltd)	Beta blocker
34976	Atenolol 25mg tablets (Tillomed Laboratories Ltd)	Beta blocker
62325	Atenolol 25mg tablets (Waymade Healthcare Plc)	Beta blocker
34575	Atenolol 25mg tablets (Wockhardt UK Ltd)	Beta blocker
54542	Atenolol 25mg tablets (Zanza Laboratories Ltd)	Beta blocker
61573	Atenolol 25mg/5ml oral solution	Beta blocker
6066	Atenolol 25mg/5ml oral solution sugar free	Beta blocker
56445	Atenolol 25mg/5ml oral solution sugar free (A A H Pharmaceuticals Ltd)	Beta blocker
51643	Atenolol 25mg/5ml oral solution sugar free (Alliance Healthcare (Distribution) Ltd)	Beta blocker
34882	Atenolol 50mg Tablet (Berk Pharmaceuticals Ltd)	Beta blocker
5	Atenolol 50mg tablets	Beta blocker
33092	Atenolol 50mg tablets (A A H Pharmaceuticals Ltd)	Beta blocker
53414	Atenolol 50mg tablets (Accord Healthcare Ltd)	Beta blocker
33850	Atenolol 50mg tablets (Actavis UK Ltd)	Beta blocker
53204	Atenolol 50mg tablets (Alliance Healthcare (Distribution) Ltd)	Beta blocker
52500	Atenolol 50mg tablets (Almus Pharmaceuticals Ltd)	Beta blocker
59695	Atenolol 50mg tablets (Boston Healthcare Ltd)	Beta blocker
53215	Atenolol 50mg tablets (Bristol Laboratories Ltd)	Beta blocker
73151	Atenolol 50mg tablets (Crescent Pharma Ltd)	Beta blocker
66548	Atenolol 50mg tablets (DE Pharmaceuticals)	Beta blocker
19182	Atenolol 50mg tablets (IVAX Pharmaceuticals UK Ltd)	Beta blocker
34695	Atenolol 50mg tablets (Kent Pharmaceuticals Ltd)	Beta blocker
33650	Atenolol 50mg tablets (Mylan)	Beta blocker
55778	Atenolol 50mg tablets (Phoenix Healthcare Distribution Ltd)	Beta blocker
34265	Atenolol 50mg tablets (Sandoz Ltd)	Beta blocker
64973	Atenolol 50mg tablets (Sigma Pharmaceuticals Plc)	Beta blocker
54752	Atenolol 50mg tablets (Strides Shasun (UK) Ltd)	Beta blocker
34365	Atenolol 50mg tablets (Teva UK Ltd)	Beta blocker
36261	Atenolol 50mg tablets (Tillomed Laboratories Ltd)	Beta blocker
72043	Atenolol 50mg tablets (Waymade Healthcare Plc)	Beta blocker
34443	Atenolol 50mg tablets (Wockhardt UK Ltd)	Beta blocker
57817	Atenolol 50mg tablets (Zentiva)	Beta blocker
69526	Atenolol 50mg/5ml oral solution	Beta blocker
197	Atenolol 5mg/10ml solution for injection ampoules	Beta blocker
19810	BEDRANOL SR 80 MG CAP	Beta blocker

20813	BETALOC S.A.	Beta blocker
24378	BETALOC S.A. (CALENDAR PACK)	Beta blocker
22167	BETAXOLOL HYDROCHLORIDE	Beta blocker
47907	Bedranol SR 160mg capsules (Almus Pharmaceuticals Ltd)	Beta blocker
28996	Bedranol SR 160mg capsules (Sandoz Ltd)	Beta blocker
57063	Bedranol SR 80mg capsules (Almus Pharmaceuticals Ltd)	Beta blocker
47833	Bedranol SR 80mg capsules (Almus Pharmaceuticals Ltd)	Beta blocker
14808	Bedranol SR 80mg capsules (Sandoz Ltd)	Beta blocker
52136	Bedranol sr 160mg Capsule (Lagap)	Beta blocker
12495	Berkolol 10mg Tablet (Berk Pharmaceuticals Ltd)	Beta blocker
24218	Berkolol 160mg Tablet (Berk Pharmaceuticals Ltd)	Beta blocker
21866	Berkolol 40mg Tablet (Berk Pharmaceuticals Ltd)	Beta blocker
21839	Berkolol 80mg Tablet (Berk Pharmaceuticals Ltd)	Beta blocker
13487	Beta-Cardone 200mg tablets (Focus Pharmaceuticals Ltd)	Beta blocker
5858	Beta-Cardone 40mg tablets (Focus Pharmaceuticals Ltd)	Beta blocker
6751	Beta-Cardone 80mg tablets (Focus Pharmaceuticals Ltd)	Beta blocker
54623	Beta-Prograne 160mg modified-release capsules (Actavis UK Ltd)	Beta blocker
45877	Beta-Prograne 160mg modified-release capsules (Teva UK Ltd)	Beta blocker
26229	Beta-Prograne 160mg modified-release capsules (Tillomed Laboratories Ltd)	Beta blocker
23326	Betadur cr 160mg Modified-release capsule (Monmouth Pharmaceuticals Ltd)	Beta blocker
3344	Betaloc 100mg tablets (AstraZeneca UK Ltd)	Beta blocker
8071	Betaloc 50mg tablets (AstraZeneca UK Ltd)	Beta blocker
24461	Betaloc I.V. 5mg/5ml solution for injection ampoules (AstraZeneca UK Ltd)	Beta blocker
3474	Betaloc-SA 200mg tablets (AstraZeneca UK Ltd)	Beta blocker
12141	Betaxolol 20mg tablets	Beta blocker
12037	Betim 10mg Tablet (ICN Pharmaceuticals France S.A.)	Beta blocker
29610	Betim 10mg tablets (Meda Pharmaceuticals Ltd)	Beta blocker
21905	Bipranix 10mg tablets (Ashbourne Pharmaceuticals Ltd)	Beta blocker
21966	Bipranix 5mg tablets (Ashbourne Pharmaceuticals Ltd)	Beta blocker
39646	Bisoprolol 0.625mg/5ml oral solution	Beta blocker
50403	Bisoprolol 1.25mg Tablet (Teva UK Ltd)	Beta blocker
599	Bisoprolol 1.25mg tablets	Beta blocker
53885	Bisoprolol 1.25mg tablets (A A H Pharmaceuticals Ltd)	Beta blocker
51528	Bisoprolol 1.25mg tablets (Actavis UK Ltd)	Beta blocker
54479	Bisoprolol 1.25mg tablets (Alliance Healthcare (Distribution) Ltd)	Beta blocker
73809	Bisoprolol 1.25mg tablets (Almus Pharmaceuticals Ltd)	Beta blocker
52548	Bisoprolol 1.25mg tablets (Almus Pharmaceuticals Ltd)	Beta blocker
62361	Bisoprolol 1.25mg tablets (Chanelle Medical UK Ltd)	Beta blocker
72285	Bisoprolol 1.25mg tablets (Mawdsley-Brooks & Company Ltd)	Beta blocker
60761	Bisoprolol 1.25mg tablets (Medreich Plc)	Beta blocker
43251	Bisoprolol 1.25mg tablets (Mylan)	Beta blocker
58511	Bisoprolol 1.25mg tablets (Sandoz Ltd)	Beta blocker
59495	Bisoprolol 1.25mg tablets (Teva UK Ltd)	Beta blocker
65805	Bisoprolol 1.25mg tablets (Waymade Healthcare Plc)	Beta blocker
57626	Bisoprolol 1.25mg/5ml oral solution	Beta blocker
58109	Bisoprolol 1.25mg/5ml oral suspension	Beta blocker

822	Bisoprolol 1.5mg/5ml oral suspension	Beta blocker
67124	Bisoprolol 10mg / Aspirin 75mg capsules	Beta blocker
1290	Bisoprolol 10mg tablets	Beta blocker
53334	Bisoprolol 10mg tablets (A A H Pharmaceuticals Ltd)	Beta blocker
57176	Bisoprolol 10mg tablets (Accord Healthcare Ltd)	Beta blocker
33839	Bisoprolol 10mg tablets (Actavis UK Ltd)	Beta blocker
73641	Bisoprolol 10mg tablets (Almus Pharmaceuticals Ltd)	Beta blocker
58982	Bisoprolol 10mg tablets (Medreich Plc)	Beta blocker
34821	Bisoprolol 10mg tablets (Mylan)	Beta blocker
58973	Bisoprolol 10mg tablets (Niche Generics Ltd)	Beta blocker
19178	Bisoprolol 10mg tablets (Ranbaxy (UK) Ltd)	Beta blocker
55298	Bisoprolol 10mg tablets (Sigma Pharmaceuticals Plc)	Beta blocker
41591	Bisoprolol 10mg tablets (Teva UK Ltd)	Beta blocker
52611	Bisoprolol 10mg/5ml oral solution	Beta blocker
72540	Bisoprolol 10mg/5ml oral suspension	Beta blocker
37837	Bisoprolol 2.5mg Tablet (Teva UK Ltd)	Beta blocker
594	Bisoprolol 2.5mg tablets	Beta blocker
37118	Bisoprolol 2.5mg tablets (A A H Pharmaceuticals Ltd)	Beta blocker
56459	Bisoprolol 2.5mg tablets (Accord Healthcare Ltd)	Beta blocker
63493	Bisoprolol 2.5mg tablets (Actavis UK Ltd)	Beta blocker
58974	Bisoprolol 2.5mg tablets (Alliance Healthcare (Distribution) Ltd)	Beta blocker
53916	Bisoprolol 2.5mg tablets (Almus Pharmaceuticals Ltd)	Beta blocker
57023	Bisoprolol 2.5mg tablets (Almus Pharmaceuticals Ltd)	Beta blocker
50514	Bisoprolol 2.5mg tablets (Chanelle Medical UK Ltd)	Beta blocker
64850	Bisoprolol 2.5mg tablets (DE Pharmaceuticals)	Beta blocker
58498	Bisoprolol 2.5mg tablets (Medreich Plc)	Beta blocker
47041	Bisoprolol 2.5mg tablets (Mylan)	Beta blocker
56768	Bisoprolol 2.5mg tablets (Niche Generics Ltd)	Beta blocker
53664	Bisoprolol 2.5mg tablets (Sandoz Ltd)	Beta blocker
63850	Bisoprolol 2.5mg tablets (Teva UK Ltd)	Beta blocker
58763	Bisoprolol 2.5mg tablets (Waymade Healthcare Plc)	Beta blocker
59148	Bisoprolol 2.5mg tablets (Zentiva)	Beta blocker
52686	Bisoprolol 2.5mg/5ml oral solution	Beta blocker
44000	Bisoprolol 2.5mg/5ml oral suspension	Beta blocker
7091	Bisoprolol 3.75mg tablets	Beta blocker
55791	Bisoprolol 3.75mg tablets (Actavis UK Ltd)	Beta blocker
60502	Bisoprolol 3.75mg tablets (DE Pharmaceuticals)	Beta blocker
69156	Bisoprolol 3.75mg tablets (Medreich Plc)	Beta blocker
56240	Bisoprolol 3.75mg tablets (Sandoz Ltd)	Beta blocker
64538	Bisoprolol 3.75mg tablets (Teva UK Ltd)	Beta blocker
61564	Bisoprolol 3.75mg tablets (Waymade Healthcare Plc)	Beta blocker
65027	Bisoprolol 5mg / Aspirin 100mg capsules	Beta blocker
43564	Bisoprolol 5mg Tablet (PLIVA Pharma Ltd)	Beta blocker
472	Bisoprolol 5mg tablets	Beta blocker
59037	Bisoprolol 5mg tablets (A A H Pharmaceuticals Ltd)	Beta blocker
55929	Bisoprolol 5mg tablets (Accord Healthcare Ltd)	Beta blocker
34963	Bisoprolol 5mg tablets (Actavis UK Ltd)	Beta blocker

52635	Bisoprolol 5mg tablets (Alliance Healthcare (Distribution) Ltd)	Beta blocker
59969	Bisoprolol 5mg tablets (Almus Pharmaceuticals Ltd)	Beta blocker
73638	Bisoprolol 5mg tablets (Almus Pharmaceuticals Ltd)	Beta blocker
61340	Bisoprolol 5mg tablets (DE Pharmaceuticals)	Beta blocker
19200	Bisoprolol 5mg tablets (IVAX Pharmaceuticals UK Ltd)	Beta blocker
60896	Bisoprolol 5mg tablets (Medreich Plc)	Beta blocker
32114	Bisoprolol 5mg tablets (Mylan)	Beta blocker
64784	Bisoprolol 5mg tablets (Niche Generics Ltd)	Beta blocker
63535	Bisoprolol 5mg tablets (Relonchem Ltd)	Beta blocker
57934	Bisoprolol 5mg tablets (Sandoz Ltd)	Beta blocker
24083	Bisoprolol 5mg tablets (Teva UK Ltd)	Beta blocker
71472	Bisoprolol 5mg tablets (Waymade Healthcare Plc)	Beta blocker
61115	Bisoprolol 5mg/5ml oral solution	Beta blocker
7553	Bisoprolol 5mg/5ml oral suspension	Beta blocker
74742	Bisoprolol 625micrograms/5ml oral suspension	Beta blocker
5713	Bisoprolol 7.5mg tablets	Beta blocker
38991	Bisoprolol 7.5mg tablets (A A H Pharmaceuticals Ltd)	Beta blocker
61651	Bisoprolol 7.5mg tablets (Almus Pharmaceuticals Ltd)	Beta blocker
58455	Bisoprolol 7.5mg tablets (Sandoz Ltd)	Beta blocker
65821	Bisoprolol 7.5mg/5ml oral suspension	Beta blocker
62407	Bisoprolol oral solution	Beta blocker
7852	Blocadren 10mg Tablet (Merck Sharp & Dohme Ltd)	Beta blocker
32135	Brevibloc Concentrate 2.5g/10ml solution for infusion ampoules (Baxter Healthcare Ltd)	Beta blocker
26922	Brevibloc Premixed 100mg/10ml solution for injection vials (Baxter Healthcare Ltd)	Beta blocker
18722	CARVEDILOL	Beta blocker
22796	CARVEDILOL 3.125 MG	Beta blocker
14058	Cardicor 1.25mg tablets (Merck Serono Ltd)	Beta blocker
19858	Cardicor 10mg tablets (Merck Serono Ltd)	Beta blocker
14030	Cardicor 2.5mg tablets (Merck Serono Ltd)	Beta blocker
57578	Cardicor 2.5mg tablets (Necessity Supplies Ltd)	Beta blocker
19853	Cardicor 3.75mg tablets (Merck Serono Ltd)	Beta blocker
17615	Cardicor 5mg tablets (Merck Serono Ltd)	Beta blocker
18185	Cardicor 7.5mg tablets (Merck Serono Ltd)	Beta blocker
29827	Carteolol HCl 10mg tablets	Beta blocker
28700	Cartrol 10mg Tablet (Novartis Consumer Health UK Ltd)	Beta blocker
54106	Carvedilol 1.5mg/5ml oral suspension	Beta blocker
2629	Carvedilol 12.5mg tablets	Beta blocker
34501	Carvedilol 12.5mg tablets (Actavis UK Ltd)	Beta blocker
33374	Carvedilol 12.5mg tablets (Genus Pharmaceuticals Ltd)	Beta blocker
74619	Carvedilol 12.5mg tablets (Teva UK Ltd)	Beta blocker
63422	Carvedilol 12.5mg tablets (Waymade Healthcare Plc)	Beta blocker
7049	Carvedilol 25mg tablets	Beta blocker
72507	Carvedilol 25mg tablets (Teva UK Ltd)	Beta blocker
817	Carvedilol 3.125mg tablets	Beta blocker
46936	Carvedilol 3.125mg tablets (A A H Pharmaceuticals Ltd)	Beta blocker

46935	Carvedilol 3.125mg tablets (Actavis UK Ltd)	Beta blocker
34741	Carvedilol 3.125mg tablets (IVAX Pharmaceuticals UK Ltd)	Beta blocker
61663	Carvedilol 3.125mg tablets (Teva UK Ltd)	Beta blocker
49142	Carvedilol 3.125mg/5ml oral suspension	Beta blocker
47107	Carvedilol 5mg/5ml oral suspension	Beta blocker
59549	Carvedilol 5mg/5ml oral suspension	Beta blocker
4410	Carvedilol 6.25mg tablets	Beta blocker
34740	Carvedilol 6.25mg tablets (Actavis UK Ltd)	Beta blocker
73451	Carvedilol 6.25mg tablets (Almus Pharmaceuticals Ltd)	Beta blocker
67661	Carvedilol 6.25mg tablets (Sigma Pharmaceuticals Plc)	Beta blocker
19202	Carvedilol 6.25mg tablets (Teva UK Ltd)	Beta blocker
4265	Celectol 200mg Tablet (Pantheon Healthcare Ltd)	Beta blocker
57573	Celectol 200mg tablets (Dowelhurst Ltd)	Beta blocker
67292	Celectol 200mg tablets (Sigma Pharmaceuticals Plc)	Beta blocker
56485	Celectol 200mg tablets (Waymade Healthcare Plc)	Beta blocker
35054	Celectol 200mg tablets (Zentiva)	Beta blocker
16776	Celectol 400mg Tablet (Pantheon Healthcare Ltd)	Beta blocker
74062	Celectol 400mg tablets (Sigma Pharmaceuticals Plc)	Beta blocker
35940	Celectol 400mg tablets (Zentiva)	Beta blocker
8262	Celiprolol 200mg tablets	Beta blocker
42795	Celiprolol 200mg tablets (Mylan)	Beta blocker
41740	Celiprolol 200mg tablets (Teva UK Ltd)	Beta blocker
7974	Celiprolol 400mg tablets	Beta blocker
74623	Celiprolol 400mg tablets (Mylan)	Beta blocker
25462	Clopamide 5mg with Pindolol 10mg tablets	Beta blocker
50300	Congescor 1.25mg tablets (Teva UK Ltd)	Beta blocker
33909	Congescor 1.25mg tablets (Tillomed Laboratories Ltd)	Beta blocker
50224	Congescor 2.5mg tablets (Teva UK Ltd)	Beta blocker
32552	Congescor 2.5mg tablets (Tillomed Laboratories Ltd)	Beta blocker
13415	Corgard 40mg tablets (Sanofi-Synthelabo Ltd)	Beta blocker
10716	Corgard 80mg tablets (Sanofi)	Beta blocker
10892	Emcor 10mg tablets (Merck Serono Ltd)	Beta blocker
4771	Emcor LS 5mg tablets (Merck Serono Ltd)	Beta blocker
39819	Esmolol 2.5g/250ml infusion bags	Beta blocker
30541	Esmolol HCl 250mg/ml concentrate solution for infusion	Beta blocker
18414	Eucardic 12.5mg tablets (Roche Products Ltd)	Beta blocker
19437	Eucardic 25mg tablets (Roche Products Ltd)	Beta blocker
14117	Eucardic 3.125mg tablets (Roche Products Ltd)	Beta blocker
14146	Eucardic 6.25mg tablets (Roche Products Ltd)	Beta blocker
25052	HALF-INDERAL LA	Beta blocker
47543	Half Beta-Prograne 80mg modified-release capsules (Actavis UK Ltd)	Beta blocker
56173	Half Beta-Prograne 80mg modified-release capsules (Actavis UK Ltd)	Beta blocker
46363	Half Beta-Prograne 80mg modified-release capsules (Teva UK Ltd)	Beta blocker
20468	Half Beta-Prograne 80mg modified-release capsules (Tillomed Laboratories Ltd)	Beta blocker
1006	Half Inderal LA 80mg capsules (AstraZeneca UK Ltd)	Beta blocker
22208	Half propanix la 80mg Modified-release capsule (Ashbourne	Beta blocker

	Pharmaceuticals Ltd)	
28788	Half propatard la 80mg Modified-release capsule (Galen Ltd)	Beta blocker
15619	Half-betadur cr 80mg Capsule (Monmouth Pharmaceuticals Ltd)	Beta blocker
2414	Inderal 10mg tablets (AstraZeneca UK Ltd)	Beta blocker
8331	Inderal 160mg Tablet (AstraZeneca UK Ltd)	Beta blocker
10294	Inderal 1mg/1ml solution for injection ampoules (AstraZeneca UK Ltd)	Beta blocker
1050	Inderal 40mg tablets (AstraZeneca UK Ltd)	Beta blocker
1048	Inderal 80mg tablets (AstraZeneca UK Ltd)	Beta blocker
3005	Inderal LA 160mg capsules (AstraZeneca UK Ltd)	Beta blocker
52609	Inderal LA 160mg capsules (Sigma Pharmaceuticals Plc)	Beta blocker
21981	KERLONE	Beta blocker
12519	Kerlone 20mg tablets (Sanofi-Synthelabo Ltd)	Beta blocker
7491	LABETALOL TAB	Beta blocker
16669	LOPRESOR SR 200 MG TAB	Beta blocker
72514	Labetalol 1.5mg/5ml oral solution	Beta blocker
34171	Labetalol 100mg Tablet (C P Pharmaceuticals Ltd)	Beta blocker
1597	Labetalol 100mg tablets	Beta blocker
34177	Labetalol 100mg tablets (A A H Pharmaceuticals Ltd)	Beta blocker
62638	Labetalol 100mg tablets (Actavis UK Ltd)	Beta blocker
41827	Labetalol 100mg tablets (Mylan)	Beta blocker
63736	Labetalol 100mg tablets (Waymade Healthcare Plc)	Beta blocker
38370	Labetalol 100mg/20ml solution for injection ampoules	Beta blocker
59222	Labetalol 100mg/20ml solution for injection ampoules (RPH Pharmaceuticals AB)	Beta blocker
47674	Labetalol 200mg Tablet (C P Pharmaceuticals Ltd)	Beta blocker
34188	Labetalol 200mg Tablet (Celltech Pharma Europe Ltd)	Beta blocker
2775	Labetalol 200mg tablets	Beta blocker
30770	Labetalol 200mg tablets (A A H Pharmaceuticals Ltd)	Beta blocker
44083	Labetalol 200mg tablets (Actavis UK Ltd)	Beta blocker
47673	Labetalol 400mg Tablet (Approved Prescription Services Ltd)	Beta blocker
1295	Labetalol 400mg tablets	Beta blocker
40240	Labetalol 400mg tablets (A A H Pharmaceuticals Ltd)	Beta blocker
45250	Labetalol 400mg tablets (Sandoz Ltd)	Beta blocker
4725	Labetalol 50mg tablets	Beta blocker
19068	Labetalol 50mg/10ml solution for injection pre-filled syringes	Beta blocker
35778	Labrocol 100mg Tablet (Lagap)	Beta blocker
22793	Labrocol 200mg Tablet (Lagap)	Beta blocker
16645	Labrocol 400mg Tablet (Lagap)	Beta blocker
26255	Lopranol la 160mg Capsule (Opus Pharmaceuticals Ltd)	Beta blocker
13499	Lopresor 100mg Tablet (Novartis Pharmaceuticals UK Ltd)	Beta blocker
46740	Lopresor 100mg tablets (Recordati Pharmaceuticals Ltd)	Beta blocker
10429	Lopresor 50mg Tablet (Novartis Pharmaceuticals UK Ltd)	Beta blocker
46614	Lopresor 50mg tablets (Recordati Pharmaceuticals Ltd)	Beta blocker
20082	Lopresor SR 200mg tablets (Recordati Pharmaceuticals Ltd)	Beta blocker
17876	METOPROLOL FUMARATE 190 MG TAB	Beta blocker
28493	METOPROLOL FUMARATE 95 MG TAB	Beta blocker
30400	Mepranix 100mg Tablet (Ashbourne Pharmaceuticals Ltd)	Beta blocker

Carr MJ, et al. BMJ Qual Saf 2022; 31:503–514. doi: 10.1136/bmjqs-2021-013613

29762	Mepranix 50mg Tablet (Ashbourne Pharmaceuticals Ltd)	Beta blocker
753	Metoprolol 100mg tablets	Beta blocker
34125	Metoprolol 100mg tablets (A A H Pharmaceuticals Ltd)	Beta blocker
34854	Metoprolol 100mg tablets (Actavis UK Ltd)	Beta blocker
66670	Metoprolol 100mg tablets (Alliance Healthcare (Distribution) Ltd)	Beta blocker
40167	Metoprolol 100mg tablets (IVAX Pharmaceuticals UK Ltd)	Beta blocker
34509	Metoprolol 100mg tablets (Mylan)	Beta blocker
34092	Metoprolol 100mg tablets (Teva UK Ltd)	Beta blocker
63724	Metoprolol 100mg tablets (Waymade Healthcare Plc)	Beta blocker
68881	Metoprolol 12.5mg capsules	Beta blocker
65227	Metoprolol 12.5mg/5ml oral solution	Beta blocker
51447	Metoprolol 12.5mg/5ml oral suspension	Beta blocker
8068	Metoprolol 200mg modified-release tablets	Beta blocker
74854	Metoprolol 25mg/5ml oral solution	Beta blocker
55979	Metoprolol 25mg/5ml oral suspension	Beta blocker
34890	Metoprolol 50mg Tablet (Berk Pharmaceuticals Ltd)	Beta blocker
739	Metoprolol 50mg tablets	Beta blocker
34094	Metoprolol 50mg tablets (A A H Pharmaceuticals Ltd)	Beta blocker
71098	Metoprolol 50mg tablets (Accord Healthcare Ltd)	Beta blocker
34430	Metoprolol 50mg tablets (Actavis UK Ltd)	Beta blocker
34584	Metoprolol 50mg tablets (IVAX Pharmaceuticals UK Ltd)	Beta blocker
32836	Metoprolol 50mg tablets (Mylan)	Beta blocker
34925	Metoprolol 50mg tablets (Sandoz Ltd)	Beta blocker
34407	Metoprolol 50mg tablets (Teva UK Ltd)	Beta blocker
70116	Metoprolol 50mg/5ml oral solution	Beta blocker
11793	Metoprolol 50mg/5ml oral suspension	Beta blocker
57240	Metoprolol 50mg/5ml oral suspension (Special Order)	Beta blocker
14502	Metoprolol 5mg/5ml solution for injection ampoules	Beta blocker
47536	Metoprolol tartrate 12.5mg/5ml Oral suspension	Beta blocker
75010	Metoprolol tartrate 50mg Tablet (C P Pharmaceuticals Ltd)	Beta blocker
45289	Metoprolol tartrate Oral solution	Beta blocker
29998	Metoros 190mg Tablet (Novartis Pharmaceuticals UK Ltd)	Beta blocker
27719	Metoros ls 95mg Tablet (Geigy Pharmaceuticals)	Beta blocker
56486	Monocor 10mg tablets (Dowelhurst Ltd)	Beta blocker
5968	Monocor 10mg tablets (Wyeth Pharmaceuticals)	Beta blocker
3588	Monocor 5mg tablets (Wyeth Pharmaceuticals)	Beta blocker
72159	Nadolol 20mg/5ml oral suspension	Beta blocker
66779	Nadolol 30mg/5ml oral suspension	Beta blocker
74930	Nadolol 30mg/5ml oral suspension (Drug Tariff Special Order)	Beta blocker
8935	Nadolol 40mg tablets	Beta blocker
66464	Nadolol 40mg/5ml oral solution	Beta blocker
73219	Nadolol 40mg/5ml oral suspension	Beta blocker
2499	Nadolol 80mg tablets	Beta blocker
67424	Nadolol 80mg/5ml oral suspension	Beta blocker
74929	Nadolol Oral solution	Beta blocker
7528	Nebilet 5mg tablets (A. Menarini Farmaceutica Internazionale SRL)	Beta blocker
66559	Nebilet 5mg tablets (Waymade Healthcare Plc)	Beta blocker

59961	Nebivolol 10mg tablets	Beta blocker
40761	Nebivolol 2.5mg tablets	Beta blocker
44808	Nebivolol 2.5mg tablets (A A H Pharmaceuticals Ltd)	Beta blocker
47300	Nebivolol 2.5mg tablets (Glenmark Pharmaceuticals Europe Ltd)	Beta blocker
54487	Nebivolol 2.5mg tablets (Sigma Pharmaceuticals Plc)	Beta blocker
751	Nebivolol 5mg tablets	Beta blocker
69115	Nebivolol 5mg tablets (A A H Pharmaceuticals Ltd)	Beta blocker
68677	Nebivolol 5mg tablets (Accord Healthcare Ltd)	Beta blocker
67595	Nebivolol 5mg tablets (Almus Pharmaceuticals Ltd)	Beta blocker
64703	Nebivolol 5mg tablets (Glenmark Pharmaceuticals Europe Ltd)	Beta blocker
74076	Nebivolol 5mg tablets (PLIVA Pharma Ltd)	Beta blocker
71032	Nebivolol 5mg tablets (Sandoz Ltd)	Beta blocker
12497	OXPRENOLOL 10 MG TAB	Beta blocker
3748	Oxprenolol 160mg Tablet	Beta blocker
1334	Oxprenolol 160mg modified-release tablets	Beta blocker
3516	Oxprenolol 20mg tablets	Beta blocker
27357	Oxprenolol 40mg Tablet (Actavis UK Ltd)	Beta blocker
1333	Oxprenolol 40mg tablets	Beta blocker
2780	Oxprenolol 80mg tablets	Beta blocker
33569	Oxprenolol sr 160mg Modified-release tablet (Hillcross Pharmaceuticals Ltd)	Beta blocker
21885	Oxyprenix SR 160mg tablets	Beta blocker
25764	PINDOLOL 10MG/CLOPAMIDE 5MG	Beta blocker
32470	PROPRANOLOL 1 MG LIQ	Beta blocker
22634	PROPRANOLOL 10 MG SUS	Beta blocker
46493	PROPRANOLOL 15 MG SYR	Beta blocker
26788	PROPRANOLOL 2.5 MG ELI	Beta blocker
4021	PROPRANOLOL 20 MG TAB	Beta blocker
29803	PROPRANOLOL 3 MG ELI	Beta blocker
25818	PROPRANOLOL 30 MG SUS	Beta blocker
27036	PROPRANOLOL POWDERS 5 MG POW	Beta blocker
23604	PROPRANOLOL S/R	Beta blocker
26105	PROPRANOLOL paed 4 MG TAB	Beta blocker
14057	Pindolol 10mg / Clopamide 5mg tablets	Beta blocker
55853	Pindolol 15mg Tablet (Hillcross Pharmaceuticals Ltd)	Beta blocker
14673	Pindolol 15mg tablets	Beta blocker
5284	Pindolol 5mg tablets	Beta blocker
73413	Pindolol 5mg tablets (A A H Pharmaceuticals Ltd)	Beta blocker
33376	Probeta LA 160mg Capsule (Trinity Pharmaceuticals Ltd)	Beta blocker
14552	Propanix 10mg Tablet (Ashbourne Pharmaceuticals Ltd)	Beta blocker
8978	Propanix 160mg Modified-release capsule (Ashbourne Pharmaceuticals Ltd)	Beta blocker
29763	Propanix 160mg Tablet (Ashbourne Pharmaceuticals Ltd)	Beta blocker
3827	Propanix 40mg Tablet (Ashbourne Pharmaceuticals Ltd)	Beta blocker
21838	Propanix 80mg Tablet (Ashbourne Pharmaceuticals Ltd)	Beta blocker
26228	Propanix LA 160mg Modified-release capsule (Ashbourne Pharmaceuticals Ltd)	Beta blocker
297	Propranolol 10mg tablets	Beta blocker
34378	Propranolol 10mg tablets (A A H Pharmaceuticals Ltd)	Beta blocker

61727	Propranolol 10mg tablets (Accord Healthcare Ltd)	Beta blocker
34783	Propranolol 10mg tablets (Actavis UK Ltd)	Beta blocker
65986	Propranolol 10mg tablets (Alliance Healthcare (Distribution) Ltd)	Beta blocker
45494	Propranolol 10mg tablets (Almus Pharmaceuticals Ltd)	Beta blocker
66555	Propranolol 10mg tablets (Boston Healthcare Ltd)	Beta blocker
43525	Propranolol 10mg tablets (IVAX Pharmaceuticals UK Ltd)	Beta blocker
58297	Propranolol 10mg tablets (Kent Pharmaceuticals Ltd)	Beta blocker
36576	Propranolol 10mg tablets (Mylan)	Beta blocker
73765	Propranolol 10mg tablets (Ranbaxy (UK) Ltd)	Beta blocker
34804	Propranolol 10mg tablets (Teva UK Ltd)	Beta blocker
65435	Propranolol 10mg tablets (Waymade Healthcare Plc)	Beta blocker
5478	Propranolol 10mg/5ml oral solution sugar free	Beta blocker
72220	Propranolol 10mg/5ml oral solution sugar free (CST Pharma Ltd)	Beta blocker
57567	Propranolol 10mg/5ml oral suspension	Beta blocker
34949	Propranolol 160mg Modified-release capsule (Actavis UK Ltd)	Beta blocker
34945	Propranolol 160mg Modified-release capsule (Lagap)	Beta blocker
34884	Propranolol 160mg Modified-release capsule (Sandoz Ltd)	Beta blocker
1448	Propranolol 160mg modified-release capsules	Beta blocker
59597	Propranolol 160mg modified-release capsules (A A H Pharmaceuticals Ltd)	Beta blocker
3167	Propranolol 160mg tablets	Beta blocker
34214	Propranolol 160mg tablets (Actavis UK Ltd)	Beta blocker
68400	Propranolol 160mg tablets (DE Pharmaceuticals)	Beta blocker
55849	Propranolol 160mg tablets (Mylan)	Beta blocker
27486	Propranolol 1mg/1ml solution for injection ampoules	Beta blocker
69661	Propranolol 3mg/5ml oral solution	Beta blocker
70681	Propranolol 3mg/5ml oral suspension	Beta blocker
707	Propranolol 40mg tablets	Beta blocker
41555	Propranolol 40mg tablets (A A H Pharmaceuticals Ltd)	Beta blocker
59415	Propranolol 40mg tablets (Accord Healthcare Ltd)	Beta blocker
27700	Propranolol 40mg tablets (Actavis UK Ltd)	Beta blocker
58491	Propranolol 40mg tablets (Alliance Healthcare (Distribution) Ltd)	Beta blocker
55416	Propranolol 40mg tablets (Almus Pharmaceuticals Ltd)	Beta blocker
55228	Propranolol 40mg tablets (Boston Healthcare Ltd)	Beta blocker
45297	Propranolol 40mg tablets (IVAX Pharmaceuticals UK Ltd)	Beta blocker
52777	Propranolol 40mg tablets (Kent Pharmaceuticals Ltd)	Beta blocker
31776	Propranolol 40mg tablets (Mylan)	Beta blocker
57342	Propranolol 40mg tablets (Phoenix Healthcare Distribution Ltd)	Beta blocker
60565	Propranolol 40mg tablets (Ranbaxy (UK) Ltd)	Beta blocker
34868	Propranolol 40mg tablets (Teva UK Ltd)	Beta blocker
56764	Propranolol 40mg tablets (Waymade Healthcare Plc)	Beta blocker
55949	Propranolol 40mg/5ml oral solution	Beta blocker
3087	Propranolol 40mg/5ml oral solution sugar free	Beta blocker
38433	Propranolol 50mg/5ml Oral solution (Rosemont Pharmaceuticals Ltd)	Beta blocker
11711	Propranolol 50mg/5ml oral solution	Beta blocker
54297	Propranolol 50mg/5ml oral solution	Beta blocker
48682	Propranolol 50mg/5ml oral solution sugar free	Beta blocker
71150	Propranolol 5mg/5ml oral solution	Beta blocker

220	Propranolol 5mg/5ml oral solution	Beta blocker
49863	Propranolol 5mg/5ml oral solution sugar free	Beta blocker
64160	Propranolol 5mg/5ml oral solution sugar free (AM Distributions (Yorkshire) Ltd)	Beta blocker
70680	Propranolol 6mg/5ml oral suspension	Beta blocker
34867	Propranolol 80mg Capsule (IVAX Pharmaceuticals UK Ltd)	Beta blocker
28128	Propranolol 80mg Modified-release capsule (Actavis UK Ltd)	Beta blocker
32162	Propranolol 80mg Modified-release capsule (Lagap)	Beta blocker
769	Propranolol 80mg modified-release capsules	Beta blocker
35938	Propranolol 80mg modified-release capsules (A A H Pharmaceuticals Ltd)	Beta blocker
72222	Propranolol 80mg modified-release capsules (DE Pharmaceuticals)	Beta blocker
60934	Propranolol 80mg modified-release capsules (Kent Pharmaceuticals Ltd)	Beta blocker
71173	Propranolol 80mg modified-release capsules (Mawdsley-Brooks & Company Ltd)	Beta blocker
39233	Propranolol 80mg modified-release capsules (Teva UK Ltd)	Beta blocker
62711	Propranolol 80mg modified-release capsules (Waymade Healthcare Plc)	Beta blocker
940	Propranolol 80mg tablets	Beta blocker
33644	Propranolol 80mg tablets (A A H Pharmaceuticals Ltd)	Beta blocker
31214	Propranolol 80mg tablets (Mylan)	Beta blocker
73653	Propranolol 80mg tablets (Ranbaxy (UK) Ltd)	Beta blocker
58407	Propranolol 80mg tablets (Teva UK Ltd)	Beta blocker
9185	Propranolol 80mg/5ml oral solution	Beta blocker
40241	Propranolol LA 160mg Capsule (Approved Prescription Services Ltd)	Beta blocker
34185	Propranolol LA 80mg Modified-release capsule (Approved Prescription Services Ltd)	Beta blocker
34208	Propranolol SR 160mg Modified-release capsule (C P Pharmaceuticals Ltd)	Beta blocker
36603	Propranolol SR 160mg Modified-release capsule (Hillcross Pharmaceuticals Ltd)	Beta blocker
45343	Propranolol SR 80mg Modified-release capsule (C P Pharmaceuticals Ltd)	Beta blocker
53177	Propranolol oral solution	Beta blocker
25359	Rapranol SR 160mg capsules (Ranbaxy (UK) Ltd)	Beta blocker
25367	Rapranol SR 80mg capsules (Ranbaxy (UK) Ltd)	Beta blocker
26290	SOTACOR 40 MG INJ	Beta blocker
3041	SOTALOL HCl 40 MG INJ	Beta blocker
12119	SOTALOL HCl S/R 80 MG TAB	Beta blocker
23598	SOTALOL HYDROCHLORIDE S/R	Beta blocker
12296	Sectral 100mg capsules (Sanofi)	Beta blocker
8555	Sectral 200mg capsules (Sanofi)	Beta blocker
8023	Sectral 400mg tablets (Sanofi)	Beta blocker
33602	Slo-Pro 160mg capsules (Mylan)	Beta blocker
23587	Sloprolol 160mg Capsule (C P Pharmaceuticals Ltd)	Beta blocker
4025	Slow-Trasicor 160mg tablets (AMCo)	Beta blocker
74015	Slow-Trasicor 160mg tablets (Sigma Pharmaceuticals Plc)	Beta blocker
29230	Slow-pren 160mg Tablet (IVAX Pharmaceuticals UK Ltd)	Beta blocker
24635	Sotacor 10mg/ml Injection (Bristol-Myers Squibb Pharmaceuticals Ltd)	Beta blocker
11380	Sotacor 160mg tablets (Bristol-Myers Squibb Pharmaceuticals Ltd)	Beta blocker
33578	Sotacor 40mg/4ml solution for injection ampoules (Bristol-Myers Squibb Pharmaceuticals Ltd)	Beta blocker

4004	Sotacor 80mg tablets (Bristol-Myers Squibb Pharmaceuticals Ltd)	Beta blocker
17679	Sotalol 10mg/ml injection	Beta blocker
9292	Sotalol 160mg tablets	Beta blocker
73481	Sotalol 160mg tablets (Teva UK Ltd)	Beta blocker
13051	Sotalol 200mg tablets	Beta blocker
51492	Sotalol 25mg/5ml oral solution	Beta blocker
35710	Sotalol 25mg/5ml oral suspension	Beta blocker
27727	Sotalol 2mg/ml injection	Beta blocker
34640	Sotalol 40mg Tablet (Tillomed Laboratories Ltd)	Beta blocker
786	Sotalol 40mg tablets	Beta blocker
34371	Sotalol 40mg tablets (A A H Pharmaceuticals Ltd)	Beta blocker
70734	Sotalol 40mg tablets (Almus Pharmaceuticals Ltd)	Beta blocker
71479	Sotalol 40mg tablets (Bristol Laboratories Ltd)	Beta blocker
43549	Sotalol 40mg tablets (IVAX Pharmaceuticals UK Ltd)	Beta blocker
74829	Sotalol 40mg tablets (Phoenix Healthcare Distribution Ltd)	Beta blocker
34600	Sotalol 40mg tablets (Teva UK Ltd)	Beta blocker
38498	Sotalol 40mg/4ml solution for injection ampoules	Beta blocker
70161	Sotalol 40mg/5ml oral solution	Beta blocker
70162	Sotalol 40mg/5ml oral suspension	Beta blocker
1572	Sotalol 80mg tablets	Beta blocker
39423	Sotalol 80mg tablets (A A H Pharmaceuticals Ltd)	Beta blocker
34520	Sotalol 80mg tablets (Mylan)	Beta blocker
34690	Sotalol 80mg tablets (Sandoz Ltd)	Beta blocker
74628	Sotalol 80mg tablets (Teva UK Ltd)	Beta blocker
72347	Sotalol 80mg/5ml oral suspension	Beta blocker
75060	Sotalol oral solution	Beta blocker
26895	Syprol 10mg/5ml oral solution (Rosemont Pharmaceuticals Ltd)	Beta blocker
45765	Syprol 40mg/5ml oral solution (Rosemont Pharmaceuticals Ltd)	Beta blocker
42152	Syprol 50mg/5ml oral solution (Rosemont Pharmaceuticals Ltd)	Beta blocker
17082	Syprol 5mg/5ml oral solution (Rosemont Pharmaceuticals Ltd)	Beta blocker
26807	TENORETIC	Beta blocker
13309	TRASICOR 2 MG INJ	Beta blocker
2587	Tenormin 100mg tablets (AstraZeneca UK Ltd)	Beta blocker
2590	Tenormin 25mg tablets (AstraZeneca UK Ltd)	Beta blocker
13394	Tenormin 25mg/5ml syrup (AstraZeneca UK Ltd)	Beta blocker
7429	Tenormin 5mg/10ml solution for injection ampoules (AstraZeneca UK Ltd)	Beta blocker
2432	Tenormin LS 50mg tablets (AstraZeneca UK Ltd)	Beta blocker
7853	Timolol 10mg tablets	Beta blocker
15730	Totamol 100mg Tablet (C P Pharmaceuticals Ltd)	Beta blocker
18950	Totamol 25mg Tablet (C P Pharmaceuticals Ltd)	Beta blocker
15176	Totamol 50mg Tablet (C P Pharmaceuticals Ltd)	Beta blocker
9016	Trandate 100mg tablets (RPH Pharmaceuticals AB)	Beta blocker
19998	Trandate 100mg/20ml solution for injection ampoules (Focus Pharmaceuticals Ltd)	Beta blocker
8707	Trandate 200mg tablets (RPH Pharmaceuticals AB)	Beta blocker
8807	Trandate 400mg tablets (RPH Pharmaceuticals AB)	Beta blocker
9273	Trandate 50mg tablets (RPH Pharmaceuticals AB)	Beta blocker

Carr MJ, *et al. BMJ Qual Saf* 2022; 31:503–514. doi: 10.1136/bmjqs-2021-013613

10777	Trasicor 160mg Tablet (Novartis Pharmaceuticals UK Ltd)	Beta blocker
7474	Trasicor 20mg Tablet (Novartis Pharmaceuticals UK Ltd)	Beta blocker
35062	Trasicor 20mg tablets (Amdipharm Plc)	Beta blocker
8290	Trasicor 40mg Tablet (Novartis Pharmaceuticals UK Ltd)	Beta blocker
24094	Trasicor 40mg tablets (Amdipharm Plc)	Beta blocker
2361	Trasicor 80mg Tablet (Novartis Pharmaceuticals UK Ltd)	Beta blocker
29180	Trasicor 80mg tablets (Amdipharm Plc)	Beta blocker
30636	Vasaten 50mg Tablet (Shire Pharmaceuticals Ltd)	Beta blocker
9143	Viskaldix tablets (AMCo)	Beta blocker
20012	Visken 15mg Tablet (Sovereign Medical Ltd)	Beta blocker
32787	Visken 15mg tablets (AMCo)	Beta blocker
4588	Visken 5mg Tablet (Sovereign Medical Ltd)	Beta blocker
35695	Visken 5mg tablets (AMCo)	Beta blocker
32630	Vivacor 10mg tablets (Lexon (UK) Ltd)	Beta blocker
39846	Vivacor 5mg tablets (Lexon (UK) Ltd)	Beta blocker
25054	ADALAT	Calcium channel blocker
27910	ADALAT 5	Calcium channel blocker
25027	ADALAT RETARD 10	Calcium channel blocker
19015	ADIZEM CONTINUS 120 MG TAB	Calcium channel blocker
9211	ADIZEM-XL 180 MG CAP	Calcium channel blocker
2521	Adalat 10mg capsules (Bayer Plc)	Calcium channel blocker
67074	Adalat 10mg capsules (DE Pharmaceuticals)	Calcium channel blocker
662	Adalat 5mg capsules (Bayer Plc)	Calcium channel blocker
74001	Adalat LA 20 tablets (Dowelhurst Ltd)	Calcium channel blocker
58557	Adalat LA 20 tablets (Necessity Supplies Ltd)	Calcium channel blocker
57653	Adalat LA 20 tablets (Sigma Pharmaceuticals Plc)	Calcium channel blocker
541	Adalat LA 20mg tablets (Bayer Plc)	Calcium channel blocker
53500	Adalat LA 30 tablets (DE Pharmaceuticals)	Calcium channel blocker
71339	Adalat LA 30 tablets (Dowelhurst Ltd)	Calcium channel blocker
52017	Adalat LA 30 tablets (Mawdsley-Brooks & Company Ltd)	Calcium channel blocker
53278	Adalat LA 30 tablets (Necessity Supplies Ltd)	Calcium channel blocker
74061	Adalat LA 30 tablets (Waymade Healthcare Plc)	Calcium channel blocker
43753	Adalat LA 30mg tablets (Bayer Plc)	Calcium channel

		blocker
73992	Adalat LA 60 tablets (Dowelhurst Ltd)	Calcium channel blocker
56469	Adalat LA 60 tablets (Necessity Supplies Ltd)	Calcium channel blocker
51917	Adalat LA 60 tablets (Sigma Pharmaceuticals Plc)	Calcium channel blocker
57531	Adalat LA 60 tablets (Waymade Healthcare Plc)	Calcium channel blocker
43818	Adalat LA 60mg tablets (Bayer Plc)	Calcium channel blocker
1854	Adalat la 30mg Tablet (Bayer Plc)	Calcium channel blocker
4227	Adalat la 60mg Tablet (Bayer Plc)	Calcium channel blocker
2280	Adalat retard 10mg tablets (Bayer Plc)	Calcium channel blocker
2343	Adalat retard 20mg tablets (Bayer Plc)	Calcium channel blocker
66191	Adalat retard 20mg tablets (DE Pharmaceuticals)	Calcium channel blocker
53629	Adalat retard 20mg tablets (Lexon (UK) Ltd)	Calcium channel blocker
45685	Adanif XL 30mg tablets (Focus Pharmaceuticals Ltd)	Calcium channel blocker
46887	Adanif XL 60mg tablets (Focus Pharmaceuticals Ltd)	Calcium channel blocker
4239	Adipine MR 10 tablets (Chiesi Ltd)	Calcium channel blocker
3711	Adipine MR 20 tablets (Chiesi Ltd)	Calcium channel blocker
13139	Adipine XL 30mg tablets (Chiesi Ltd)	Calcium channel blocker
10246	Adipine XL 60mg tablets (Chiesi Ltd)	Calcium channel blocker
41979	Adipine la 30mg Modified-release tablet (Chiesi Ltd)	Calcium channel blocker
47217	Adipine la 60mg Modified-release tablet (Chiesi Ltd)	Calcium channel blocker
9374	Adizem 60mg Modified-release tablet (Napp Pharmaceuticals Ltd)	Calcium channel blocker
517	Adizem sr 120mg Modified-release capsule (Napp Pharmaceuticals Ltd)	Calcium channel blocker
4852	Adizem sr 120mg Modified-release tablet (Napp Pharmaceuticals Ltd)	Calcium channel blocker
2811	Adizem sr 180mg Modified-release capsule (Napp Pharmaceuticals Ltd)	Calcium channel blocker
3118	Adizem sr 90mg Modified-release capsule (Napp Pharmaceuticals Ltd)	Calcium channel blocker
8558	Adizem xl 120mg Capsule (Napp Pharmaceuticals Ltd)	Calcium channel blocker
9240	Adizem xl 180mg Capsule (Napp Pharmaceuticals Ltd)	Calcium channel

		blocker
793	Adizem xl 240mg Capsule (Napp Pharmaceuticals Ltd)	Calcium channel blocker
6309	Adizem xl 300mg Capsule (Napp Pharmaceuticals Ltd)	Calcium channel blocker
38818	Adizem-SR 120mg capsules (Napp Pharmaceuticals Ltd)	Calcium channel blocker
62207	Adizem-SR 120mg capsules (Waymade Healthcare Plc)	Calcium channel blocker
38964	Adizem-SR 120mg tablets (Napp Pharmaceuticals Ltd)	Calcium channel blocker
65504	Adizem-SR 180mg capsules (Lexon (UK) Ltd)	Calcium channel blocker
38831	Adizem-SR 180mg capsules (Napp Pharmaceuticals Ltd)	Calcium channel blocker
48870	Adizem-SR 90mg capsules (DE Pharmaceuticals)	Calcium channel blocker
38632	Adizem-SR 90mg capsules (Napp Pharmaceuticals Ltd)	Calcium channel blocker
65636	Adizem-XL 120mg capsules (Lexon (UK) Ltd)	Calcium channel blocker
38865	Adizem-XL 120mg capsules (Napp Pharmaceuticals Ltd)	Calcium channel blocker
65602	Adizem-XL 120mg capsules (Waymade Healthcare Plc)	Calcium channel blocker
52276	Adizem-XL 180mg capsules (DE Pharmaceuticals)	Calcium channel blocker
38855	Adizem-XL 180mg capsules (Napp Pharmaceuticals Ltd)	Calcium channel blocker
10267	Adizem-XL 200mg capsules (Napp Pharmaceuticals Ltd)	Calcium channel blocker
69277	Adizem-XL 240mg capsules (Lexon (UK) Ltd)	Calcium channel blocker
38882	Adizem-XL 240mg capsules (Napp Pharmaceuticals Ltd)	Calcium channel blocker
60620	Adizem-XL 240mg capsules (Waymade Healthcare Plc)	Calcium channel blocker
69108	Adizem-XL 300mg capsules (Lexon (UK) Ltd)	Calcium channel blocker
38634	Adizem-XL 300mg capsules (Napp Pharmaceuticals Ltd)	Calcium channel blocker
6856	Amlodipine 10mg tablets	Calcium channel blocker
34093	Amlodipine 10mg tablets (A A H Pharmaceuticals Ltd)	Calcium channel blocker
58580	Amlodipine 10mg tablets (APC Pharmaceuticals & Chemicals (Europe) Ltd)	Calcium channel blocker
56147	Amlodipine 10mg tablets (Accord Healthcare Ltd)	Calcium channel blocker
36202	Amlodipine 10mg tablets (Actavis UK Ltd)	Calcium channel blocker
54515	Amlodipine 10mg tablets (Alliance Healthcare (Distribution) Ltd)	Calcium channel

		blocker
54654	Amlodipine 10mg tablets (Almus Pharmaceuticals Ltd)	Calcium channel blocker
56334	Amlodipine 10mg tablets (Bristol Laboratories Ltd)	Calcium channel blocker
49636	Amlodipine 10mg tablets (DE Pharmaceuticals)	Calcium channel blocker
63515	Amlodipine 10mg tablets (Dr Reddy's Laboratories (UK) Ltd)	Calcium channel blocker
71939	Amlodipine 10mg tablets (Focus Pharmaceuticals Ltd)	Calcium channel blocker
68221	Amlodipine 10mg tablets (Kent Pharmaceuticals Ltd)	Calcium channel blocker
59001	Amlodipine 10mg tablets (Mylan)	Calcium channel blocker
60244	Amlodipine 10mg tablets (Phoenix Healthcare Distribution Ltd)	Calcium channel blocker
54696	Amlodipine 10mg tablets (Sandoz Ltd)	Calcium channel blocker
74820	Amlodipine 10mg tablets (Sigma Pharmaceuticals Plc)	Calcium channel blocker
74795	Amlodipine 10mg tablets (Somex Pharma)	Calcium channel blocker
59762	Amlodipine 10mg tablets (Teva UK Ltd)	Calcium channel blocker
66817	Amlodipine 10mg tablets (Wockhardt UK Ltd)	Calcium channel blocker
42210	Amlodipine 10mg tablets (Zentiva)	Calcium channel blocker
52440	Amlodipine 10mg/5ml oral solution	Calcium channel blocker
64623	Amlodipine 10mg/5ml oral solution sugar free	Calcium channel blocker
72430	Amlodipine 10mg/5ml oral solution sugar free (Alliance Healthcare (Distribution) Ltd)	Calcium channel blocker
45070	Amlodipine 10mg/5ml oral suspension	Calcium channel blocker
47002	Amlodipine 10mg/5ml sugar free Oral suspension	Calcium channel blocker
54983	Amlodipine 2.5mg/5ml oral suspension	Calcium channel blocker
61374	Amlodipine 4mg/5ml oral suspension	Calcium channel blocker
749	Amlodipine 5mg tablets	Calcium channel blocker
32595	Amlodipine 5mg tablets (A A H Pharmaceuticals Ltd)	Calcium channel blocker
70999	Amlodipine 5mg tablets (APC Pharmaceuticals & Chemicals (Europe) Ltd)	Calcium channel blocker
61422	Amlodipine 5mg tablets (Accord Healthcare Ltd)	Calcium channel blocker
53868	Amlodipine 5mg tablets (Actavis UK Ltd)	Calcium channel

		blocker
64441	Amlodipine 5mg tablets (Alliance Healthcare (Distribution) Ltd)	Calcium channel blocker
43880	Amlodipine 5mg tablets (Almus Pharmaceuticals Ltd)	Calcium channel blocker
74821	Amlodipine 5mg tablets (Apotex UK Ltd)	Calcium channel blocker
72199	Amlodipine 5mg tablets (Aurobindo Pharma Ltd)	Calcium channel blocker
54633	Amlodipine 5mg tablets (Bristol Laboratories Ltd)	Calcium channel blocker
65745	Amlodipine 5mg tablets (DE Pharmaceuticals)	Calcium channel blocker
71353	Amlodipine 5mg tablets (Dowelhurst Ltd)	Calcium channel blocker
39804	Amlodipine 5mg tablets (Dr Reddy's Laboratories (UK) Ltd)	Calcium channel blocker
64606	Amlodipine 5mg tablets (Focus Pharmaceuticals Ltd)	Calcium channel blocker
32917	Amlodipine 5mg tablets (IVAX Pharmaceuticals UK Ltd)	Calcium channel blocker
66430	Amlodipine 5mg tablets (Kent Pharmaceuticals Ltd)	Calcium channel blocker
72049	Amlodipine 5mg tablets (Mawdsley-Brooks & Company Ltd)	Calcium channel blocker
64327	Amlodipine 5mg tablets (Mylan)	Calcium channel blocker
66574	Amlodipine 5mg tablets (Phoenix Healthcare Distribution Ltd)	Calcium channel blocker
45279	Amlodipine 5mg tablets (Sandoz Ltd)	Calcium channel blocker
74108	Amlodipine 5mg tablets (Sigma Pharmaceuticals Plc)	Calcium channel blocker
64447	Amlodipine 5mg tablets (Somex Pharma)	Calcium channel blocker
39914	Amlodipine 5mg tablets (Teva UK Ltd)	Calcium channel blocker
64418	Amlodipine 5mg tablets (Waymade Healthcare Plc)	Calcium channel blocker
71344	Amlodipine 5mg tablets (Waymade Healthcare Plc)	Calcium channel blocker
43470	Amlodipine 5mg tablets (Wockhardt UK Ltd)	Calcium channel blocker
46724	Amlodipine 5mg/5ml oral solution	Calcium channel blocker
64166	Amlodipine 5mg/5ml oral solution sugar free	Calcium channel blocker
68311	Amlodipine 5mg/5ml oral solution sugar free (A A H Pharmaceuticals Ltd)	Calcium channel blocker
73612	Amlodipine 5mg/5ml oral solution sugar free (DE Pharmaceuticals)	Calcium channel blocker
74121	Amlodipine 5mg/5ml oral solution sugar free (Thame Laboratories Ltd)	Calcium channel

		blocker
16162	Amlodipine 5mg/5ml oral suspension	Calcium channel blocker
46233	Amlodipine Oral solution	Calcium channel blocker
71	Amlodipine besilate 10mg tablets	Calcium channel blocker
29	Amlodipine besilate 5mg tablets	Calcium channel blocker
6477	Amlodipine maleate 10mg tablets	Calcium channel blocker
729	Amlodipine maleate 5mg tablets	Calcium channel blocker
31761	Amlostin 10mg tablets (Discovery Pharmaceuticals)	Calcium channel blocker
17640	Amlostin 5mg tablets (Discovery Pharmaceuticals)	Calcium channel blocker
20878	Angiopine 10 capsules (Ashbourne Pharmaceuticals Ltd)	Calcium channel blocker
21872	Angiopine 5mg Capsule (Ashbourne Pharmaceuticals Ltd)	Calcium channel blocker
13672	Angiopine MR 10mg tablets (Ashbourne Pharmaceuticals Ltd)	Calcium channel blocker
5181	Angiopine MR 20mg tablets (Ashbourne Pharmaceuticals Ltd)	Calcium channel blocker
13699	Angiopine la 40mg Tablet (Ashbourne Pharmaceuticals Ltd)	Calcium channel blocker
13410	Angiozem 60mg modified-release tablets (Ashbourne Pharmaceuticals Ltd)	Calcium channel blocker
16850	Angiozem CR 120mg tablets (Ashbourne Pharmaceuticals Ltd)	Calcium channel blocker
12705	Angiozem CR 90mg tablets (Ashbourne Pharmaceuticals Ltd)	Calcium channel blocker
9410	Angitil SR 120 capsules (Ethypharm UK Ltd)	Calcium channel blocker
5054	Angitil SR 180 capsules (Ethypharm UK Ltd)	Calcium channel blocker
11223	Angitil SR 90 capsules (Ethypharm UK Ltd)	Calcium channel blocker
13033	Angitil XL 240 capsules (Ethypharm UK Ltd)	Calcium channel blocker
15288	Angitil XL 300 capsules (Ethypharm UK Ltd)	Calcium channel blocker
28844	Berkatens 120mg Tablet (Berk Pharmaceuticals Ltd)	Calcium channel blocker
26252	Berkatens 160mg Tablet (Berk Pharmaceuticals Ltd)	Calcium channel blocker
23872	Berkatens 40mg Tablet (Berk Pharmaceuticals Ltd)	Calcium channel blocker
25059	Berkatens 80mg Tablet (Berk Pharmaceuticals Ltd)	Calcium channel blocker
41489	Bi-Carzem SR 120mg capsules (Tillomed Laboratories Ltd)	Calcium channel

		blocker
39171	Bi-Carzem SR 60mg capsules (Tillomed Laboratories Ltd)	Calcium channel blocker
39298	Bi-Carzem SR 90mg capsules (Tillomed Laboratories Ltd)	Calcium channel blocker
47724	Bi-Carzem XL 240mg capsules (Tillomed Laboratories Ltd)	Calcium channel blocker
28949	Bi-carzem sr 120mg Modified-release capsule (Tillomed Laboratories Ltd)	Calcium channel blocker
20642	Bi-carzem sr 60mg Modified-release capsule (Tillomed Laboratories Ltd)	Calcium channel blocker
23233	Bi-carzem sr 90mg Modified-release capsule (Tillomed Laboratories Ltd)	Calcium channel blocker
31489	Bi-carzem xl 240mg Capsule (Tillomed Laboratories Ltd)	Calcium channel blocker
44887	Bi-carzem xl 300mg Capsule (Tillomed Laboratories Ltd)	Calcium channel blocker
22619	Britiazim 60mg Modified-release tablet (Thames Laboratories Ltd)	Calcium channel blocker
21665	CORDILOX	Calcium channel blocker
26337	Cabren 10mg modified-release tablets (Teva UK Ltd)	Calcium channel blocker
30915	Cabren 2.5mg modified-release tablets (Teva UK Ltd)	Calcium channel blocker
30991	Cabren 5mg modified-release tablets (Teva UK Ltd)	Calcium channel blocker
22019	Calanif 10mg Capsule (Berk Pharmaceuticals Ltd)	Calcium channel blocker
26265	Calanif 5mg Capsule (Berk Pharmaceuticals Ltd)	Calcium channel blocker
29676	Calazem 60mg Modified-release tablet (Berk Pharmaceuticals Ltd)	Calcium channel blocker
14861	Calchan MR 10 tablets (Ranbaxy (UK) Ltd)	Calcium channel blocker
11769	Calchan MR 20 tablets (Ranbaxy (UK) Ltd)	Calcium channel blocker
18975	Calcicard 60mg Tablet (3M Health Care Ltd)	Calcium channel blocker
11973	Calcicard CR 120mg tablets (Teva UK Ltd)	Calcium channel blocker
9723	Calcicard CR 90mg tablets (Teva UK Ltd)	Calcium channel blocker
22142	Calcilat 10mg Capsule (Eastern Pharmaceuticals Ltd)	Calcium channel blocker
11943	Cardene 20mg capsules (Astellas Pharma Ltd)	Calcium channel blocker
7562	Cardene 30mg capsules (Astellas Pharma Ltd)	Calcium channel blocker
3302	Cardene SR 30mg capsules (Astellas Pharma Ltd)	Calcium channel blocker
12875	Cardene SR 45mg capsules (Astellas Pharma Ltd)	Calcium channel

		blocker
17325	Cardilate MR 10mg tablets (Teva UK Ltd)	Calcium channel blocker
20257	Cardilate MR 20mg tablets (IVAX Pharmaceuticals UK Ltd)	Calcium channel blocker
24366	Cardioplen XL 10mg tablets (Chiesi Ltd)	Calcium channel blocker
35592	Cardioplen XL 2.5mg tablets (Chiesi Ltd)	Calcium channel blocker
24365	Cardioplen XL 5mg tablets (Chiesi Ltd)	Calcium channel blocker
19013	Clinium 120mg Tablet (LEO Pharma)	Calcium channel blocker
2746	Coracten SR 10mg capsules (UCB Pharma Ltd)	Calcium channel blocker
4856	Coracten SR 20mg capsules (UCB Pharma Ltd)	Calcium channel blocker
3712	Coracten XL 30mg capsules (UCB Pharma Ltd)	Calcium channel blocker
4939	Coracten XL 60mg capsules (UCB Pharma Ltd)	Calcium channel blocker
1748	Cordilox 120mg tablets (IVAX Pharmaceuticals UK Ltd)	Calcium channel blocker
12104	Cordilox 160mg tablets (IVAX Pharmaceuticals UK Ltd)	Calcium channel blocker
19325	Cordilox 2.5mg/ml Injection (IVAX Pharmaceuticals UK Ltd)	Calcium channel blocker
8884	Cordilox 40mg tablets (IVAX Pharmaceuticals UK Ltd)	Calcium channel blocker
16677	Cordilox 80mg tablets (IVAX Pharmaceuticals UK Ltd)	Calcium channel blocker
13965	Cordilox MR 240mg tablets (Teva UK Ltd)	Calcium channel blocker
30473	Coroday MR 20mg tablets (Mylan)	Calcium channel blocker
15659	DILTIAZEM HCL S/R 180 CAP	Calcium channel blocker
9094	DILTIAZEM HCl SR 300 MG CAP	Calcium channel blocker
8024	DILTIAZEM HCl XL 300 MG CAP	Calcium channel blocker
30491	DILTIAZEM HYDROCHLORIDE	Calcium channel blocker
25777	Dilcardia SR 120mg capsules (Mylan)	Calcium channel blocker
21145	Dilcardia SR 60mg capsules (Mylan)	Calcium channel blocker
18379	Dilcardia SR 90mg capsules (Mylan)	Calcium channel blocker
32658	Dilcardia xl 120mg Modified-release capsule (Generics (UK) Ltd)	Calcium channel blocker
15221	Dilcardia xl 180mg Modified-release capsule (Generics (UK) Ltd)	Calcium channel

		blocker
26460	Dilcardia xl 240mg Modified-release capsule (Generics (UK) Ltd)	Calcium channel blocker
30197	Diltiazem 120mg modified-release capsules	Calcium channel blocker
48288	Diltiazem 120mg modified-release capsules (A A H Pharmaceuticals Ltd)	Calcium channel blocker
71413	Diltiazem 120mg modified-release capsules (A A H Pharmaceuticals Ltd)	Calcium channel blocker
62912	Diltiazem 120mg modified-release capsules (AM Distributions (Yorkshire) Ltd)	Calcium channel blocker
49289	Diltiazem 120mg modified-release capsules (Alliance Healthcare (Distribution) Ltd)	Calcium channel blocker
57208	Diltiazem 120mg modified-release capsules (Cubic Pharmaceuticals Ltd)	Calcium channel blocker
74915	Diltiazem 120mg modified-release capsules (DE Pharmaceuticals)	Calcium channel blocker
68429	Diltiazem 120mg modified-release capsules (Ethigen Ltd)	Calcium channel blocker
61532	Diltiazem 120mg modified-release capsules (Sigma Pharmaceuticals Plc)	Calcium channel blocker
219	Diltiazem 120mg modified-release tablets	Calcium channel blocker
43430	Diltiazem 120mg modified-release tablets (A A H Pharmaceuticals Ltd)	Calcium channel blocker
49001	Diltiazem 120mg modified-release tablets (Alliance Healthcare (Distribution) Ltd)	Calcium channel blocker
61010	Diltiazem 120mg modified-release tablets (Cubic Pharmaceuticals Ltd)	Calcium channel blocker
62064	Diltiazem 120mg modified-release tablets (Mawdsley-Brooks & Company Ltd)	Calcium channel blocker
1995	Diltiazem 12hr 120mg modified-release capsules	Calcium channel blocker
3061	Diltiazem 12hr 180mg modified-release capsules	Calcium channel blocker
30242	Diltiazem 180mg modified-release capsules	Calcium channel blocker
72091	Diltiazem 180mg modified-release capsules (DE Pharmaceuticals)	Calcium channel blocker
70306	Diltiazem 180mg modified-release capsules (Mawdsley-Brooks & Company Ltd)	Calcium channel blocker
4635	Diltiazem 200mg modified-release capsules	Calcium channel blocker
2663	Diltiazem 240mg modified-release capsules	Calcium channel blocker
4808	Diltiazem 240mg modified-release capsules	Calcium channel blocker
66701	Diltiazem 240mg modified-release capsules (DE Pharmaceuticals)	Calcium channel blocker
74689	Diltiazem 240mg modified-release capsules (Ennogen Pharma Ltd)	Calcium channel blocker
66850	Diltiazem 240mg modified-release capsules (Icarus Pharmaceuticals Ltd)	Calcium channel

		blocker
9708	Diltiazem 24hr 120mg modified-release capsules	Calcium channel blocker
4923	Diltiazem 24hr 180mg modified-release capsules	Calcium channel blocker
5326	Diltiazem 24hr 300mg modified-release capsules	Calcium channel blocker
5348	Diltiazem 300mg modified-release capsules	Calcium channel blocker
72488	Diltiazem 300mg modified-release capsules (A A H Pharmaceuticals Ltd)	Calcium channel blocker
72839	Diltiazem 300mg modified-release capsules (DE Pharmaceuticals)	Calcium channel blocker
67344	Diltiazem 300mg modified-release capsules (Ennogen Pharma Ltd)	Calcium channel blocker
71969	Diltiazem 300mg modified-release capsules (Sigma Pharmaceuticals Plc)	Calcium channel blocker
13926	Diltiazem 360mg modified-release capsules	Calcium channel blocker
636	Diltiazem 60mg modified-release capsules	Calcium channel blocker
2453	Diltiazem 60mg modified-release capsules	Calcium channel blocker
18404	Diltiazem 60mg modified-release capsules (A A H Pharmaceuticals Ltd)	Calcium channel blocker
48272	Diltiazem 60mg modified-release capsules (Alliance Healthcare (Distribution) Ltd)	Calcium channel blocker
69028	Diltiazem 60mg modified-release capsules (DE Pharmaceuticals)	Calcium channel blocker
61245	Diltiazem 60mg modified-release capsules (Sigma Pharmaceuticals Plc)	Calcium channel blocker
1836	Diltiazem 60mg modified-release tablets	Calcium channel blocker
21763	Diltiazem 60mg modified-release tablets (A A H Pharmaceuticals Ltd)	Calcium channel blocker
46937	Diltiazem 60mg modified-release tablets (Actavis UK Ltd)	Calcium channel blocker
68054	Diltiazem 60mg modified-release tablets (Alliance Healthcare (Distribution) Ltd)	Calcium channel blocker
69116	Diltiazem 60mg modified-release tablets (DE Pharmaceuticals)	Calcium channel blocker
41635	Diltiazem 60mg modified-release tablets (IVAX Pharmaceuticals UK Ltd)	Calcium channel blocker
32870	Diltiazem 60mg modified-release tablets (Sterwin Medicines)	Calcium channel blocker
21778	Diltiazem 60mg modified-release tablets (Teva UK Ltd)	Calcium channel blocker
1538	Diltiazem 60mg tablets	Calcium channel blocker
55257	Diltiazem 60mg/5ml oral solution	Calcium channel blocker
71702	Diltiazem 60mg/5ml oral solution (Special Order)	Calcium channel

		blocker
11922	Diltiazem 60mg/5ml oral suspension	Calcium channel blocker
1686	Diltiazem 90mg modified-release capsules	Calcium channel blocker
48282	Diltiazem 90mg modified-release capsules (A A H Pharmaceuticals Ltd)	Calcium channel blocker
48457	Diltiazem 90mg modified-release capsules (Alliance Healthcare (Distribution) Ltd)	Calcium channel blocker
56758	Diltiazem 90mg modified-release capsules (Cubic Pharmaceuticals Ltd)	Calcium channel blocker
4732	Diltiazem 90mg modified-release tablets	Calcium channel blocker
27136	Diltiazem 90mg modified-release tablets (A A H Pharmaceuticals Ltd)	Calcium channel blocker
49390	Diltiazem 90mg modified-release tablets (Alliance Healthcare (Distribution) Ltd)	Calcium channel blocker
62065	Diltiazem 90mg modified-release tablets (Colorama Pharmaceuticals Ltd)	Calcium channel blocker
57859	Diltiazem 90mg modified-release tablets (Cubic Pharmaceuticals Ltd)	Calcium channel blocker
32089	Diltiazem HCl 120mg Modified-release capsule (Hillcross Pharmaceuticals Ltd)	Calcium channel blocker
31676	Diltiazem HCl 120mg Modified-release tablet (Actavis UK Ltd)	Calcium channel blocker
34824	Diltiazem HCl 120mg Modified-release tablet (IVAX Pharmaceuticals UK Ltd)	Calcium channel blocker
42804	Diltiazem HCl 180mg Capsule (PLIVA Pharma Ltd)	Calcium channel blocker
18403	Diltiazem HCl 180mg Modified-release capsule (Hillcross Pharmaceuticals Ltd)	Calcium channel blocker
45759	Diltiazem HCl 240mg Capsule (PLIVA Pharma Ltd)	Calcium channel blocker
27685	Diltiazem HCl 300mg Capsule (PLIVA Pharma Ltd)	Calcium channel blocker
34581	Diltiazem HCl 60mg Modified-release tablet (Kent Pharmaceuticals Ltd)	Calcium channel blocker
38066	Diltiazem HCl 60mg Modified-release tablet (Lagap)	Calcium channel blocker
32262	Diltiazem HCl 60mg Tablet (C P Pharmaceuticals Ltd)	Calcium channel blocker
21773	Diltiazem HCl 60mg Tablet (Generics (UK) Ltd)	Calcium channel blocker
34377	Diltiazem HCl 90mg Modified-release capsule (Hillcross Pharmaceuticals Ltd)	Calcium channel blocker
12639	Diltiazem HCl 90mg Modified-release tablet (Actavis UK Ltd)	Calcium channel blocker
34475	Diltiazem HCl 90mg Modified-release tablet (IVAX Pharmaceuticals UK Ltd)	Calcium channel blocker
42731	Diltiazem sr 120mg Capsule (Hillcross Pharmaceuticals Ltd)	Calcium channel blocker
47415	Diltiazem sr 60mg Capsule (Hillcross Pharmaceuticals Ltd)	Calcium channel

		blocker
27135	Diltiazem sr 90mg Capsule (Hillcross Pharmaceuticals Ltd)	Calcium channel blocker
42819	Diltiazem xl 240mg Capsule (Hillcross Pharmaceuticals Ltd)	Calcium channel blocker
16038	Dilzem SR 120 capsules (Teva UK Ltd)	Calcium channel blocker
11770	Dilzem SR 60 capsules (Teva UK Ltd)	Calcium channel blocker
13302	Dilzem SR 90 capsules (Teva UK Ltd)	Calcium channel blocker
67317	Dilzem XL 120 capsules (Mawdsley-Brooks & Company Ltd)	Calcium channel blocker
13240	Dilzem XL 120 capsules (Teva UK Ltd)	Calcium channel blocker
66635	Dilzem XL 180 capsules (DE Pharmaceuticals)	Calcium channel blocker
59098	Dilzem XL 180 capsules (Lexon (UK) Ltd)	Calcium channel blocker
60415	Dilzem XL 180 capsules (Sigma Pharmaceuticals Plc)	Calcium channel blocker
13075	Dilzem XL 180 capsules (Teva UK Ltd)	Calcium channel blocker
59863	Dilzem XL 240 capsules (Lexon (UK) Ltd)	Calcium channel blocker
74053	Dilzem XL 240 capsules (Sigma Pharmaceuticals Plc)	Calcium channel blocker
13127	Dilzem XL 240 capsules (Teva UK Ltd)	Calcium channel blocker
71342	Dilzem XL 240 capsules (Waymade Healthcare Plc)	Calcium channel blocker
5194	Dilzem sr 120mg Capsule (Elan Pharma)	Calcium channel blocker
5513	Dilzem sr 60mg Capsule (Elan Pharma)	Calcium channel blocker
4308	Dilzem sr 90mg Capsule (Elan Pharma)	Calcium channel blocker
3370	Dilzem xl mr 120mg Modified-release capsule (Elan Pharma)	Calcium channel blocker
3676	Dilzem xl mr 180mg Modified-release capsule (Elan Pharma)	Calcium channel blocker
2686	Dilzem xl mr 240mg Modified-release capsule (Elan Pharma)	Calcium channel blocker
18852	Disogram SR 120mg capsules (Ranbaxy (UK) Ltd)	Calcium channel blocker
18874	Disogram SR 180mg capsules (Ranbaxy (UK) Ltd)	Calcium channel blocker
19426	Disogram SR 240mg capsules (Ranbaxy (UK) Ltd)	Calcium channel blocker
19440	Disogram SR 300mg capsules (Ranbaxy (UK) Ltd)	Calcium channel blocker
18834	Disogram SR 60mg capsules (Ranbaxy (UK) Ltd)	Calcium channel

		blocker
18830	Disogram SR 90mg capsules (Ranbaxy (UK) Ltd)	Calcium channel blocker
30462	Ethimil MR 240mg tablets (Genus Pharmaceuticals Ltd)	Calcium channel blocker
20459	Felendil xl 10mg Modified-release tablet (Ratiopharm UK Ltd)	Calcium channel blocker
29145	Felendil xl 2.5mg Modified-release tablet (Ratiopharm UK Ltd)	Calcium channel blocker
10153	Felendil xl 5mg Modified-release tablet (Ratiopharm UK Ltd)	Calcium channel blocker
32922	Felodipine 10mg Modified-release tablet (Sandoz Ltd)	Calcium channel blocker
568	Felodipine 10mg modified-release tablets	Calcium channel blocker
33091	Felodipine 10mg modified-release tablets (A A H Pharmaceuticals Ltd)	Calcium channel blocker
64760	Felodipine 10mg modified-release tablets (Phoenix Healthcare Distribution Ltd)	Calcium channel blocker
64917	Felodipine 10mg modified-release tablets (Waymade Healthcare Plc)	Calcium channel blocker
491	Felodipine 2.5mg modified-release tablets	Calcium channel blocker
64474	Felodipine 2.5mg modified-release tablets (A A H Pharmaceuticals Ltd)	Calcium channel blocker
66910	Felodipine 2.5mg modified-release tablets (DE Pharmaceuticals)	Calcium channel blocker
60884	Felodipine 2.5mg modified-release tablets (Phoenix Healthcare Distribution Ltd)	Calcium channel blocker
64719	Felodipine 2.5mg modified-release tablets (Sigma Pharmaceuticals Plc)	Calcium channel blocker
60569	Felodipine 2.5mg modified-release tablets (Waymade Healthcare Plc)	Calcium channel blocker
69206	Felodipine 2.5mg/5ml oral solution	Calcium channel blocker
48009	Felodipine 5mg Modified-release tablet (Sandoz Ltd)	Calcium channel blocker
501	Felodipine 5mg modified-release tablets	Calcium channel blocker
43512	Felodipine 5mg modified-release tablets (A A H Pharmaceuticals Ltd)	Calcium channel blocker
68828	Felodipine 5mg modified-release tablets (DE Pharmaceuticals)	Calcium channel blocker
66095	Felodipine 5mg modified-release tablets (Mawdsley-Brooks & Company Ltd)	Calcium channel blocker
73715	Felodipine 5mg modified-release tablets (Phoenix Healthcare Distribution Ltd)	Calcium channel blocker
68181	Felodipine 5mg modified-release tablets (Sigma Pharmaceuticals Plc)	Calcium channel blocker
72221	Felodipine 5mg modified-release tablets (Waymade Healthcare Plc)	Calcium channel blocker
72181	Felodipine 5mg/5ml oral solution	Calcium channel

		blocker
68499	Felodipine sr 10mg Tablet (Approved Prescription Services Ltd)	Calcium channel blocker
44859	Felodipine sr 5mg Tablet (Approved Prescription Services Ltd)	Calcium channel blocker
30557	Felogen XL 10mg tablets (Mylan)	Calcium channel blocker
25572	Felogen XL 5mg tablets (Mylan)	Calcium channel blocker
17566	Felotens XL 10mg tablets (Thornton & Ross Ltd)	Calcium channel blocker
37897	Felotens XL 2.5mg tablets (Thornton & Ross Ltd)	Calcium channel blocker
17557	Felotens XL 5mg tablets (Thornton & Ross Ltd)	Calcium channel blocker
65349	Folpik XL 10mg tablets (Teva UK Ltd)	Calcium channel blocker
63331	Folpik XL 2.5mg tablets (Teva UK Ltd)	Calcium channel blocker
55306	Folpik XL 5mg tablets (Teva UK Ltd)	Calcium channel blocker
5277	Fortipine LA 40 tablets (AMCo)	Calcium channel blocker
15715	Genalat retard 20mg Modified-release tablet (Wyeth Pharmaceuticals)	Calcium channel blocker
67293	Half Securon SR 120mg tablets (Mawdsley-Brooks & Company Ltd)	Calcium channel blocker
3343	Half Securon SR 120mg tablets (Mylan)	Calcium channel blocker
47530	Horizem SR 60mg capsules (Horizon lifecare)	Calcium channel blocker
21216	Hypolar Retard 10mg tablets (Sandoz Ltd)	Calcium channel blocker
9485	Hypolar Retard 20 tablets (Sandoz Ltd)	Calcium channel blocker
23736	Hypolar XL 30 tablets (Sandoz Ltd)	Calcium channel blocker
8310	Isradipine 2.5mg tablets	Calcium channel blocker
67662	Istin 10mg tablets (DE Pharmaceuticals)	Calcium channel blocker
5914	Istin 10mg tablets (Pfizer Ltd)	Calcium channel blocker
3917	Istin 5mg tablets (Pfizer Ltd)	Calcium channel blocker
38434	Keloc SR 10mg tablets (Teva UK Ltd)	Calcium channel blocker
35696	Kenzem SR 120mg capsules (Kent Pharmaceuticals Ltd)	Calcium channel blocker
37774	Kenzem SR 60mg capsules (Kent Pharmaceuticals Ltd)	Calcium channel blocker
27401	Kenzem SR 90mg capsules (Kent Pharmaceuticals Ltd)	Calcium channel

		blocker
5158	Lacidipine 2mg tablets	Calcium channel blocker
70990	Lacidipine 2mg tablets (A A H Pharmaceuticals Ltd)	Calcium channel blocker
60699	Lacidipine 2mg tablets (Sigma Pharmaceuticals Plc)	Calcium channel blocker
3221	Lacidipine 4mg tablets	Calcium channel blocker
57680	Lacidipine 4mg tablets (A A H Pharmaceuticals Ltd)	Calcium channel blocker
56994	Lacidipine 4mg tablets (Teva UK Ltd)	Calcium channel blocker
5593	Lercanidipine 10mg tablets	Calcium channel blocker
69239	Lercanidipine 10mg tablets (A A H Pharmaceuticals Ltd)	Calcium channel blocker
64227	Lercanidipine 10mg tablets (Actavis UK Ltd)	Calcium channel blocker
57444	Lercanidipine 10mg tablets (Aptil Pharma Ltd)	Calcium channel blocker
71018	Lercanidipine 10mg tablets (Arrow Generics Ltd)	Calcium channel blocker
61611	Lercanidipine 10mg tablets (DE Pharmaceuticals)	Calcium channel blocker
47331	Lercanidipine 10mg tablets (Mylan)	Calcium channel blocker
70827	Lercanidipine 10mg tablets (Teva UK Ltd)	Calcium channel blocker
13243	Lercanidipine 20mg tablets	Calcium channel blocker
63917	Lercanidipine 20mg tablets (A A H Pharmaceuticals Ltd)	Calcium channel blocker
59233	Lercanidipine 20mg tablets (Actavis UK Ltd)	Calcium channel blocker
70732	Lercanidipine 20mg tablets (Alliance Healthcare (Distribution) Ltd)	Calcium channel blocker
71030	Lercanidipine 20mg tablets (Arrow Generics Ltd)	Calcium channel blocker
56767	Lercanidipine 20mg tablets (Mylan)	Calcium channel blocker
65659	Lercanidipine 20mg tablets (Teva UK Ltd)	Calcium channel blocker
64424	Lercanidipine 20mg tablets (Zentiva)	Calcium channel blocker
30758	Lidoflazine 120mg Tablet	Calcium channel blocker
22241	Mibefradil 100mg Tablet	Calcium channel blocker
15652	Mibefradil 50mg Tablet	Calcium channel blocker
11966	Motens 2mg tablets (GlaxoSmithKline UK Ltd)	Calcium channel

		blocker
9670	Motens 4mg tablets (GlaxoSmithKline UK Ltd)	Calcium channel blocker
25055	NIFEDIPINE	Calcium channel blocker
25026	NIFEDIPINE RETARD	Calcium channel blocker
25044	NIFEDIPINE RETARD	Calcium channel blocker
7823	NIFEDIPINE TAB 5 mg	Calcium channel blocker
29044	Neofel XL 10mg tablets (Kent Pharmaceuticals Ltd)	Calcium channel blocker
55740	Neofel XL 2.5mg tablets (Actavis UK Ltd)	Calcium channel blocker
58339	Neofel XL 2.5mg tablets (Almus Pharmaceuticals Ltd)	Calcium channel blocker
39357	Neofel XL 2.5mg tablets (Kent Pharmaceuticals Ltd)	Calcium channel blocker
28721	Neofel XL 5mg tablets (Kent Pharmaceuticals Ltd)	Calcium channel blocker
37530	Neozipine XL 30mg tablets (Kent Pharmaceuticals Ltd)	Calcium channel blocker
35646	Neozipine XL 60mg tablets (Kent Pharmaceuticals Ltd)	Calcium channel blocker
2926	Nicardipine 20mg capsules	Calcium channel blocker
73968	Nicardipine 20mg capsules (Teva UK Ltd)	Calcium channel blocker
73646	Nicardipine 20mg capsules (Tillomed Laboratories Ltd)	Calcium channel blocker
8201	Nicardipine 30mg capsules	Calcium channel blocker
45292	Nicardipine 30mg capsules (A A H Pharmaceuticals Ltd)	Calcium channel blocker
5477	Nicardipine 30mg modified-release capsules	Calcium channel blocker
9386	Nicardipine 45mg modified-release capsules	Calcium channel blocker
72321	Nidef 30mg modified-release tablets (Morningside Healthcare Ltd)	Calcium channel blocker
71653	Nidef 60mg modified-release tablets (Morningside Healthcare Ltd)	Calcium channel blocker
37726	Nifedipine 100mg/5ml oral suspension	Calcium channel blocker
34247	Nifedipine 10mg Capsule (Berk Pharmaceuticals Ltd)	Calcium channel blocker
74648	Nifedipine 10mg Capsule (C P Pharmaceuticals Ltd)	Calcium channel blocker
34187	Nifedipine 10mg Modified-release tablet (Generics (UK) Ltd)	Calcium channel blocker
47027	Nifedipine 10mg Modified-release tablet (Kent Pharmaceuticals Ltd)	Calcium channel

		blocker
452	Nifedipine 10mg capsules	Calcium channel blocker
43511	Nifedipine 10mg capsules (A A H Pharmaceuticals Ltd)	Calcium channel blocker
43515	Nifedipine 10mg capsules (Actavis UK Ltd)	Calcium channel blocker
46445	Nifedipine 10mg capsules (IVAX Pharmaceuticals UK Ltd)	Calcium channel blocker
63041	Nifedipine 10mg capsules (Mylan)	Calcium channel blocker
55455	Nifedipine 10mg capsules (Strides Shasun (UK) Ltd)	Calcium channel blocker
42912	Nifedipine 10mg capsules (Teva UK Ltd)	Calcium channel blocker
2605	Nifedipine 10mg modified-release capsules	Calcium channel blocker
410	Nifedipine 10mg modified-release tablets	Calcium channel blocker
28688	Nifedipine 10mg modified-release tablets (A A H Pharmaceuticals Ltd)	Calcium channel blocker
63246	Nifedipine 10mg modified-release tablets (AM Distributions (Yorkshire) Ltd)	Calcium channel blocker
49762	Nifedipine 10mg modified-release tablets (Alliance Healthcare (Distribution) Ltd)	Calcium channel blocker
58990	Nifedipine 10mg modified-release tablets (Cubic Pharmaceuticals Ltd)	Calcium channel blocker
71760	Nifedipine 10mg modified-release tablets (Ethigen Ltd)	Calcium channel blocker
60856	Nifedipine 10mg modified-release tablets (Sigma Pharmaceuticals Plc)	Calcium channel blocker
26774	Nifedipine 10mg/5ml Oral suspension	Calcium channel blocker
53357	Nifedipine 10mg/5ml oral suspension	Calcium channel blocker
1262	Nifedipine 12 20mg Modified-release tablet	Calcium channel blocker
71601	Nifedipine 2.5mg/5ml oral suspension	Calcium channel blocker
40074	Nifedipine 20mg Capsule	Calcium channel blocker
55824	Nifedipine 20mg Modified-release tablet (Berk Pharmaceuticals Ltd)	Calcium channel blocker
66236	Nifedipine 20mg Modified-release tablet (Kent Pharmaceuticals Ltd)	Calcium channel blocker
737	Nifedipine 20mg modified-release capsules	Calcium channel blocker
37025	Nifedipine 20mg modified-release tablets	Calcium channel blocker
25919	Nifedipine 20mg modified-release tablets (A A H Pharmaceuticals Ltd)	Calcium channel blocker
49338	Nifedipine 20mg modified-release tablets (Alliance Healthcare (Distribution)	Calcium channel

	Ltd)	blocker
59163	Nifedipine 20mg modified-release tablets (Cubic Pharmaceuticals Ltd)	Calcium channel blocker
74330	Nifedipine 20mg modified-release tablets (Ennogen Healthcare Ltd)	Calcium channel blocker
47529	Nifedipine 20mg/ml oral drops	Calcium channel blocker
8213	Nifedipine 24 20mg Modified-release tablet	Calcium channel blocker
1449	Nifedipine 24 30mg Modified-release tablet	Calcium channel blocker
69202	Nifedipine 2mg/5ml oral suspension	Calcium channel blocker
5162	Nifedipine 30mg modified-release capsules	Calcium channel blocker
30199	Nifedipine 30mg modified-release tablets	Calcium channel blocker
47614	Nifedipine 30mg modified-release tablets (A A H Pharmaceuticals Ltd)	Calcium channel blocker
72253	Nifedipine 30mg modified-release tablets (Sigma Pharmaceuticals Plc)	Calcium channel blocker
69668	Nifedipine 30mg/5ml oral suspension	Calcium channel blocker
9269	Nifedipine 40mg modified-release tablets	Calcium channel blocker
269	Nifedipine 5mg capsules	Calcium channel blocker
34522	Nifedipine 5mg capsules (A A H Pharmaceuticals Ltd)	Calcium channel blocker
34607	Nifedipine 5mg capsules (IVAX Pharmaceuticals UK Ltd)	Calcium channel blocker
34975	Nifedipine 5mg capsules (Teva UK Ltd)	Calcium channel blocker
53990	Nifedipine 5mg/5ml oral suspension	Calcium channel blocker
34115	Nifedipine 60mg Modified-release tablet	Calcium channel blocker
9750	Nifedipine 60mg modified-release capsules	Calcium channel blocker
3930	Nifedipine 60mg modified-release tablets	Calcium channel blocker
47707	Nifedipine Oral solution	Calcium channel blocker
43410	Nifedipine extra 60mg Modified-release tablet	Calcium channel blocker
34146	Nifedipine mr 10mg Modified-release tablet (IVAX Pharmaceuticals UK Ltd)	Calcium channel blocker
34101	Nifedipine mr 20mg Modified-release tablet (IVAX Pharmaceuticals UK Ltd)	Calcium channel blocker
38107	Nifedipine sr 30mg Tablet (Hillcross Pharmaceuticals Ltd)	Calcium channel blocker
47285	Nifedipine xl 60mg Tablet (Hillcross Pharmaceuticals Ltd)	Calcium channel

		blocker
11512	Nifedipress MR 10 tablets (Dexcel-Pharma Ltd)	Calcium channel blocker
16073	Nifedipress MR 10 tablets (Teva UK Ltd)	Calcium channel blocker
21886	Nifedipress MR 20 tablets (Actavis UK Ltd)	Calcium channel blocker
10136	Nifedipress MR 20 tablets (Dexcel-Pharma Ltd)	Calcium channel blocker
20591	Nifedipress MR 20 tablets (Teva UK Ltd)	Calcium channel blocker
21245	Nifedipress mr 10mg Modified-release tablet (Actavis UK Ltd)	Calcium channel blocker
10135	Nifedipress mr 10mg Modified-release tablet (Sandoz Ltd)	Calcium channel blocker
17448	Nifedipress mr 10mg Modified-release tablet (Sterwin Medicines)	Calcium channel blocker
20311	Nifedipress mr 20mg Modified-release tablet (Generics (UK) Ltd)	Calcium channel blocker
17338	Nifedotard 20 mr 20mg Modified-release tablet (Galen Ltd)	Calcium channel blocker
12606	Nifelease 20mg Modified-release tablet (Eastern Pharmaceuticals Ltd)	Calcium channel blocker
1300	Nifensar xl 20mg Modified-release tablet (Rhone-Poulenc Rorer Ltd)	Calcium channel blocker
25132	Nifopress MR 20mg tablets (Teva UK Ltd)	Calcium channel blocker
7541	Nifopress Retard 20mg tablets (AMCo)	Calcium channel blocker
71529	Nimodipine 10mg/50ml solution for infusion vials	Calcium channel blocker
11547	Nimodipine 30mg tablets	Calcium channel blocker
22217	Nimodrel 10mg modified-release tablet (Opus Pharmaceuticals Ltd)	Calcium channel blocker
24228	Nimodrel 20mg modified-release tablet (Opus Pharmaceuticals Ltd)	Calcium channel blocker
33025	Nimodrel XL 30mg tablets (Zurich Pharmaceuticals)	Calcium channel blocker
47887	Nimodrel XL 60mg tablets (Zurich Pharmaceuticals)	Calcium channel blocker
10595	Nimotop 30mg tablets (Bayer Plc)	Calcium channel blocker
23805	Nisoldipine 10mg modified-release tablets	Calcium channel blocker
18038	Nisoldipine 20mg modified-release tablets	Calcium channel blocker
23823	Nisoldipine 30mg modified-release tablets	Calcium channel blocker
17342	Nivaten retard 10mg Modified-release tablet (Actavis UK Ltd)	Calcium channel blocker
25646	Nivaten retard 20mg Modified-release tablet (Actavis UK Ltd)	Calcium channel

Carr MJ, et al. BMJ Qual Saf 2022; 31:503–514. doi: 10.1136/bmjqs-2021-013613

		blocker
21918	Optil 60mg modified-release tablets (Opus Pharmaceuticals Ltd)	Calcium channel blocker
26267	Optil sr 120mg Modified-release capsule (Opus Pharmaceuticals Ltd)	Calcium channel blocker
26269	Optil sr 180mg Modified-release capsule (Opus Pharmaceuticals Ltd)	Calcium channel blocker
23733	Optil sr 90mg Modified-release capsule (Opus Pharmaceuticals Ltd)	Calcium channel blocker
26309	Optil xl 240mg Modified-release capsule (Opus Pharmaceuticals Ltd)	Calcium channel blocker
26270	Optil xl 300mg Modified-release capsule (Opus Pharmaceuticals Ltd)	Calcium channel blocker
36620	Parmid XL 10mg tablets (Sandoz Ltd)	Calcium channel blocker
60652	Parmid XL 2.5mg tablets (Sandoz Ltd)	Calcium channel blocker
33932	Parmid XL 5mg tablets (Sandoz Ltd)	Calcium channel blocker
43394	Pinefeld XL 10mg tablets (Tillomed Laboratories Ltd)	Calcium channel blocker
7280	Plendil 10mg modified-release tablets (AstraZeneca UK Ltd)	Calcium channel blocker
9334	Plendil 2.5mg modified-release tablets (AstraZeneca UK Ltd)	Calcium channel blocker
74012	Plendil 2.5mg modified-release tablets (Waymade Healthcare Plc)	Calcium channel blocker
9437	Plendil 5mg modified-release tablets (AstraZeneca UK Ltd)	Calcium channel blocker
74004	Plendil 5mg modified-release tablets (Dowelhurst Ltd)	Calcium channel blocker
64504	Plendil 5mg modified-release tablets (Necessity Supplies Ltd)	Calcium channel blocker
3931	Posicor 100mg Tablet (Roche Products Ltd)	Calcium channel blocker
1529	Posicor 50mg Tablet (Roche Products Ltd)	Calcium channel blocker
8257	Prescal 2.5mg tablets (Novartis Pharmaceuticals UK Ltd)	Calcium channel blocker
19457	Ranvera MR 240mg tablets (Ranbaxy (UK) Ltd)	Calcium channel blocker
21795	Retalzem 60 modified-release tablets (Kent Pharmaceuticals Ltd)	Calcium channel blocker
18690	SECURON (CALENDAR PACK) 120 MG TAB	Calcium channel blocker
36684	SLOFEDIPINE 20 MG TAB	Calcium channel blocker
3057	Securon 120mg tablets (Abbott Laboratories Ltd)	Calcium channel blocker
22826	Securon 160mg Tablet (Abbott Laboratories Ltd)	Calcium channel blocker
8524	Securon 40mg Tablet (Abbott Laboratories Ltd)	Calcium channel

		blocker
10832	Securon 80mg Tablet (Abbott Laboratories Ltd)	Calcium channel blocker
27295	Securon IV 5mg/2ml solution for injection ampoules (Mylan)	Calcium channel blocker
59264	Securon SR 240mg tablets (DE Pharmaceuticals)	Calcium channel blocker
3342	Securon SR 240mg tablets (Mylan)	Calcium channel blocker
51461	Securon SR 240mg tablets (Waymade Healthcare Plc)	Calcium channel blocker
22696	Slofedipine 20mg tablets (Sterwin Medicines)	Calcium channel blocker
9573	Slofedipine XL 30mg tablets (Zentiva)	Calcium channel blocker
9553	Slofedipine XL 60 tablets (Zentiva)	Calcium channel blocker
2528	Slozem 120mg capsules (Merck Serono Ltd)	Calcium channel blocker
5234	Slozem 180mg capsules (Merck Serono Ltd)	Calcium channel blocker
4408	Slozem 240mg capsules (Merck Serono Ltd)	Calcium channel blocker
17586	Slozem 300mg capsules (Merck Serono Ltd)	Calcium channel blocker
19129	Syscor MR 10 tablets (Forest Laboratories UK Ltd)	Calcium channel blocker
31337	Syscor MR 20 tablets (Forest Laboratories UK Ltd)	Calcium channel blocker
31336	Syscor MR 30 tablets (Forest Laboratories UK Ltd)	Calcium channel blocker
19170	Tensipine MR 10 tablets (Thornton & Ross Ltd)	Calcium channel blocker
5806	Tensipine MR 20 tablets (Thornton & Ross Ltd)	Calcium channel blocker
56467	Tildiem 60mg modified-release tablets (DE Pharmaceuticals)	Calcium channel blocker
2888	Tildiem 60mg modified-release tablets (Sanofi)	Calcium channel blocker
57594	Tildiem 60mg modified-release tablets (Waymade Healthcare Plc)	Calcium channel blocker
52701	Tildiem LA 200 capsules (Mawdsley-Brooks & Company Ltd)	Calcium channel blocker
38545	Tildiem LA 200 capsules (Sanofi)	Calcium channel blocker
74022	Tildiem LA 200 capsules (Waymade Healthcare Plc)	Calcium channel blocker
54799	Tildiem LA 300 capsules (Mawdsley-Brooks & Company Ltd)	Calcium channel blocker
38876	Tildiem LA 300 capsules (Sanofi)	Calcium channel blocker
66048	Tildiem Retard 120mg tablets (DE Pharmaceuticals)	Calcium channel

		blocker
51261	Tildiem Retard 120mg tablets (Mawdsley-Brooks & Company Ltd)	Calcium channel blocker
1289	Tildiem Retard 120mg tablets (Sanofi)	Calcium channel blocker
70961	Tildiem Retard 90mg tablets (DE Pharmaceuticals)	Calcium channel blocker
74029	Tildiem Retard 90mg tablets (Dowelhurst Ltd)	Calcium channel blocker
939	Tildiem Retard 90mg tablets (Sanofi)	Calcium channel blocker
536	Tildiem la 200mg Modified-release capsule (Sanofi)	Calcium channel blocker
5296	Tildiem la 300mg Modified-release capsule (Sanofi)	Calcium channel blocker
59585	Uard 120XL capsules (Ennogen Healthcare Ltd)	Calcium channel blocker
66172	Uard 180XL capsules (Ennogen Healthcare Ltd)	Calcium channel blocker
66834	Uard 240XL capsules (Ennogen Healthcare Ltd)	Calcium channel blocker
67890	Uard 300XL capsules (Ennogen Healthcare Ltd)	Calcium channel blocker
12613	Unipine xl 30mg Modified-release tablet (Genus Pharmaceuticals Ltd)	Calcium channel blocker
6510	Univer 120mg modified-release capsules (Teva UK Ltd)	Calcium channel blocker
12392	Univer 180mg modified-release capsules (Teva UK Ltd)	Calcium channel blocker
8945	Univer 240mg modified-release capsules (Teva UK Ltd)	Calcium channel blocker
23730	VERAPAMIL 100 MG TAB	Calcium channel blocker
18631	VERAPAMIL HCL MR	Calcium channel blocker
10897	VERAPAMIL S/F 40 MG/5ML SOL	Calcium channel blocker
23458	VERAPAMIL SR	Calcium channel blocker
43222	Valni 20 Retard tablets (Tillomed Laboratories Ltd)	Calcium channel blocker
37184	Valni XL 30mg tablets (Zentiva)	Calcium channel blocker
39800	Valni XL 60mg tablets (Zentiva)	Calcium channel blocker
14305	Vascalpha 10mg modified-release tablets (Actavis UK Ltd)	Calcium channel blocker
43790	Vascalpha 10mg modified-release tablets (Almus Pharmaceuticals Ltd)	Calcium channel blocker
35084	Vascalpha 5mg modified-release tablets (Actavis UK Ltd)	Calcium channel blocker
40633	Vascalpha 5mg modified-release tablets (Almus Pharmaceuticals Ltd)	Calcium channel

		blocker
42625	Vera-Til SR 120mg tablets (Actavis UK Ltd)	Calcium channel blocker
700	Vera-Til SR 120mg tablets (Tillomed Laboratories Ltd)	Calcium channel blocker
43879	Vera-Til SR 240mg tablets (Actavis UK Ltd)	Calcium channel blocker
13251	Vera-Til SR 240mg tablets (Tillomed Laboratories Ltd)	Calcium channel blocker
1574	Verapamil 120mg modified-release capsules	Calcium channel blocker
9569	Verapamil 120mg modified-release tablets	Calcium channel blocker
47222	Verapamil 120mg modified-release tablets (A A H Pharmaceuticals Ltd)	Calcium channel blocker
1747	Verapamil 120mg tablets	Calcium channel blocker
46009	Verapamil 120mg tablets (Kent Pharmaceuticals Ltd)	Calcium channel blocker
41693	Verapamil 120mg tablets (Mylan)	Calcium channel blocker
40405	Verapamil 120mg tablets (Teva UK Ltd)	Calcium channel blocker
10688	Verapamil 160mg tablets	Calcium channel blocker
8975	Verapamil 180mg modified-release capsules	Calcium channel blocker
3943	Verapamil 240mg modified-release capsules	Calcium channel blocker
1298	Verapamil 240mg modified-release tablets	Calcium channel blocker
19459	Verapamil 240mg modified-release tablets (A A H Pharmaceuticals Ltd)	Calcium channel blocker
45308	Verapamil 240mg modified-release tablets (Mylan)	Calcium channel blocker
47230	Verapamil 240mg modified-release tablets (Teva UK Ltd)	Calcium channel blocker
1118	Verapamil 40mg tablets	Calcium channel blocker
34959	Verapamil 40mg tablets (A A H Pharmaceuticals Ltd)	Calcium channel blocker
33471	Verapamil 40mg tablets (Actavis UK Ltd)	Calcium channel blocker
19175	Verapamil 40mg tablets (IVAX Pharmaceuticals UK Ltd)	Calcium channel blocker
71009	Verapamil 40mg tablets (Kent Pharmaceuticals Ltd)	Calcium channel blocker
32590	Verapamil 40mg tablets (Mylan)	Calcium channel blocker
39009	Verapamil 40mg tablets (Teva UK Ltd)	Calcium channel blocker
11777	Verapamil 40mg/5ml oral solution sugar free	Calcium channel

		blocker
68531	Verapamil 40mg/5ml oral suspension	Calcium channel blocker
26674	Verapamil 5mg/2ml solution for injection ampoules	Calcium channel blocker
1120	Verapamil 80mg tablets	Calcium channel blocker
31711	Verapamil 80mg tablets (A A H Pharmaceuticals Ltd)	Calcium channel blocker
41586	Verapamil 80mg tablets (Actavis UK Ltd)	Calcium channel blocker
62552	Verapamil 80mg tablets (Alliance Healthcare (Distribution) Ltd)	Calcium channel blocker
41679	Verapamil 80mg tablets (IVAX Pharmaceuticals UK Ltd)	Calcium channel blocker
46955	Verapamil 80mg tablets (Mylan)	Calcium channel blocker
35729	Verapamil 80mg tablets (Teva UK Ltd)	Calcium channel blocker
45051	Verapamil hc 240mg Modified-release tablet (Actavis UK Ltd)	Calcium channel blocker
46884	Verapamil hc 240mg Modified-release tablet (Sandoz Ltd)	Calcium channel blocker
28843	Verapamil hc 80mg Tablet (Celltech Pharma Europe Ltd)	Calcium channel blocker
73467	Verapamil hc 80mg Tablet (Ranbaxy (UK) Ltd)	Calcium channel blocker
8759	Verapamil hcl 120mg modified release tablets	Calcium channel blocker
13856	Verapress MR 240mg tablets (Actavis UK Ltd)	Calcium channel blocker
16328	Verapress MR 240mg tablets (Dexcel-Pharma Ltd)	Calcium channel blocker
17599	Verapress MR 240mg tablets (Sandoz Ltd)	Calcium channel blocker
29637	Verapress MR 240mg tablets (Teva UK Ltd)	Calcium channel blocker
11972	Vertab SR 240 tablets (Chiesi Ltd)	Calcium channel blocker
2592	Viazem XL 120mg capsules (Thornton & Ross Ltd)	Calcium channel blocker
17666	Viazem XL 180mg capsules (Thornton & Ross Ltd)	Calcium channel blocker
13027	Viazem XL 240mg capsules (Thornton & Ross Ltd)	Calcium channel blocker
1130	Viazem XL 300mg capsules (Thornton & Ross Ltd)	Calcium channel blocker
7398	Viazem XL 360mg capsules (Thornton & Ross Ltd)	Calcium channel blocker
5570	Zanidip 10mg tablets (Recordati Pharmaceuticals Ltd)	Calcium channel blocker
14300	Zanidip 20mg tablets (Recordati Pharmaceuticals Ltd)	Calcium channel

		blocker
47732	Zemret 180 XL capsules (Tillomed Laboratories Ltd)	Calcium channel blocker
44192	Zemret 240 XL capsules (Tillomed Laboratories Ltd)	Calcium channel blocker
47608	Zemret 300 XL capsules (Tillomed Laboratories Ltd)	Calcium channel blocker
36583	Zemret xl 180mg Capsule (Neo Laboratories Ltd)	Calcium channel blocker
26463	Zemret xl 240mg Capsule (Neo Laboratories Ltd)	Calcium channel blocker
36664	Zemret xl 300mg Capsule (Neo Laboratories Ltd)	Calcium channel blocker
17425	Zemtard 120 XL capsules (Galen Ltd)	Calcium channel blocker
17406	Zemtard 180 XL capsules (Galen Ltd)	Calcium channel blocker
20890	Zemtard 240 XL capsules (Galen Ltd)	Calcium channel blocker
17492	Zemtard 300 XL capsules (Galen Ltd)	Calcium channel blocker
31737	Zildil SR 120mg capsules (Chanelle Medical UK Ltd)	Calcium channel blocker
26759	Zildil SR 60mg capsules (Chanelle Medical UK Ltd)	Calcium channel blocker
31490	Zolvera 40mg/5ml oral solution (Rosemont Pharmaceuticals Ltd)	Calcium channel blocker
30129	Abicol Tablet (Knoll Ltd)	Central-acting
8033	Aldomet 125mg Tablet (Merck Sharp & Dohme Ltd)	Central-acting
7626	Aldomet 250mg Tablet (Merck Sharp & Dohme Ltd)	Central-acting
43988	Aldomet 250mg tablets (Aspen Pharma Trading Ltd)	Central-acting
71385	Aldomet 250mg tablets (Sigma Pharmaceuticals Plc)	Central-acting
14390	Aldomet 250mg/5ml Liquid (Merck Sharp & Dohme Ltd)	Central-acting
7642	Aldomet 500mg Tablet (Merck Sharp & Dohme Ltd)	Central-acting
43989	Aldomet 500mg tablets (Aspen Pharma Trading Ltd)	Central-acting
71110	Aldomet 500mg tablets (Sigma Pharmaceuticals Plc)	Central-acting
7416	Aldomet 50mg/ml Injection (Merck Sharp & Dohme Ltd)	Central-acting
25088	CATAPRES	Central-acting
28790	CATAPRES	Central-acting
27883	CATAPRES 15 MCG/ML INJ	Central-acting
20808	CATAPRES PERLONGETS	Central-acting
27894	CLONIDINE HYDROCHLORIDE	Central-acting
4215	Catapres 100microgram tablets (Boehringer Ingelheim Ltd)	Central-acting
30293	Catapres 150micrograms/1ml solution for injection ampoules (Boehringer Ingelheim Ltd)	Central-acting
23380	Catapres 300microgram tablets (Boehringer Ingelheim Ltd)	Central-acting
8296	Catapres PL Perlongets 250microgram capsules (Boehringer Ingelheim Ltd)	Central-acting
2878	Clonidine 100microgram tablets	Central-acting
49684	Clonidine 100micrograms/24hours transdermal patches	Central-acting
60136	Clonidine 100micrograms/5ml oral solution	Central-acting

72130	Clonidine 100micrograms/5ml oral suspension	Central-acting
67200	Clonidine 10micrograms/5ml oral solution	Central-acting
16248	Clonidine 150micrograms/1ml solution for injection ampoules	Central-acting
72807	Clonidine 15micrograms/5ml oral solution	Central-acting
58529	Clonidine 200micrograms/24hours transdermal patches	Central-acting
5289	Clonidine 250microgram modified-release capsules	Central-acting
74534	Clonidine 250micrograms/5ml oral suspension	Central-acting
338	Clonidine 25microgram tablets	Central-acting
45578	Clonidine 25microgram tablets (A A H Pharmaceuticals Ltd)	Central-acting
33093	Clonidine 25microgram tablets (Sandoz Ltd)	Central-acting
63971	Clonidine 25microgram tablets (Sigma Pharmaceuticals Plc)	Central-acting
61710	Clonidine 25microgram tablets (Teva UK Ltd)	Central-acting
60089	Clonidine 25microgram tablets (Waymade Healthcare Plc)	Central-acting
55797	Clonidine 25micrograms/5ml oral solution	Central-acting
6694	Clonidine 300microgram tablets	Central-acting
54467	Clonidine 300micrograms/24hours transdermal patches	Central-acting
52555	Clonidine 50micrograms/5ml oral solution	Central-acting
53142	Clonidine 50micrograms/5ml oral suspension	Central-acting
61256	Clonidine 5micrograms/5ml oral suspension	Central-acting
58090	Clonidine 75micrograms/5ml oral solution	Central-acting
19635	DIXARIT	Central-acting
25393	Decaserpyl 10mg Tablet (Roussel Laboratories Ltd)	Central-acting
10713	Decaserpyl 5mg Tablet (Roussel Laboratories Ltd)	Central-acting
4406	Decaserpyl plus Tablet (Roussel Laboratories Ltd)	Central-acting
2630	Dixarit 25microgram tablets (Boehringer Ingelheim Ltd)	Central-acting
72227	Dixarit 25microgram tablets (DE Pharmaceuticals)	Central-acting
64284	Dixarit 25microgram tablets (Lexon (UK) Ltd)	Central-acting
29570	Dopamet 125mg Tablet (Berk Pharmaceuticals Ltd)	Central-acting
24196	Dopamet 250mg Tablet (Berk Pharmaceuticals Ltd)	Central-acting
25289	Dopamet 500mg Tablet (Berk Pharmaceuticals Ltd)	Central-acting
67005	Guanfacine 1mg modified-release tablets	Central-acting
67516	Guanfacine 2mg modified-release tablets	Central-acting
68529	Guanfacine 3mg modified-release tablets	Central-acting
67675	Guanfacine 4mg modified-release tablets	Central-acting
21346	Hydromet Tablet (MSD Thomas Morson Pharmaceuticals)	Central-acting
23345	Hypercal 2mg Tablet (Carlton Laboratories)	Central-acting
66016	Intuniv 1mg modified-release tablets (Shire Pharmaceuticals Ltd)	Central-acting
66936	Intuniv 2mg modified-release tablets (Shire Pharmaceuticals Ltd)	Central-acting
66388	Intuniv 3mg modified-release tablets (Shire Pharmaceuticals Ltd)	Central-acting
66429	Intuniv 4mg modified-release tablets (Shire Pharmaceuticals Ltd)	Central-acting
25836	METHYLDOPA 200 MG TAB	Central-acting
2649	METHYLDOPA 250 MG CAP	Central-acting
25275	Metalpha 500mg Tablet (Ashbourne Pharmaceuticals Ltd)	Central-acting
29187	Methoserpidine 10mg Tablet	Central-acting
10714	Methoserpidine 5mg Tablet	Central-acting
29696	Methoserpidine with benzthiazide Tablet	Central-acting
3049	Methyldopa 125mg tablets	Central-acting

9225	Methyldopa 250mg Capsule	Central-acting
41661	Methyldopa 250mg Tablet (C P Pharmaceuticals Ltd)	Central-acting
1707	Methyldopa 250mg tablets	Central-acting
32913	Methyldopa 250mg tablets (Actavis UK Ltd)	Central-acting
72819	Methyldopa 250mg tablets (Phoenix Healthcare Distribution Ltd)	Central-acting
73640	Methyldopa 250mg tablets (Sandoz Ltd)	Central-acting
62513	Methyldopa 250mg tablets (Sovereign Medical Ltd)	Central-acting
23761	Methyldopa 250mg/5ml oral suspension	Central-acting
3070	Methyldopa 500mg tablets	Central-acting
26919	Methyldopa 50mg/ml Injection	Central-acting
28738	Methyldopa with hydrochlorothiazide Tablet	Central-acting
4993	Moxonidine 200microgram tablets	Central-acting
62853	Moxonidine 200microgram tablets (A A H Pharmaceuticals Ltd)	Central-acting
60898	Moxonidine 200microgram tablets (Mylan)	Central-acting
33322	Moxonidine 200microgram tablets (Sandoz Ltd)	Central-acting
40310	Moxonidine 200microgram tablets (Teva UK Ltd)	Central-acting
10253	Moxonidine 300microgram tablets	Central-acting
63938	Moxonidine 300microgram tablets (Sandoz Ltd)	Central-acting
7174	Moxonidine 400microgram tablets	Central-acting
67665	Moxonidine 400microgram tablets (Actavis UK Ltd)	Central-acting
43531	Moxonidine 400microgram tablets (Sandoz Ltd)	Central-acting
67808	Physiotens 200microgram tablets (Actavis UK Ltd)	Central-acting
11177	Physiotens 200microgram tablets (Mylan)	Central-acting
61036	Physiotens 300microgram tablets (Actavis UK Ltd)	Central-acting
9876	Physiotens 300microgram tablets (Mylan)	Central-acting
9749	Physiotens 400microgram tablets (Mylan)	Central-acting
41894	RESERPINE/BENDROFLUAZIDE 150 MCG TAB	Central-acting
2104	Rauwiloid 2mg Tablet (3M Health Care Ltd)	Central-acting
22853	Reserpine 100micrograms tablet	Central-acting
20690	Reserpine 250micrograms tablet	Central-acting
15493	Reserpine with hydrochlorothiazide tablet	Central-acting
27602	SERPASIL	Central-acting
22466	SERPASIL	Central-acting
25645	Seominal Tablet (Sterling-Winthrop)	Central-acting
19892	Serpasil -esidrex Tablet (Novartis Pharmaceuticals UK Ltd)	Central-acting
21502	Serpasil 100microgram Tablet (Novartis Pharmaceuticals UK Ltd)	Central-acting
20656	Serpasil 250microgram Tablet (Novartis Pharmaceuticals UK Ltd)	Central-acting
17721	ALDACTIDE 100 MG TAB	Combined
19721	AMILORIDE 5MG/HYDROCHLORTHIAZIDE 50MG	Combined
19611	AMILORIDE 5MG/HYDROCHLORTHIAZIDE 50MG	Combined
15031	Accuretic 12.5mg/10mg tablets (Pfizer Ltd)	Combined
14126	Acebutolol 200mg / Hydrochlorothiazide 12.5mg tablets	Combined
18263	Acezide 25mg/50mg tablets (Bristol-Myers Squibb Pharmaceuticals Ltd)	Combined
62376	Actelsar HCT 80mg/12.5mg tablets (Actavis UK Ltd)	Combined
71748	Actelsar HCT 80mg/25mg tablets (Actavis UK Ltd)	Combined
23505	Adizem xl plus 150mg+12.5mg Modified-release capsule (Napp Pharmaceuticals Ltd)	Combined

2001	Aldactide 25 tablets (Pfizer Ltd)	Combined
1297	Aldactide 50 tablets (Pfizer Ltd)	Combined
20066	Amil-Co 5mg/50mg tablets (IVAX Pharmaceuticals UK Ltd)	Combined
18361	Amilmaxco 5mg/50mg tablets (Ashbourne Pharmaceuticals Ltd)	Combined
18497	Amiloride 10mg / furosemide 80mg tablets	Combined
5727	Amiloride 2.5mg / Cyclopenthiazide 250microgram tablets	Combined
15874	Amiloride 2.5mg / furosemide 20mg tablets	Combined
3701	Amiloride 2.5mg / hydrochlorothiazide 25mg tablets	Combined
14587	Amiloride 5mg / Bumetanide 1mg tablets	Combined
9456	Amiloride 5mg / furosemide 40mg tablets	Combined
2002	Amiloride 5mg / hydrochlorothiazide 50mg tablets	Combined
4034	Amiloride 5mg / hydrochlorothiazide 50mg/5ml solution	Combined
3526	Amiloride with atenolol with hydrochlorothiazide capsules	Combined
30519	Amiloride with timolol with hydrochlorothiazide tablets	Combined
35189	Amlodipine 10mg / Valsartan 160mg tablets	Combined
35343	Amlodipine 5mg / Valsartan 160mg tablets	Combined
35329	Amlodipine 5mg / Valsartan 80mg tablets	Combined
18332	Aridil 20mg+2.5mg Tablet (C P Pharmaceuticals Ltd)	Combined
13526	Atenix Co 100 tablets (Ashbourne Pharmaceuticals Ltd)	Combined
21873	Atenix Co 50 tablets (Ashbourne Pharmaceuticals Ltd)	Combined
1788	Atenolol 100mg with Chlortalidone 25mg tablets	Combined
9178	Atenolol 25mg / Bendroflumethiazide 1.25mg capsules	Combined
4542	Atenolol 50mg / Nifedipine 20mg modified-release capsules	Combined
581	Atenolol 50mg with Chlortalidone 12.5mg tablets	Combined
4983	Atenolol with amiloride and hydrochlorothiazide capsules	Combined
22912	Bendroflumethiazide 2.5mg with Propanolol 80mg capsules	Combined
19142	Bendroflumethiazide 2.5mg with Timolol maleate 10mg tablets	Combined
11338	Bendroflumethiazide 5mg with Nadolol 40mg tablets	Combined
69334	Bendroflumethiazide 5mg with Nadolol 80mg tablets	Combined
23131	Bendroflumethiazide 5mg with Propanolol 160mg modified-release capsules	Combined
30272	Benthiazide with Triamterene capsules	Combined
1684	Beta-Adalat modified-release capsules (Bayer Plc)	Combined
52728	Beta-Adalat modified-release capsules (Lexon (UK) Ltd)	Combined
68020	Beta-Adalat modified-release capsules (Sigma Pharmaceuticals Plc)	Combined
61719	Beta-Adalat modified-release capsules (Waymade Healthcare Plc)	Combined
17462	Bisoprolol 10mg / Hydrochlorothiazide 6.25mg tablets	Combined
2495	Bumetanide with Amiloride tablets	Combined
2493	Burinex A 5mg/1mg tablets (LEO Pharma)	Combined
30592	CHLOROTHIAZIDE / SPIRONOLACTONE / GLUCOS 60 MG POW	Combined
30034	CHLOROTHIAZIDE / SPIRONOLACTONE 100 MG POW	Combined
30035	CHLOROTHIAZIDE 40MG /SPIRONOLACTONE 4MG POW	Combined
30367	CHLOROTHIAZIDE 60MG / SPIRONOLACTONE 6MG POW	Combined
30368	CHLOROTHIAZIDE/SPIRONOLACTONE SACHETS 100 MG	Combined
55050	CHLOROTHIAZIDE/SPIRONOLACTONE/LACT SACH 50 MG	Combined
30365	CHLOROTHIAZIDE/SPIRONOLACTONE/LACTOSE MG POW	Combined
41897	CO-BETALOC	Combined
1520	Capozide 25mg/50mg tablets (Bristol-Myers Squibb Pharmaceuticals Ltd)	Combined

Carr MJ, et al. BMJ Qual Saf 2022; 31:503–514. doi: 10.1136/bmjqs-2021-013613

39227	Capozide LS 12.5mg/25mg tablets (Bristol-Myers Squibb Pharmaceuticals Ltd)	Combined
3203	Capozide LS Tablet (E R Squibb and Sons Ltd)	Combined
32166	Capto-co 25mg+50mg Tablet (IVAX Pharmaceuticals UK Ltd)	Combined
11641	Captopril 25mg with Hydrochlorothiazide 12.5mg tablets	Combined
10902	Captopril 50mg with Hydrochlorothiazide 25mg tablets	Combined
39242	Carace 10 Plus tablets (Merck Sharp & Dohme Ltd)	Combined
17655	Carace 10 Tablet (Bristol-Myers Squibb Pharmaceuticals Ltd)	Combined
39147	Carace 20 Plus tablets (Merck Sharp & Dohme Ltd)	Combined
9764	Carace 20 Tablet (Bristol-Myers Squibb Pharmaceuticals Ltd)	Combined
21231	Caralpha 20mg/12.5mg tablets (Actavis UK Ltd)	Combined
19055	Chlortalidone 12.5mg with Atenolol 50mg tablets	Combined
16786	Chlortalidone 25mg with Atenolol 100mg tablets	Combined
18287	Co-Betaloc SA tablets (Pfizer Ltd)	Combined
10627	Co-Betaloc tablets (Pfizer Ltd)	Combined
6877	Co-Diovan 160mg/12.5mg tablets (Novartis Pharmaceuticals UK Ltd)	Combined
25382	Co-Diovan 160mg/25mg tablets (Novartis Pharmaceuticals UK Ltd)	Combined
764	Co-Diovan 80mg/12.5mg tablets (Novartis Pharmaceuticals UK Ltd)	Combined
52858	Co-Diovan 80mg/12.5mg tablets (Sigma Pharmaceuticals Plc)	Combined
3793	Co-amilofruse 10mg/80mg tablets	Combined
34622	Co-amilofruse 10mg/80mg tablets (Wockhardt UK Ltd)	Combined
193	Co-amilofruse 2.5mg/20mg tablets	Combined
60258	Co-amilofruse 2.5mg/20mg tablets (Milpharm Ltd)	Combined
34280	Co-amilofruse 2.5mg/20mg tablets (Sandoz Ltd)	Combined
41533	Co-amilofruse 2.5mg/20mg tablets (Teva UK Ltd)	Combined
25965	Co-amilofruse 2.5mg/20mg tablets (Wockhardt UK Ltd)	Combined
30773	Co-amilofruse 5mg+40mg Tablet (Berk Pharmaceuticals Ltd)	Combined
56	Co-amilofruse 5mg/40mg tablets	Combined
33658	Co-amilofruse 5mg/40mg tablets (A A H Pharmaceuticals Ltd)	Combined
41719	Co-amilofruse 5mg/40mg tablets (Actavis UK Ltd)	Combined
57908	Co-amilofruse 5mg/40mg tablets (Kent Pharmaceuticals Ltd)	Combined
33527	Co-amilofruse 5mg/40mg tablets (Mylan)	Combined
43508	Co-amilofruse 5mg/40mg tablets (Sandoz Ltd)	Combined
28129	Co-amilofruse 5mg/40mg tablets (Teva UK Ltd)	Combined
59412	Co-amilofruse 5mg/40mg tablets (Waymade Healthcare Plc)	Combined
31773	Co-amilofruse 5mg/40mg tablets (Wockhardt UK Ltd)	Combined
47647	Co-amilofruse oral liquid	Combined
924	Co-amilozide 2.5mg/25mg tablets	Combined
60354	Co-amilozide 2.5mg/25mg tablets (Kent Pharmaceuticals Ltd)	Combined
75069	Co-amilozide 2.5mg/25mg tablets (Mawdsley-Brooks & Company Ltd)	Combined
34367	Co-amilozide 2.5mg/25mg tablets (Wockhardt UK Ltd)	Combined
18733	Co-amilozide 5mg with 50mg/ml oral solution	Combined
923	Co-amilozide 5mg/50mg tablets	Combined
46916	Co-amilozide 5mg/50mg tablets (A A H Pharmaceuticals Ltd)	Combined
62249	Co-amilozide 5mg/50mg tablets (Alliance Healthcare (Distribution) Ltd)	Combined
31150	Co-amilozide 5mg/50mg tablets (IVAX Pharmaceuticals UK Ltd)	Combined
62700	Co-amilozide 5mg/50mg tablets (Phoenix Healthcare Distribution Ltd)	Combined

Carr MJ, *et al. BMJ Qual Saf* 2022; 31:503–514. doi: 10.1136/bmjqs-2021-013613

41556	Co-amilozide 5mg/50mg tablets (Teva UK Ltd)	Combined
73337	Co-amilozide 5mg/50mg tablets (Wockhardt UK Ltd)	Combined
15811	Co-flumactone 25mg/25mg tablets	Combined
11384	Co-flumactone 50mg/50mg tablets	Combined
13871	Co-prenozide 160mg/0.25mg modified-release tablets	Combined
5721	Co-tenidone 100mg/25mg tablets	Combined
34899	Co-tenidone 100mg/25mg tablets (A A H Pharmaceuticals Ltd)	Combined
46952	Co-tenidone 100mg/25mg tablets (Actavis UK Ltd)	Combined
62537	Co-tenidone 100mg/25mg tablets (DE Pharmaceuticals)	Combined
34012	Co-tenidone 100mg/25mg tablets (IVAX Pharmaceuticals UK Ltd)	Combined
37725	Co-tenidone 100mg/25mg tablets (Mylan)	Combined
41572	Co-tenidone 100mg/25mg tablets (Teva UK Ltd)	Combined
9783	Co-tenidone 50mg/12.5mg tablets	Combined
32094	Co-tenidone 50mg/12.5mg tablets (A A H Pharmaceuticals Ltd)	Combined
31708	Co-tenidone 50mg/12.5mg tablets (Actavis UK Ltd)	Combined
34034	Co-tenidone 50mg/12.5mg tablets (IVAX Pharmaceuticals UK Ltd)	Combined
34449	Co-tenidone 50mg/12.5mg tablets (Mylan)	Combined
34825	Co-tenidone 50mg/12.5mg tablets (Teva UK Ltd)	Combined
5416	Co-triamterzide 50mg/25mg tablets	Combined
47804	Co-triamterzide 50mg/25mg tablets (A A H Pharmaceuticals Ltd)	Combined
11561	Co-zidocapt 12.5mg/25mg tablets	Combined
11351	Co-zidocapt 25mg/50mg tablets	Combined
10316	CoAprovel 150mg/12.5mg tablets (Sanofi)	Combined
11526	CoAprovel 300mg/12.5mg tablets (Sanofi)	Combined
35196	CoAprovel 300mg/25mg tablets (Sanofi)	Combined
5330	Corgaretic 40mg tablets (Sanofi-Synthelabo Ltd)	Combined
14438	Corgaretic 80mg tablets (Sanofi-Synthelabo Ltd)	Combined
51258	Coversyl Arginine Plus 5mg/1.25mg tablets (DE Pharmaceuticals)	Combined
37908	Coversyl Arginine Plus 5mg/1.25mg tablets (Servier Laboratories Ltd)	Combined
14228	Coversyl Plus tablets (Servier Laboratories Ltd)	Combined
37747	Cozaar-Comp 100mg/12.5mg tablets (Merck Sharp & Dohme Ltd)	Combined
21423	Cozaar-Comp 100mg/25mg tablets (Merck Sharp & Dohme Ltd)	Combined
57796	Cozaar-Comp 50mg/12.5mg tablets (DE Pharmaceuticals)	Combined
4540	Cozaar-Comp 50mg/12.5mg tablets (Merck Sharp & Dohme Ltd)	Combined
55160	Cozaar-Comp 50mg/12.5mg tablets (Sigma Pharmaceuticals Plc)	Combined
52145	Cyclopenthiazide 0.25mg with oxprenolol 160mg modified-release tablets	Combined
26220	Delvas Tablet (Berk Pharmaceuticals Ltd)	Combined
18606	Diltiazem and hydrochlorothiazide 150mg+12.5mg modified-release capsules	Combined
1721	Dyazide 50mg/25mg tablets (AMCo)	Combined
67801	Dyazide 50mg/25mg tablets (Lexon (UK) Ltd)	Combined
7136	Dytide capsules (Mercury Pharma Group Ltd)	Combined
5189	Enalapril 20mg / Hydrochlorothiazide 12.5mg tablets	Combined
35096	Exforge 10mg/160mg tablets (Novartis Pharmaceuticals UK Ltd)	Combined
35697	Exforge 5mg/160mg tablets (Novartis Pharmaceuticals UK Ltd)	Combined
35317	Exforge 5mg/80mg tablets (Novartis Pharmaceuticals UK Ltd)	Combined
3902	FRUSEMIDE 20MG/SPIRONOLACTONE 50MG MG CAP	Combined

25075	FRUSEMIDE 40MG/AMILORIDE HYD 5MG	Combined
21162	Felodipine 2.5mg modified-release / Ramipril 2.5mg tablets	Combined
17474	Felodipine 5mg modified-release / Ramipril 5mg tablets	Combined
21938	Froop Co 5mg/40mg tablets (Ashbourne Pharmaceuticals Ltd)	Combined
4873	Fru-Co 5mg/40mg tablets (Teva UK Ltd)	Combined
211	Frumil 40mg+5mg Tablet (Helios Healthcare Ltd)	Combined
74800	Frumil 40mg/5mg tablets (Lexon (UK) Ltd)	Combined
39807	Frumil 40mg/5mg tablets (Sanofi)	Combined
73993	Frumil 40mg/5mg tablets (Sigma Pharmaceuticals Plc)	Combined
71348	Frumil 40mg/5mg tablets (Waymade Healthcare Plc)	Combined
13435	Frumil Forte 10mg/80mg tablets (Sanofi)	Combined
38901	Frumil LS 20mg/2.5mg tablets (Sanofi)	Combined
71377	Frumil LS 20mg/2.5mg tablets (Waymade Healthcare Plc)	Combined
1301	Frumil ls 20mg+2.5mg Tablet (Helios Healthcare Ltd)	Combined
9431	Frusemek 40mg+5mg Tablet (Approved Prescription Services Ltd)	Combined
2961	Frusene 50mg/40mg tablets (Orion Pharma (UK) Ltd)	Combined
4211	Furosemide with amiloride 20mg+2.5mg Tablet	Combined
1369	Furosemide with amiloride 40mg+5mg Tablet	Combined
5220	Furosemide with amiloride 80mg+10mg Tablet	Combined
26529	Furosemide with penbutolol Tablet	Combined
3050	Furosemide with triamterene 40mgwith50mg Tablet	Combined
60780	Generic Sevikar HCT 20mg/5mg/12.5mg tablets	Combined
60007	Generic Sevikar HCT 40mg/10mg/12.5mg tablets	Combined
24520	HYDROCHLOROTHIAZIDE /METOPROLOL TARTRATE 25 MG TAB	Combined
19890	Hydrochlorothiazide with amiloride 25mgwith2.5mg Tablet	Combined
22923	Hydrochlorothiazide with amiloride 50mg with 5mg Tablet	Combined
28177	Hydrochlorothiazide with atenolol and amiloride Capsule	Combined
15135	Hydrochlorothiazide with captopril 12.5mg with 25mg Tablet	Combined
11133	Hydrochlorothiazide with captopril 25mg with 50mg Tablet	Combined
38367	Hydrochlorothiazide with losartan 12.5mg with 100mg Tablet	Combined
14738	Hydrochlorothiazide with losartan 12.5mg with 50mg Tablet	Combined
24632	Hydrochlorothiazide with losartan 25mg with 100mg Tablet	Combined
29427	Hydrochlorothiazide with metoprolol tartrate 12.5mg with 100mg tablet	Combined
33659	Hydrochlorothiazide with metoprolol tartrate 25mg with 200mg Modified-release tablet	Combined
35380	Hydrochlorothiazide with olmesartan medoxomil 12.5mg with 20mg tablet	Combined
39021	Hydrochlorothiazide with olmesartan medoxomil 25mg with 20mg tablet	Combined
21182	Hydrochlorothiazide with timolol and amiloride 25mg with 10mg with 2.5mg Tablet	Combined
15127	Hydrochlorothiazide with triamterene 25mgwith50mg Tablet	Combined
24484	Hydrochlorothiazide with valsartan 12.5mg with 160mg Tablet	Combined
24268	Hydrochlorothiazide with valsartan 12.5mg with 80mg Tablet	Combined
23456	Hydrochlorothiazide with valsartan 25mg with 160mg Tablet	Combined
29529	Hydroflumethiazide with spironolactone 25mg+25mg Tablet	Combined
45916	Hydroflumethiazide with spironolactone 50mg+50mg Tablet	Combined
25500	Hypertane 50 Tablet (Schwarz Pharma Ltd)	Combined
4796	Inderetic 80mg/2.5mg capsules (AstraZeneca UK Ltd)	Combined

8369	Inderex 160mg/5mg modified-release capsules (AstraZeneca UK Ltd)	Combined
1021	Innozide 20mg/12.5mg tablets (Merck Sharp & Dohme Ltd)	Combined
11448	Irbesartan 150mg / Hydrochlorothiazide 12.5mg tablets	Combined
11469	Irbesartan 300mg / Hydrochlorothiazide 12.5mg tablets	Combined
62337	Irbesartan 300mg / Hydrochlorothiazide 12.5mg tablets (Actavis UK Ltd)	Combined
35481	Irbesartan 300mg / Hydrochlorothiazide 25mg tablets	Combined
12546	Kalspare Tablet (Dominion Pharma)	Combined
28157	Kalspare ls Tablet (Dominion Pharma)	Combined
16498	Kalspare tablets (DHP Healthcare Ltd)	Combined
7543	Kalten capsules (M & A Pharmachem Ltd)	Combined
7441	Lasilactone 20mg/50mg capsules (Sanofi)	Combined
24832	Lasipressin Tablet (Hoechst UK Ltd)	Combined
2772	Lasoride 5mg/40mg tablets (Sanofi)	Combined
6786	Lisinopril 10mg / Hydrochlorothiazide 12.5mg tablets	Combined
67767	Lisinopril 10mg / Hydrochlorothiazide 12.5mg tablets (Almus Pharmaceuticals Ltd)	Combined
37710	Lisinopril 10mg / Hydrochlorothiazide 12.5mg tablets (Teva UK Ltd)	Combined
6468	Lisinopril 20mg / Hydrochlorothiazide 12.5mg tablets	Combined
55399	Lisinopril 20mg / Hydrochlorothiazide 12.5mg tablets (A A H Pharmaceuticals Ltd)	Combined
54201	Lisinopril 20mg / Hydrochlorothiazide 12.5mg tablets (Almus Pharmaceuticals Ltd)	Combined
33353	Lisinopril 20mg / Hydrochlorothiazide 12.5mg tablets (Teva UK Ltd)	Combined
56244	Lisinopril 20mg / Hydrochlorothiazide 12.5mg tablets (Tillomed Laboratories Ltd)	Combined
8147	Lopresoretic Tablet (Novartis Pharmaceuticals UK Ltd)	Combined
37650	Losartan 100mg / Hydrochlorothiazide 12.5mg tablets	Combined
71618	Losartan 100mg / Hydrochlorothiazide 12.5mg tablets (A A H Pharmaceuticals Ltd)	Combined
70922	Losartan 100mg / Hydrochlorothiazide 12.5mg tablets (Phoenix Healthcare Distribution Ltd)	Combined
48039	Losartan 100mg / Hydrochlorothiazide 12.5mg tablets (Teva UK Ltd)	Combined
10323	Losartan 100mg / Hydrochlorothiazide 25mg tablets	Combined
52189	Losartan 100mg / Hydrochlorothiazide 25mg tablets (A A H Pharmaceuticals Ltd)	Combined
71682	Losartan 100mg / Hydrochlorothiazide 25mg tablets (Mawdsley-Brooks & Company Ltd)	Combined
6437	Losartan 50mg / Hydrochlorothiazide 12.5mg tablets	Combined
56975	Losartan 50mg / Hydrochlorothiazide 12.5mg tablets (A A H Pharmaceuticals Ltd)	Combined
56204	Losartan 50mg / Hydrochlorothiazide 12.5mg tablets (Actavis UK Ltd)	Combined
74215	Losartan 50mg / Hydrochlorothiazide 12.5mg tablets (DE Pharmaceuticals)	Combined
66598	Losartan 50mg / Hydrochlorothiazide 12.5mg tablets (Lupin Healthcare (UK) Ltd)	Combined
70754	Losartan 50mg / Hydrochlorothiazide 12.5mg tablets (Ranbaxy (UK) Ltd)	Combined
62911	Losartan 50mg / Hydrochlorothiazide 12.5mg tablets (Teva UK Ltd)	Combined
41892	METOPROLOL 100MG/HYDROCHLOROTHIAZ.12.5MG	Combined
19687	MODUCREN	Combined
7066	Metoprolol 100mg / Hydrochlorothiazide 12.5mg tablets	Combined

20093	Metoprolol 200mg modified-release / Hydrochlorothiazide 25mg tablets	Combined
15488	Metoprolol tartrate with chlortalidone Tablet	Combined
18202	MicardisPlus 40mg/12.5mg tablets (Boehringer Ingelheim Ltd)	Combined
66997	MicardisPlus 40mg/12.5mg tablets (Waymade Healthcare Plc)	Combined
17689	MicardisPlus 80mg/12.5mg tablets (Boehringer Ingelheim Ltd)	Combined
63890	MicardisPlus 80mg/12.5mg tablets (Waymade Healthcare Plc)	Combined
38889	MicardisPlus 80mg/25mg tablets (Boehringer Ingelheim Ltd)	Combined
4605	Moducren tablets (Merck Sharp & Dohme Ltd)	Combined
1251	Moduret 25 tablets (Merck Sharp & Dohme Ltd)	Combined
42142	Moduretic 5mg/50mg tablets (Merck Sharp & Dohme Ltd)	Combined
74017	Moduretic 5mg/50mg tablets (Waymade Healthcare Plc)	Combined
3293	Moduretic Oral solution (Bristol-Myers Squibb Pharmaceuticals Ltd)	Combined
348	Moduretic Tablet (Bristol-Myers Squibb Pharmaceuticals Ltd)	Combined
17149	Monozide 10 tablets (Wyeth Pharmaceuticals)	Combined
27086	NADOLOL 80MG/BENDROFLUAZIDE 5MG MG TAB	Combined
23134	Nadolol 40mg / Bendroflumethiazide 5mg tablets	Combined
27946	Nadolol 80mg / Bendroflumethiazide 5mg tablets	Combined
2255	Navispare 2.5mg/250microgram tablets (AMCo)	Combined
15117	Nifedipine with atenolol 20mg + 50mg Capsule	Combined
8058	Normetic Tablet (Abbott Laboratories Ltd)	Combined
40316	Olmesartan medoxomil 20mg / Amlodipine 5mg tablets	Combined
18200	Olmesartan medoxomil 20mg / Hydrochlorothiazide 12.5mg tablets	Combined
18903	Olmesartan medoxomil 20mg / Hydrochlorothiazide 25mg tablets	Combined
40668	Olmesartan medoxomil 40mg / Amlodipine 10mg tablets	Combined
40639	Olmesartan medoxomil 40mg / Amlodipine 5mg tablets	Combined
43322	Olmesartan medoxomil 40mg / Hydrochlorothiazide 12.5mg tablets	Combined
46687	Olmesartan medoxomil with amlodipine and hydrochlorothiazide 20mg + 5mg + 12.5mg Tablet	Combined
46715	Olmesartan medoxomil with amlodipine and hydrochlorothiazide 40mg + 10mg + 12.5mg Tablet	Combined
55358	Olmesartan medoxomil with amlodipine and hydrochlorothiazide 40mg + 10mg + 25mg Tablet	Combined
46792	Olmesartan medoxomil with amlodipine and hydrochlorothiazide 40mg + 5mg + 12.5mg Tablet	Combined
47467	Olmesartan medoxomil with amlodipine and hydrochlorothiazide 40mg + 5mg + 25mg Tablet	Combined
29634	Olmetec Plus 20mg/12.5mg tablets (Daiichi Sankyo UK Ltd)	Combined
27520	Olmetec Plus 20mg/25mg tablets (Daiichi Sankyo UK Ltd)	Combined
43915	Olmetec Plus 40mg/12.5mg tablets (Daiichi Sankyo UK Ltd)	Combined
8673	Oxprenolol with cyclopenthiazide 160mg+0.25mg Modified-release tablet	Combined
50607	Perindopril arginine 2mg with Indapamide 625 micrograms tablet	Combined
48098	Perindopril arginine 4mg with Indapamide 1.25mg tablet	Combined
37978	Perindopril arginine 5mg / Indapamide 1.25mg tablets	Combined
60684	Perindopril erbumine 4mg / Amlodipine 10mg tablets	Combined
60067	Perindopril erbumine 4mg / Amlodipine 5mg tablets	Combined
6794	Perindopril erbumine 4mg / Indapamide 1.25mg tablets	Combined
63149	Perindopril erbumine 8mg / Amlodipine 10mg tablets	Combined
60744	Perindopril erbumine 8mg / Amlodipine 5mg tablets	Combined

56157	Perindopril tosilate 5mg / Indapamide 1.25mg tablets	Combined
8623	Prestim Tablet (ICN Pharmaceuticals France S.A.)	Combined
21025	Prestim forte Tablet (LEO Pharma)	Combined
25363	Prestim tablets (Meda Pharmaceuticals Ltd)	Combined
8987	Propranolol 160mg modified-release / Bendroflumethiazide 5mg capsules	Combined
12054	Propranolol 80mg / Bendroflumethiazide 2.5mg capsules	Combined
15108	Quinapril 10mg / Hydrochlorothiazide 12.5mg tablets	Combined
11965	Ramipril 2.5mg with felodipine 2.5mg modified-release tablet	Combined
11567	Ramipril 5mg with felodipine 5mg modified-release tablet	Combined
29976	SOTAZIDE	Combined
19003	SPIRONOLACTONE/PROPRANOLOL 50 MG TAB	Combined
8189	Secadrex 200mg/12.5mg tablets (Sanofi)	Combined
39984	Sevikar 20mg/5mg tablets (Daiichi Sankyo UK Ltd)	Combined
41203	Sevikar 40mg/10mg tablets (Daiichi Sankyo UK Ltd)	Combined
41205	Sevikar 40mg/5mg tablets (Daiichi Sankyo UK Ltd)	Combined
46355	Sevikar HCT 20mg/5mg/12.5mg tablets (Daiichi Sankyo UK Ltd)	Combined
47616	Sevikar HCT 40mg/10mg/12.5mg tablets (Daiichi Sankyo UK Ltd)	Combined
53220	Sevikar HCT 40mg/10mg/25mg tablets (Daiichi Sankyo UK Ltd)	Combined
47573	Sevikar HCT 40mg/5mg/12.5mg tablets (Daiichi Sankyo UK Ltd)	Combined
47727	Sevikar HCT 40mg/5mg/25mg tablets (Daiichi Sankyo UK Ltd)	Combined
3691	Sotalol 160mg with hydrochlorothiazide 25mg tablet	Combined
8061	Sotalol 80mg with hydrochlorothiazide 12.5mg tablet	Combined
12456	Sotazide Tablet (Bristol-Myers Squibb Pharmaceuticals Ltd)	Combined
31131	Spiro-co 25mg+25mg Tablet (IVAX Pharmaceuticals UK Ltd)	Combined
25505	Spiro-co 50mg+50mg Tablet (IVAX Pharmaceuticals UK Ltd)	Combined
8521	Spironolactone 25mg with hydroflumethiazide 25mg tablet	Combined
4661	Spironolactone 50mg / Furosemide 20mg capsules	Combined
7961	Spironolactone 50mg with hydroflumethiazide 50mg tablet	Combined
17783	Spiroprop Tablet (Pharmacia Ltd)	Combined
8788	TIMOLOL 10MG/BENDROFLUAZIDE 2.5MG TAB	Combined
25787	TOTARETIC 100 TAB	Combined
26731	TOTARETIC 50 TAB	Combined
41889	TRIAMTERENE 50MG HYDROCHLOROTHIAZIDE25MG	Combined
3962	TRIAMTERENE 50MG HYDROCHLOROTHIAZIDE25MG TAB	Combined
20579	Tarka modified-release capsules (Abbott Laboratories Ltd)	Combined
14870	Telmisartan 40mg / Hydrochlorothiazide 12.5mg tablets	Combined
16161	Telmisartan 80mg / Hydrochlorothiazide 12.5mg tablets	Combined
38459	Telmisartan 80mg / Hydrochlorothiazide 25mg tablets	Combined
18743	Tenben 25mg/1.25mg capsules (Galen Ltd)	Combined
26248	Tenchlor 100mg/25mg tablets (Teva UK Ltd)	Combined
31470	Tenchlor 50mg/12.5mg tablets (Teva UK Ltd)	Combined
8642	Tenif 50mg/20mg modified-release capsules (AstraZeneca UK Ltd)	Combined
74039	Tenif 50mg/20mg modified-release capsules (Sigma Pharmaceuticals Plc)	Combined
1288	Tenoret 50mg/12.5mg tablets (AstraZeneca UK Ltd)	Combined
1124	Tenoretic 100mg/25mg tablets (AstraZeneca UK Ltd)	Combined
48745	Timolol 10mg / Amiloride 2.5mg / Hydrochlorothiazide 25mg tablets	Combined
12651	Timolol 10mg / Bendroflumethiazide 2.5mg tablets	Combined

Carr MJ, et al. BMJ Qual Saf 2022; 31:503–514. doi: 10.1136/bmjqs-2021-013613

25730	Timolol maleate with amiloride and hydrochlorothiazide Tablet	Combined
12517	Timolol maleate with bendroflumethiazide 20mg + 5mg Tablet	Combined
15042	Tolerzide Tablet (Bristol-Myers Squibb Pharmaceuticals Ltd)	Combined
24280	Totaretic 100mg+25mg Tablet (C P Pharmaceuticals Ltd)	Combined
26741	Totaretic 50mg+12.5mg Tablet (C P Pharmaceuticals Ltd)	Combined
18223	Trandolapril with verapamil 2mg + 180mg Modified-release capsule	Combined
4429	Trasidrex modified-release tablets (Mercury Pharma Group Ltd)	Combined
8897	Triam-Co 50mg/25mg tablets (IVAX Pharmaceuticals UK Ltd)	Combined
18726	Triamaxco 50mg/25mg tablets (Ashbourne Pharmaceuticals Ltd)	Combined
7740	Triamterene 50mg / Benzthiazide 25mg capsules	Combined
12547	Triamterene 50mg / Chlortalidone 50mg tablets	Combined
11265	Triamterene 50mg / Furosemide 40mg tablets	Combined
37294	Triamterene with chlortalidone 50mg + 25mg Tablet	Combined
9223	Triamterene with hydrochlorothiazide 50mg + 25mg Tablet	Combined
28438	Triapin 2.5mg/2.5mg modified-release tablets (Sanofi)	Combined
17006	Triapin 5mg/5mg modified-release tablets (Sanofi)	Combined
11864	Valsartan 160mg / Hydrochlorothiazide 12.5mg tablets	Combined
72086	Valsartan 160mg / Hydrochlorothiazide 12.5mg tablets (Actavis UK Ltd)	Combined
67664	Valsartan 160mg / Hydrochlorothiazide 12.5mg tablets (Teva UK Ltd)	Combined
14283	Valsartan 160mg / Hydrochlorothiazide 25mg tablets	Combined
35304	Valsartan 160mg with amlodipine 10mg tablets	Combined
35173	Valsartan 160mg with amlodipine 5mg tablets	Combined
16060	Valsartan 80mg / Hydrochlorothiazide 12.5mg tablets	Combined
35174	Valsartan 80mg with amlodipine 5mg tablets	Combined
24008	Vasetic Tablet (Shire Pharmaceuticals Ltd)	Combined
19690	Verapamil 180mg modified-release / Trandolapril 2mg capsules	Combined
39137	Zestoretic 10 tablets (AstraZeneca UK Ltd)	Combined
57539	Zestoretic 10 tablets (Sigma Pharmaceuticals Plc)	Combined
71115	Zestoretic 10 tablets (Waymade Healthcare Plc)	Combined
6359	Zestoretic 10- 10mg+12.5mg Tablet (AstraZeneca UK Ltd)	Combined
38995	Zestoretic 20 tablets (AstraZeneca UK Ltd)	Combined
74874	Zestoretic 20 tablets (DE Pharmaceuticals)	Combined
74040	Zestoretic 20 tablets (Lexon (UK) Ltd)	Combined
74008	Zestoretic 20 tablets (Waymade Healthcare Plc)	Combined
2982	Zestoretic 20- 20mg+12.5mg Tablet (AstraZeneca UK Ltd)	Combined
26219	Zida-co 5mg+50mg Tablet (Opus Pharmaceuticals Ltd)	Combined
2681	AMILORIDE 10 MG TAB	Diuretics
19695	AMILORIDE HYDROCHLORIDE	Diuretics
3285	AMILORIDE S/F 5 MG/5ML SOL	Diuretics
21848	AMILOSPARE 5 MG TAB	Diuretics
7952	Aldactone 100mg tablets (Pfizer Ltd)	Diuretics
2389	Aldactone 25mg tablets (Pfizer Ltd)	Diuretics
4960	Aldactone 50mg tablets (Pfizer Ltd)	Diuretics
31375	Amilamont 5mg/5ml oral solution sugar free (Rosemont Pharmaceuticals Ltd)	Diuretics
44254	Amiloride 5.67mg tablets	Diuretics
41630	Amiloride 5mg Tablet (IVAX Pharmaceuticals UK Ltd)	Diuretics

1060	Amiloride 5mg tablets	Diuretics
33837	Amiloride 5mg tablets (A A H Pharmaceuticals Ltd)	Diuretics
34750	Amiloride 5mg tablets (Actavis UK Ltd)	Diuretics
43523	Amiloride 5mg tablets (Mylan)	Diuretics
34324	Amiloride 5mg tablets (Teva UK Ltd)	Diuretics
46930	Amiloride 5mg tablets (Wockhardt UK Ltd)	Diuretics
9935	Amiloride 5mg/5ml oral solution sugar free	Diuretics
60149	Amiloride 5mg/5ml oral suspension	Diuretics
24893	Amilospare Tablet (Ashbourne Pharmaceuticals Ltd)	Diuretics
8526	Aprinox 2.5mg tablets (AMCo)	Diuretics
7698	Aprinox 5mg tablets (Amdipharm Plc)	Diuretics
7709	Arelix 6mg Capsule (Hoechst Marion Roussel)	Diuretics
27555	BURINEX	Diuretics
19683	BURINEX K	Diuretics
66517	Bendroflumethiazide 1.25mg/5ml oral suspension	Diuretics
17561	Bendroflumethiazide 2.5mg / Potassium chloride 573mg (potassium 7.7mmol) modified-release tablets	Diuretics
1211	Bendroflumethiazide 2.5mg / Potassium chloride 630mg (potassium 8.4mmol) modified-release tablets	Diuretics
72914	Bendroflumethiazide 2.5mg Tablet (Celltech Pharma Europe Ltd)	Diuretics
34803	Bendroflumethiazide 2.5mg Tablet (Regent Laboratories Ltd)	Diuretics
2	Bendroflumethiazide 2.5mg tablets	Diuretics
33651	Bendroflumethiazide 2.5mg tablets (A A H Pharmaceuticals Ltd)	Diuretics
34059	Bendroflumethiazide 2.5mg tablets (Actavis UK Ltd)	Diuretics
72042	Bendroflumethiazide 2.5mg tablets (Alliance Healthcare (Distribution) Ltd)	Diuretics
40886	Bendroflumethiazide 2.5mg tablets (Almus Pharmaceuticals Ltd)	Diuretics
67737	Bendroflumethiazide 2.5mg tablets (Dr Reddy's Laboratories (UK) Ltd)	Diuretics
70989	Bendroflumethiazide 2.5mg tablets (Genesis Pharmaceuticals Ltd)	Diuretics
27689	Bendroflumethiazide 2.5mg tablets (IVAX Pharmaceuticals UK Ltd)	Diuretics
47844	Bendroflumethiazide 2.5mg tablets (Kent Pharmaceuticals Ltd)	Diuretics
33415	Bendroflumethiazide 2.5mg tablets (Mylan)	Diuretics
34602	Bendroflumethiazide 2.5mg tablets (Sovereign Medical Ltd)	Diuretics
31670	Bendroflumethiazide 2.5mg tablets (Teva UK Ltd)	Diuretics
27256	Bendroflumethiazide 2.5mg tablets (Wockhardt UK Ltd)	Diuretics
7351	Bendroflumethiazide 2.5mg/5ml oral suspension	Diuretics
58	Bendroflumethiazide 5mg tablets	Diuretics
23427	Bendroflumethiazide 5mg tablets (A A H Pharmaceuticals Ltd)	Diuretics
34124	Bendroflumethiazide 5mg tablets (Actavis UK Ltd)	Diuretics
64907	Bendroflumethiazide 5mg tablets (Almus Pharmaceuticals Ltd)	Diuretics
67780	Bendroflumethiazide 5mg tablets (DE Pharmaceuticals)	Diuretics
67738	Bendroflumethiazide 5mg tablets (Dr Reddy's Laboratories (UK) Ltd)	Diuretics
40149	Bendroflumethiazide 5mg tablets (IVAX Pharmaceuticals UK Ltd)	Diuretics
41517	Bendroflumethiazide 5mg tablets (Teva UK Ltd)	Diuretics
72083	Bendroflumethiazide 5mg tablets (Waymade Healthcare Plc)	Diuretics
31820	Bendroflumethiazide 5mg tablets (Wockhardt UK Ltd)	Diuretics
53812	Bendroflumethiazide oral solution	Diuretics
26217	Berkamil 5mg Tablet (Berk Pharmaceuticals Ltd)	Diuretics

21803	Berkozide 2.5mg Tablet (Berk Pharmaceuticals Ltd)	Diuretics
21867	Berkozide 5mg Tablet (Berk Pharmaceuticals Ltd)	Diuretics
30913	Betinex 1mg Tablet (Berk Pharmaceuticals Ltd)	Diuretics
814	Bumetanide 1mg tablets	Diuretics
32091	Bumetanide 1mg tablets (A A H Pharmaceuticals Ltd)	Diuretics
39602	Bumetanide 1mg tablets (Actavis UK Ltd)	Diuretics
55548	Bumetanide 1mg tablets (Alliance Healthcare (Distribution) Ltd)	Diuretics
66195	Bumetanide 1mg tablets (Almus Pharmaceuticals Ltd)	Diuretics
31932	Bumetanide 1mg tablets (C P Pharmaceuticals Ltd)	Diuretics
73195	Bumetanide 1mg tablets (DE Pharmaceuticals)	Diuretics
36767	Bumetanide 1mg tablets (IVAX Pharmaceuticals UK Ltd)	Diuretics
34934	Bumetanide 1mg tablets (Mylan)	Diuretics
74814	Bumetanide 1mg tablets (Niche Generics Ltd)	Diuretics
45305	Bumetanide 1mg tablets (Teva UK Ltd)	Diuretics
73152	Bumetanide 1mg tablets (Waymade Healthcare Plc)	Diuretics
5218	Bumetanide 1mg/5ml oral solution sugar free	Diuretics
19300	Bumetanide 2mg/4ml solution for injection ampoules	Diuretics
6160	Bumetanide 500microgram / Potassium chloride 573mg (potassium 7.7mmol) modified-release tablets	Diuretics
7806	Bumetanide 5mg tablets	Diuretics
62024	Bumetanide 5mg tablets (A A H Pharmaceuticals Ltd)	Diuretics
34613	Bumetanide 5mg tablets (Teva UK Ltd)	Diuretics
15341	Burinex 0.5mg/ml Injection (LEO Pharma)	Diuretics
2788	Burinex 1mg tablets (LEO Pharma)	Diuretics
12226	Burinex 1mg/5ml Oral solution (LEO Pharma)	Diuretics
12294	Burinex 5mg tablets (LEO Pharma)	Diuretics
1776	Burinex K modified-release tablets (LEO Pharma)	Diuretics
2833	CYCLOPENTHIAZIDE -K tablets	Diuretics
23492	CYCLOPENTHIAZIDE 250MCG/K 8.1MMOL	Diuretics
62066	Cardide SR 1.5mg tablets (Teva UK Ltd)	Diuretics
18973	Centyl 2.5mg Tablet (Edwin Burgess Ltd)	Diuretics
29991	Centyl 5mg Tablet (Edwin Burgess Ltd)	Diuretics
20431	Centyl K modified-release tablets (LEO Pharma)	Diuretics
20426	Centyl k 2.5mg+7.7mmol Tablet (Edwin Burgess Ltd)	Diuretics
2979	Centyl k Tablet (Edwin Burgess Ltd)	Diuretics
74153	Chlorothiazide 10mg/5ml oral solution	Diuretics
64798	Chlorothiazide 120mg/5ml oral solution	Diuretics
13246	Chlorothiazide 150mg/5ml oral suspension	Diuretics
73441	Chlorothiazide 200mg/5ml oral solution	Diuretics
54679	Chlorothiazide 250mg tablets	Diuretics
59834	Chlorothiazide 250mg/5ml oral solution	Diuretics
6816	Chlorothiazide 250mg/5ml oral suspension	Diuretics
56804	Chlorothiazide 25mg/5ml oral suspension	Diuretics
8836	Chlorothiazide 500mg tablets	Diuretics
60603	Chlorothiazide 50mg/5ml oral suspension	Diuretics
54341	Chlorothiazide 5mg/5ml oral suspension	Diuretics
71871	Chlorothiazide 60mg/5ml oral suspension	Diuretics

63227	Chlorothiazide 70mg/5ml oral solution	Diuretics
55889	Chlorothiazide oral solution	Diuretics
3548	Chlortalidone 100mg tablets	Diuretics
605	Chlortalidone 50mg tablets	Diuretics
74942	Chlortalidone 50mg/5ml oral suspension	Diuretics
1170	Cyclopenthiazide 500microgram tablets	Diuretics
27556	DYAZIDE	Diuretics
25494	Diatensec 50mg Tablet (Pharmacia Ltd)	Diuretics
7734	Diumide-K Continus tablets (Teofarma)	Diuretics
26292	Diuresal 40mg Tablet (Lagap)	Diuretics
4044	Diurexan 20mg tablets (Meda Pharmaceuticals Ltd)	Diuretics
33724	Diuril 250mg/5ml oral suspension (Imported (United States))	Diuretics
18716	Dryptal 10mg/ml Injection (Berk Pharmaceuticals Ltd)	Diuretics
21849	Dryptal 40mg Tablet (Berk Pharmaceuticals Ltd)	Diuretics
4068	Dytac 50mg capsules (AMCo)	Diuretics
13472	ESIDREX-K TAB	Diuretics
18267	Enduron 5mg Tablet (Abbott Laboratories Ltd)	Diuretics
10251	Eplerenone 25mg tablets	Diuretics
70370	Eplerenone 25mg tablets (Actavis UK Ltd)	Diuretics
70918	Eplerenone 25mg tablets (Alliance Healthcare (Distribution) Ltd)	Diuretics
16531	Eplerenone 50mg tablets	Diuretics
66011	Eplerenone 50mg tablets (A A H Pharmaceuticals Ltd)	Diuretics
70543	Eplerenone 50mg tablets (Actavis UK Ltd)	Diuretics
36519	Esidrex -k Tablet (Novartis Pharmaceuticals UK Ltd)	Diuretics
17252	Esidrex 25mg Tablet (Novartis Pharmaceuticals UK Ltd)	Diuretics
13363	Esidrex 50mg Tablet (Novartis Pharmaceuticals UK Ltd)	Diuretics
41885	Ethibide XL 1.5mg tablets (Genus Pharmaceuticals Ltd)	Diuretics
20785	FRUMIL	Diuretics
16206	Froop 40mg tablets (Ashbourne Pharmaceuticals Ltd)	Diuretics
20538	Frumax 40mg Tablet (Ashbourne Pharmaceuticals Ltd)	Diuretics
14761	Frusid 40mg tablets (Dr Reddy's Laboratories (UK) Ltd)	Diuretics
5868	Frusol 20mg/5ml oral solution (Rosemont Pharmaceuticals Ltd)	Diuretics
9680	Frusol 40mg/5ml oral solution (Rosemont Pharmaceuticals Ltd)	Diuretics
14837	Frusol 50mg/5ml oral solution (Rosemont Pharmaceuticals Ltd)	Diuretics
58224	Furosemide 10mg/5ml oral solution	Diuretics
562	Furosemide 10mg/ml Injection	Diuretics
46116	Furosemide 10mg/ml Injection (Antigen Pharmaceuticals)	Diuretics
40247	Furosemide 10mg/ml Injection (Martindale Pharmaceuticals Ltd)	Diuretics
71406	Furosemide 1mg/5ml oral solution	Diuretics
3287	Furosemide 1mg/ml Oral solution	Diuretics
17960	Furosemide 20mg / Potassium chloride 750mg (potassium 10mmol) modified-release tablets	Diuretics
29780	Furosemide 20mg Tablet (C P Pharmaceuticals Ltd)	Diuretics
47815	Furosemide 20mg Tablet (Celltech Pharma Europe Ltd)	Diuretics
55	Furosemide 20mg tablets	Diuretics
30625	Furosemide 20mg tablets (A A H Pharmaceuticals Ltd)	Diuretics
31548	Furosemide 20mg tablets (Actavis UK Ltd)	Diuretics

Carr MJ, *et al. BMJ Qual Saf* 2022; 31:503–514. doi: 10.1136/bmjqs-2021-013613

59290	Furosemide 20mg tablets (Alliance Healthcare (Distribution) Ltd)	Diuretics
66017	Furosemide 20mg tablets (Almus Pharmaceuticals Ltd)	Diuretics
63237	Furosemide 20mg tablets (Boston Healthcare Ltd)	Diuretics
53967	Furosemide 20mg tablets (Bristol Laboratories Ltd)	Diuretics
74926	Furosemide 20mg tablets (Crescent Pharma Ltd)	Diuretics
61475	Furosemide 20mg tablets (DE Pharmaceuticals)	Diuretics
56051	Furosemide 20mg tablets (Kent Pharmaceuticals Ltd)	Diuretics
27926	Furosemide 20mg tablets (Mylan)	Diuretics
59884	Furosemide 20mg tablets (Phoenix Healthcare Distribution Ltd)	Diuretics
32918	Furosemide 20mg tablets (Sandoz Ltd)	Diuretics
54825	Furosemide 20mg tablets (Sigma Pharmaceuticals Plc)	Diuretics
19194	Furosemide 20mg tablets (Teva UK Ltd)	Diuretics
71950	Furosemide 20mg tablets (Waymade Healthcare Plc)	Diuretics
41292	Furosemide 20mg tablets (Wockhardt UK Ltd)	Diuretics
4705	Furosemide 20mg/2ml Injection	Diuretics
35162	Furosemide 20mg/2ml solution for injection ampoules	Diuretics
52887	Furosemide 20mg/2ml solution for injection ampoules (A A H Pharmaceuticals Ltd)	Diuretics
57600	Furosemide 20mg/2ml solution for injection ampoules (Alliance Healthcare (Distribution) Ltd)	Diuretics
59030	Furosemide 20mg/5ml oral solution	Diuretics
6118	Furosemide 20mg/5ml oral solution sugar free	Diuretics
57610	Furosemide 20mg/5ml oral solution sugar free (Focus Pharmaceuticals Ltd)	Diuretics
59939	Furosemide 20mg/5ml oral suspension	Diuretics
73480	Furosemide 20mg/5ml sugar free Oral solution (Rosemont Pharmaceuticals Ltd)	Diuretics
30875	Furosemide 250mg/25ml solution for injection ampoules	Diuretics
52045	Furosemide 250mg/5ml solution for injection vials	Diuretics
64255	Furosemide 2mg/5ml oral solution	Diuretics
65583	Furosemide 3mg/5ml oral solution	Diuretics
74381	Furosemide 3mg/5ml oral suspension	Diuretics
66149	Furosemide 4.5mg/5ml oral solution	Diuretics
8102	Furosemide 40mg / Potassium chloride 600mg (potassium 8mmol) modified-release tablets	Diuretics
19192	Furosemide 40mg Tablet (M & A Pharmachem Ltd)	Diuretics
6	Furosemide 40mg tablets	Diuretics
27690	Furosemide 40mg tablets (A A H Pharmaceuticals Ltd)	Diuretics
60291	Furosemide 40mg tablets (AMCo)	Diuretics
56375	Furosemide 40mg tablets (Accord Healthcare Ltd)	Diuretics
34006	Furosemide 40mg tablets (Actavis UK Ltd)	Diuretics
59911	Furosemide 40mg tablets (Alliance Healthcare (Distribution) Ltd)	Diuretics
46699	Furosemide 40mg tablets (Almus Pharmaceuticals Ltd)	Diuretics
46948	Furosemide 40mg tablets (Arrow Generics Ltd)	Diuretics
64677	Furosemide 40mg tablets (DE Pharmaceuticals)	Diuretics
34557	Furosemide 40mg tablets (IVAX Pharmaceuticals UK Ltd)	Diuretics
27696	Furosemide 40mg tablets (Kent Pharmaceuticals Ltd)	Diuretics
74677	Furosemide 40mg tablets (Mawdsley-Brooks & Company Ltd)	Diuretics

25717	Furosemide 40mg tablets (Mylan)	Diuretics
32896	Furosemide 40mg tablets (Ranbaxy (UK) Ltd)	Diuretics
34374	Furosemide 40mg tablets (Teva UK Ltd)	Diuretics
27447	Furosemide 40mg tablets (Wockhardt UK Ltd)	Diuretics
67910	Furosemide 40mg/5ml oral solution	Diuretics
5728	Furosemide 40mg/5ml oral solution sugar free	Diuretics
42488	Furosemide 40mg/5ml oral solution sugar free (A A H Pharmaceuticals Ltd)	Diuretics
42388	Furosemide 40mg/5ml oral solution sugar free (Focus Pharmaceuticals Ltd)	Diuretics
69445	Furosemide 40mg/5ml oral solution sugar free (Sigma Pharmaceuticals Plc)	Diuretics
61365	Furosemide 40mg/5ml oral suspension	Diuretics
69338	Furosemide 40mg/5ml sugar free Oral solution (Rosemont Pharmaceuticals Ltd)	Diuretics
68068	Furosemide 4mg/5ml oral solution	Diuretics
3248	Furosemide 500mg tablets	Diuretics
25334	Furosemide 500mg tablets (A A H Pharmaceuticals Ltd)	Diuretics
41828	Furosemide 500mg tablets (Actavis UK Ltd)	Diuretics
41405	Furosemide 500mg tablets (Teva UK Ltd)	Diuretics
5249	Furosemide 50mg/5ml oral solution sugar free	Diuretics
49268	Furosemide 50mg/5ml oral suspension	Diuretics
19258	Furosemide 50mg/5ml solution for injection ampoules	Diuretics
55738	Furosemide 50mg/5ml solution for injection ampoules (Hameln Pharmaceuticals Ltd)	Diuretics
70650	Furosemide 50mg/5ml solution for injection ampoules (Peckforton Pharmaceuticals Ltd)	Diuretics
19056	Furosemide 50mg/5ml sugar free Oral solution (Rosemont Pharmaceuticals Ltd)	Diuretics
60465	Furosemide 5mg/5ml oral solution	Diuretics
36190	Furosemide 5mg/5ml oral solution sugar free	Diuretics
51983	Furosemide 5mg/5ml oral suspension	Diuretics
52900	Furosemide 80mg/8ml solution for injection Minijet pre-filled syringes (UCB Pharma Ltd)	Diuretics
32277	Furosemide 80mg/8ml solution for injection pre-filled syringes	Diuretics
58078	Furosemide 8mg/5ml oral solution	Diuretics
64745	Furosemide 8mg/5ml oral suspension	Diuretics
73171	Furosemide Oral solution	Diuretics
23483	HYDROCHLOROTHIAZIDE 12.5MG/K 8.1MMOL S/R	Diuretics
15577	HYDROCHLOROTHIAZIDE 12.5MG/K 8.1MMOL S/R 12.5 MG TAB	Diuretics
13525	Hydrenox 50mg Tablet (Knoll Ltd)	Diuretics
62516	Hydrochlorothiazide 12.5mg tablets	Diuretics
542	Hydrochlorothiazide 25mg tablets	Diuretics
3517	Hydrochlorothiazide 50mg tablets	Diuretics
48132	Hydrochlorothiazide Capsule	Diuretics
57488	Hydrochlorothiazide Oral solution	Diuretics
12110	Hydroflumethiazide 50mg Tablet	Diuretics
12440	Hydrosaluric 25mg tablets (Merck Sharp & Dohme Ltd)	Diuretics
16632	Hydrosaluric 50mg tablets (Merck Sharp & Dohme Ltd)	Diuretics
8891	Hygroton -k Tablet (Novartis Pharmaceuticals UK Ltd)	Diuretics
3054	Hygroton 100mg Tablet (Alliance Pharmaceuticals Ltd)	Diuretics

3997	Hygroton 50mg tablets (Alliance Pharmaceuticals Ltd)	Diuretics
5112	Indapamide 1.5mg modified-release tablets	Diuretics
46675	Indapamide 1.5mg modified-release tablets (A A H Pharmaceuticals Ltd)	Diuretics
62771	Indapamide 1.5mg modified-release tablets (DE Pharmaceuticals)	Diuretics
74172	Indapamide 1.5mg modified-release tablets (Sigma Pharmaceuticals Plc)	Diuretics
60020	Indapamide 1.5mg modified-release tablets (Waymade Healthcare Plc)	Diuretics
2612	Indapamide 2.5mg tablets	Diuretics
48099	Indapamide 2.5mg tablets (A A H Pharmaceuticals Ltd)	Diuretics
43516	Indapamide 2.5mg tablets (Actavis UK Ltd)	Diuretics
54316	Indapamide 2.5mg tablets (Alliance Healthcare (Distribution) Ltd)	Diuretics
56296	Indapamide 2.5mg tablets (Boston Healthcare Ltd)	Diuretics
70509	Indapamide 2.5mg tablets (DE Pharmaceuticals)	Diuretics
40907	Indapamide 2.5mg tablets (Genus Pharmaceuticals Ltd)	Diuretics
55259	Indapamide 2.5mg tablets (Kent Pharmaceuticals Ltd)	Diuretics
34551	Indapamide 2.5mg tablets (Mylan)	Diuretics
42906	Indapamide 2.5mg tablets (Niche Generics Ltd)	Diuretics
49529	Indapamide 2.5mg tablets (Phoenix Healthcare Distribution Ltd)	Diuretics
56760	Indapamide 2.5mg tablets (Strides Shasun (UK) Ltd)	Diuretics
33083	Indapamide 2.5mg tablets (Teva UK Ltd)	Diuretics
48079	Indapamide 2.5mg tablets (Zentiva)	Diuretics
64066	Indapamide 2.5mg/5ml oral suspension	Diuretics
44168	Indipam XL 1.5mg tablets (Actavis UK Ltd)	Diuretics
14144	Inspra 25mg tablets (Pfizer Ltd)	Diuretics
29694	Inspra 50mg tablets (Pfizer Ltd)	Diuretics
26328	LASIX (25ML)	Diuretics
22539	LASIX (2ML)	Diuretics
20513	LASIX 10 MG INJ	Diuretics
23256	LASIX PAED	Diuretics
7582	Lasikal modified-release tablets (Borg Medicare)	Diuretics
7799	Lasix 20mg tablets (Borg Medicare)	Diuretics
4258	Lasix 20mg/2ml solution for injection ampoules (Sanofi)	Diuretics
12318	Lasix 250mg/25ml Injection (Hoechst Marion Roussel)	Diuretics
7606	Lasix 40mg tablets (Sanofi)	Diuretics
10392	Lasix 500mg tablets (Sanofi)	Diuretics
10422	Lasix 50mg/5ml Injection (Hoechst UK Ltd)	Diuretics
4182	Lasix 5mg/5ml oral solution (Borg Medicare)	Diuretics
10781	Lasix with k Tablet (Hoechst Marion Roussel)	Diuretics
20779	MODURETIC	Diuretics
43184	Mapemid XL 1.5mg tablets (Teva UK Ltd)	Diuretics
8464	Meprobamate with bendroflumethiazide Tablet	Diuretics
8602	Metenix 5mg tablets (Sanofi)	Diuretics
20057	Methyclothiazide 5mg Tablet	Diuretics
53674	Metolazone 2.5mg tablets	Diuretics
49752	Metolazone 2.5mg/5ml oral solution	Diuretics
68432	Metolazone 2.5mg/5ml oral suspension	Diuretics
4334	Metolazone 500microgram low dose Tablet	Diuretics
4332	Metolazone 5mg tablets	Diuretics

Carr MJ, *et al. BMJ Qual Saf* 2022; 31:503–514. doi: 10.1136/bmjqs-2021-013613

55777	Metolazone 5mg/5ml oral solution	Diuretics
54329	Metolazone 5mg/5ml oral suspension	Diuretics
54643	Metolazone Oral solution	Diuretics
13352	Midamor 5mg Tablet (MSD Thomas Morson Pharmaceuticals)	Diuretics
24835	Min-i-jet furosemide 10mg/ml Injection (Celltech Pharma Europe Ltd)	Diuretics
27546	NATRILIX	Diuretics
15602	NATRILIX 5 MG TAB	Diuretics
22242	NAVIDREX	Diuretics
27957	Natramid 2.5mg Tablet (Trinity Pharmaceuticals Ltd)	Diuretics
7641	Natrilix 2.5mg tablets (Servier Laboratories Ltd)	Diuretics
3056	Natrilix SR 1.5mg tablets (Servier Laboratories Ltd)	Diuretics
1125	Navidrex -k Tablet (Novartis Pharmaceuticals UK Ltd)	Diuretics
2046	Navidrex 500microgram tablets (AMCo)	Diuretics
46302	Neo-Naclex 2.5mg tablets (AMCo)	Diuretics
1209	Neo-Naclex 5mg tablets (Mercury Pharma Group Ltd)	Diuretics
1213	Neo-Naclex-K modified-release tablets (Mercury Pharma Group Ltd)	Diuretics
24189	Neo-bendromax 2.5mg Tablet (Ashbourne Pharmaceuticals Ltd)	Diuretics
24190	Neo-bendromax 5mg Tablet (Ashbourne Pharmaceuticals Ltd)	Diuretics
12360	Nephril 1mg Tablet (Pfizer Ltd)	Diuretics
26275	Nindaxa 2.5 tablets (Ashbourne Pharmaceuticals Ltd)	Diuretics
26256	Opumide 2.5mg Tablet (Opus Pharmaceuticals Ltd)	Diuretics
12367	Piretanide 6mg capsule	Diuretics
12926	Polythiazide 1mg tablets	Diuretics
59616	Rawel XL 1.5mg tablets (Consilient Health Ltd)	Diuretics
25086	SPIRONOLACTONE	Diuretics
15053	SPIRONOLACTONE 10 MG/5ML LIQ	Diuretics
17720	Saluric 500mg Tablet (Merck Sharp & Dohme Ltd)	Diuretics
29397	Spiretic 100mg Tablet (DDSA Pharmaceuticals Ltd)	Diuretics
47687	Spiretic 25mg Tablet (DDSA Pharmaceuticals Ltd)	Diuretics
7991	Spiroctan 100mg Capsule (Roche Products Ltd)	Diuretics
4161	Spiroctan 25mg Tablet (Roche Products Ltd)	Diuretics
15052	Spiroctan 50mg Tablet (Roche Products Ltd)	Diuretics
17902	Spirolone 100mg Tablet (Berk Pharmaceuticals Ltd)	Diuretics
11156	Spirolone 25mg Tablet (Berk Pharmaceuticals Ltd)	Diuretics
17950	Spirolone 50mg Tablet (Berk Pharmaceuticals Ltd)	Diuretics
787	Spironolactone 100mg capsule	Diuretics
2142	Spironolactone 100mg tablets	Diuretics
31219	Spironolactone 100mg tablets (A A H Pharmaceuticals Ltd)	Diuretics
41592	Spironolactone 100mg tablets (Actavis UK Ltd)	Diuretics
41660	Spironolactone 100mg tablets (Teva UK Ltd)	Diuretics
56536	Spironolactone 100mg/5ml oral solution	Diuretics
14109	Spironolactone 100mg/5ml oral solution sugar free	Diuretics
49388	Spironolactone 100mg/5ml oral suspension	Diuretics
52970	Spironolactone 10mg/5ml oral solution	Diuretics
50079	Spironolactone 10mg/5ml oral suspension	Diuretics
12946	Spironolactone 10mg/5ml oral suspension sugar free	Diuretics
71398	Spironolactone 12.5mg/5ml oral solution	Diuretics

65822	Spironolactone 12.5mg/5ml oral suspension	Diuretics
57556	Spironolactone 12mg/5ml oral solution	Diuretics
13264	Spironolactone 15mg/5ml oral suspension	Diuretics
58225	Spironolactone 2.5mg/5ml oral suspension	Diuretics
57104	Spironolactone 200mg/5ml oral suspension	Diuretics
61025	Spironolactone 20mg/5ml oral solution	Diuretics
58757	Spironolactone 20mg/5ml oral suspension	Diuretics
35789	Spironolactone 25mg Tablet (Celltech Pharma Europe Ltd)	Diuretics
692	Spironolactone 25mg tablets	Diuretics
34296	Spironolactone 25mg tablets (A A H Pharmaceuticals Ltd)	Diuretics
34347	Spironolactone 25mg tablets (Actavis UK Ltd)	Diuretics
41074	Spironolactone 25mg tablets (Almus Pharmaceuticals Ltd)	Diuretics
51652	Spironolactone 25mg tablets (DE Pharmaceuticals)	Diuretics
73644	Spironolactone 25mg tablets (Dr Reddy's Laboratories (UK) Ltd)	Diuretics
71010	Spironolactone 25mg tablets (Genesis Pharmaceuticals Ltd)	Diuretics
34908	Spironolactone 25mg tablets (IVAX Pharmaceuticals UK Ltd)	Diuretics
60343	Spironolactone 25mg tablets (Kent Pharmaceuticals Ltd)	Diuretics
31529	Spironolactone 25mg tablets (Teva UK Ltd)	Diuretics
45078	Spironolactone 25mg/5ml Oral solution sugar free (Rosemont Pharmaceuticals Ltd)	Diuretics
51720	Spironolactone 25mg/5ml oral solution	Diuretics
47018	Spironolactone 25mg/5ml oral suspension	Diuretics
74285	Spironolactone 25mg/5ml oral suspension (Drug Tariff Special Order)	Diuretics
11519	Spironolactone 25mg/5ml oral suspension sugar free	Diuretics
74154	Spironolactone 2mg/5ml oral suspension	Diuretics
69473	Spironolactone 3.5mg/5ml oral suspension	Diuretics
65582	Spironolactone 3mg/5ml oral solution	Diuretics
60660	Spironolactone 3mg/5ml oral suspension	Diuretics
56274	Spironolactone 4.5mg/5ml oral suspension	Diuretics
57933	Spironolactone 40mg/5ml oral suspension	Diuretics
56067	Spironolactone 4mg/5ml oral solution	Diuretics
54120	Spironolactone 4mg/5ml oral suspension	Diuretics
19195	Spironolactone 50mg Tablet (Wyeth Pharmaceuticals)	Diuretics
708	Spironolactone 50mg tablets	Diuretics
43514	Spironolactone 50mg tablets (A A H Pharmaceuticals Ltd)	Diuretics
74631	Spironolactone 50mg tablets (Actavis UK Ltd)	Diuretics
41706	Spironolactone 50mg tablets (IVAX Pharmaceuticals UK Ltd)	Diuretics
67913	Spironolactone 50mg tablets (Kent Pharmaceuticals Ltd)	Diuretics
32837	Spironolactone 50mg tablets (Teva UK Ltd)	Diuretics
46674	Spironolactone 50mg/5ml Oral suspension sugar free (Rosemont Pharmaceuticals Ltd)	Diuretics
51933	Spironolactone 50mg/5ml oral solution	Diuretics
46990	Spironolactone 50mg/5ml oral suspension	Diuretics
53253	Spironolactone 50mg/5ml oral suspension (Drug Tariff Special Order)	Diuretics
6815	Spironolactone 50mg/5ml oral suspension sugar free	Diuretics
52366	Spironolactone 5mg/5ml oral solution	Diuretics
50370	Spironolactone 5mg/5ml oral suspension	Diuretics

10214	Spironolactone 5mg/5ml oral suspension sugar free	Diuretics
63309	Spironolactone 6mg/5ml oral suspension	Diuretics
73208	Spironolactone 7mg/5ml oral suspension	Diuretics
58077	Spironolactone 8mg/5ml oral suspension	Diuretics
23091	Spirospare 100 tablets (Ashbourne Pharmaceuticals Ltd)	Diuretics
21911	Spirospare 25mg Tablet (Ashbourne Pharmaceuticals Ltd)	Diuretics
8303	Tenavoid Tablet (Edwin Burgess Ltd)	Diuretics
41861	Tensaid XL 1.5mg tablets (Mylan)	Diuretics
18096	Torasemide 10mg tablets	Diuretics
11487	Torasemide 2.5mg tablets	Diuretics
8052	Torasemide 5mg tablets	Diuretics
40898	Torasemide 5mg tablets (A A H Pharmaceuticals Ltd)	Diuretics
46525	Torasemide 5mg tablets (Teva UK Ltd)	Diuretics
40190	Torasemide iv 20mg/4ml Intravenous injection	Diuretics
22658	Torem 10mg tablets (Meda Pharmaceuticals Ltd)	Diuretics
11268	Torem 2.5mg tablets (Meda Pharmaceuticals Ltd)	Diuretics
10066	Torem 5mg tablets (Meda Pharmaceuticals Ltd)	Diuretics
40738	Torem iv 10mg/2ml Intravenous injection (Boehringer Mannheim UK Ltd)	Diuretics
2179	Triamterene 50mg capsules	Diuretics
39447	Varbim XL 1.5mg tablets (Teva UK Ltd)	Diuretics
26675	XIPAMIDE	Diuretics
7618	Xipamide 20mg tablets	Diuretics
19352	Xuret 0.5mg Tablet (Galen Ltd)	Diuretics
61846	Zaroxolyn 2.5mg tablets (IDIS)	Diuretics
69009	Zaroxolyn 5mg tablets (Imported (Canada))	Diuretics
7923	Guanethidine 10mg Tablet	Peripheral adrenergic inhibitor
31080	Guanethidine 10mg/1ml solution for injection ampoules	Peripheral adrenergic inhibitor
17291	Guanethidine 25mg Tablet	Peripheral adrenergic inhibitor
7922	Ismelin 10mg Tablet (Sovereign Medical Ltd)	Peripheral adrenergic inhibitor
48189	Ismelin 10mg/1ml solution for injection ampoules (Amdipharm Plc)	Peripheral adrenergic inhibitor
30127	Ismelin 10mg/ml Injection (Sovereign Medical Ltd)	Peripheral adrenergic inhibitor
13379	Ismelin 25mg Tablet (Sovereign Medical Ltd)	Peripheral adrenergic inhibitor
13317	Apresoline 20mg powder for solution for injection ampoules (AMCo)	Vasodilators
2362	Apresoline 25mg tablets (AMCo)	Vasodilators
2680	Apresoline 50mg Tablet (Sovereign Medical Ltd)	Vasodilators
25506	DIAZOXIDE 15 MG INJ	Vasodilators
54340	Diazoxide 10mg/5ml oral solution	Vasodilators
74301	Diazoxide 125mg/5ml oral solution	Vasodilators
74299	Diazoxide 125mg/5ml oral suspension	Vasodilators
42677	Diazoxide 250mg/5ml oral solution	Vasodilators
39956	Diazoxide 250mg/5ml oral suspension	Vasodilators

74300	Diazoxide 25mg capsules	Vasodilators
55989	Diazoxide 25mg/5ml oral solution	Vasodilators
32267	Diazoxide 300mg/20ml solution for injection ampoules	Vasodilators
6265	Diazoxide 50mg tablets	Vasodilators
51018	Diazoxide 50mg/5ml oral solution	Vasodilators
35960	Diazoxide 50mg/5ml oral suspension	Vasodilators
31417	Diazoxide 50mg/ml oral solution	Vasodilators
25887	Eudemine 50mg tablets (RPH Pharmaceuticals AB)	Vasodilators
214	HYDRALAZINE 1 MG SYR	Vasodilators
31971	HYDRALAZINE 6.25 MG SYR	Vasodilators
23746	HYDRALAZINE HCl 10 MG TAB	Vasodilators
21749	HYDRALAZINE HCl 100 MG TAB	Vasodilators
4507	HYDRALAZINE HCl 12.5 MG TAB	Vasodilators
74749	Hydralazine 10mg tablets	Vasodilators
18861	Hydralazine 10mg/5ml oral suspension	Vasodilators
71256	Hydralazine 20mg powder for concentrate for solution for injection ampoules (AMCo)	Vasodilators
504	Hydralazine 20mg powder for solution for injection ampoules	Vasodilators
573	Hydralazine 25mg tablets	Vasodilators
31220	Hydralazine 25mg tablets (A A H Pharmaceuticals Ltd)	Vasodilators
43500	Hydralazine 25mg tablets (Actavis UK Ltd)	Vasodilators
70655	Hydralazine 25mg/5ml oral suspension	Vasodilators
1296	Hydralazine 50mg tablets	Vasodilators
41639	Hydralazine 50mg tablets (Actavis UK Ltd)	Vasodilators
71097	Hydralazine 50mg tablets (Almus Pharmaceuticals Ltd)	Vasodilators
59512	Hydralazine 50mg/5ml oral solution	Vasodilators
61116	Hydralazine 50mg/5ml oral suspension	Vasodilators
64253	Hydralazine 5mg/5ml oral suspension	Vasodilators
63652	Hydralazine Tablet	Vasodilators
14495	Loniten 10mg tablets (Pfizer Ltd)	Vasodilators
9697	Loniten 2.5mg tablets (Pfizer Ltd)	Vasodilators
9463	Loniten 5mg tablets (Pfizer Ltd)	Vasodilators
18938	MINOXIDIL 1 % TAB	Vasodilators
2968	Minoxidil 10mg tablets	Vasodilators
2970	Minoxidil 2.5mg tablets	Vasodilators
2967	Minoxidil 5mg tablets	Vasodilators
65386	Proglycem 250mg/5ml oral suspension (Imported (United States))	Vasodilators
36612	Sodium nitroprusside 50mg powder for solution for infusion vials	Vasodilators

Carr MJ, *et al. BMJ Qual Saf* 2022; 31:503–514. doi: 10.1136/bmjqs-2021-013613

Appendix 21. Product codes for prescriptions of lipid-lowering medications

Product code	Term	Drug class
653	Ezetimibe 10mg tablets	Ezetimibe
74161	Ezetimibe 10mg tablets (Mylan)	Ezetimibe
6120	Ezetrol 10mg tablets (Merck Sharp & Dohme Ltd)	Ezetimibe
25018	BEZAFIBRATE	Fibrate
184	Bezafibrate 200mg tablets	Fibrate
29328	Bezafibrate 200mg tablets (A A H Pharmaceuticals Ltd)	Fibrate
31221	Bezafibrate 200mg tablets (Mylan)	Fibrate
33944	Bezafibrate 200mg tablets (Teva UK Ltd)	Fibrate
34181	Bezafibrate 400mg Modified-release tablet (Hillcross Pharmaceuticals Ltd)	Fibrate
49609	Bezafibrate 400mg Modified-release tablet (Sandoz Ltd)	Fibrate
602	Bezafibrate 400mg modified-release tablets	Fibrate
66425	Bezafibrate 400mg modified-release tablets (A A H Pharmaceuticals Ltd)	Fibrate
52814	Bezafibrate 400mg modified-release tablets (Alliance Healthcare (Distribution) Ltd)	Fibrate
59002	Bezafibrate 400mg modified-release tablets (DE Pharmaceuticals)	Fibrate
66564	Bezafibrate 400mg modified-release tablets (Phoenix Healthcare Distribution Ltd)	Fibrate
42801	Bezafibrate xl 400mg Modified-release tablet (Generics (UK) Ltd)	Fibrate
29213	Bezagen XL 400mg tablets (Mylan)	Fibrate
1324	Bezalip 200mg Tablet (Roche Products Ltd)	Fibrate
39576	Bezalip 200mg tablets (Teva UK Ltd)	Fibrate
1214	Bezalip 400mg Tablet (Roche Products Ltd)	Fibrate
58635	Bezalip Mono 400mg modified-release tablets (DE Pharmaceuticals)	Fibrate
74009	Bezalip Mono 400mg modified-release tablets (Dowelhurst Ltd)	Fibrate
60385	Bezalip Mono 400mg modified-release tablets (Lexon (UK) Ltd)	Fibrate
70751	Bezalip Mono 400mg modified-release tablets (Sigma Pharmaceuticals Plc)	Fibrate
39420	Bezalip Mono 400mg modified-release tablets (Teva UK Ltd)	Fibrate
64503	Bezalip Mono 400mg modified-release tablets (Waymade Healthcare Plc)	Fibrate
5216	Bezalip mono 400mg Modified-release tablet (Roche Products Ltd)	Fibrate
3089	Ciprofibrate 100mg tablets	Fibrate
71016	Ciprofibrate 100mg tablets (DE Pharmaceuticals)	Fibrate
57489	Ciprofibrate 100mg tablets (Zentiva)	Fibrate
1215	Fenofibrate 100mg Capsule	Fibrate
50071	Fenofibrate 160mg Tablet (Teva UK Ltd)	Fibrate
47935	Fenofibrate 200mg Capsule (Teva UK Ltd)	Fibrate
3159	Fenofibrate 200mg capsules	Fibrate
9639	Fenofibrate micronised 160mg tablets	Fibrate
64984	Fenofibrate micronised 160mg tablets (Phoenix Healthcare Distribution Ltd)	Fibrate
65572	Fenofibrate micronised 160mg tablets (Thornton & Ross Ltd)	Fibrate
72092	Fenofibrate micronised 160mg tablets (Waymade Healthcare Plc)	Fibrate
4920	Fenofibrate micronised 200mg capsules	Fibrate
41396	Fenofibrate micronised 200mg capsules (A A H Pharmaceuticals Ltd)	Fibrate
73384	Fenofibrate micronised 200mg capsules (DE Pharmaceuticals)	Fibrate
73385	Fenofibrate micronised 200mg capsules (Kent Pharmaceuticals Ltd)	Fibrate
67157	Fenofibrate micronised 200mg capsules (Phoenix Healthcare Distribution Ltd)	Fibrate

57219	Fenofibrate micronised 200mg capsules (Sandoz Ltd)	Fibrate
70950	Fenofibrate micronised 200mg capsules (Waymade Healthcare Plc)	Fibrate
5390	Fenofibrate micronised 267mg capsules	Fibrate
73491	Fenofibrate micronised 267mg capsules (A A H Pharmaceuticals Ltd)	Fibrate
64933	Fenofibrate micronised 267mg capsules (Ranbaxy (UK) Ltd)	Fibrate
63737	Fenofibrate micronised 267mg capsules (Sigma Pharmaceuticals Plc)	Fibrate
60788	Fenofibrate micronised 267mg capsules (Zentiva)	Fibrate
9491	Fenofibrate micronised 67mg capsules	Fibrate
31783	Fenogal 200mg capsules (Genus Pharmaceuticals Ltd)	Fibrate
33603	Fibrazate XL 400mg tablets (Sandoz Ltd)	Fibrate
23634	GEMFIBROZIL	Fibrate
3318	Gemfibrozil 300mg capsules	Fibrate
8082	Gemfibrozil 600mg tablets	Fibrate
34277	Gemfibrozil 600mg tablets (Teva UK Ltd)	Fibrate
2435	Lipantil 100mg Capsule (Fournier Pharmaceuticals Ltd)	Fibrate
4928	Lipantil Micro 200 capsules (Mylan)	Fibrate
74825	Lipantil Micro 200 capsules (Waymade Healthcare Plc)	Fibrate
67329	Lipantil Micro 267 capsules (DE Pharmaceuticals)	Fibrate
7540	Lipantil Micro 267 capsules (Mylan)	Fibrate
14379	Lipantil Micro 67 capsules (Mylan)	Fibrate
1217	Lipantil micro 200 200mg Capsule (Fournier Pharmaceuticals Ltd)	Fibrate
23153	Liparol 400 XL tablets (Ashbourne Pharmaceuticals Ltd)	Fibrate
2215	Lopid 300mg capsules (Pfizer Ltd)	Fibrate
4062	Lopid 600mg tablets (Pfizer Ltd)	Fibrate
53250	Modalim 100mg tablets (Lexon (UK) Ltd)	Fibrate
66087	Modalim 100mg tablets (Mawdsley-Brooks & Company Ltd)	Fibrate
8706	Modalim 100mg tablets (Sanofi)	Fibrate
9716	Supralip 160mg tablets (Mylan)	Fibrate
17614	Zimbacol XL 400mg tablets (Archimedes Pharma UK Ltd)	Fibrate
8104	Acipimox 250mg capsules	Other
71793	Alirocumab 150mg/1ml solution for injection pre-filled disposable devices	Other
70673	Alirocumab 75mg/1ml solution for injection pre-filled disposable devices	Other
8907	CHOLESTYRAMINE 325 MG CAP	Other
12231	CHOLESTYRAMINE POW	Other
20736	CHOLESTYRAMINE SACHETS	Other
26566	COLESTID SACHETS	Other
15594	COLESTIPOL HCl GRA	Other
37953	Cholestagel 625mg tablets (Sanofi)	Other
37266	Colesevelam 625mg tablets	Other
6365	Colestid 5g granules sachets plain (Pfizer Ltd)	Other
18081	Colestid Orange 5g granules sachets (Pfizer Ltd)	Other
5564	Colestid Orange sachets (Pharmacia Ltd)	Other
73910	Colestipol 1g tablets	Other
1212	Colestipol 5g granules sachets sugar free	Other
19938	Colestipol with aspartame granules	Other
14037	Eicosapentaenoic acid 170mg / Docosahexaenoic acid 115mg capsules	Other
14209	Eicosapentaenoic acid 170mg/g / Docosahexaenoic acid 115mg/g oral liquid	Other

6024	Eicosapentaenoic acid 460mg / Docosahexaenoic acid 380mg capsules	Other
57583	Eicosapentaenoic acid 460mg / Docosahexaenoic acid 380mg capsules (Huxley Europe Ltd)	Other
70580	Evolocumab 140mg/1ml solution for injection pre-filled disposable devices	Other
74346	Evolocumab 140mg/1ml solution for injection pre-filled syringes	Other
72032	G & G Omega 3 1000mg softgels capsules (G & G Food Supplies Ltd)	Other
67883	Lomitapide 5mg capsules	Other
2662	MaxEPA 1g capsules (Seven Seas Ltd)	Other
74035	MaxEPA 1g capsules (Waymade Healthcare Plc)	Other
3204	MaxEPA liquid (Seven Seas Ltd)	Other
23956	Maxepa Liquid (Seven Seas Ltd)	Other
24009	NICOTINIC ACID 500 MG TAB	Other
61269	Nebbaro 1000mg capsules (Zentiva)	Other
7551	Niaspan 1g modified-release tablets (Abbott Laboratories Ltd)	Other
11976	Niaspan 500mg modified-release tablets (Abbott Laboratories Ltd)	Other
7544	Niaspan 750mg modified-release tablets (Abbott Laboratories Ltd)	Other
10094	Niaspan titration pack (Abbott Laboratories Ltd)	Other
17813	Nicotinic acid 100mg Tablet	Other
40885	Nicotinic acid 1g / Laropiprant 20mg modified-release tablets	Other
18126	Nicotinic acid 1g modified-release tablets	Other
17824	Nicotinic acid 25mg Tablet	Other
18098	Nicotinic acid 375mg + 500mg + 750mg Modified-release tablet	Other
48585	Nicotinic acid 500mg capsules	Other
14963	Nicotinic acid 500mg modified-release tablets	Other
12211	Nicotinic acid 50mg tablets	Other
24583	Nicotinic acid 750mg modified-release tablets	Other
4067	Olbetam 250mg capsules (Pfizer Ltd)	Other
57234	Omacor capsules (DE Pharmaceuticals)	Other
6572	Omacor capsules (Mylan)	Other
61523	Omega 3 1000mg capsules (Alissa Healthcare Research Ltd)	Other
58655	Omega 3-acid-ethyl esters 1000mg capsules (Glenmark Pharmaceuticals Europe Ltd)	Other
55971	Prestylon 1g capsules (Teva UK Ltd)	Other
19792	QUESTRAN SACHET	Other
1716	Questran 4g oral powder sachets (Bristol-Myers Squibb Pharmaceuticals Ltd)	Other
1764	Questran Light 4g oral powder sachets (Bristol-Myers Squibb Pharmaceuticals Ltd)	Other
61087	Questran Light 4g oral powder sachets (Mawdsley-Brooks & Company Ltd)	Other
70397	Repatha 140mg/1ml solution for injection pre-filled syringes (Amgen Ltd)	Other
74067	Repatha SureClick 140mg/1ml solution for injection pre-filled pens (Amgen Ltd)	Other
53823	Solgar Niacin 500mg capsules (Solgar Vitamin and Herb)	Other
56426	Teromeg 1000mg capsules (AMCo)	Other
40729	Tredaptive 1000mg/20mg modified-release tablets (Merck Sharp & Dohme Ltd)	Other
56097	Atorvastatin 10mg chewable tablets sugar free	Statin
47090	Atorvastatin 10mg chewable tablets sugar free	Statin
28	Atorvastatin 10mg tablets	Statin
51134	Atorvastatin 10mg tablets (A A H Pharmaceuticals Ltd)	Statin
55727	Atorvastatin 10mg tablets (Actavis UK Ltd)	Statin

63140	Atorvastatin 10mg tablets (Alliance Healthcare (Distribution) Ltd)	Statin
67846	Atorvastatin 10mg tablets (Almus Pharmaceuticals Ltd)	Statin
68023	Atorvastatin 10mg tablets (Aspire Pharma Ltd)	Statin
57348	Atorvastatin 10mg tablets (Consilient Health Ltd)	Statin
58834	Atorvastatin 10mg tablets (DE Pharmaceuticals)	Statin
67573	Atorvastatin 10mg tablets (DE Pharmaceuticals)	Statin
55032	Atorvastatin 10mg tablets (Dexcel-Pharma Ltd)	Statin
70987	Atorvastatin 10mg tablets (Dr Reddy's Laboratories (UK) Ltd)	Statin
68785	Atorvastatin 10mg tablets (Mylan)	Statin
54535	Atorvastatin 10mg tablets (Pfizer Ltd)	Statin
64825	Atorvastatin 10mg tablets (Phoenix Healthcare Distribution Ltd)	Statin
59357	Atorvastatin 10mg tablets (Ranbaxy (UK) Ltd)	Statin
58868	Atorvastatin 10mg tablets (Sigma Pharmaceuticals Plc)	Statin
70693	Atorvastatin 10mg tablets (Sigma Pharmaceuticals Plc)	Statin
59859	Atorvastatin 10mg tablets (Teva UK Ltd)	Statin
61149	Atorvastatin 10mg tablets (Waymade Healthcare Plc)	Statin
74518	Atorvastatin 10mg tablets (Wockhardt UK Ltd)	Statin
50236	Atorvastatin 10mg tablets (Zentiva)	Statin
48518	Atorvastatin 10mg/5ml oral solution	Statin
54992	Atorvastatin 10mg/5ml oral suspension	Statin
56165	Atorvastatin 20mg chewable tablets sugar free	Statin
47065	Atorvastatin 20mg chewable tablets sugar free	Statin
75	Atorvastatin 20mg tablets	Statin
49558	Atorvastatin 20mg tablets (A A H Pharmaceuticals Ltd)	Statin
52211	Atorvastatin 20mg tablets (Actavis UK Ltd)	Statin
58394	Atorvastatin 20mg tablets (Alliance Healthcare (Distribution) Ltd)	Statin
56564	Atorvastatin 20mg tablets (Almus Pharmaceuticals Ltd)	Statin
51359	Atorvastatin 20mg tablets (Arrow Generics Ltd)	Statin
52168	Atorvastatin 20mg tablets (Aspire Pharma Ltd)	Statin
72164	Atorvastatin 20mg tablets (Bristol Laboratories Ltd)	Statin
51622	Atorvastatin 20mg tablets (Consilient Health Ltd)	Statin
62429	Atorvastatin 20mg tablets (DE Pharmaceuticals)	Statin
62219	Atorvastatin 20mg tablets (DE Pharmaceuticals)	Statin
50790	Atorvastatin 20mg tablets (Dexcel-Pharma Ltd)	Statin
71017	Atorvastatin 20mg tablets (Dr Reddy's Laboratories (UK) Ltd)	Statin
68467	Atorvastatin 20mg tablets (Kent Pharmaceuticals Ltd)	Statin
68827	Atorvastatin 20mg tablets (Mylan)	Statin
50788	Atorvastatin 20mg tablets (Pfizer Ltd)	Statin
68048	Atorvastatin 20mg tablets (Phoenix Healthcare Distribution Ltd)	Statin
65193	Atorvastatin 20mg tablets (Ranbaxy (UK) Ltd)	Statin
56248	Atorvastatin 20mg tablets (Sigma Pharmaceuticals Plc)	Statin
58041	Atorvastatin 20mg tablets (Teva UK Ltd)	Statin
59272	Atorvastatin 20mg tablets (Waymade Healthcare Plc)	Statin
72213	Atorvastatin 20mg tablets (Wockhardt UK Ltd)	Statin
58110	Atorvastatin 20mg tablets (Zentiva)	Statin
64067	Atorvastatin 20mg/5ml oral solution	Statin
60464	Atorvastatin 20mg/5ml oral suspension	Statin

48973	Atorvastatin 30mg tablets	Statin
64702	Atorvastatin 30mg tablets (A A H Pharmaceuticals Ltd)	Statin
63469	Atorvastatin 30mg tablets (Consilient Health Ltd)	Statin
745	Atorvastatin 40mg tablets	Statin
52398	Atorvastatin 40mg tablets (A A H Pharmaceuticals Ltd)	Statin
53887	Atorvastatin 40mg tablets (Actavis UK Ltd)	Statin
49751	Atorvastatin 40mg tablets (Alliance Healthcare (Distribution) Ltd)	Statin
59446	Atorvastatin 40mg tablets (Almus Pharmaceuticals Ltd)	Statin
51200	Atorvastatin 40mg tablets (Arrow Generics Ltd)	Statin
52460	Atorvastatin 40mg tablets (Aspire Pharma Ltd)	Statin
51876	Atorvastatin 40mg tablets (Consilient Health Ltd)	Statin
57834	Atorvastatin 40mg tablets (DE Pharmaceuticals)	Statin
56841	Atorvastatin 40mg tablets (Dexcel-Pharma Ltd)	Statin
52397	Atorvastatin 40mg tablets (Dr Reddy's Laboratories (UK) Ltd)	Statin
67402	Atorvastatin 40mg tablets (Kent Pharmaceuticals Ltd)	Statin
69427	Atorvastatin 40mg tablets (Mylan)	Statin
50272	Atorvastatin 40mg tablets (Pfizer Ltd)	Statin
64810	Atorvastatin 40mg tablets (Phoenix Healthcare Distribution Ltd)	Statin
60511	Atorvastatin 40mg tablets (Ranbaxy (UK) Ltd)	Statin
64868	Atorvastatin 40mg tablets (Sigma Pharmaceuticals Plc)	Statin
50963	Atorvastatin 40mg tablets (Teva UK Ltd)	Statin
52097	Atorvastatin 40mg tablets (Wockhardt UK Ltd)	Statin
55444	Atorvastatin 40mg tablets (Zentiva)	Statin
72641	Atorvastatin 40mg/5ml oral solution	Statin
55034	Atorvastatin 40mg/5ml oral suspension	Statin
48346	Atorvastatin 60mg tablets	Statin
5775	Atorvastatin 80mg tablets	Statin
58418	Atorvastatin 80mg tablets (A A H Pharmaceuticals Ltd)	Statin
52459	Atorvastatin 80mg tablets (Actavis UK Ltd)	Statin
53772	Atorvastatin 80mg tablets (Alliance Healthcare (Distribution) Ltd)	Statin
62476	Atorvastatin 80mg tablets (Almus Pharmaceuticals Ltd)	Statin
58742	Atorvastatin 80mg tablets (Arrow Generics Ltd)	Statin
59776	Atorvastatin 80mg tablets (Aspire Pharma Ltd)	Statin
63249	Atorvastatin 80mg tablets (Consilient Health Ltd)	Statin
60607	Atorvastatin 80mg tablets (DE Pharmaceuticals)	Statin
52821	Atorvastatin 80mg tablets (Dr Reddy's Laboratories (UK) Ltd)	Statin
53890	Atorvastatin 80mg tablets (Pfizer Ltd)	Statin
60989	Atorvastatin 80mg tablets (Phoenix Healthcare Distribution Ltd)	Statin
67660	Atorvastatin 80mg tablets (Ranbaxy (UK) Ltd)	Statin
66963	Atorvastatin 80mg tablets (Sigma Pharmaceuticals Plc)	Statin
57836	Atorvastatin 80mg tablets (Teva UK Ltd)	Statin
57117	Atorvastatin 80mg tablets (Waymade Healthcare Plc)	Statin
69093	Atorvastatin 80mg tablets (Wockhardt UK Ltd)	Statin
56182	Atorvastatin 80mg tablets (Zentiva)	Statin
420	Cerivastatin 100microgram tablets	Statin
5009	Cerivastatin 200microgram tablets	Statin
5251	Cerivastatin 300microgram tablets	Statin

5278	Cerivastatin 400microgram tablets	Statin
31658	Cerivastatin 800microgram tablets	Statin
7347	Crestor 10mg tablets (AstraZeneca UK Ltd)	Statin
53460	Crestor 10mg tablets (DE Pharmaceuticals)	Statin
15252	Crestor 20mg tablets (AstraZeneca UK Ltd)	Statin
70308	Crestor 20mg tablets (Sigma Pharmaceuticals Plc)	Statin
59447	Crestor 20mg tablets (Waymade Healthcare Plc)	Statin
9930	Crestor 40mg tablets (AstraZeneca UK Ltd)	Statin
57999	Crestor 40mg tablets (Lexon (UK) Ltd)	Statin
17688	Crestor 5mg tablets (AstraZeneca UK Ltd)	Statin
379	Fluvastatin 20mg capsules	Statin
62148	Fluvastatin 20mg capsules (Actavis UK Ltd)	Statin
72308	Fluvastatin 20mg capsules (Alliance Healthcare (Distribution) Ltd)	Statin
59278	Fluvastatin 20mg capsules (Zentiva)	Statin
2137	Fluvastatin 40mg capsules	Statin
53770	Fluvastatin 40mg capsules (A A H Pharmaceuticals Ltd)	Statin
73383	Fluvastatin 40mg capsules (Actavis UK Ltd)	Statin
71029	Fluvastatin 40mg capsules (Sandoz Ltd)	Statin
11627	Fluvastatin 80mg modified-release tablets	Statin
8380	Lescol 20mg capsules (Novartis Pharmaceuticals UK Ltd)	Statin
9153	Lescol 40mg capsules (Novartis Pharmaceuticals UK Ltd)	Statin
74085	Lescol 40mg capsules (Sigma Pharmaceuticals Plc)	Statin
67328	Lescol XL 80mg tablets (Mawdsley-Brooks & Company Ltd)	Statin
5985	Lescol XL 80mg tablets (Novartis Pharmaceuticals UK Ltd)	Statin
47721	Lipitor 10mg chewable tablets (Pfizer Ltd)	Statin
59331	Lipitor 10mg tablets (DE Pharmaceuticals)	Statin
3411	Lipitor 10mg tablets (Pfizer Ltd)	Statin
47630	Lipitor 20mg chewable tablets (Pfizer Ltd)	Statin
56016	Lipitor 20mg chewable tablets (Pfizer Ltd)	Statin
73520	Lipitor 20mg tablets (DE Pharmaceuticals)	Statin
7374	Lipitor 20mg tablets (Pfizer Ltd)	Statin
2955	Lipitor 40mg tablets (Pfizer Ltd)	Statin
53594	Lipitor 80mg tablets (Mawdsley-Brooks & Company Ltd)	Statin
17683	Lipitor 80mg tablets (Pfizer Ltd)	Statin
9315	Lipobay 100microgram Tablet (Bayer Plc)	Statin
53813	Lipobay 100microgram tablets (Bayer Plc)	Statin
9316	Lipobay 200microgram Tablet (Bayer Plc)	Statin
55207	Lipobay 200microgram tablets (Bayer Plc)	Statin
4961	Lipobay 300microgram Tablet (Bayer Plc)	Statin
58480	Lipobay 300microgram tablets (Bayer Plc)	Statin
18442	Lipobay 400microgram Tablet (Bayer Plc)	Statin
62132	Lipobay 400microgram tablets (Bayer Plc)	Statin
1221	Lipostat 10mg tablets (Bristol-Myers Squibb Pharmaceuticals Ltd)	Statin
3690	Lipostat 20mg tablets (Bristol-Myers Squibb Pharmaceuticals Ltd)	Statin
1223	Lipostat 40mg tablets (Bristol-Myers Squibb Pharmaceuticals Ltd)	Statin
32921	Pravastatin 10mg Tablet (Dr Reddy's Laboratories (UK) Ltd)	Statin
490	Pravastatin 10mg tablets	Statin

Carr MJ, *et al. BMJ Qual Saf* 2022; 31:503–514. doi: 10.1136/bmjqs-2021-013613

68156	Pravastatin 10mg tablets (A A H Pharmaceuticals Ltd)	Statin
57397	Pravastatin 10mg tablets (Accord Healthcare Ltd)	Statin
57137	Pravastatin 10mg tablets (Almus Pharmaceuticals Ltd)	Statin
71015	Pravastatin 10mg tablets (Medreich Plc)	Statin
60251	Pravastatin 10mg tablets (Sandoz Ltd)	Statin
50925	Pravastatin 10mg tablets (Sigma Pharmaceuticals Plc)	Statin
43218	Pravastatin 10mg tablets (Teva UK Ltd)	Statin
63787	Pravastatin 10mg tablets (Tillomed Laboratories Ltd)	Statin
56146	Pravastatin 10mg tablets (Waymade Healthcare Plc)	Statin
730	Pravastatin 20mg tablets	Statin
40382	Pravastatin 20mg tablets (A A H Pharmaceuticals Ltd)	Statin
59508	Pravastatin 20mg tablets (Accord Healthcare Ltd)	Statin
52755	Pravastatin 20mg tablets (Alliance Healthcare (Distribution) Ltd)	Statin
54607	Pravastatin 20mg tablets (Almus Pharmaceuticals Ltd)	Statin
51890	Pravastatin 20mg tablets (Medreich Plc)	Statin
56735	Pravastatin 20mg tablets (Mylan)	Statin
63074	Pravastatin 20mg tablets (PLIVA Pharma Ltd)	Statin
57296	Pravastatin 20mg tablets (Phoenix Healthcare Distribution Ltd)	Statin
67829	Pravastatin 20mg tablets (Sandoz Ltd)	Statin
61134	Pravastatin 20mg tablets (Sigma Pharmaceuticals Plc)	Statin
36377	Pravastatin 20mg tablets (Teva UK Ltd)	Statin
56607	Pravastatin 20mg tablets (Waymade Healthcare Plc)	Statin
1219	Pravastatin 40mg tablets	Statin
34820	Pravastatin 40mg tablets (A A H Pharmaceuticals Ltd)	Statin
56893	Pravastatin 40mg tablets (Accord Healthcare Ltd)	Statin
72048	Pravastatin 40mg tablets (Actavis UK Ltd)	Statin
55912	Pravastatin 40mg tablets (Alliance Healthcare (Distribution) Ltd)	Statin
54435	Pravastatin 40mg tablets (Almus Pharmaceuticals Ltd)	Statin
62979	Pravastatin 40mg tablets (Kent Pharmaceuticals Ltd)	Statin
51676	Pravastatin 40mg tablets (Medreich Plc)	Statin
47988	Pravastatin 40mg tablets (Mylan)	Statin
56916	Pravastatin 40mg tablets (PLIVA Pharma Ltd)	Statin
48097	Pravastatin 40mg tablets (Teva UK Ltd)	Statin
57108	Pravastatin 40mg tablets (Waymade Healthcare Plc)	Statin
72149	Pravastatin 5mg/5ml oral suspension	Statin
44878	Ranzolont 10mg tablets (Ranbaxy (UK) Ltd)	Statin
713	Rosuvastatin 10mg tablets	Statin
74552	Rosuvastatin 10mg tablets (Milpharm Ltd)	Statin
57763	Rosuvastatin 10mg tablets (Waymade Healthcare Plc)	Statin
6213	Rosuvastatin 20mg tablets	Statin
73025	Rosuvastatin 20mg tablets (Mylan)	Statin
71014	Rosuvastatin 20mg tablets (Waymade Healthcare Plc)	Statin
58617	Rosuvastatin 20mg/5ml oral suspension	Statin
9897	Rosuvastatin 40mg tablets	Statin
7554	Rosuvastatin 5mg tablets	Statin
60160	Rosuvastatin 5mg tablets (Mawdsley-Brooks & Company Ltd)	Statin
59452	Rosuvastatin 5mg tablets (Waymade Healthcare Plc)	Statin

Carr MJ, et al. BMJ Qual Saf 2022; 31:503–514. doi: 10.1136/bmjqs-2021-013613

24509	SIMVASTATIN	Statin
29438	SIMVASTATIN	Statin
13041	Simvador 10mg tablets (Discovery Pharmaceuticals)	Statin
9920	Simvador 20mg tablets (Discovery Pharmaceuticals)	Statin
802	Simvador 40mg tablets (Discovery Pharmaceuticals)	Statin
39870	Simvador 80mg tablets (Discovery Pharmaceuticals)	Statin
34560	Simvastatin 10mg Tablet (Ratiopharm UK Ltd)	Statin
42	Simvastatin 10mg tablets	Statin
34955	Simvastatin 10mg tablets (A A H Pharmaceuticals Ltd)	Statin
54655	Simvastatin 10mg tablets (Accord Healthcare Ltd)	Statin
48078	Simvastatin 10mg tablets (Actavis UK Ltd)	Statin
51233	Simvastatin 10mg tablets (Alliance Healthcare (Distribution) Ltd)	Statin
61360	Simvastatin 10mg tablets (Almus Pharmaceuticals Ltd)	Statin
47774	Simvastatin 10mg tablets (Arrow Generics Ltd)	Statin
53822	Simvastatin 10mg tablets (Bristol Laboratories Ltd)	Statin
67098	Simvastatin 10mg tablets (Brown & Burk UK Ltd)	Statin
64180	Simvastatin 10mg tablets (Crescent Pharma Ltd)	Statin
64968	Simvastatin 10mg tablets (DE Pharmaceuticals)	Statin
53908	Simvastatin 10mg tablets (Dexcel-Pharma Ltd)	Statin
72050	Simvastatin 10mg tablets (Genesis Pharmaceuticals Ltd)	Statin
34481	Simvastatin 10mg tablets (IVAX Pharmaceuticals UK Ltd)	Statin
48051	Simvastatin 10mg tablets (Kent Pharmaceuticals Ltd)	Statin
51085	Simvastatin 10mg tablets (Medreich Plc)	Statin
53415	Simvastatin 10mg tablets (Milpharm Ltd)	Statin
34535	Simvastatin 10mg tablets (Mylan)	Statin
58755	Simvastatin 10mg tablets (Phoenix Healthcare Distribution Ltd)	Statin
48058	Simvastatin 10mg tablets (Ranbaxy (UK) Ltd)	Statin
54493	Simvastatin 10mg tablets (Relonchem Ltd)	Statin
61321	Simvastatin 10mg tablets (Sandoz Ltd)	Statin
51715	Simvastatin 10mg tablets (Sigma Pharmaceuticals Plc)	Statin
54976	Simvastatin 10mg tablets (Somex Pharma)	Statin
40340	Simvastatin 10mg tablets (Teva UK Ltd)	Statin
47948	Simvastatin 10mg tablets (Tillomed Laboratories Ltd)	Statin
61665	Simvastatin 10mg tablets (Waymade Healthcare Plc)	Statin
52625	Simvastatin 10mg tablets (Wockhardt UK Ltd)	Statin
67745	Simvastatin 10mg tablets (Zentiva)	Statin
52676	Simvastatin 10mg/5ml oral suspension	Statin
34476	Simvastatin 20mg Tablet (Ratiopharm UK Ltd)	Statin
25	Simvastatin 20mg tablets	Statin
33082	Simvastatin 20mg tablets (A A H Pharmaceuticals Ltd)	Statin
52257	Simvastatin 20mg tablets (Accord Healthcare Ltd)	Statin
45245	Simvastatin 20mg tablets (Actavis UK Ltd)	Statin
49062	Simvastatin 20mg tablets (Alliance Healthcare (Distribution) Ltd)	Statin
54947	Simvastatin 20mg tablets (Almus Pharmaceuticals Ltd)	Statin
48018	Simvastatin 20mg tablets (Arrow Generics Ltd)	Statin
52953	Simvastatin 20mg tablets (Bristol Laboratories Ltd)	Statin
69413	Simvastatin 20mg tablets (Brown & Burk UK Ltd)	Statin

64104	Simvastatin 20mg tablets (Crescent Pharma Ltd)	Statin
65679	Simvastatin 20mg tablets (DE Pharmaceuticals)	Statin
39060	Simvastatin 20mg tablets (Dexcel-Pharma Ltd)	Statin
68686	Simvastatin 20mg tablets (Genesis Pharmaceuticals Ltd)	Statin
34366	Simvastatin 20mg tablets (IVAX Pharmaceuticals UK Ltd)	Statin
34891	Simvastatin 20mg tablets (Kent Pharmaceuticals Ltd)	Statin
50754	Simvastatin 20mg tablets (Medreich Plc)	Statin
51483	Simvastatin 20mg tablets (Milpharm Ltd)	Statin
34312	Simvastatin 20mg tablets (Mylan)	Statin
55452	Simvastatin 20mg tablets (Phoenix Healthcare Distribution Ltd)	Statin
40601	Simvastatin 20mg tablets (Ranbaxy (UK) Ltd)	Statin
50564	Simvastatin 20mg tablets (Relonchem Ltd)	Statin
45235	Simvastatin 20mg tablets (Sandoz Ltd)	Statin
52812	Simvastatin 20mg tablets (Sigma Pharmaceuticals Plc)	Statin
53087	Simvastatin 20mg tablets (Somex Pharma)	Statin
34316	Simvastatin 20mg tablets (Teva UK Ltd)	Statin
53676	Simvastatin 20mg tablets (Tillomed Laboratories Ltd)	Statin
58315	Simvastatin 20mg tablets (Waymade Healthcare Plc)	Statin
34814	Simvastatin 20mg tablets (Wockhardt UK Ltd)	Statin
67773	Simvastatin 20mg tablets (Zentiva)	Statin
39675	Simvastatin 20mg/5ml Oral suspension (Martindale Pharmaceuticals Ltd)	Statin
818	Simvastatin 20mg/5ml oral solution sugar free	Statin
54266	Simvastatin 20mg/5ml oral suspension	Statin
48221	Simvastatin 20mg/5ml oral suspension sugar free	Statin
54606	Simvastatin 20mg/5ml oral suspension sugar free (A A H Pharmaceuticals Ltd)	Statin
65925	Simvastatin 20mg/5ml oral suspension sugar free (Alliance Healthcare (Distribution) Ltd)	Statin
44528	Simvastatin 20mg/5ml oral suspension sugar free (Rosemont Pharmaceuticals Ltd)	Statin
56065	Simvastatin 20mg/5ml oral suspension sugar free (Waymade Healthcare Plc)	Statin
57329	Simvastatin 25mg/5ml oral suspension	Statin
34879	Simvastatin 40mg Tablet (Niche Generics Ltd)	Statin
34545	Simvastatin 40mg Tablet (Ratiopharm UK Ltd)	Statin
51	Simvastatin 40mg tablets	Statin
34502	Simvastatin 40mg tablets (A A H Pharmaceuticals Ltd)	Statin
50703	Simvastatin 40mg tablets (Accord Healthcare Ltd)	Statin
34969	Simvastatin 40mg tablets (Actavis UK Ltd)	Statin
48867	Simvastatin 40mg tablets (Alliance Healthcare (Distribution) Ltd)	Statin
46878	Simvastatin 40mg tablets (Almus Pharmaceuticals Ltd)	Statin
45346	Simvastatin 40mg tablets (Arrow Generics Ltd)	Statin
49061	Simvastatin 40mg tablets (Bristol Laboratories Ltd)	Statin
68563	Simvastatin 40mg tablets (Brown & Burk UK Ltd)	Statin
64307	Simvastatin 40mg tablets (Crescent Pharma Ltd)	Statin
65181	Simvastatin 40mg tablets (DE Pharmaceuticals)	Statin
44650	Simvastatin 40mg tablets (Dexcel-Pharma Ltd)	Statin
34381	Simvastatin 40mg tablets (IVAX Pharmaceuticals UK Ltd)	Statin
45219	Simvastatin 40mg tablets (Kent Pharmaceuticals Ltd)	Statin

51166	Simvastatin 40mg tablets (Medreich Plc)	Statin
50670	Simvastatin 40mg tablets (Milpharm Ltd)	Statin
34353	Simvastatin 40mg tablets (Mylan)	Statin
53966	Simvastatin 40mg tablets (Phoenix Healthcare Distribution Ltd)	Statin
52098	Simvastatin 40mg tablets (Ranbaxy (UK) Ltd)	Statin
50483	Simvastatin 40mg tablets (Relonchem Ltd)	Statin
37434	Simvastatin 40mg tablets (Sandoz Ltd)	Statin
54240	Simvastatin 40mg tablets (Sigma Pharmaceuticals Plc)	Statin
50882	Simvastatin 40mg tablets (Somex Pharma)	Statin
34376	Simvastatin 40mg tablets (Teva UK Ltd)	Statin
62137	Simvastatin 40mg tablets (Waymade Healthcare Plc)	Statin
34907	Simvastatin 40mg tablets (Wockhardt UK Ltd)	Statin
65901	Simvastatin 40mg tablets (Zentiva)	Statin
39652	Simvastatin 40mg/5ml oral solution sugar free	Statin
54985	Simvastatin 40mg/5ml oral suspension	Statin
48431	Simvastatin 40mg/5ml oral suspension sugar free	Statin
61155	Simvastatin 40mg/5ml oral suspension sugar free (A A H Pharmaceuticals Ltd)	Statin
54819	Simvastatin 40mg/5ml oral suspension sugar free (Rosemont Pharmaceuticals Ltd)	Statin
5148	Simvastatin 80mg tablets	Statin
32909	Simvastatin 80mg tablets (A A H Pharmaceuticals Ltd)	Statin
49587	Simvastatin 80mg tablets (Almus Pharmaceuticals Ltd)	Statin
46956	Simvastatin 80mg tablets (Arrow Generics Ltd)	Statin
71773	Simvastatin 80mg tablets (Brown & Burk UK Ltd)	Statin
52962	Simvastatin 80mg tablets (Medreich Plc)	Statin
41657	Simvastatin 80mg tablets (Teva UK Ltd)	Statin
57568	Zocor 10mg tablets (Lexon (UK) Ltd)	Statin
2718	Zocor 10mg tablets (Merck Sharp & Dohme Ltd)	Statin
56481	Zocor 10mg tablets (Sigma Pharmaceuticals Plc)	Statin
7196	Zocor 20mg tablets (Merck Sharp & Dohme Ltd)	Statin
56494	Zocor 20mg tablets (Sigma Pharmaceuticals Plc)	Statin
53340	Zocor 40mg tablets (Lexon (UK) Ltd)	Statin
6168	Zocor 40mg tablets (Merck Sharp & Dohme Ltd)	Statin
22579	Zocor 80mg tablets (Merck Sharp & Dohme Ltd)	Statin
31930	Zocor heart-pro 10mg Tablet (McNeil Products Ltd)	Statin
21020	Inegy 10mg/20mg tablets (Merck Sharp & Dohme Ltd)	Statin/Ezetimibe
17059	Inegy 10mg/40mg tablets (Merck Sharp & Dohme Ltd)	Statin/Ezetimibe
16186	Inegy 10mg/80mg tablets (Merck Sharp & Dohme Ltd)	Statin/Ezetimibe
7552	Simvastatin 20mg / Ezetimibe 10mg tablets	Statin/Ezetimibe
11815	Simvastatin 20mg with ezetimibe 10mg tablet	Statin/Ezetimibe
10172	Simvastatin 40mg / Ezetimibe 10mg tablets	Statin/Ezetimibe
10183	Simvastatin 40mg with ezetimibe 10mg tablet	Statin/Ezetimibe
14219	Simvastatin 80mg / Ezetimibe 10mg tablets	Statin/Ezetimibe
10206	Simvastatin 80mg with ezetimibe 10mg tablet	Statin/Ezetimibe
69528	Cholib 145mg/20mg tablets (Mylan)	Statin/Fibrate
70486	Cholib 145mg/40mg tablets (Mylan)	Statin/Fibrate
66780	Fenofibrate 145mg / Simvastatin 20mg tablets	Statin/Fibrate

66505	Fenofibrate 145mg / Simvastatin 40mg tablets	Statin/Fibrate

Appendix 22. Product codes for prescriptions of antiplatelet medications

Product code	Term	Drug class
23495	ASPIRIN	Aspirin
15517	ASPIRIN 100 MG SUP	Aspirin
30695	ASPIRIN 120 MG SUP	Aspirin
26792	ASPIRIN 125 MG SUP	Aspirin
2924	ASPIRIN 150 MG TAB	Aspirin
26099	ASPIRIN 175 MG SUP	Aspirin
26424	ASPIRIN 200 MG SUP	Aspirin
24857	ASPIRIN 250 MG SUP	Aspirin
28238	ASPIRIN 300MG/LYSINE 245MG 300 MG TAB	Aspirin
7462	ASPIRIN 325 MG CAP	Aspirin
8843	ASPIRIN 325 MG TAB	Aspirin
7486	ASPIRIN 37.5 MG TAB	Aspirin
111	ASPIRIN 40 MG CAP	Aspirin
7417	ASPIRIN 40 MG TAB	Aspirin
4523	ASPIRIN 50 MG CAP	Aspirin
20206	ASPIRIN 50 MG SUP	Aspirin
23491	ASPIRIN 500 MG SUP	Aspirin
383	ASPIRIN 60 MG TAB	Aspirin
33075	ASPIRIN 600MG/GLYCINE 300MG 600 MG TAB	Aspirin
42061	ASPIRIN 65 MG SUP	Aspirin
216	ASPIRIN 70 MG TAB	Aspirin
1486	ASPIRIN 75 MG SUP	Aspirin
19674	ASPIRIN DISPERSIBLE	Aspirin
28707	ASPIRIN M/F 324 MG TAB	Aspirin
22863	ASPIRIN S/R 500 MG TAB	Aspirin
11941	ASPIRIN SACHETS 30 MG	Aspirin
19813	ASPIRIN SOLUBLE	Aspirin
12102	ASPIRIN SOLUBLE 100 MG TAB	Aspirin
2754	ASPIRIN SOLUBLE 150 MG TAB	Aspirin
4271	ASPIRIN SOLUBLE 200 MG TAB	Aspirin
7944	ASPIRIN SOLUBLE 40 MG CAP	Aspirin
27467	ASPIRIN SOLUBLE 400 MG TAB	Aspirin
15397	ASPIRIN SOLUBLE 50 MG TAB	Aspirin
8920	ASPIRIN SOLUBLE 500 MG TAB	Aspirin
15447	ASPIRIN SOLUBLE 600 MG TAB	Aspirin
7915	ASPIRIN SR 100 MG TAB	Aspirin
7665	ASPIRIN SR 300 MG TAB	Aspirin
9027	ASPIRIN disp 150 MG TAB	Aspirin
22107	ASPIRIN disp 200 MG TAB	Aspirin
8734	ASPIRIN disp 37.5 MG TAB	Aspirin
15044	ASPIRIN disp 500 MG TAB	Aspirin
22824	ASPIRIN disp 600 MG TAB	Aspirin
25718	Angettes 75 tablets (Bristol-Myers Squibb Pharmaceuticals Ltd)	Aspirin
59244	Aspirin 100mg capsules	Aspirin

36543	Aspirin 100mg effervescent tablets	Aspirin
9301	Aspirin 100mg modified-release tablets	Aspirin
21382	Aspirin 150mg / Isosorbide mononitrate 60mg modified-release tablets	Aspirin
66171	Aspirin 150mg Suppository (Distriphar (UK))	Aspirin
15364	Aspirin 150mg suppositories	Aspirin
49799	Aspirin 150mg suppositories (A A H Pharmaceuticals Ltd)	Aspirin
53791	Aspirin 150mg suppositories (Alliance Healthcare (Distribution) Ltd)	Aspirin
68051	Aspirin 150mg suppositories (Colorama Pharmaceuticals Ltd)	Aspirin
51474	Aspirin 150mg suppositories (Martindale Pharmaceuticals Ltd)	Aspirin
60693	Aspirin 15mg/5ml oral solution	Aspirin
67521	Aspirin 15mg/5ml oral suspension	Aspirin
10305	Aspirin 162.5mg capsules	Aspirin
39738	Aspirin 162.5mg modified-release capsules	Aspirin
67858	Aspirin 25mg capsules	Aspirin
60694	Aspirin 25mg/5ml oral solution	Aspirin
33662	Aspirin 300mg Dispersible tablet (A A H Pharmaceuticals Ltd)	Aspirin
70841	Aspirin 300mg Dispersible tablet (Family Health)	Aspirin
30920	Aspirin 300mg Dispersible tablet (M & A Pharmachem Ltd)	Aspirin
44639	Aspirin 300mg Dispersible tablet (Nucare Plc)	Aspirin
45840	Aspirin 300mg Dispersible tablet (Numark Management Ltd)	Aspirin
33668	Aspirin 300mg Dispersible tablet (Rusco Ltd)	Aspirin
41594	Aspirin 300mg Dispersible tablet (Teva UK Ltd)	Aspirin
40144	Aspirin 300mg Dispersible tablet (Thornton & Ross Ltd)	Aspirin
34762	Aspirin 300mg Gastro-resistant tablet (Galen Ltd)	Aspirin
43060	Aspirin 300mg Soluble tablet (Celltech Pharma Europe Ltd)	Aspirin
45851	Aspirin 300mg Soluble tablet (Ranbaxy (UK) Ltd)	Aspirin
31210	Aspirin 300mg Tablet (Co-operative)	Aspirin
53622	Aspirin 300mg Tablet (M & A Pharmachem Ltd)	Aspirin
53711	Aspirin 300mg Tablet (Nucare Plc)	Aspirin
71821	Aspirin 300mg Tablet (Numark Management Ltd)	Aspirin
52280	Aspirin 300mg Tablet (Wockhardt UK Ltd)	Aspirin
52044	Aspirin 300mg caplets (The Boots Company Plc)	Aspirin
62334	Aspirin 300mg caplets (Wockhardt UK Ltd)	Aspirin
377	Aspirin 300mg dispersible tablets	Aspirin
34309	Aspirin 300mg dispersible tablets (A A H Pharmaceuticals Ltd)	Aspirin
32210	Aspirin 300mg dispersible tablets (Actavis UK Ltd)	Aspirin
53816	Aspirin 300mg dispersible tablets (Alliance Healthcare (Distribution) Ltd)	Aspirin
67754	Aspirin 300mg dispersible tablets (Almus Pharmaceuticals Ltd)	Aspirin
50555	Aspirin 300mg dispersible tablets (DE Pharmaceuticals)	Aspirin
55230	Aspirin 300mg dispersible tablets (Kent Pharmaceuticals Ltd)	Aspirin
67160	Aspirin 300mg dispersible tablets (Lloyds Pharmacy Ltd)	Aspirin
71078	Aspirin 300mg dispersible tablets (Mawdsley-Brooks & Company Ltd)	Aspirin
56007	Aspirin 300mg dispersible tablets (Sigma Pharmaceuticals Plc)	Aspirin
8645	Aspirin 300mg effervescent tablets	Aspirin
7516	Aspirin 300mg effervescent tablets sugar free	Aspirin
434	Aspirin 300mg gastro-resistant tablets	Aspirin
43434	Aspirin 300mg gastro-resistant tablets (A A H Pharmaceuticals Ltd)	Aspirin

53804	Aspirin 300mg gastro-resistant tablets (Alliance Healthcare (Distribution) Ltd)	Aspirin
58331	Aspirin 300mg gastro-resistant tablets (Mylan)	Aspirin
43806	Aspirin 300mg gastro-resistant tablets (Sandoz Ltd)	Aspirin
8186	Aspirin 300mg modified-release tablets	Aspirin
18217	Aspirin 300mg orodispersible tablets sugar free	Aspirin
645	Aspirin 300mg suppositories	Aspirin
62430	Aspirin 300mg suppositories (A A H Pharmaceuticals Ltd)	Aspirin
67362	Aspirin 300mg suppositories (Alliance Healthcare (Distribution) Ltd)	Aspirin
254	Aspirin 300mg tablets	Aspirin
41569	Aspirin 300mg tablets (A A H Pharmaceuticals Ltd)	Aspirin
34386	Aspirin 300mg tablets (Actavis UK Ltd)	Aspirin
54526	Aspirin 300mg tablets (Alliance Healthcare (Distribution) Ltd)	Aspirin
55579	Aspirin 300mg tablets (Almus Pharmaceuticals Ltd)	Aspirin
48165	Aspirin 300mg tablets (Aspar Pharmaceuticals Ltd)	Aspirin
60278	Aspirin 300mg tablets (DE Pharmaceuticals)	Aspirin
49220	Aspirin 300mg tablets (Kent Pharmaceuticals Ltd)	Aspirin
52905	Aspirin 300mg tablets (Lloyds Pharmacy Ltd)	Aspirin
48000	Aspirin 300mg tablets (Sigma Pharmaceuticals Plc)	Aspirin
24960	Aspirin 300mg tablets (Vantage)	Aspirin
56736	Aspirin 300mg tablets (Waymade Healthcare Plc)	Aspirin
54734	Aspirin 300mg tablets (Wockhardt UK Ltd)	Aspirin
28810	Aspirin 300mg with Glycine 133mg soluble tablets	Aspirin
29848	Aspirin 300mg with Glycine 150mg chewable tablets	Aspirin
31870	Aspirin 320mg tablets	Aspirin
22138	Aspirin 324mg modified-release tablets	Aspirin
9939	Aspirin 500mg effervescent tablets sugar free	Aspirin
657	Aspirin 500mg granules sachets sugar free	Aspirin
36521	Aspirin 500mg modified-release tablets	Aspirin
74096	Aspirin 50mg/5ml oral suspension	Aspirin
21380	Aspirin 75mg / Isosorbide mononitrate 60mg modified-release tablets	Aspirin
31211	Aspirin 75mg Dispersible tablet (A A H Pharmaceuticals Ltd)	Aspirin
34942	Aspirin 75mg Dispersible tablet (Nucare Plc)	Aspirin
33320	Aspirin 75mg Dispersible tablet (Sovereign Medical Ltd)	Aspirin
34796	Aspirin 75mg Gastro-resistant tablet (Galen Ltd)	Aspirin
40381	Aspirin 75mg Soluble tablet (C P Pharmaceuticals Ltd)	Aspirin
45643	Aspirin 75mg Soluble tablet (Celltech Pharma Europe Ltd)	Aspirin
34385	Aspirin 75mg Soluble tablet (Co-operative)	Aspirin
48021	Aspirin 75mg Tablet (Hillcross Pharmaceuticals Ltd)	Aspirin
3	Aspirin 75mg dispersible tablets	Aspirin
33656	Aspirin 75mg dispersible tablets (A A H Pharmaceuticals Ltd)	Aspirin
32036	Aspirin 75mg dispersible tablets (Actavis UK Ltd)	Aspirin
49060	Aspirin 75mg dispersible tablets (Alliance Healthcare (Distribution) Ltd)	Aspirin
54284	Aspirin 75mg dispersible tablets (Almus Pharmaceuticals Ltd)	Aspirin
59791	Aspirin 75mg dispersible tablets (Aspar Pharmaceuticals Ltd)	Aspirin
52618	Aspirin 75mg dispersible tablets (Bristol Laboratories Ltd)	Aspirin
66345	Aspirin 75mg dispersible tablets (DE Pharmaceuticals)	Aspirin

54997	Aspirin 75mg dispersible tablets (Dowelhurst Ltd)	Aspirin
31953	Aspirin 75mg dispersible tablets (IVAX Pharmaceuticals UK Ltd)	Aspirin
33676	Aspirin 75mg dispersible tablets (Kent Pharmaceuticals Ltd)	Aspirin
54565	Aspirin 75mg dispersible tablets (Lloyds Pharmacy Ltd)	Aspirin
71676	Aspirin 75mg dispersible tablets (Mawdsley-Brooks & Company Ltd)	Aspirin
66546	Aspirin 75mg dispersible tablets (Numark Ltd)	Aspirin
56995	Aspirin 75mg dispersible tablets (Phoenix Healthcare Distribution Ltd)	Aspirin
49685	Aspirin 75mg dispersible tablets (Sigma Pharmaceuticals Plc)	Aspirin
31954	Aspirin 75mg dispersible tablets (Teva UK Ltd)	Aspirin
50926	Aspirin 75mg dispersible tablets (The Boots Company Plc)	Aspirin
34434	Aspirin 75mg dispersible tablets (Thornton & Ross Ltd)	Aspirin
56996	Aspirin 75mg dispersible tablets (Waymade Healthcare Plc)	Aspirin
47937	Aspirin 75mg dispersible tablets (Wockhardt UK Ltd)	Aspirin
57057	Aspirin 75mg dispersible tablets (Wockhardt UK Ltd)	Aspirin
66861	Aspirin 75mg effervescent tablets	Aspirin
34	Aspirin 75mg gastro-resistant tablets	Aspirin
47992	Aspirin 75mg gastro-resistant tablets (A A H Pharmaceuticals Ltd)	Aspirin
34797	Aspirin 75mg gastro-resistant tablets (Actavis UK Ltd)	Aspirin
74786	Aspirin 75mg gastro-resistant tablets (Alliance Healthcare (Distribution) Ltd)	Aspirin
43709	Aspirin 75mg gastro-resistant tablets (Almus Pharmaceuticals Ltd)	Aspirin
59021	Aspirin 75mg gastro-resistant tablets (Bristol Laboratories Ltd)	Aspirin
34611	Aspirin 75mg gastro-resistant tablets (C P Pharmaceuticals Ltd)	Aspirin
60777	Aspirin 75mg gastro-resistant tablets (DE Pharmaceuticals)	Aspirin
73994	Aspirin 75mg gastro-resistant tablets (Greenfield Pharmaceuticals Ltd)	Aspirin
34485	Aspirin 75mg gastro-resistant tablets (IVAX Pharmaceuticals UK Ltd)	Aspirin
31956	Aspirin 75mg gastro-resistant tablets (Kent Pharmaceuticals Ltd)	Aspirin
32992	Aspirin 75mg gastro-resistant tablets (Mylan)	Aspirin
66563	Aspirin 75mg gastro-resistant tablets (Phoenix Healthcare Distribution Ltd)	Aspirin
31938	Aspirin 75mg gastro-resistant tablets (Sandoz Ltd)	Aspirin
33293	Aspirin 75mg gastro-resistant tablets (Sterwin Medicines)	Aspirin
41512	Aspirin 75mg gastro-resistant tablets (Teva UK Ltd)	Aspirin
59253	Aspirin 75mg gastro-resistant tablets (Waymade Healthcare Plc)	Aspirin
53178	Aspirin 75mg gastro-resistant tablets (Wockhardt UK Ltd)	Aspirin
51561	Aspirin 75mg gastro-resistant tablets (Zanza Laboratories Ltd)	Aspirin
16	Aspirin 75mg tablets	Aspirin
50949	Aspirin 75mg tablets (A A H Pharmaceuticals Ltd)	Aspirin
71663	Aspirin 75mg tablets (Actavis UK Ltd)	Aspirin
59728	Aspirin 75mg tablets (Alissa Healthcare Research Ltd)	Aspirin
54430	Aspirin 75mg tablets (Alliance Healthcare (Distribution) Ltd)	Aspirin
60127	Aspirin 75mg tablets (DE Pharmaceuticals)	Aspirin
71192	Aspirin 75mg tablets (Kent Pharmaceuticals Ltd)	Aspirin
48974	Aspirin 75mg tablets (Phoenix Healthcare Distribution Ltd)	Aspirin
68752	Aspirin 75mg tablets (Sigma Pharmaceuticals Plc)	Aspirin
56883	Aspirin 75mg tablets (Waymade Healthcare Plc)	Aspirin
74302	Aspirin 75mg/5ml oral suspension	Aspirin

34666	Aspirin ec 300mg Gastro-resistant tablet (A A H Pharmaceuticals Ltd)	Aspirin
395	Aspirin mixture	Aspirin
10310	Aspirin powder	Aspirin
64071	Aspirin powder (J M Loveridge Ltd)	Aspirin
1902	Aspirn 600mg gastro-resistant tablets	Aspirin
23932	Aspro Clear 300mg effervescent tablets (Bayer Plc)	Aspirin
29759	Aspro Tablet (Roche Consumer Health)	Aspirin
11977	Aspro clear maximum strength tablets	Aspirin
67124	Bisoprolol 10mg / Aspirin 75mg capsules	Aspirin
65027	Bisoprolol 5mg / Aspirin 100mg capsules	Aspirin
27150	CAPRIN E/C 324 MG TAB	Aspirin
24025	Caprin 300mg gastro-resistant tablets (Pinewood Healthcare)	Aspirin
9144	Caprin 75mg gastro-resistant tablets (Wockhardt UK Ltd)	Aspirin
31858	Caspac xl 162.5mg Capsule (Pharmacia Ltd)	Aspirin
23488	Claradin 300mg Tablet (Nicholas Laboratories Ltd)	Aspirin
17828	DISPRIN CV 100 MG TAB	Aspirin
361	DISPRIN TAB	Aspirin
70549	Danamep 75mg dispersible tablets (Ecogen Europe Ltd)	Aspirin
393	Disprin 300mg dispersible tablets (Reckitt Benckiser Healthcare (UK) Ltd)	Aspirin
8185	Disprin CV 300mg modified-release tablets (Reckitt Benckiser Healthcare (UK) Ltd)	Aspirin
22232	Disprin Direct 300mg orodispersible tablets (Reckitt Benckiser Healthcare (UK) Ltd)	Aspirin
17920	Disprin cv 100mg Modified-release tablet (Reckitt Benckiser Healthcare (UK) Ltd)	Aspirin
18329	Enprin 75mg gastro-resistant tablets (Galpharm International Ltd)	Aspirin
43679	Flamasacard 162.5mg Modified-release capsule (Abbey Pharmaceuticals Ltd)	Aspirin
25212	IMAZIN XL	Aspirin
25217	IMAZIN XL FORTE	Aspirin
18030	Imazin XL forte tablets (Napp Pharmaceuticals Ltd)	Aspirin
13882	Imazin XL tablets (Napp Pharmaceuticals Ltd)	Aspirin
63603	Laboprin Tablet (Laboratories For Applied Biology Ltd)	Aspirin
41766	Maximum Strength Aspro Clear 500mg effervescent tablets (Bayer Plc)	Aspirin
19189	Micropirin 75mg Gastro-resistant tablet (Ratiopharm UK Ltd)	Aspirin
6696	Micropirin 75mg gastro-resistant tablets (Dexcel-Pharma Ltd)	Aspirin
19577	NU-SEALS ASPIRIN	Aspirin
19797	NU-SEALS ASPIRIN	Aspirin
6007	Nu-Seals 300 gastro-resistant tablets (Alliance Pharmaceuticals Ltd)	Aspirin
6006	Nu-Seals 75 gastro-resistant tablets (Alliance Pharmaceuticals Ltd)	Aspirin
1049	Nu-seals aspirin 600mg Tablet (Eli Lilly and Company Ltd)	Aspirin
1137	Nu-seals aspirin ec 300mg Gastro-resistant tablet (Eli Lilly and Company Ltd)	Aspirin
2628	Nu-seals aspirin ec 75mg Gastro-resistant tablet (Eli Lilly and Company Ltd)	Aspirin
23878	Nu-seals cardio ec 75mg Gastro-resistant tablet (Genus Pharmaceuticals Ltd)	Aspirin
2607	Paynocil Tablet (Beecham Research Laboratories)	Aspirin

17704	Platet 100mg Effervescent tablet (Roche Products Ltd)	Aspirin
25335	PostMI 75 EC tablets (Ashbourne Pharmaceuticals Ltd)	Aspirin
23593	PostMI 75 dispersible tablets (Ashbourne Pharmaceuticals Ltd)	Aspirin
21921	Postmi ec 300mg Gastro-resistant tablet (Ashbourne Pharmaceuticals Ltd)	Aspirin
2105	Solprin 300mg Tablet (Reckitt Benckiser Healthcare (UK) Ltd)	Aspirin
22618	Solprin 75mg Tablet (Reckitt Benckiser Healthcare (UK) Ltd)	Aspirin
4679	Asasantin Retard capsules (Boehringer Ingelheim Ltd)	Aspirin/Other
57585	Asasantin Retard capsules (Dowelhurst Ltd)	Aspirin/Other
56503	Asasantin Retard capsules (Mawdsley-Brooks & Company Ltd)	Aspirin/Other
10031	Aspirin 25mg with Dipyridamole 200mg modified-release capsules	Aspirin/Other
6666	Dipyridamole 200mg modified-release / Aspirin 25mg capsules	Aspirin/Other
69216	Dipyridamole 200mg modified-release / Aspirin 25mg capsules (A A H Pharmaceuticals Ltd)	Aspirin/Other
68764	Molita 200mg/25mg modified-release capsules (Dr Reddy's Laboratories (UK) Ltd)	Aspirin/Other
17817	CLOPIDOGREL FC	Clopidogrel
45905	Clopidogrel 1mg/ml oral suspension	Clopidogrel
38349	Clopidogrel 300mg tablets	Clopidogrel
489	Clopidogrel 75mg tablets	Clopidogrel
54700	Clopidogrel 75mg tablets (A A H Pharmaceuticals Ltd)	Clopidogrel
42750	Clopidogrel 75mg tablets (Actavis UK Ltd)	Clopidogrel
62855	Clopidogrel 75mg tablets (Alliance Healthcare (Distribution) Ltd)	Clopidogrel
63450	Clopidogrel 75mg tablets (Almus Pharmaceuticals Ltd)	Clopidogrel
58448	Clopidogrel 75mg tablets (Aspire Pharma Ltd)	Clopidogrel
58347	Clopidogrel 75mg tablets (DE Pharmaceuticals)	Clopidogrel
52761	Clopidogrel 75mg tablets (Dr Reddy's Laboratories (UK) Ltd)	Clopidogrel
65909	Clopidogrel 75mg tablets (Milpharm Ltd)	Clopidogrel
57036	Clopidogrel 75mg tablets (Mylan)	Clopidogrel
53751	Clopidogrel 75mg tablets (Phoenix Healthcare Distribution Ltd)	Clopidogrel
62978	Clopidogrel 75mg tablets (Sandoz Ltd)	Clopidogrel
56807	Clopidogrel 75mg tablets (Teva UK Ltd)	Clopidogrel
55161	Clopidogrel 75mg tablets (Wockhardt UK Ltd)	Clopidogrel
67037	Clopidogrel 75mg tablets (Zentiva)	Clopidogrel
59904	Clopidogrel 75mg/5ml oral solution	Clopidogrel
46891	Clopidogrel 75mg/5ml oral suspension	Clopidogrel
40913	Grepid 75mg tablets (Kent Pharmaceuticals Ltd)	Clopidogrel
17816	PLAVIX FC	Clopidogrel
38998	Plavix 300mg tablets (Sanofi)	Clopidogrel
836	Plavix 75mg tablets (Sanofi)	Clopidogrel
30554	Abciximab 10mg/5ml solution for injection vials	Other
33877	Aggrastat 12.5mg/250ml infusion bags (Correvio GmbH)	Other
66784	Attia 200mg modified-release capsules (Dr Reddy's Laboratories (UK) Ltd)	Other
68710	Brilique 60mg tablets (AstraZeneca UK Ltd)	Other
47895	Brilique 90mg tablets (AstraZeneca UK Ltd)	Other
21989	Cerebrovase 100mg Tablet (Ashbourne Pharmaceuticals Ltd)	Other
25284	Cerebrovase 25mg Tablet (Ashbourne Pharmaceuticals Ltd)	Other
8641	DIPYRIDAMOLE 100 MG SUS	Other

73466	Dipyridamole 100mg Tablet (Approved Prescription Services Ltd)	Other
75011	Dipyridamole 100mg Tablet (C P Pharmaceuticals Ltd)	Other
1814	Dipyridamole 100mg tablets	Other
34709	Dipyridamole 100mg tablets (A A H Pharmaceuticals Ltd)	Other
43530	Dipyridamole 100mg tablets (Actavis UK Ltd)	Other
73435	Dipyridamole 100mg tablets (DE Pharmaceuticals)	Other
67403	Dipyridamole 100mg tablets (Dr Reddy's Laboratories (UK) Ltd)	Other
46976	Dipyridamole 100mg tablets (Kent Pharmaceuticals Ltd)	Other
51358	Dipyridamole 100mg tablets (Mylan)	Other
67567	Dipyridamole 100mg/5ml oral solution	Other
35108	Dipyridamole 100mg/5ml oral suspension	Other
16597	Dipyridamole 10mg/2ml solution for injection ampoules	Other
714	Dipyridamole 200mg modified-release capsules	Other
64929	Dipyridamole 200mg/5ml oral suspension	Other
66840	Dipyridamole 200mg/5ml oral suspension sugar free	Other
771	Dipyridamole 25mg tablets	Other
60605	Dipyridamole 25mg tablets (A A H Pharmaceuticals Ltd)	Other
62707	Dipyridamole 25mg tablets (Alliance Healthcare (Distribution) Ltd)	Other
60321	Dipyridamole 25mg tablets (Phoenix Healthcare Distribution Ltd)	Other
64222	Dipyridamole 50mg/5ml oral solution	Other
5882	Dipyridamole 50mg/5ml oral suspension sugar free	Other
72650	Dipyridamole oral liquid	Other
41229	Efient 10mg tablets (Eli Lilly and Company Ltd)	Other
40591	Efient 5mg tablets (Eli Lilly and Company Ltd)	Other
30976	Modaplate 100mg Tablet (Berk Pharmaceuticals Ltd)	Other
30975	Modaplate 25mg Tablet (Berk Pharmaceuticals Ltd)	Other
64837	Ofcram PR 200mg capsules (Focus Pharmaceuticals Ltd)	Other
4277	PERSANTIN 10 MG SUS	Other
10286	PERSANTIN 100 MG SUS	Other
2106	Persantin 100mg tablets (Boehringer Ingelheim Ltd)	Other
17130	Persantin 10mg/2ml solution for injection ampoules (Boehringer Ingelheim Ltd)	Other
689	Persantin 25mg tablets (Boehringer Ingelheim Ltd)	Other
3832	Persantin Retard 200mg capsules (Boehringer Ingelheim Ltd)	Other
59470	Persantin Retard 200mg capsules (Consilient Health Ltd)	Other
39932	Prasugrel 10mg tablets	Other
40114	Prasugrel 5mg tablets	Other
21019	ReoPro 10mg/5ml solution for injection vials (Janssen-Cilag Ltd)	Other
66973	Ticagrelor 60mg tablets	Other
70606	Ticagrelor 90mg orodispersible tablets sugar free	Other
45576	Ticagrelor 90mg tablets	Other
35809	Tirofiban 12.5mg/250ml infusion bags	Other
25232	Vasyrol cold sore 100mg Tablet (Shire Pharmaceuticals Ltd)	Other
31192	Vasyrol cold sore 25mg Tablet (Shire Pharmaceuticals Ltd)	Other
